# Supplementary material for: Separating the effects of early and later life adiposity on colorectal cancer risk: a Mendelian randomization study
Source: BMC Med. 2023 Jan 4;21:5. doi: 10.1186/s12916-022-02702-9 (PMC9814460; doi:10.1186/s12916-022-02702-9)
Supplement: Supplementary file 1 — Additional file 1: Table S1. Beta estimates for genome-wide significant SNPs for early/adult life body size in UK Biobank (overall, men, women). Table S2. Summary information on colorectal cancer risk for the SNPs used in the analysis. Table S3. Number of cancer cases by sex and subsite. Table S4. Sample size and power calculations for each phenotype and group in the Mendelian randomization study of early and adult life body size and risk of colorectal cancer. Table S5. Beta estimates for early/adult life body size in UK Biobank for the SNPs included in the MVMR analysis (overall, men, women). Table S6. STROBE-MR checklist of recommended items to address in reports of Mendelian randomization studies. Table S7. Univariable Mendelian randomization estimates between early and adult body size and colorectal cancer risk. Table S8. Fearly life body size, Fadult body size and Qa in the multivariable MR between early, adult body size and colorectal cancer. Table S9. Multivariable MR Egger analysis to assess the effect of both predicted early life and adult body size on colorectal cancer. [file 12916_2022_2702_MOESM1_ESM.docx]

**Legends of supplementary tables**

**Supplementary Table 1**: Beta estimates for genome-wide significant SNPs for early/adult life body size in UK Biobank (overall, men, women)

**Supplementary Table 2**: Summary information on colorectal cancer risk for the SNPs used in the analysis

**Supplementary Table 3**: Number of cancer cases by sex and subsite

**Supplementary Table 4**: Sample size and power calculations for each phenotype and group in the Mendelian randomization study of early and adult life body size and risk of colorectal cancer

**Supplementary Table 5**: Beta estimates for early/adult life body size in UK Biobank for the SNPs included in the MVMR analysis (overall, men, women)

**Supplementary Table 6**: STROBE-MR checklist of recommended items to address in reports of Mendelian randomization studies

**Supplementary Table 7**: Univariable Mendelian randomization estimates between early and adult body size and colorectal cancer risk

**Supplementary Table 8**: $F_{early life body size}$, $F_{adult body size}$ and $Q_{a}$ in the multivariable MR between early, adult body size and colorectal cancer

**Supplementary Table 9**: Multivariable MR Egger analysis to assess the effect of both predicted early life and adult body size on colorectal cancer

| **Supplementary table 1: Beta estimates for genome-wide significant SNPs for early/adult life body size in UK Biobank (overall, men, women)** | | | | | | | | | | | | | | | | | | | | | | | | | | | | | | | | | | | | | | | | | | | | | | | | | | | | |
| --- | --- | --- | --- | --- | --- | --- | --- | --- | --- | --- | --- | --- | --- | --- | --- | --- | --- | --- | --- | --- | --- | --- | --- | --- | --- | --- | --- | --- | --- | --- | --- | --- | --- | --- | --- | --- | --- | --- | --- | --- | --- | --- | --- | --- | --- | --- | --- | --- | --- | --- | --- | --- |
| Genome-wide significant SNPs for early life body size in UK Biobank (overall) | | | | | | | | | | | | | | | | | | | | | | | | | | | | | | | | | | | | | | | | | | | | | | | | | | | | |
| SNP | Chr | | | Position | | | | Closest gene | | | | | | | | EA | | | OA | | | | EAF | | | | Beta | | | | | SE | | | | | | | pvalue | | | | | | R^2^ | | | | F statistic | | | |
| rs2229330 | 1 | | | 6649228 | | | | ZBTB48 | | | | | | | | T | | | G | | | | 0.93 | | | | -0.020 | | | | | 0.003 | | | | | | | 2.20E-13 | | | | | | 1.19E-04 | | | | 54 | | | |
| rs2175171 | 1 | | | 7028842 | | | | CAMTA1 | | | | | | | | G | | | C | | | | 0.44 | | | | -0.008 | | | | | 0.001 | | | | | | | 3.90E-08 | | | | | | 6.66E-05 | | | | 30 | | | |
| rs6577497 | 1 | | | 8605667 | | | | RERE | | | | | | | | A | | | T | | | | 0.61 | | | | 0.008 | | | | | 0.001 | | | | | | | 1.90E-08 | | | | | | 6.97E-05 | | | | 32 | | | |
| rs12045879 | 1 | | | 15817090 | | | | CELA2B | | | | | | | | C | | | T | | | | 0.69 | | | | 0.010 | | | | | 0.002 | | | | | | | 5.30E-11 | | | | | | 9.51E-05 | | | | 43 | | | |
| rs212517 | 1 | | | 21577159 | | | | ECE1 | | | | | | | | T | | | A | | | | 0.40 | | | | 0.009 | | | | | 0.001 | | | | | | | 8.90E-10 | | | | | | 8.29E-05 | | | | 38 | | | |
| rs2356864 | 1 | | | 50839740 | | | | DMRTA2 | | | | | | | | G | | | A | | | | 0.48 | | | | -0.009 | | | | | 0.001 | | | | | | | 8.00E-10 | | | | | | 8.33E-05 | | | | 38 | | | |
| rs630602 | 1 | | | 54728864 | | | | SSBP3 | | | | | | | | G | | | C | | | | 0.39 | | | | -0.010 | | | | | 0.001 | | | | | | | 1.60E-12 | | | | | | 1.10E-04 | | | | 50 | | | |
| rs12140153 | 1 | | | 62579891 | | | | INADL | | | | | | | | G | | | T | | | | 0.91 | | | | 0.022 | | | | | 0.002 | | | | | | | 6.20E-19 | | | | | | 1.74E-04 | | | | 79 | | | |
| rs2767486 | 1 | | | 65991203 | | | | LEPR | | | | | | | | A | | | G | | | | 0.80 | | | | -0.015 | | | | | 0.002 | | | | | | | 1.10E-18 | | | | | | 1.72E-04 | | | | 78 | | | |
| rs7522014 | 1 | | | 66551759 | | | | PDE4B | | | | | | | | A | | | G | | | | 0.67 | | | | 0.009 | | | | | 0.002 | | | | | | | 8.00E-10 | | | | | | 8.33E-05 | | | | 38 | | | |
| rs2755253 | 1 | | | 67470843 | | | | SLC35D1 | | | | | | | | C | | | T | | | | 0.29 | | | | 0.009 | | | | | 0.002 | | | | | | | 1.60E-08 | | | | | | 7.06E-05 | | | | 32 | | | |
| rs11209943 | 1 | | | 72750500 | | | | NEGR1 | | | | | | | | A | | | G | | | | 0.40 | | | | -0.018 | | | | | 0.001 | | | | | | | 9.70E-38 | | | | | | 3.64E-04 | | | | 165 | | | |
| rs12042908 | 1 | | | 74997762 | | | | TNNI3K | | | | | | | | A | | | G | | | | 0.44 | | | | 0.027 | | | | | 0.001 | | | | | | | 2.30E-84 | | | | | | 8.35E-04 | | | | 379 | | | |
| rs34517439 | 1 | | | 78450517 | | | | DNAJB4 | | | | | | | | C | | | A | | | | 0.88 | | | | -0.014 | | | | | 0.002 | | | | | | | 2.20E-10 | | | | | | 8.89E-05 | | | | 40 | | | |
| rs11165687 | 1 | | | 97099767 | | | | PTBP2 | | | | | | | | C | | | T | | | | 0.59 | | | | -0.008 | | | | | 0.001 | | | | | | | 9.40E-09 | | | | | | 7.27E-05 | | | | 33 | | | |
| rs7550711 | 1 | | | 110082886 | | | | GPR61 | | | | | | | | C | | | T | | | | 0.97 | | | | -0.048 | | | | | 0.004 | | | | | | | 4.40E-28 | | | | | | 2.66E-04 | | | | 121 | | | |
| rs3013431 | 1 | | | 113242488 | | | | MOV10 | | | | | | | | C | | | T | | | | 0.39 | | | | 0.009 | | | | | 0.001 | | | | | | | 1.40E-09 | | | | | | 8.10E-05 | | | | 37 | | | |
| rs12132598 | 1 | | | 115235785 | | | | AMPD1 | | | | | | | | A | | | G | | | | 0.67 | | | | -0.008 | | | | | 0.001 | | | | | | | 2.10E-08 | | | | | | 6.94E-05 | | | | 31 | | | |
| rs7536458 | 1 | | | 118864602 | | | | SPAG17 | | | | | | | | T | | | G | | | | 0.74 | | | | -0.010 | | | | | 0.002 | | | | | | | 1.80E-10 | | | | | | 8.98E-05 | | | | 41 | | | |
| rs11205303 | 1 | | | 149906413 | | | | MTMR11 | | | | | | | | T | | | C | | | | 0.59 | | | | 0.010 | | | | | 0.001 | | | | | | | 1.70E-11 | | | | | | 1.00E-04 | | | | 45 | | | |
| rs35588936 | 1 | | | 155950963 | | | | ARHGEF2 | | | | | | | | C | | | T | | | | 0.94 | | | | 0.018 | | | | | 0.003 | | | | | | | 3.00E-10 | | | | | | 8.76E-05 | | | | 40 | | | |
| rs12748436 | 1 | | | 177761109 | | | | SEC16B | | | | | | | | C | | | G | | | | 0.92 | | | | -0.016 | | | | | 0.003 | | | | | | | 3.50E-09 | | | | | | 7.69E-05 | | | | 35 | | | |
| rs543874 | 1 | | | 177889480 | | | | SEC16B | | | | | | | | A | | | G | | | | 0.79 | | | | -0.047 | | | | | 0.002 | | | | | | | 8.80E-163 | | | | | | 1.63E-03 | | | | 739 | | | |
| rs78444298 | 1 | | | 184672098 | | | | EDEM3 | | | | | | | | G | | | A | | | | 0.98 | | | | 0.042 | | | | | 0.005 | | | | | | | 1.70E-16 | | | | | | 1.50E-04 | | | | 68 | | | |
| rs4074404 | 1 | | | 187683956 | | | | PLA2G4A | | | | | | | | T | | | A | | | | 0.84 | | | | -0.013 | | | | | 0.002 | | | | | | | 4.30E-11 | | | | | | 9.59E-05 | | | | 43 | | | |
| rs16839832 | 1 | | | 196349909 | | | | KCNT2 | | | | | | | | G | | | T | | | | 0.92 | | | | -0.014 | | | | | 0.003 | | | | | | | 3.30E-08 | | | | | | 6.74E-05 | | | | 31 | | | |
| rs9438393 | 1 | | | 205782718 | | | | SLC41A1 | | | | | | | | A | | | G | | | | 0.59 | | | | 0.010 | | | | | 0.001 | | | | | | | 3.10E-13 | | | | | | 1.17E-04 | | | | 53 | | | |
| rs7354849 | 1 | | | 232765308 | | | | SIPA1L2 | | | | | | | | A | | | G | | | | 0.54 | | | | -0.008 | | | | | 0.001 | | | | | | | 7.40E-09 | | | | | | 7.38E-05 | | | | 33 | | | |
| rs62106258 | 2 | | | 417167 | | | | FAM150B | | | | | | | | T | | | C | | | | 0.95 | | | | 0.080 | | | | | 0.003 | | | | | | | 2.40E-134 | | | | | | 1.34E-03 | | | | 609 | | | |
| rs12992672 | 2 | | | 632592 | | | | TMEM18 | | | | | | | | G | | | A | | | | 0.17 | | | | -0.043 | | | | | 0.002 | | | | | | | 5.50E-121 | | | | | | 1.21E-03 | | | | 547 | | | |
| rs2867116 | 2 | | | 682363 | | | | TMEM18 | | | | | | | | C | | | A | | | | 0.86 | | | | -0.012 | | | | | 0.002 | | | | | | | 1.40E-09 | | | | | | 8.09E-05 | | | | 37 | | | |
| rs2141004 | 2 | | | 6194359 | | | | DKFZP761K2322 | | | | | | | | A | | | C | | | | 0.74 | | | | 0.010 | | | | | 0.002 | | | | | | | 2.60E-10 | | | | | | 8.82E-05 | | | | 40 | | | |
| rs10182458 | 2 | | | 25150641 | | | | ADCY3 | | | | | | | | A | | | G | | | | 0.51 | | | | -0.036 | | | | | 0.001 | | | | | | | 3.10E-145 | | | | | | 1.45E-03 | | | | 659 | | | |
| rs146910503 | 2 | | | 25446473 | | | | DNMT3A | | | | | | | | G | | | A | | | | 0.98 | | | | 0.033 | | | | | 0.005 | | | | | | | 2.70E-11 | | | | | | 9.79E-05 | | | | 44 | | | |
| rs6719507 | 2 | | | 29733801 | | | | ALK | | | | | | | | G | | | A | | | | 0.56 | | | | 0.008 | | | | | 0.001 | | | | | | | 2.90E-08 | | | | | | 6.79E-05 | | | | 31 | | | |
| rs62134189 | 2 | | | 45046339 | | | | CAMKMT | | | | | | | | A | | | G | | | | 0.90 | | | | 0.014 | | | | | 0.002 | | | | | | | 1.00E-09 | | | | | | 8.22E-05 | | | | 37 | | | |
| rs2902142 | 2 | | | 58849805 | | | | FANCL | | | | | | | | C | | | T | | | | 0.63 | | | | 0.009 | | | | | 0.001 | | | | | | | 1.80E-09 | | | | | | 7.99E-05 | | | | 36 | | | |
| rs2539692 | 2 | | | 59841988 | | | | BCL11A | | | | | | | | T | | | A | | | | 0.39 | | | | 0.008 | | | | | 0.001 | | | | | | | 1.80E-08 | | | | | | 7.01E-05 | | | | 32 | | | |
| rs1177279 | 2 | | | 61295122 | | | | KIAA1841 | | | | | | | | A | | | G | | | | 0.28 | | | | 0.009 | | | | | 0.002 | | | | | | | 1.40E-08 | | | | | | 7.10E-05 | | | | 32 | | | |
| rs7565437 | 2 | | | 65646966 | | | | SPRED2 | | | | | | | | T | | | C | | | | 0.59 | | | | 0.008 | | | | | 0.001 | | | | | | | 9.50E-09 | | | | | | 7.27E-05 | | | | 33 | | | |
| rs12713889 | 2 | | | 77225361 | | | | LRRTM4 | | | | | | | | T | | | C | | | | 0.67 | | | | 0.012 | | | | | 0.001 | | | | | | | 3.70E-15 | | | | | | 1.36E-04 | | | | 62 | | | |
| rs772175 | 2 | | | 96944553 | | | | SNRNP200 | | | | | | | | G | | | A | | | | 0.66 | | | | 0.008 | | | | | 0.001 | | | | | | | 5.00E-08 | | | | | | 6.56E-05 | | | | 30 | | | |
| rs1384660 | 2 | | | 142299735 | | | | LRP1B | | | | | | | | G | | | A | | | | 0.81 | | | | 0.016 | | | | | 0.002 | | | | | | | 1.90E-18 | | | | | | 1.69E-04 | | | | 77 | | | |
| rs62175963 | 2 | | | 161339964 | | | | RBMS1 | | | | | | | | T | | | C | | | | 0.58 | | | | 0.009 | | | | | 0.001 | | | | | | | 2.20E-10 | | | | | | 8.88E-05 | | | | 40 | | | |
| rs115319174 | 2 | | | 207066474 | | | | GPR1 | | | | | | | | G | | | C | | | | 0.94 | | | | -0.042 | | | | | 0.003 | | | | | | | 4.00E-44 | | | | | | 4.28E-04 | | | | 194 | | | |
| rs11891707 | 2 | | | 207120604 | | | | ZDBF2 | | | | | | | | T | | | C | | | | 0.86 | | | | 0.012 | | | | | 0.002 | | | | | | | 3.50E-09 | | | | | | 7.70E-05 | | | | 35 | | | |
| rs3791478 | 2 | | | 240064139 | | | | HDAC4 | | | | | | | | T | | | C | | | | 0.89 | | | | 0.012 | | | | | 0.002 | | | | | | | 3.50E-08 | | | | | | 6.71E-05 | | | | 30 | | | |
| rs1476698 | 2 | | | 242296449 | | | | FARP2 | | | | | | | | A | | | G | | | | 0.63 | | | | 0.008 | | | | | 0.001 | | | | | | | 1.50E-08 | | | | | | 7.08E-05 | | | | 32 | | | |
| rs2594994 | 3 | | | 11339960 | | | | ATG7 | | | | | | | | T | | | A | | | | 0.18 | | | | 0.015 | | | | | 0.002 | | | | | | | 2.50E-17 | | | | | | 1.58E-04 | | | | 72 | | | |
| rs7619139 | 3 | | | 25110415 | | | | RARB | | | | | | | | T | | | A | | | | 0.41 | | | | -0.010 | | | | | 0.001 | | | | | | | 6.20E-12 | | | | | | 1.04E-04 | | | | 47 | | | |
| rs1402989 | 3 | | | 27056851 | | | | NEK10 | | | | | | | | C | | | T | | | | 0.51 | | | | -0.008 | | | | | 0.001 | | | | | | | 2.60E-08 | | | | | | 6.84E-05 | | | | 31 | | | |
| rs2268762 | 3 | | | 38516075 | | | | ACVR2B | | | | | | | | A | | | G | | | | 0.39 | | | | -0.008 | | | | | 0.001 | | | | | | | 2.50E-08 | | | | | | 6.85E-05 | | | | 31 | | | |
| rs754635 | 3 | | | 42305131 | | | | CCK | | | | | | | | C | | | G | | | | 0.11 | | | | -0.016 | | | | | 0.002 | | | | | | | 5.50E-13 | | | | | | 1.15E-04 | | | | 52 | | | |
| rs2034963 | 3 | | | 48170802 | | | | CDC25A | | | | | | | | G | | | C | | | | 0.35 | | | | 0.011 | | | | | 0.001 | | | | | | | 6.50E-14 | | | | | | 1.24E-04 | | | | 56 | | | |
| rs35926495 | 3 | | | 50255663 | | | | GNAI2 | | | | | | | | C | | | T | | | | 0.62 | | | | -0.009 | | | | | 0.001 | | | | | | | 4.80E-10 | | | | | | 8.55E-05 | | | | 39 | | | |
| rs3774604 | 3 | | | 53824136 | | | | CACNA1D | | | | | | | | C | | | T | | | | 0.63 | | | | 0.008 | | | | | 0.001 | | | | | | | 4.60E-08 | | | | | | 6.59E-05 | | | | 30 | | | |
| rs2629881 | 3 | | | 59778271 | | | | FHIT | | | | | | | | C | | | T | | | | 0.22 | | | | -0.011 | | | | | 0.002 | | | | | | | 3.50E-10 | | | | | | 8.69E-05 | | | | 39 | | | |
| rs538579 | 3 | | | 62711674 | | | | CADPS | | | | | | | | G | | | C | | | | 0.69 | | | | -0.008 | | | | | 0.002 | | | | | | | 2.50E-08 | | | | | | 6.86E-05 | | | | 31 | | | |
| rs115903965 | 3 | | | 66009529 | | | | MAGI1 | | | | | | | | G | | | A | | | | 0.98 | | | | -0.026 | | | | | 0.005 | | | | | | | 1.60E-08 | | | | | | 7.04E-05 | | | | 32 | | | |
| rs4677156 | 3 | | | 72417857 | | | | RYBP | | | | | | | | A | | | T | | | | 0.23 | | | | 0.009 | | | | | 0.002 | | | | | | | 3.70E-08 | | | | | | 6.68E-05 | | | | 30 | | | |
| rs1666132 | 3 | | | 77649190 | | | | ROBO2 | | | | | | | | C | | | T | | | | 0.42 | | | | 0.008 | | | | | 0.001 | | | | | | | 6.90E-09 | | | | | | 7.41E-05 | | | | 34 | | | |
| rs1357798 | 3 | | | 83780583 | | | | CADM2 | | | | | | | | C | | | T | | | | 0.21 | | | | -0.009 | | | | | 0.002 | | | | | | | 4.20E-08 | | | | | | 6.63E-05 | | | | 30 | | | |
| rs6783281 | 3 | | | 84791851 | | | | CADM2 | | | | | | | | A | | | G | | | | 0.71 | | | | -0.010 | | | | | 0.002 | | | | | | | 5.70E-10 | | | | | | 8.47E-05 | | | | 38 | | | |
| rs7355953 | 3 | | | 85792137 | | | | CADM2 | | | | | | | | T | | | C | | | | 0.78 | | | | -0.015 | | | | | 0.002 | | | | | | | 6.60E-19 | | | | | | 1.74E-04 | | | | 79 | | | |
| rs2735556 | 3 | | | 88105360 | | | | CGGBP1 | | | | | | | | T | | | C | | | | 0.89 | | | | 0.017 | | | | | 0.002 | | | | | | | 4.80E-15 | | | | | | 1.35E-04 | | | | 61 | | | |
| rs11925138 | 3 | | | 131626048 | | | | CPNE4 | | | | | | | | G | | | A | | | | 0.90 | | | | 0.013 | | | | | 0.002 | | | | | | | 3.10E-08 | | | | | | 6.76E-05 | | | | 31 | | | |
| rs7625768 | 3 | | | 131774642 | | | | CPNE4 | | | | | | | | G | | | A | | | | 0.68 | | | | -0.010 | | | | | 0.002 | | | | | | | 1.30E-11 | | | | | | 1.01E-04 | | | | 46 | | | |
| rs1199333 | 3 | | | 138091701 | | | | MRAS | | | | | | | | G | | | T | | | | 0.18 | | | | 0.014 | | | | | 0.002 | | | | | | | 6.10E-14 | | | | | | 1.24E-04 | | | | 56 | | | |
| rs59714050 | 3 | | | 141267294 | | | | RASA2 | | | | | | | | T | | | A | | | | 0.93 | | | | -0.023 | | | | | 0.003 | | | | | | | 5.20E-16 | | | | | | 1.45E-04 | | | | 66 | | | |
| rs355748 | 3 | | | 153964496 | | | | ARHGEF26 | | | | | | | | G | | | T | | | | 0.62 | | | | -0.010 | | | | | 0.001 | | | | | | | 7.30E-12 | | | | | | 1.04E-04 | | | | 47 | | | |
| rs7633995 | 3 | | | 180369750 | | | | CCDC39 | | | | | | | | A | | | G | | | | 0.90 | | | | -0.014 | | | | | 0.002 | | | | | | | 4.70E-09 | | | | | | 7.57E-05 | | | | 34 | | | |
| rs10937241 | 3 | | | 185822774 | | | | ETV5 | | | | | | | | A | | | G | | | | 0.16 | | | | -0.011 | | | | | 0.002 | | | | | | | 1.10E-08 | | | | | | 7.21E-05 | | | | 33 | | | |
| rs34811474 | 4 | | | 25408838 | | | | ANAPC4 | | | | | | | | G | | | A | | | | 0.77 | | | | 0.010 | | | | | 0.002 | | | | | | | 8.80E-10 | | | | | | 8.29E-05 | | | | 38 | | | |
| rs7656673 | 4 | | | 30840331 | | | | PCDH7 | | | | | | | | A | | | G | | | | 0.60 | | | | -0.012 | | | | | 0.001 | | | | | | | 6.80E-16 | | | | | | 1.44E-04 | | | | 65 | | | |
| rs34722008 | 4 | | | 38659594 | | | | AC021860.1 | | | | | | | | G | | | A | | | | 0.65 | | | | 0.008 | | | | | 0.001 | | | | | | | 1.60E-08 | | | | | | 7.04E-05 | | | | 32 | | | |
| rs7439324 | 4 | | | 44501351 | | | | KCTD8 | | | | | | | | C | | | T | | | | 0.84 | | | | 0.011 | | | | | 0.002 | | | | | | | 1.40E-08 | | | | | | 7.10E-05 | | | | 32 | | | |
| rs12641981 | 4 | | | 45179883 | | | | GNPDA2 | | | | | | | | C | | | T | | | | 0.57 | | | | -0.022 | | | | | 0.001 | | | | | | | 2.20E-55 | | | | | | 5.42E-04 | | | | 246 | | | |
| rs788858 | 4 | | | 82138300 | | | | PRKG2 | | | | | | | | A | | | G | | | | 0.71 | | | | 0.012 | | | | | 0.002 | | | | | | | 1.50E-15 | | | | | | 1.40E-04 | | | | 64 | | | |
| rs7377083 | 4 | | | 102708997 | | | | BANK1 | | | | | | | | C | | | A | | | | 0.57 | | | | -0.014 | | | | | 0.001 | | | | | | | 6.40E-22 | | | | | | 2.04E-04 | | | | 93 | | | |
| rs72675820 | 4 | | | 130746149 | | | | C4orf33 | | | | | | | | A | | | C | | | | 0.36 | | | | 0.010 | | | | | 0.001 | | | | | | | 1.00E-11 | | | | | | 1.02E-04 | | | | 46 | | | |
| rs35189091 | 4 | | | 137147696 | | | | PCDH18 | | | | | | | | A | | | G | | | | 0.63 | | | | 0.009 | | | | | 0.001 | | | | | | | 5.50E-10 | | | | | | 8.49E-05 | | | | 38 | | | |
| rs11727676 | 4 | | | 145659064 | | | | HHIP | | | | | | | | T | | | C | | | | 0.90 | | | | 0.013 | | | | | 0.002 | | | | | | | 3.20E-08 | | | | | | 6.75E-05 | | | | 31 | | | |
| rs3936511 | 5 | | | 55860781 | | | | AC022431.2 | | | | | | | | A | | | G | | | | 0.81 | | | | 0.011 | | | | | 0.002 | | | | | | | 1.10E-09 | | | | | | 8.19E-05 | | | | 37 | | | |
| rs6449532 | 5 | | | 60715446 | | | | ZSWIM6 | | | | | | | | C | | | T | | | | 0.64 | | | | 0.011 | | | | | 0.001 | | | | | | | 3.30E-14 | | | | | | 1.27E-04 | | | | 58 | | | |
| rs9291816 | 5 | | | 63932508 | | | | RGS7BP | | | | | | | | C | | | T | | | | 0.68 | | | | 0.013 | | | | | 0.001 | | | | | | | 3.20E-19 | | | | | | 1.77E-04 | | | | 80 | | | |
| rs39862 | 5 | | | 66185151 | | | | MAST4 | | | | | | | | T | | | C | | | | 0.72 | | | | 0.012 | | | | | 0.002 | | | | | | | 7.40E-14 | | | | | | 1.23E-04 | | | | 56 | | | |
| rs2307111 | 5 | | | 75003678 | | | | POC5 | | | | | | | | T | | | C | | | | 0.60 | | | | 0.009 | | | | | 0.001 | | | | | | | 1.10E-09 | | | | | | 8.20E-05 | | | | 37 | | | |
| rs1422067 | 5 | | | 77424836 | | | | AP3B1 | | | | | | | | C | | | T | | | | 0.76 | | | | 0.011 | | | | | 0.002 | | | | | | | 4.60E-11 | | | | | | 9.56E-05 | | | | 43 | | | |
| rs2115885 | 5 | | | 87598818 | | | | TMEM161B | | | | | | | | G | | | A | | | | 0.79 | | | | 0.011 | | | | | 0.002 | | | | | | | 1.00E-09 | | | | | | 8.23E-05 | | | | 37 | | | |
| rs77960 | 5 | | | 103964585 | | | | NUDT12 | | | | | | | | G | | | A | | | | 0.67 | | | | 0.010 | | | | | 0.001 | | | | | | | 2.20E-12 | | | | | | 1.09E-04 | | | | 49 | | | |
| rs4958568 | 5 | | | 152016093 | | | | NMUR2 | | | | | | | | G | | | A | | | | 0.72 | | | | 0.010 | | | | | 0.002 | | | | | | | 2.20E-10 | | | | | | 8.88E-05 | | | | 40 | | | |
| rs7719067 | 5 | | | 153538241 | | | | MFAP3 | | | | | | | | A | | | G | | | | 0.43 | | | | 0.013 | | | | | 0.001 | | | | | | | 1.40E-21 | | | | | | 2.01E-04 | | | | 91 | | | |
| rs7711823 | 5 | | | 158489315 | | | | EBF1 | | | | | | | | A | | | G | | | | 0.64 | | | | 0.008 | | | | | 0.001 | | | | | | | 9.00E-09 | | | | | | 7.29E-05 | | | | 33 | | | |
| rs918472 | 5 | | | 170738836 | | | | TLX3 | | | | | | | | G | | | A | | | | 0.28 | | | | -0.010 | | | | | 0.002 | | | | | | | 2.60E-10 | | | | | | 8.82E-05 | | | | 40 | | | |
| rs12214497 | 6 | | | 10015908 | | | | OFCC1 | | | | | | | | G | | | T | | | | 0.65 | | | | 0.011 | | | | | 0.001 | | | | | | | 9.70E-13 | | | | | | 1.12E-04 | | | | 51 | | | |
| rs10498713 | 6 | | | 22729300 | | | | HDGFL1 | | | | | | | | G | | | T | | | | 0.85 | | | | -0.012 | | | | | 0.002 | | | | | | | 2.10E-09 | | | | | | 7.91E-05 | | | | 36 | | | |
| rs35162296 | 6 | | | 26318262 | | | | HIST1H4H | | | | | | | | C | | | T | | | | 0.89 | | | | -0.020 | | | | | 0.002 | | | | | | | 1.20E-18 | | | | | | 1.72E-04 | | | | 78 | | | |
| rs34196306 | 6 | | | 27425644 | | | | ZNF184 | | | | | | | | G | | | C | | | | 0.89 | | | | -0.020 | | | | | 0.002 | | | | | | | 2.60E-18 | | | | | | 1.68E-04 | | | | 76 | | | |
| rs3131336 | 6 | | | 28831611 | | | | TRIM27 | | | | | | | | C | | | T | | | | 0.88 | | | | -0.019 | | | | | 0.002 | | | | | | | 2.10E-18 | | | | | | 1.69E-04 | | | | 77 | | | |
| rs3129942 | 6 | | | 32338283 | | | | C6orf10 | | | | | | | | G | | | T | | | | 0.74 | | | | -0.014 | | | | | 0.002 | | | | | | | 2.90E-18 | | | | | | 1.68E-04 | | | | 76 | | | |
| rs9366803 | 6 | | | 32621132 | | | | HLA-DQB1 | | | | | | | | T | | | C | | | | 0.68 | | | | -0.011 | | | | | 0.002 | | | | | | | 1.10E-10 | | | | | | 9.17E-05 | | | | 42 | | | |
| rs686431 | 6 | | | 35974217 | | | | SLC26A8 | | | | | | | | C | | | T | | | | 0.98 | | | | -0.030 | | | | | 0.005 | | | | | | | 6.70E-09 | | | | | | 7.42E-05 | | | | 34 | | | |
| rs73422097 | 6 | | | 41727740 | | | | PGC | | | | | | | | A | | | G | | | | 0.70 | | | | -0.011 | | | | | 0.002 | | | | | | | 3.10E-12 | | | | | | 1.07E-04 | | | | 49 | | | |
| rs76187039 | 6 | | | 43233990 | | | | TTBK1 | | | | | | | | G | | | T | | | | 0.87 | | | | -0.011 | | | | | 0.002 | | | | | | | 3.40E-08 | | | | | | 6.72E-05 | | | | 30 | | | |
| rs3798519 | 6 | | | 50788778 | | | | TFAP2B | | | | | | | | A | | | C | | | | 0.82 | | | | -0.025 | | | | | 0.002 | | | | | | | 9.70E-43 | | | | | | 4.14E-04 | | | | 188 | | | |
| rs1775255 | 6 | | | 51243035 | | | | PKHD1 | | | | | | | | G | | | T | | | | 0.52 | | | | -0.013 | | | | | 0.001 | | | | | | | 2.30E-21 | | | | | | 1.99E-04 | | | | 90 | | | |
| rs1342831 | 6 | | | 54096151 | | | | MLIP | | | | | | | | T | | | C | | | | 0.94 | | | | -0.022 | | | | | 0.003 | | | | | | | 8.70E-14 | | | | | | 1.23E-04 | | | | 56 | | | |
| rs12110721 | 6 | | | 55190480 | | | | GFRAL | | | | | | | | G | | | A | | | | 0.83 | | | | -0.019 | | | | | 0.002 | | | | | | | 1.60E-23 | | | | | | 2.20E-04 | | | | 100 | | | |
| rs9370527 | 6 | | | 56245812 | | | | COL21A1 | | | | | | | | G | | | A | | | | 0.76 | | | | -0.009 | | | | | 0.002 | | | | | | | 6.90E-09 | | | | | | 7.41E-05 | | | | 34 | | | |
| rs435775 | 6 | | | 97237609 | | | | GPR63 | | | | | | | | A | | | G | | | | 0.25 | | | | -0.010 | | | | | 0.002 | | | | | | | 5.20E-10 | | | | | | 8.51E-05 | | | | 39 | | | |
| rs6931604 | 6 | | | 98578215 | | | | POU3F2 | | | | | | | | C | | | T | | | | 0.40 | | | | -0.008 | | | | | 0.001 | | | | | | | 6.20E-09 | | | | | | 7.45E-05 | | | | 34 | | | |
| rs34260097 | 6 | | | 100727703 | | | | SIM1 | | | | | | | | T | | | G | | | | 0.78 | | | | -0.018 | | | | | 0.002 | | | | | | | 2.50E-26 | | | | | | 2.49E-04 | | | | 113 | | | |
| rs7759938 | 6 | | | 105378954 | | | | LIN28B | | | | | | | | C | | | T | | | | 0.32 | | | | -0.010 | | | | | 0.001 | | | | | | | 3.20E-11 | | | | | | 9.71E-05 | | | | 44 | | | |
| rs7753558 | 6 | | | 117523471 | | | | VGLL2 | | | | | | | | C | | | A | | | | 0.37 | | | | 0.009 | | | | | 0.001 | | | | | | | 5.10E-10 | | | | | | 8.53E-05 | | | | 39 | | | |
| rs1452991 | 6 | | | 141473363 | | | | NMBR | | | | | | | | G | | | A | | | | 0.63 | | | | -0.010 | | | | | 0.001 | | | | | | | 5.40E-12 | | | | | | 1.05E-04 | | | | 48 | | | |
| rs796915 | 6 | | | 154304628 | | | | OPRM1 | | | | | | | | C | | | G | | | | 0.31 | | | | -0.013 | | | | | 0.002 | | | | | | | 2.60E-17 | | | | | | 1.58E-04 | | | | 72 | | | |
| rs62425398 | 6 | | | 166416028 | | | | PDE10A | | | | | | | | C | | | A | | | | 0.89 | | | | -0.015 | | | | | 0.002 | | | | | | | 1.90E-11 | | | | | | 9.94E-05 | | | | 45 | | | |
| rs2349179 | 7 | | | 8404389 | | | | NXPH1 | | | | | | | | T | | | A | | | | 0.62 | | | | -0.008 | | | | | 0.001 | | | | | | | 5.00E-08 | | | | | | 6.56E-05 | | | | 30 | | | |
| rs2722406 | 7 | | | 24306762 | | | | NPY | | | | | | | | C | | | T | | | | 0.72 | | | | -0.012 | | | | | 0.002 | | | | | | | 4.60E-15 | | | | | | 1.36E-04 | | | | 61 | | | |
| rs4723263 | 7 | | | 33194826 | | | | BBS9 | | | | | | | | G | | | C | | | | 0.57 | | | | -0.008 | | | | | 0.001 | | | | | | | 3.60E-09 | | | | | | 7.69E-05 | | | | 35 | | | |
| rs10234366 | 7 | | | 46743746 | | | | AC011294.3 | | | | | | | | G | | | A | | | | 0.90 | | | | -0.014 | | | | | 0.002 | | | | | | | 1.10E-09 | | | | | | 8.18E-05 | | | | 37 | | | |
| rs1852006 | 7 | | | 77829768 | | | | MAGI2 | | | | | | | | G | | | A | | | | 0.64 | | | | 0.009 | | | | | 0.001 | | | | | | | 1.70E-09 | | | | | | 8.00E-05 | | | | 36 | | | |
| rs6974282 | 7 | | | 100098295 | | | | NYAP1 | | | | | | | | C | | | T | | | | 0.80 | | | | 0.010 | | | | | 0.002 | | | | | | | 1.50E-08 | | | | | | 7.06E-05 | | | | 32 | | | |
| rs7808296 | 7 | | | 103127620 | | | | RELN | | | | | | | | C | | | T | | | | 0.68 | | | | -0.010 | | | | | 0.002 | | | | | | | 2.80E-11 | | | | | | 9.78E-05 | | | | 44 | | | |
| rs262338 | 7 | | | 103417134 | | | | RELN | | | | | | | | G | | | T | | | | 0.55 | | | | -0.009 | | | | | 0.001 | | | | | | | 4.80E-10 | | | | | | 8.55E-05 | | | | 39 | | | |
| rs10953577 | 7 | | | 108263540 | | | | DNAJB9 | | | | | | | | T | | | C | | | | 0.62 | | | | -0.009 | | | | | 0.001 | | | | | | | 3.40E-09 | | | | | | 7.71E-05 | | | | 35 | | | |
| rs67679818 | 7 | | | 110672704 | | | | IMMP2L | | | | | | | | C | | | T | | | | 0.42 | | | | 0.008 | | | | | 0.001 | | | | | | | 4.70E-08 | | | | | | 6.58E-05 | | | | 30 | | | |
| rs6979832 | 7 | | | 127856276 | | | | LEP | | | | | | | | A | | | G | | | | 0.50 | | | | -0.009 | | | | | 0.001 | | | | | | | 2.70E-11 | | | | | | 9.79E-05 | | | | 44 | | | |
| rs11525873 | 7 | | | 138817193 | | | | TTC26 | | | | | | | | T | | | C | | | | 0.90 | | | | 0.017 | | | | | 0.002 | | | | | | | 1.60E-13 | | | | | | 1.20E-04 | | | | 54 | | | |
| rs10503246 | 8 | | | 4130363 | | | | CSMD1 | | | | | | | | A | | | G | | | | 0.71 | | | | -0.010 | | | | | 0.002 | | | | | | | 4.50E-10 | | | | | | 8.58E-05 | | | | 39 | | | |
| rs77976727 | 8 | | | 4300554 | | | | CSMD1 | | | | | | | | C | | | T | | | | 0.91 | | | | -0.015 | | | | | 0.002 | | | | | | | 3.10E-10 | | | | | | 8.74E-05 | | | | 40 | | | |
| rs7814267 | 8 | | | 5545084 | | | | CSMD1 | | | | | | | | A | | | G | | | | 0.81 | | | | -0.011 | | | | | 0.002 | | | | | | | 2.30E-09 | | | | | | 7.87E-05 | | | | 36 | | | |
| rs11777719 | 8 | | | 8581038 | | | | CLDN23 | | | | | | | | A | | | G | | | | 0.72 | | | | -0.012 | | | | | 0.002 | | | | | | | 8.50E-14 | | | | | | 1.23E-04 | | | | 56 | | | |
| rs13256357 | 8 | | | 9979535 | | | | MSRA | | | | | | | | C | | | T | | | | 0.80 | | | | -0.014 | | | | | 0.002 | | | | | | | 6.30E-15 | | | | | | 1.34E-04 | | | | 61 | | | |
| rs2409743 | 8 | | | 11070360 | | | | XKR6 | | | | | | | | C | | | G | | | | 0.50 | | | | 0.011 | | | | | 0.001 | | | | | | | 3.00E-15 | | | | | | 1.37E-04 | | | | 62 | | | |
| rs10503555 | 8 | | | 15763818 | | | | TUSC3 | | | | | | | | A | | | G | | | | 0.56 | | | | 0.008 | | | | | 0.001 | | | | | | | 3.90E-08 | | | | | | 6.66E-05 | | | | 30 | | | |
| rs884152 | 8 | | | 25770557 | | | | EBF2 | | | | | | | | G | | | T | | | | 0.36 | | | | -0.009 | | | | | 0.001 | | | | | | | 6.00E-09 | | | | | | 7.47E-05 | | | | 34 | | | |
| rs7012648 | 8 | | | 28091482 | | | | ELP3 | | | | | | | | G | | | A | | | | 0.41 | | | | -0.010 | | | | | 0.001 | | | | | | | 8.40E-12 | | | | | | 1.03E-04 | | | | 47 | | | |
| rs4739558 | 8 | | | 38337264 | | | | FGFR1 | | | | | | | | A | | | G | | | | 0.40 | | | | 0.008 | | | | | 0.001 | | | | | | | 6.60E-09 | | | | | | 7.43E-05 | | | | 34 | | | |
| rs10095724 | 8 | | | 53739232 | | | | RB1CC1 | | | | | | | | G | | | A | | | | 0.64 | | | | 0.009 | | | | | 0.001 | | | | | | | 1.40E-10 | | | | | | 9.10E-05 | | | | 41 | | | |
| rs10111937 | 8 | | | 54160092 | | | | OPRK1 | | | | | | | | C | | | T | | | | 0.70 | | | | -0.008 | | | | | 0.002 | | | | | | | 4.20E-08 | | | | | | 6.63E-05 | | | | 30 | | | |
| rs7840305 | 8 | | | 57168101 | | | | CHCHD7 | | | | | | | | A | | | G | | | | 0.63 | | | | 0.008 | | | | | 0.001 | | | | | | | 2.20E-08 | | | | | | 6.92E-05 | | | | 31 | | | |
| rs13254613 | 8 | | | 64804804 | | | | YTHDF3 | | | | | | | | A | | | C | | | | 0.65 | | | | -0.013 | | | | | 0.001 | | | | | | | 1.60E-17 | | | | | | 1.60E-04 | | | | 73 | | | |
| rs7817581 | 8 | | | 143136114 | | | | TSNARE1 | | | | | | | | G | | | A | | | | 0.67 | | | | 0.009 | | | | | 0.001 | | | | | | | 6.80E-09 | | | | | | 7.41E-05 | | | | 34 | | | |
| rs10962279 | 9 | | | 16103030 | | | | CCDC171 | | | | | | | | T | | | C | | | | 0.78 | | | | -0.010 | | | | | 0.002 | | | | | | | 1.70E-09 | | | | | | 8.00E-05 | | | | 36 | | | |
| rs3118252 | 9 | | | 25115154 | | | | TUSC1 | | | | | | | | G | | | C | | | | 0.42 | | | | -0.008 | | | | | 0.001 | | | | | | | 6.10E-09 | | | | | | 7.46E-05 | | | | 34 | | | |
| rs1935354 | 9 | | | 27822531 | | | | LINGO2 | | | | | | | | T | | | C | | | | 0.52 | | | | -0.010 | | | | | 0.001 | | | | | | | 5.60E-13 | | | | | | 1.15E-04 | | | | 52 | | | |
| rs1619120 | 9 | | | 87302196 | | | | NTRK2 | | | | | | | | A | | | G | | | | 0.40 | | | | -0.008 | | | | | 0.001 | | | | | | | 1.80E-08 | | | | | | 7.00E-05 | | | | 32 | | | |
| rs4744246 | 9 | | | 96254464 | | | | FAM120A | | | | | | | | A | | | G | | | | 0.66 | | | | -0.016 | | | | | 0.001 | | | | | | | 1.70E-26 | | | | | | 2.50E-04 | | | | 113 | | | |
| rs7020564 | 9 | | | 109670016 | | | | ZNF462 | | | | | | | | A | | | T | | | | 0.70 | | | | 0.010 | | | | | 0.002 | | | | | | | 1.40E-10 | | | | | | 9.10E-05 | | | | 41 | | | |
| rs957512 | 9 | | | 120405705 | | | | TLR4 | | | | | | | | T | | | C | | | | 0.67 | | | | 0.010 | | | | | 0.001 | | | | | | | 4.70E-12 | | | | | | 1.05E-04 | | | | 48 | | | |
| rs10116891 | 9 | | | 122651993 | | | | RP11-295D22.1 | | | | | | | | G | | | A | | | | 0.90 | | | | -0.014 | | | | | 0.002 | | | | | | | 5.60E-09 | | | | | | 7.49E-05 | | | | 34 | | | |
| rs2275241 | 9 | | | 129370576 | | | | LMX1B | | | | | | | | G | | | A | | | | 0.63 | | | | -0.010 | | | | | 0.001 | | | | | | | 6.30E-13 | | | | | | 1.14E-04 | | | | 52 | | | |
| rs117911387 | 9 | | | 130446836 | | | | STXBP1 | | | | | | | | G | | | A | | | | 0.95 | | | | -0.025 | | | | | 0.003 | | | | | | | 1.60E-13 | | | | | | 1.20E-04 | | | | 54 | | | |
| rs7084503 | 10 | | | 2666859 | | | | PFKP | | | | | | | | T | | | C | | | | 0.49 | | | | 0.013 | | | | | 0.001 | | | | | | | 5.70E-20 | | | | | | 1.85E-04 | | | | 84 | | | |
| rs11256627 | 10 | | | 10535954 | | | | CELF2 | | | | | | | | G | | | A | | | | 0.30 | | | | -0.009 | | | | | 0.002 | | | | | | | 9.30E-09 | | | | | | 7.28E-05 | | | | 33 | | | |
| rs4572029 | 10 | | | 70889053 | | | | VPS26A | | | | | | | | A | | | G | | | | 0.79 | | | | 0.011 | | | | | 0.002 | | | | | | | 2.00E-10 | | | | | | 8.93E-05 | | | | 40 | | | |
| rs10823504 | 10 | | | 72034062 | | | | NPFFR1 | | | | | | | | G | | | A | | | | 0.94 | | | | 0.016 | | | | | 0.003 | | | | | | | 3.70E-08 | | | | | | 6.68E-05 | | | | 30 | | | |
| rs2242258 | 10 | | | 75607168 | | | | CAMK2G | | | | | | | | T | | | C | | | | 0.74 | | | | -0.009 | | | | | 0.002 | | | | | | | 8.80E-09 | | | | | | 7.30E-05 | | | | 33 | | | |
| rs17399739 | 10 | | | 87490850 | | | | GRID1 | | | | | | | | A | | | G | | | | 0.93 | | | | -0.021 | | | | | 0.003 | | | | | | | 3.80E-14 | | | | | | 1.26E-04 | | | | 57 | | | |
| rs10887571 | 10 | | | 88030441 | | | | GRID1 | | | | | | | | C | | | T | | | | 0.55 | | | | -0.008 | | | | | 0.001 | | | | | | | 1.80E-08 | | | | | | 6.99E-05 | | | | 32 | | | |
| rs41310284 | 10 | | | 102447647 | | | | PAX2 | | | | | | | | C | | | A | | | | 0.90 | | | | 0.021 | | | | | 0.002 | | | | | | | 1.40E-18 | | | | | | 1.71E-04 | | | | 77 | | | |
| rs75387636 | 10 | | | 120278394 | | | | PRLHR | | | | | | | | G | | | A | | | | 0.96 | | | | -0.021 | | | | | 0.004 | | | | | | | 1.50E-09 | | | | | | 8.07E-05 | | | | 37 | | | |
| rs2939931 | 10 | | | 121636406 | | | | MCMBP | | | | | | | | T | | | C | | | | 0.52 | | | | -0.008 | | | | | 0.001 | | | | | | | 1.90E-08 | | | | | | 6.96E-05 | | | | 32 | | | |
| rs1061072 | 10 | | | 126684796 | | | | CTBP2 | | | | | | | | G | | | A | | | | 0.89 | | | | 0.013 | | | | | 0.002 | | | | | | | 2.40E-08 | | | | | | 6.88E-05 | | | | 31 | | | |
| rs56133711 | 11 | | | 27723334 | | | | BDNF | | | | | | | | G | | | A | | | | 0.74 | | | | -0.016 | | | | | 0.002 | | | | | | | 3.30E-24 | | | | | | 2.27E-04 | | | | 103 | | | |
| rs4267058 | 11 | | | 28645584 | | | | METTL15 | | | | | | | | T | | | C | | | | 0.63 | | | | 0.009 | | | | | 0.001 | | | | | | | 1.30E-09 | | | | | | 8.12E-05 | | | | 37 | | | |
| rs661878 | 11 | | | 29188691 | | | | METTL15 | | | | | | | | A | | | G | | | | 0.87 | | | | 0.014 | | | | | 0.002 | | | | | | | 2.80E-11 | | | | | | 9.78E-05 | | | | 44 | | | |
| rs3181269 | 11 | | | 33755956 | | | | CD59 | | | | | | | | C | | | T | | | | 0.75 | | | | 0.009 | | | | | 0.002 | | | | | | | 5.30E-09 | | | | | | 7.52E-05 | | | | 34 | | | |
| rs7951870 | 11 | | | 46373311 | | | | DGKZ | | | | | | | | T | | | C | | | | 0.83 | | | | -0.012 | | | | | 0.002 | | | | | | | 5.30E-10 | | | | | | 8.51E-05 | | | | 39 | | | |
| rs12798028 | 11 | | | 47604639 | | | | NDUFS3 | | | | | | | | C | | | T | | | | 0.59 | | | | -0.015 | | | | | 0.001 | | | | | | | 1.10E-24 | | | | | | 2.32E-04 | | | | 105 | | | |
| rs3862342 | 11 | | | 49357043 | | | | FOLH1 | | | | | | | | C | | | T | | | | 0.29 | | | | -0.009 | | | | | 0.002 | | | | | | | 3.00E-08 | | | | | | 6.77E-05 | | | | 31 | | | |
| rs2958542 | 11 | | | 62181882 | | | | SCGB1A1 | | | | | | | | C | | | T | | | | 0.63 | | | | 0.008 | | | | | 0.001 | | | | | | | 1.80E-08 | | | | | | 7.00E-05 | | | | 32 | | | |
| rs10791902 | 11 | | | 67093360 | | | | SSH3 | | | | | | | | C | | | T | | | | 0.59 | | | | -0.008 | | | | | 0.001 | | | | | | | 3.60E-08 | | | | | | 6.70E-05 | | | | 30 | | | |
| rs10896348 | 11 | | | 68357368 | | | | PPP6R3 | | | | | | | | T | | | C | | | | 0.72 | | | | 0.012 | | | | | 0.002 | | | | | | | 3.40E-14 | | | | | | 1.27E-04 | | | | 57 | | | |
| rs10796828 | 11 | | | 69490346 | | | | ORAOV1 | | | | | | | | T | | | G | | | | 0.37 | | | | -0.011 | | | | | 0.001 | | | | | | | 7.10E-15 | | | | | | 1.34E-04 | | | | 61 | | | |
| rs11215403 | 11 | | | 115058585 | | | | CADM1 | | | | | | | | G | | | A | | | | 0.76 | | | | 0.013 | | | | | 0.002 | | | | | | | 5.10E-16 | | | | | | 1.45E-04 | | | | 66 | | | |
| rs7123283 | 11 | | | 122809055 | | | | C11orf63 | | | | | | | | C | | | T | | | | 0.53 | | | | 0.008 | | | | | 0.001 | | | | | | | 3.70E-09 | | | | | | 7.67E-05 | | | | 35 | | | |
| rs10790809 | 11 | | | 126372550 | | | | KIRREL3 | | | | | | | | A | | | G | | | | 0.44 | | | | -0.010 | | | | | 0.001 | | | | | | | 7.20E-12 | | | | | | 1.04E-04 | | | | 47 | | | |
| rs55726687 | 12 | | | 991306 | | | | WNK1 | | | | | | | | G | | | A | | | | 0.79 | | | | -0.015 | | | | | 0.002 | | | | | | | 2.70E-17 | | | | | | 1.58E-04 | | | | 72 | | | |
| rs2187642 | 12 | | | 11855624 | | | | ETV6 | | | | | | | | A | | | C | | | | 0.38 | | | | -0.011 | | | | | 0.001 | | | | | | | 1.10E-14 | | | | | | 1.32E-04 | | | | 60 | | | |
| rs10841379 | 12 | | | 19992303 | | | | AEBP2 | | | | | | | | A | | | G | | | | 0.32 | | | | -0.008 | | | | | 0.002 | | | | | | | 2.90E-08 | | | | | | 6.79E-05 | | | | 31 | | | |
| rs10842356 | 12 | | | 24621348 | | | | BCAT1 | | | | | | | | A | | | T | | | | 0.48 | | | | 0.008 | | | | | 0.001 | | | | | | | 4.10E-09 | | | | | | 7.63E-05 | | | | 35 | | | |
| rs61937656 | 12 | | | 39483502 | | | | CPNE8 | | | | | | | | G | | | A | | | | 0.77 | | | | 0.012 | | | | | 0.002 | | | | | | | 2.70E-12 | | | | | | 1.08E-04 | | | | 49 | | | |
| rs7958241 | 12 | | | 49509262 | | | | LMBR1L | | | | | | | | A | | | G | | | | 0.66 | | | | -0.013 | | | | | 0.001 | | | | | | | 7.90E-19 | | | | | | 1.73E-04 | | | | 79 | | | |
| rs7132908 | 12 | | | 50263148 | | | | FAIM2 | | | | | | | | G | | | A | | | | 0.62 | | | | -0.031 | | | | | 0.001 | | | | | | | 1.70E-104 | | | | | | 1.04E-03 | | | | 471 | | | |
| rs836179 | 12 | | | 50503082 | | | | GPD1 | | | | | | | | A | | | G | | | | 0.63 | | | | 0.010 | | | | | 0.001 | | | | | | | 1.20E-11 | | | | | | 1.01E-04 | | | | 46 | | | |
| rs78607331 | 12 | | | 57648644 | | | | R3HDM2 | | | | | | | | C | | | T | | | | 0.96 | | | | -0.024 | | | | | 0.003 | | | | | | | 1.70E-12 | | | | | | 1.10E-04 | | | | 50 | | | |
| rs7306710 | 12 | | | 66376091 | | | | HMGA2 | | | | | | | | T | | | C | | | | 0.48 | | | | 0.010 | | | | | 0.001 | | | | | | | 1.60E-12 | | | | | | 1.10E-04 | | | | 50 | | | |
| rs10860295 | 12 | | | 98542699 | | | | RP11-181C3.1 | | | | | | | | T | | | C | | | | 0.55 | | | | -0.008 | | | | | 0.001 | | | | | | | 1.80E-09 | | | | | | 7.98E-05 | | | | 36 | | | |
| rs1552759 | 12 | | | 99640557 | | | | ANKS1B | | | | | | | | T | | | C | | | | 0.34 | | | | 0.009 | | | | | 0.001 | | | | | | | 6.30E-10 | | | | | | 8.43E-05 | | | | 38 | | | |
| rs12817542 | 12 | | | 103736499 | | | | C12orf42 | | | | | | | | C | | | T | | | | 0.94 | | | | -0.018 | | | | | 0.003 | | | | | | | 1.70E-09 | | | | | | 8.02E-05 | | | | 36 | | | |
| rs61936936 | 12 | | | 116391685 | | | | MED13L | | | | | | | | A | | | T | | | | 0.90 | | | | -0.014 | | | | | 0.002 | | | | | | | 7.80E-09 | | | | | | 7.35E-05 | | | | 33 | | | |
| rs7305424 | 12 | | | 118399491 | | | | KSR2 | | | | | | | | A | | | T | | | | 0.66 | | | | -0.010 | | | | | 0.001 | | | | | | | 2.30E-12 | | | | | | 1.09E-04 | | | | 49 | | | |
| rs12308065 | 12 | | | 120624085 | | | | GCN1L1 | | | | | | | | A | | | G | | | | 0.37 | | | | -0.008 | | | | | 0.001 | | | | | | | 1.10E-08 | | | | | | 7.19E-05 | | | | 33 | | | |
| rs28629903 | 12 | | | 122497001 | | | | BCL7A | | | | | | | | T | | | C | | | | 0.44 | | | | 0.009 | | | | | 0.001 | | | | | | | 1.50E-10 | | | | | | 9.06E-05 | | | | 41 | | | |
| rs7989098 | 13 | | | 27925496 | | | | GTF3A | | | | | | | | T | | | C | | | | 0.26 | | | | -0.012 | | | | | 0.002 | | | | | | | 1.00E-13 | | | | | | 1.22E-04 | | | | 55 | | | |
| rs9652090 | 13 | | | 27983367 | | | | GTF3A | | | | | | | | G | | | T | | | | 0.54 | | | | -0.008 | | | | | 0.001 | | | | | | | 4.10E-09 | | | | | | 7.63E-05 | | | | 35 | | | |
| rs1933437 | 13 | | | 28624294 | | | | FLT3 | | | | | | | | G | | | A | | | | 0.37 | | | | 0.014 | | | | | 0.001 | | | | | | | 1.60E-22 | | | | | | 2.10E-04 | | | | 95 | | | |
| rs9603697 | 13 | | | 40783323 | | | | AL133318.1 | | | | | | | | C | | | T | | | | 0.67 | | | | -0.013 | | | | | 0.001 | | | | | | | 5.00E-18 | | | | | | 1.65E-04 | | | | 75 | | | |
| rs9594686 | 13 | | | 42723197 | | | | DGKH | | | | | | | | C | | | T | | | | 0.82 | | | | 0.011 | | | | | 0.002 | | | | | | | 6.40E-09 | | | | | | 7.44E-05 | | | | 34 | | | |
| rs12429545 | 13 | | | 54102206 | | | | OLFM4 | | | | | | | | G | | | A | | | | 0.87 | | | | -0.021 | | | | | 0.002 | | | | | | | 1.20E-22 | | | | | | 2.12E-04 | | | | 96 | | | |
| rs9538141 | 13 | | | 59178258 | | | | PCDH17 | | | | | | | | G | | | A | | | | 0.49 | | | | -0.012 | | | | | 0.001 | | | | | | | 2.20E-16 | | | | | | 1.49E-04 | | | | 67 | | | |
| rs1333010 | 13 | | | 66205228 | | | | PCDH9 | | | | | | | | G | | | A | | | | 0.39 | | | | 0.012 | | | | | 0.001 | | | | | | | 5.20E-17 | | | | | | 1.55E-04 | | | | 70 | | | |
| rs1576655 | 13 | | | 79587841 | | | | RBM26 | | | | | | | | A | | | C | | | | 0.40 | | | | -0.012 | | | | | 0.001 | | | | | | | 4.30E-16 | | | | | | 1.46E-04 | | | | 66 | | | |
| rs61978655 | 14 | | | 30491807 | | | | PRKD1 | | | | | | | | G | | | A | | | | 0.96 | | | | -0.034 | | | | | 0.004 | | | | | | | 1.30E-20 | | | | | | 1.91E-04 | | | | 87 | | | |
| rs7161424 | 14 | | | 33309274 | | | | AKAP6 | | | | | | | | G | | | A | | | | 0.52 | | | | -0.010 | | | | | 0.001 | | | | | | | 4.50E-13 | | | | | | 1.16E-04 | | | | 52 | | | |
| rs1865719 | 14 | | | 79923667 | | | | NRXN3 | | | | | | | | A | | | G | | | | 0.37 | | | | -0.011 | | | | | 0.001 | | | | | | | 8.40E-14 | | | | | | 1.23E-04 | | | | 56 | | | |
| rs10133279 | 14 | | | 82702712 | | | | SEL1L | | | | | | | | C | | | T | | | | 0.56 | | | | -0.009 | | | | | 0.001 | | | | | | | 3.00E-10 | | | | | | 8.75E-05 | | | | 40 | | | |
| rs7145052 | 14 | | | 92461192 | | | | TRIP11 | | | | | | | | C | | | T | | | | 0.55 | | | | -0.008 | | | | | 0.001 | | | | | | | 3.70E-09 | | | | | | 7.67E-05 | | | | 35 | | | |
| rs7159126 | 14 | | | 93783176 | | | | BTBD7 | | | | | | | | T | | | C | | | | 0.72 | | | | 0.009 | | | | | 0.002 | | | | | | | 2.50E-08 | | | | | | 6.86E-05 | | | | 31 | | | |
| rs78420139 | 14 | | | 101201687 | | | | DLK1 | | | | | | | | G | | | A | | | | 0.94 | | | | 0.017 | | | | | 0.003 | | | | | | | 3.60E-08 | | | | | | 6.70E-05 | | | | 30 | | | |
| rs12436513 | 14 | | | 103269755 | | | | TRAF3 | | | | | | | | C | | | A | | | | 0.36 | | | | 0.009 | | | | | 0.001 | | | | | | | 4.20E-09 | | | | | | 7.62E-05 | | | | 35 | | | |
| rs824207 | 15 | | | 24007729 | | | | NDN | | | | | | | | A | | | G | | | | 0.47 | | | | -0.009 | | | | | 0.001 | | | | | | | 3.70E-11 | | | | | | 9.66E-05 | | | | 44 | | | |
| rs62048187 | 15 | | | 38117049 | | | | TMCO5A | | | | | | | | G | | | C | | | | 0.68 | | | | -0.008 | | | | | 0.002 | | | | | | | 4.00E-08 | | | | | | 6.66E-05 | | | | 30 | | | |
| rs10519136 | 15 | | | 47933163 | | | | SEMA6D | | | | | | | | C | | | T | | | | 0.60 | | | | 0.008 | | | | | 0.001 | | | | | | | 1.60E-08 | | | | | | 7.04E-05 | | | | 32 | | | |
| rs8030456 | 15 | | | 68076856 | | | | MAP2K5 | | | | | | | | C | | | T | | | | 0.77 | | | | 0.021 | | | | | 0.002 | | | | | | | 2.60E-37 | | | | | | 3.59E-04 | | | | 163 | | | |
| rs7162542 | 15 | | | 84514290 | | | | ADAMTSL3 | | | | | | | | C | | | G | | | | 0.44 | | | | 0.009 | | | | | 0.001 | | | | | | | 3.80E-11 | | | | | | 9.65E-05 | | | | 44 | | | |
| rs3817428 | 15 | | | 89415247 | | | | ACAN | | | | | | | | C | | | G | | | | 0.74 | | | | -0.011 | | | | | 0.002 | | | | | | | 1.30E-11 | | | | | | 1.01E-04 | | | | 46 | | | |
| rs1000471 | 15 | | | 89986583 | | | | RHCG | | | | | | | | C | | | T | | | | 0.78 | | | | -0.009 | | | | | 0.002 | | | | | | | 3.70E-08 | | | | | | 6.68E-05 | | | | 30 | | | |
| rs2970356 | 15 | | | 90623540 | | | | ZNF710 | | | | | | | | C | | | G | | | | 0.68 | | | | -0.010 | | | | | 0.002 | | | | | | | 4.50E-11 | | | | | | 9.57E-05 | | | | 43 | | | |
| rs72755233 | 15 | | | 100692953 | | | | ADAMTS17 | | | | | | | | G | | | A | | | | 0.89 | | | | -0.018 | | | | | 0.002 | | | | | | | 2.10E-15 | | | | | | 1.39E-04 | | | | 63 | | | |
| rs2238435 | 16 | | | 4014282 | | | | ADCY9 | | | | | | | | C | | | G | | | | 0.39 | | | | -0.016 | | | | | 0.001 | | | | | | | 2.90E-30 | | | | | | 2.88E-04 | | | | 131 | | | |
| rs55880046 | 16 | | | 19941557 | | | | GPRC5B | | | | | | | | T | | | G | | | | 0.86 | | | | 0.030 | | | | | 0.002 | | | | | | | 3.70E-50 | | | | | | 4.89E-04 | | | | 222 | | | |
| rs4432271 | 16 | | | 20245283 | | | | GP2 | | | | | | | | C | | | T | | | | 0.13 | | | | -0.017 | | | | | 0.002 | | | | | | | 1.00E-16 | | | | | | 1.52E-04 | | | | 69 | | | |
| rs9922288 | 16 | | | 24550930 | | | | RBBP6 | | | | | | | | A | | | G | | | | 0.24 | | | | 0.010 | | | | | 0.002 | | | | | | | 3.40E-10 | | | | | | 8.69E-05 | | | | 39 | | | |
| rs62037365 | 16 | | | 28868962 | | | | SH2B1 | | | | | | | | C | | | G | | | | 0.60 | | | | -0.014 | | | | | 0.001 | | | | | | | 9.90E-22 | | | | | | 2.02E-04 | | | | 92 | | | |
| rs4889630 | 16 | | | 30877544 | | | | BCL7C | | | | | | | | T | | | C | | | | 0.20 | | | | 0.012 | | | | | 0.002 | | | | | | | 1.90E-12 | | | | | | 1.09E-04 | | | | 50 | | | |
| rs4783789 | 16 | | | 51446707 | | | | SALL1 | | | | | | | | T | | | C | | | | 0.78 | | | | 0.010 | | | | | 0.002 | | | | | | | 1.40E-08 | | | | | | 7.10E-05 | | | | 32 | | | |
| rs1421085 | 16 | | | 53800954 | | | | FTO | | | | | | | | T | | | C | | | | 0.60 | | | | -0.047 | | | | | 0.001 | | | | | | | 1.10E-241 | | | | | | 2.43E-03 | | | | 1102 | | | |
| rs594585 | 16 | | | 65939803 | | | | CDH5 | | | | | | | | T | | | G | | | | 0.41 | | | | 0.008 | | | | | 0.001 | | | | | | | 3.00E-08 | | | | | | 6.78E-05 | | | | 31 | | | |
| rs117903946 | 16 | | | 67449639 | | | | ZDHHC1 | | | | | | | | G | | | A | | | | 0.97 | | | | -0.032 | | | | | 0.004 | | | | | | | 3.20E-16 | | | | | | 1.47E-04 | | | | 67 | | | |
| rs7672 | 16 | | | 68294800 | | | | PLA2G15 | | | | | | | | C | | | G | | | | 0.29 | | | | 0.009 | | | | | 0.002 | | | | | | | 7.90E-09 | | | | | | 7.35E-05 | | | | 33 | | | |
| rs4985555 | 16 | | | 70690166 | | | | IL34 | | | | | | | | A | | | G | | | | 0.50 | | | | 0.009 | | | | | 0.001 | | | | | | | 4.60E-10 | | | | | | 8.57E-05 | | | | 39 | | | |
| rs11642090 | 16 | | | 81730582 | | | | CMIP | | | | | | | | T | | | C | | | | 0.63 | | | | -0.012 | | | | | 0.001 | | | | | | | 9.20E-16 | | | | | | 1.43E-04 | | | | 65 | | | |
| rs72819571 | 17 | | | 2170501 | | | | SMG6 | | | | | | | | G | | | T | | | | 0.65 | | | | 0.012 | | | | | 0.001 | | | | | | | 6.70E-16 | | | | | | 1.44E-04 | | | | 65 | | | |
| rs67603370 | 17 | | | 7524504 | | | | SHBG | | | | | | | | G | | | A | | | | 0.92 | | | | -0.016 | | | | | 0.003 | | | | | | | 3.40E-09 | | | | | | 7.71E-05 | | | | 35 | | | |
| rs3815156 | 17 | | | 29685150 | | | | NF1 | | | | | | | | A | | | G | | | | 0.82 | | | | -0.010 | | | | | 0.002 | | | | | | | 2.20E-08 | | | | | | 6.91E-05 | | | | 31 | | | |
| rs12601380 | 17 | | | 34904985 | | | | GGNBP2 | | | | | | | | A | | | C | | | | 0.59 | | | | 0.008 | | | | | 0.001 | | | | | | | 4.30E-08 | | | | | | 6.62E-05 | | | | 30 | | | |
| rs9299 | 17 | | | 46669430 | | | | HOXB3 | | | | | | | | C | | | T | | | | 0.34 | | | | -0.010 | | | | | 0.001 | | | | | | | 7.30E-11 | | | | | | 9.36E-05 | | | | 42 | | | |
| rs17637472 | 17 | | | 47461433 | | | | RP11-81K2.1 | | | | | | | | G | | | A | | | | 0.60 | | | | -0.011 | | | | | 0.001 | | | | | | | 4.10E-14 | | | | | | 1.26E-04 | | | | 57 | | | |
| rs7217460 | 17 | | | 65393344 | | | | PITPNC1 | | | | | | | | G | | | A | | | | 0.77 | | | | 0.009 | | | | | 0.002 | | | | | | | 2.80E-08 | | | | | | 6.81E-05 | | | | 31 | | | |
| rs12941038 | 17 | | | 66509143 | | | | PRKAR1A | | | | | | | | C | | | T | | | | 0.77 | | | | -0.010 | | | | | 0.002 | | | | | | | 1.00E-08 | | | | | | 7.24E-05 | | | | 33 | | | |
| rs2246623 | 17 | | | 74084449 | | | | EXOC7 | | | | | | | | C | | | T | | | | 0.55 | | | | 0.009 | | | | | 0.001 | | | | | | | 2.20E-10 | | | | | | 8.88E-05 | | | | 40 | | | |
| rs11150745 | 17 | | | 78757626 | | | | RPTOR | | | | | | | | A | | | G | | | | 0.68 | | | | 0.012 | | | | | 0.002 | | | | | | | 7.00E-16 | | | | | | 1.44E-04 | | | | 65 | | | |
| rs7503580 | 17 | | | 79087036 | | | | BAIAP2 | | | | | | | | C | | | T | | | | 0.84 | | | | -0.011 | | | | | 0.002 | | | | | | | 8.70E-09 | | | | | | 7.31E-05 | | | | 33 | | | |
| rs1013737 | 18 | | | 937050 | | | | ADCYAP1 | | | | | | | | G | | | C | | | | 0.51 | | | | -0.011 | | | | | 0.001 | | | | | | | 3.30E-14 | | | | | | 1.27E-04 | | | | 58 | | | |
| rs1808579 | 18 | | | 21104888 | | | | NPC1 | | | | | | | | C | | | T | | | | 0.52 | | | | 0.009 | | | | | 0.001 | | | | | | | 7.70E-11 | | | | | | 9.34E-05 | | | | 42 | | | |
| rs7237444 | 18 | | | 39468982 | | | | PIK3C3 | | | | | | | | G | | | A | | | | 0.33 | | | | 0.010 | | | | | 0.002 | | | | | | | 3.40E-11 | | | | | | 9.69E-05 | | | | 44 | | | |
| rs7239114 | 18 | | | 45921214 | | | | ZBTB7C | | | | | | | | G | | | A | | | | 0.46 | | | | -0.013 | | | | | 0.001 | | | | | | | 1.80E-21 | | | | | | 2.00E-04 | | | | 91 | | | |
| rs68015088 | 18 | | | 51484010 | | | | MBD2 | | | | | | | | G | | | A | | | | 0.65 | | | | 0.008 | | | | | 0.001 | | | | | | | 4.30E-08 | | | | | | 6.62E-05 | | | | 30 | | | |
| rs12606230 | 18 | | | 52492252 | | | | RAB27B | | | | | | | | T | | | C | | | | 0.77 | | | | -0.013 | | | | | 0.002 | | | | | | | 1.50E-14 | | | | | | 1.31E-04 | | | | 59 | | | |
| rs663129 | 18 | | | 57838401 | | | | MC4R | | | | | | | | G | | | A | | | | 0.77 | | | | -0.033 | | | | | 0.002 | | | | | | | 8.80E-90 | | | | | | 8.90E-04 | | | | 404 | | | |
| rs113728099 | 18 | | | 58157767 | | | | MC4R | | | | | | | | G | | | A | | | | 0.98 | | | | 0.044 | | | | | 0.005 | | | | | | | 4.30E-22 | | | | | | 2.06E-04 | | | | 93 | | | |
| rs8096658 | 18 | | | 77156537 | | | | NFATC1 | | | | | | | | C | | | G | | | | 0.51 | | | | 0.009 | | | | | 0.001 | | | | | | | 2.30E-10 | | | | | | 8.87E-05 | | | | 40 | | | |
| rs62621197 | 19 | | | 8670147 | | | | ADAMTS10 | | | | | | | | C | | | T | | | | 0.96 | | | | -0.023 | | | | | 0.004 | | | | | | | 1.00E-09 | | | | | | 8.22E-05 | | | | 37 | | | |
| rs4545941 | 19 | | | 16534207 | | | | EPS15L1 | | | | | | | | T | | | C | | | | 0.83 | | | | -0.011 | | | | | 0.002 | | | | | | | 1.10E-08 | | | | | | 7.22E-05 | | | | 33 | | | |
| rs116399833 | 19 | | | 18502374 | | | | LRRC25 | | | | | | | | C | | | A | | | | 0.77 | | | | -0.012 | | | | | 0.002 | | | | | | | 4.60E-13 | | | | | | 1.16E-04 | | | | 52 | | | |
| rs4808961 | 19 | | | 19577215 | | | | GATAD2A | | | | | | | | C | | | G | | | | 0.63 | | | | 0.009 | | | | | 0.001 | | | | | | | 1.20E-09 | | | | | | 8.16E-05 | | | | 37 | | | |
| rs3810304 | 19 | | | 30861683 | | | | ZNF536 | | | | | | | | A | | | G | | | | 0.23 | | | | 0.010 | | | | | 0.002 | | | | | | | 8.50E-10 | | | | | | 8.30E-05 | | | | 38 | | | |
| rs1800437 | 19 | | | 46181392 | | | | GIPR | | | | | | | | G | | | C | | | | 0.81 | | | | 0.012 | | | | | 0.002 | | | | | | | 2.90E-11 | | | | | | 9.76E-05 | | | | 44 | | | |
| rs3810291 | 19 | | | 47569003 | | | | ZC3H4 | | | | | | | | G | | | A | | | | 0.32 | | | | -0.015 | | | | | 0.001 | | | | | | | 5.60E-23 | | | | | | 2.15E-04 | | | | 97 | | | |
| rs601338 | 19 | | | 49206674 | | | | FUT2 | | | | | | | | G | | | A | | | | 0.49 | | | | 0.009 | | | | | 0.001 | | | | | | | 2.10E-11 | | | | | | 9.90E-05 | | | | 45 | | | |
| rs16996644 | 20 | | | 15813475 | | | | MACROD2 | | | | | | | | C | | | G | | | | 0.87 | | | | -0.019 | | | | | 0.002 | | | | | | | 4.80E-20 | | | | | | 1.85E-04 | | | | 84 | | | |
| rs947088 | 20 | | | 17171373 | | | | PCSK2 | | | | | | | | G | | | T | | | | 0.28 | | | | -0.011 | | | | | 0.002 | | | | | | | 9.50E-12 | | | | | | 1.02E-04 | | | | 46 | | | |
| rs8117463 | 20 | | | 17231063 | | | | PCSK2 | | | | | | | | G | | | A | | | | 0.68 | | | | 0.009 | | | | | 0.001 | | | | | | | 3.30E-09 | | | | | | 7.72E-05 | | | | 35 | | | |
| rs73085586 | 20 | | | 22430241 | | | | FOXA2 | | | | | | | | G | | | A | | | | 0.80 | | | | -0.010 | | | | | 0.002 | | | | | | | 3.00E-08 | | | | | | 6.78E-05 | | | | 31 | | | |
| rs2281148 | 20 | | | 36433288 | | | | CTNNBL1 | | | | | | | | T | | | C | | | | 0.75 | | | | -0.010 | | | | | 0.002 | | | | | | | 1.50E-09 | | | | | | 8.06E-05 | | | | 37 | | | |
| rs2207894 | 20 | | | 54387343 | | | | CBLN4 | | | | | | | | C | | | T | | | | 0.81 | | | | 0.014 | | | | | 0.002 | | | | | | | 8.00E-16 | | | | | | 1.43E-04 | | | | 65 | | | |
| rs117455294 | 20 | | | 57427951 | | | | GNAS | | | | | | | | C | | | A | | | | 0.95 | | | | 0.019 | | | | | 0.003 | | | | | | | 1.20E-09 | | | | | | 8.17E-05 | | | | 37 | | | |
| rs8130408 | 21 | | | 39237138 | | | | KCNJ6 | | | | | | | | A | | | C | | | | 0.26 | | | | -0.009 | | | | | 0.002 | | | | | | | 2.30E-08 | | | | | | 6.89E-05 | | | | 31 | | | |
| rs13047416 | 21 | | | 40309436 | | | | ETS2 | | | | | | | | C | | | G | | | | 0.62 | | | | 0.012 | | | | | 0.001 | | | | | | | 1.40E-17 | | | | | | 1.61E-04 | | | | 73 | | | |
| rs78907487 | 22 | | | 22151939 | | | | MAPK1 | | | | | | | | A | | | C | | | | 0.85 | | | | -0.012 | | | | | 0.002 | | | | | | | 4.10E-10 | | | | | | 8.62E-05 | | | | 39 | | | |
| rs9610387 | 22 | | | 36476762 | | | | RBFOX2 | | | | | | | | G | | | A | | | | 0.91 | | | | 0.014 | | | | | 0.003 | | | | | | | 3.90E-08 | | | | | | 6.66E-05 | | | | 30 | | | |
| rs6001872 | 22 | | | 40703245 | | | | TNRC6B | | | | | | | | A | | | G | | | | 0.65 | | | | 0.013 | | | | | 0.001 | | | | | | | 1.50E-17 | | | | | | 1.60E-04 | | | | 73 | | | |
| rs9611560 | 22 | | | 41750622 | | | | ZC3H7B | | | | | | | | T | | | C | | | | 0.26 | | | | 0.009 | | | | | 0.002 | | | | | | | 6.50E-09 | | | | | | 7.43E-05 | | | | 34 | | | |
| Genome-wide significant SNPs for early life body size in UK Biobank (men only) | | | | | | | | | | | | | | | | | | | | | | | | | | | | | | | | | | | | | | | | | | | | | | | | | | | | |
| SNP | | | Chr | | | Position | | | | Closest gene | | | | | EA | | | | OA | | | | EAF | | | Beta | | | | | SE | | | | | | pvalue | | | | | | R^2^ | | | | | | | F statistic | | |
| rs12140153 | | | 1 | | | 62579891 | | | | INADL | | | | | G | | | | T | | | | 0.905 | | | 0.023 | | | | | 0.004 | | | | | | 6.4E-11 | | | | | | 2.07E-04 | | | | | | | 43 | | |
| rs2012697 | | | 1 | | | 72819612 | | | | NEGR1 | | | | | T | | | | C | | | | 0.401 | | | -0.019 | | | | | 0.002 | | | | | | 4.5E-20 | | | | | | 4.07E-04 | | | | | | | 84 | | |
| rs4650277 | | | 1 | | | 74993721 | | | | TNNI3K | | | | | A | | | | G | | | | 0.435 | | | 0.023 | | | | | 0.002 | | | | | | 1.4E-27 | | | | | | 5.73E-04 | | | | | | | 118 | | |
| rs7550711 | | | 1 | | | 110082886 | | | | GPR61 | | | | | C | | | | T | | | | 0.974 | | | -0.051 | | | | | 0.006 | | | | | | 5.8E-15 | | | | | | 2.95E-04 | | | | | | | 61 | | |
| rs539515 | | | 1 | | | 177889025 | | | | SEC16B | | | | | A | | | | C | | | | 0.795 | | | -0.039 | | | | | 0.003 | | | | | | 1.3E-52 | | | | | | 1.13E-03 | | | | | | | 233 | | |
| rs78444298 | | | 1 | | | 184672098 | | | | EDEM3 | | | | | G | | | | A | | | | 0.981 | | | 0.047 | | | | | 0.008 | | | | | | 3.5E-10 | | | | | | 1.90E-04 | | | | | | | 39 | | |
| rs1772143 | | | 1 | | | 205799987 | | | | PM20D1 | | | | | T | | | | A | | | | 0.587 | | | 0.015 | | | | | 0.002 | | | | | | 2.2E-12 | | | | | | 2.38E-04 | | | | | | | 49 | | |
| rs77165542 | | | 2 | | | 430975 | | | | FAM150B | | | | | C | | | | T | | | | 0.965 | | | 0.073 | | | | | 0.006 | | | | | | 1.7E-38 | | | | | | 8.14E-04 | | | | | | | 168 | | |
| rs6749422 | | | 2 | | | 25150011 | | | | ADCY3 | | | | | C | | | | G | | | | 0.514 | | | -0.031 | | | | | 0.002 | | | | | | 1.4E-52 | | | | | | 1.13E-03 | | | | | | | 233 | | |
| rs2862874 | | | 2 | | | 58990485 | | | | FANCL | | | | | G | | | | T | | | | 0.388 | | | -0.012 | | | | | 0.002 | | | | | | 1E-08 | | | | | | 1.59E-04 | | | | | | | 33 | | |
| rs10496885 | | | 2 | | | 142285767 | | | | LRP1B | | | | | G | | | | A | | | | 0.814 | | | 0.017 | | | | | 0.003 | | | | | | 3.3E-10 | | | | | | 1.91E-04 | | | | | | | 39 | | |
| rs115319174 | | | 2 | | | 207066474 | | | | GPR1 | | | | | G | | | | C | | | | 0.942 | | | -0.043 | | | | | 0.004 | | | | | | 1.4E-22 | | | | | | 4.63E-04 | | | | | | | 96 | | |
| rs9880272 | | | 3 | | | 85687785 | | | | CADM2 | | | | | T | | | | G | | | | 0.759 | | | -0.014 | | | | | 0.002 | | | | | | 7.8E-09 | | | | | | 1.61E-04 | | | | | | | 33 | | |
| rs34722008 | | | 4 | | | 38659594 | | | | AC021860.1 | | | | | G | | | | A | | | | 0.646 | | | 0.012 | | | | | 0.002 | | | | | | 4E-08 | | | | | | 1.46E-04 | | | | | | | 30 | | |
| rs10938398 | | | 4 | | | 45186139 | | | | GNPDA2 | | | | | G | | | | A | | | | 0.565 | | | -0.021 | | | | | 0.002 | | | | | | 1.7E-23 | | | | | | 4.83E-04 | | | | | | | 100 | | |
| rs13107325 | | | 4 | | | 103188709 | | | | SLC39A8 | | | | | C | | | | T | | | | 0.924 | | | -0.023 | | | | | 0.004 | | | | | | 5.2E-09 | | | | | | 1.65E-04 | | | | | | | 34 | | |
| rs3212519 | | | 5 | | | 52351182 | | | | ITGA2 | | | | | A | | | | G | | | | 0.930 | | | 0.022 | | | | | 0.004 | | | | | | 2.9E-08 | | | | | | 1.49E-04 | | | | | | | 31 | | |
| rs75577466 | | | 5 | | | 60605507 | | | | ZSWIM6 | | | | | G | | | | C | | | | 0.823 | | | 0.017 | | | | | 0.003 | | | | | | 3.6E-10 | | | | | | 1.90E-04 | | | | | | | 39 | | |
| rs25842 | | | 5 | | | 66163135 | | | | MAST4 | | | | | C | | | | T | | | | 0.723 | | | 0.013 | | | | | 0.002 | | | | | | 5.4E-09 | | | | | | 1.65E-04 | | | | | | | 34 | | |
| rs55654862 | | | 5 | | | 77428253 | | | | AP3B1 | | | | | A | | | | G | | | | 0.763 | | | 0.014 | | | | | 0.002 | | | | | | 1.6E-08 | | | | | | 1.54E-04 | | | | | | | 32 | | |
| rs4958361 | | | 5 | | | 153545587 | | | | MFAP3 | | | | | C | | | | G | | | | 0.472 | | | 0.012 | | | | | 0.002 | | | | | | 3.8E-09 | | | | | | 1.68E-04 | | | | | | | 35 | | |
| rs2240071 | | | 6 | | | 30070932 | | | | TRIM31 | | | | | C | | | | G | | | | 0.747 | | | -0.013 | | | | | 0.002 | | | | | | 3E-08 | | | | | | 1.49E-04 | | | | | | | 31 | | |
| rs62405422 | | | 6 | | | 50796905 | | | | TFAP2B | | | | | T | | | | C | | | | 0.820 | | | -0.022 | | | | | 0.003 | | | | | | 2.5E-16 | | | | | | 3.25E-04 | | | | | | | 67 | | |
| rs1775255 | | | 6 | | | 51243035 | | | | PKHD1 | | | | | G | | | | T | | | | 0.524 | | | -0.013 | | | | | 0.002 | | | | | | 1.1E-10 | | | | | | 2.02E-04 | | | | | | | 42 | | |
| rs115597956 | | | 6 | | | 54042871 | | | | MLIP | | | | | G | | | | A | | | | 0.945 | | | -0.030 | | | | | 0.005 | | | | | | 3.5E-11 | | | | | | 2.12E-04 | | | | | | | 44 | | |
| rs12110721 | | | 6 | | | 55190480 | | | | GFRAL | | | | | G | | | | A | | | | 0.826 | | | -0.018 | | | | | 0.003 | | | | | | 5.3E-11 | | | | | | 2.08E-04 | | | | | | | 43 | | |
| rs2693560 | | | 6 | | | 117523671 | | | | VGLL2 | | | | | A | | | | G | | | | 0.371 | | | 0.012 | | | | | 0.002 | | | | | | 4.9E-08 | | | | | | 1.44E-04 | | | | | | | 30 | | |
| rs1452991 | | | 6 | | | 141473363 | | | | NMBR | | | | | G | | | | A | | | | 0.627 | | | -0.012 | | | | | 0.002 | | | | | | 1.5E-08 | | | | | | 1.55E-04 | | | | | | | 32 | | |
| rs16120 | | | 7 | | | 24334724 | | | | NPY | | | | | A | | | | G | | | | 0.480 | | | 0.012 | | | | | 0.002 | | | | | | 1.4E-08 | | | | | | 1.56E-04 | | | | | | | 32 | | |
| rs7796922 | | | 7 | | | 33171110 | | | | BBS9 | | | | | A | | | | G | | | | 0.906 | | | 0.020 | | | | | 0.004 | | | | | | 2.9E-08 | | | | | | 1.49E-04 | | | | | | | 31 | | |
| rs2979139 | | | 8 | | | 8268313 | | | | SGK223 | | | | | A | | | | G | | | | 0.507 | | | -0.012 | | | | | 0.002 | | | | | | 4.2E-09 | | | | | | 1.67E-04 | | | | | | | 35 | | |
| rs12674871 | | | 8 | | | 9979144 | | | | MSRA | | | | | C | | | | T | | | | 0.799 | | | -0.014 | | | | | 0.003 | | | | | | 2.1E-08 | | | | | | 1.52E-04 | | | | | | | 31 | | |
| rs10504620 | | | 8 | | | 77288303 | | | | ZFHX4 | | | | | T | | | | C | | | | 0.301 | | | -0.015 | | | | | 0.002 | | | | | | 1.3E-11 | | | | | | 2.22E-04 | | | | | | | 46 | | |
| rs10968101 | | | 9 | | | 27772542 | | | | LINGO2 | | | | | G | | | | A | | | | 0.522 | | | -0.014 | | | | | 0.002 | | | | | | 2.2E-11 | | | | | | 2.17E-04 | | | | | | | 45 | | |
| rs11790060 | | | 9 | | | 96202932 | | | | FAM120AOS | | | | | T | | | | C | | | | 0.669 | | | -0.013 | | | | | 0.002 | | | | | | 4.2E-09 | | | | | | 1.67E-04 | | | | | | | 35 | | |
| rs41310284 | | | 10 | | | 102447647 | | | | PAX2 | | | | | C | | | | A | | | | 0.899 | | | 0.024 | | | | | 0.003 | | | | | | 1.9E-12 | | | | | | 2.40E-04 | | | | | | | 50 | | |
| rs11030102 | | | 11 | | | 27681596 | | | | BDNF | | | | | C | | | | G | | | | 0.740 | | | -0.018 | | | | | 0.002 | | | | | | 4.1E-14 | | | | | | 2.76E-04 | | | | | | | 57 | | |
| rs3817334 | | | 11 | | | 47650993 | | | | MTCH2 | | | | | C | | | | T | | | | 0.593 | | | -0.014 | | | | | 0.002 | | | | | | 6E-11 | | | | | | 2.07E-04 | | | | | | | 43 | | |
| rs10896348 | | | 11 | | | 68357368 | | | | PPP6R3 | | | | | T | | | | C | | | | 0.723 | | | 0.013 | | | | | 0.002 | | | | | | 6E-09 | | | | | | 1.64E-04 | | | | | | | 34 | | |
| rs11218734 | | | 11 | | | 122523246 | | | | UBASH3B | | | | | A | | | | G | | | | 0.743 | | | -0.013 | | | | | 0.002 | | | | | | 3.6E-08 | | | | | | 1.47E-04 | | | | | | | 30 | | |
| rs7978659 | | | 12 | | | 49507127 | | | | LMBR1L | | | | | G | | | | T | | | | 0.658 | | | -0.013 | | | | | 0.002 | | | | | | 7E-10 | | | | | | 1.84E-04 | | | | | | | 38 | | |
| rs7132908 | | | 12 | | | 50263148 | | | | FAIM2 | | | | | G | | | | A | | | | 0.615 | | | -0.032 | | | | | 0.002 | | | | | | 3.6E-51 | | | | | | 1.09E-03 | | | | | | | 226 | | |
| rs7306710 | | | 12 | | | 66376091 | | | | HMGA2 | | | | | T | | | | C | | | | 0.479 | | | 0.012 | | | | | 0.002 | | | | | | 2E-09 | | | | | | 1.74E-04 | | | | | | | 36 | | |
| rs7316962 | | | 13 | | | 28613993 | | | | FLT3 | | | | | A | | | | G | | | | 0.453 | | | 0.013 | | | | | 0.002 | | | | | | 4E-10 | | | | | | 1.89E-04 | | | | | | | 39 | | |
| rs9568868 | | | 13 | | | 54107583 | | | | OLFM4 | | | | | G | | | | T | | | | 0.870 | | | -0.025 | | | | | 0.003 | | | | | | 7.6E-16 | | | | | | 3.14E-04 | | | | | | | 65 | | |
| rs1576655 | | | 13 | | | 79587841 | | | | RBM26 | | | | | A | | | | C | | | | 0.404 | | | -0.012 | | | | | 0.002 | | | | | | 3.7E-08 | | | | | | 1.47E-04 | | | | | | | 30 | | |
| rs61978655 | | | 14 | | | 30491807 | | | | PRKD1 | | | | | G | | | | A | | | | 0.961 | | | -0.030 | | | | | 0.005 | | | | | | 1.2E-08 | | | | | | 1.57E-04 | | | | | | | 32 | | |
| rs2143975 | | | 14 | | | 33297398 | | | | AKAP6 | | | | | C | | | | G | | | | 0.466 | | | 0.012 | | | | | 0.002 | | | | | | 3.2E-09 | | | | | | 1.70E-04 | | | | | | | 35 | | |
| rs7159126 | | | 14 | | | 93783176 | | | | BTBD7 | | | | | T | | | | C | | | | 0.718 | | | 0.013 | | | | | 0.002 | | | | | | 3.6E-08 | | | | | | 1.47E-04 | | | | | | | 30 | | |
| rs3784710 | | | 15 | | | 68072458 | | | | MAP2K5 | | | | | T | | | | C | | | | 0.774 | | | 0.019 | | | | | 0.002 | | | | | | 5.1E-15 | | | | | | 2.96E-04 | | | | | | | 61 | | |
| rs7190603 | | | 16 | | | 19928662 | | | | GPRC5B | | | | | T | | | | C | | | | 0.858 | | | 0.030 | | | | | 0.003 | | | | | | 1.2E-23 | | | | | | 4.86E-04 | | | | | | | 101 | | |
| rs56094641 | | | 16 | | | 53806453 | | | | FTO | | | | | A | | | | G | | | | 0.593 | | | -0.046 | | | | | 0.002 | | | | | | 1.2E-108 | | | | | | 2.37E-03 | | | | | | | 490 | | |
| rs17637472 | | | 17 | | | 47461433 | | | | RP11-81K2.1 | | | | | G | | | | A | | | | 0.602 | | | -0.012 | | | | | 0.002 | | | | | | 5.2E-09 | | | | | | 1.65E-04 | | | | | | | 34 | | |
| rs2250081 | | | 17 | | | 74090700 | | | | EXOC7 | | | | | G | | | | A | | | | 0.552 | | | 0.011 | | | | | 0.002 | | | | | | 2.7E-08 | | | | | | 1.50E-04 | | | | | | | 31 | | |
| rs7239114 | | | 18 | | | 45921214 | | | | ZBTB7C | | | | | G | | | | A | | | | 0.459 | | | -0.013 | | | | | 0.002 | | | | | | 8.1E-10 | | | | | | 1.83E-04 | | | | | | | 38 | | |
| rs3764516 | | | 18 | | | 52494374 | | | | RAB27B | | | | | A | | | | C | | | | 0.235 | | | 0.013 | | | | | 0.002 | | | | | | 3.3E-08 | | | | | | 1.48E-04 | | | | | | | 31 | | |
| rs663129 | | | 18 | | | 57838401 | | | | MC4R | | | | | G | | | | A | | | | 0.766 | | | -0.031 | | | | | 0.002 | | | | | | 2.5E-36 | | | | | | 7.66E-04 | | | | | | | 158 | | |
| rs1532127 | | | 19 | | | 47571938 | | | | ZC3H4 | | | | | G | | | | A | | | | 0.318 | | | -0.016 | | | | | 0.002 | | | | | | 1.2E-12 | | | | | | 2.45E-04 | | | | | | | 51 | | |
| rs1321434 | | | 20 | | | 6624443 | | | | BMP2 | | | | | A | | | | G | | | | 0.540 | | | -0.013 | | | | | 0.002 | | | | | | 5.4E-10 | | | | | | 1.86E-04 | | | | | | | 39 | | |
| rs73898513 | | | 20 | | | 15806210 | | | | MACROD2 | | | | | C | | | | T | | | | 0.878 | | | -0.019 | | | | | 0.003 | | | | | | 3.7E-09 | | | | | | 1.68E-04 | | | | | | | 35 | | |
| Genome-wide significant SNPs for early life body size in UK Biobank (women only) | | | | | | | | | | | | | | | | | | | | | | | | | | | | | | | | | | | | | | | | | | | | | | | | | | | | | |
| SNP | | | Chr | | | | Position | | | | | Closest gene | | | | | EA | | | | OA | | | EAF | | | Beta | | | | | | SE | | | | | pvalue | | | | | | R^2^ | | | | | | F statistic | | | |
| rs212540 | | | 1 | | | | 21593117 | | | | | ECE1 | | | | | C | | | | T | | | 0.393 | | | 0.013 | | | | | | 0.002 | | | | | 2.3E-10 | | | | | | 1.63E-04 | | | | | | 40 | | | |
| rs582220 | | | 1 | | | | 54724762 | | | | | SSBP3 | | | | | A | | | | G | | | 0.433 | | | -0.011 | | | | | | 0.002 | | | | | 4.5E-09 | | | | | | 1.39E-04 | | | | | | 34 | | | |
| rs12140153 | | | 1 | | | | 62579891 | | | | | INADL | | | | | G | | | | T | | | 0.906 | | | 0.020 | | | | | | 0.003 | | | | | 5.5E-09 | | | | | | 1.38E-04 | | | | | | 34 | | | |
| rs2767486 | | | 1 | | | | 65991203 | | | | | LEPR | | | | | A | | | | G | | | 0.798 | | | -0.020 | | | | | | 0.002 | | | | | 3.5E-17 | | | | | | 2.88E-04 | | | | | | 71 | | | |
| rs7522014 | | | 1 | | | | 66551759 | | | | | PDE4B | | | | | A | | | | G | | | 0.673 | | | 0.012 | | | | | | 0.002 | | | | | 4.2E-08 | | | | | | 1.22E-04 | | | | | | 30 | | | |
| rs11209943 | | | 1 | | | | 72750500 | | | | | NEGR1 | | | | | A | | | | G | | | 0.397 | | | -0.017 | | | | | | 0.002 | | | | | 8.1E-19 | | | | | | 3.18E-04 | | | | | | 78 | | | |
| rs12042908 | | | 1 | | | | 74997762 | | | | | TNNI3K | | | | | A | | | | G | | | 0.438 | | | 0.032 | | | | | | 0.002 | | | | | 2.1E-59 | | | | | | 1.07E-03 | | | | | | 264 | | | |
| rs41279738 | | | 1 | | | | 110082551 | | | | | GPR61 | | | | | T | | | | G | | | 0.974 | | | -0.046 | | | | | | 0.006 | | | | | 2.2E-14 | | | | | | 2.36E-04 | | | | | | 58 | | | |
| rs543874 | | | 1 | | | | 177889480 | | | | | SEC16B | | | | | A | | | | G | | | 0.795 | | | -0.055 | | | | | | 0.002 | | | | | 1.6E-115 | | | | | | 2.11E-03 | | | | | | 522 | | | |
| rs10798139 | | | 1 | | | | 187714179 | | | | | PLA2G4A | | | | | C | | | | T | | | 0.783 | | | -0.014 | | | | | | 0.002 | | | | | 5.9E-09 | | | | | | 1.37E-04 | | | | | | 34 | | | |
| rs815339 | | | 1 | | | | 190116575 | | | | | BRINP3 | | | | | T | | | | A | | | 0.495 | | | -0.011 | | | | | | 0.002 | | | | | 3.8E-08 | | | | | | 1.23E-04 | | | | | | 30 | | | |
| rs4971239 | | | 1 | | | | 203491150 | | | | | OPTC | | | | | G | | | | A | | | 0.831 | | | -0.015 | | | | | | 0.003 | | | | | 9.9E-09 | | | | | | 1.33E-04 | | | | | | 33 | | | |
| rs62106258 | | | 2 | | | | 417167 | | | | | FAM150B | | | | | T | | | | C | | | 0.951 | | | 0.087 | | | | | | 0.004 | | | | | 3.4E-84 | | | | | | 1.53E-03 | | | | | | 378 | | | |
| rs12992672 | | | 2 | | | | 632592 | | | | | TMEM18 | | | | | G | | | | A | | | 0.172 | | | -0.044 | | | | | | 0.003 | | | | | 1.1E-66 | | | | | | 1.21E-03 | | | | | | 298 | | | |
| rs6738433 | | | 2 | | | | 25159501 | | | | | DNAJC27 | | | | | G | | | | C | | | 0.521 | | | -0.040 | | | | | | 0.002 | | | | | 7.6E-97 | | | | | | 1.77E-03 | | | | | | 436 | | | |
| rs146910503 | | | 2 | | | | 25446473 | | | | | DNMT3A | | | | | G | | | | A | | | 0.979 | | | 0.047 | | | | | | 0.007 | | | | | 1.3E-11 | | | | | | 1.86E-04 | | | | | | 46 | | | |
| rs1446725 | | | 2 | | | | 77222421 | | | | | LRRTM4 | | | | | T | | | | G | | | 0.587 | | | 0.014 | | | | | | 0.002 | | | | | 4.8E-12 | | | | | | 1.94E-04 | | | | | | 48 | | | |
| rs1483153 | | | 2 | | | | 142358477 | | | | | LRP1B | | | | | C | | | | T | | | 0.219 | | | -0.015 | | | | | | 0.002 | | | | | 5.3E-10 | | | | | | 1.56E-04 | | | | | | 39 | | | |
| rs55959207 | | | 2 | | | | 161016981 | | | | | ITGB6 | | | | | A | | | | C | | | 0.574 | | | 0.011 | | | | | | 0.002 | | | | | 2E-08 | | | | | | 1.28E-04 | | | | | | 31 | | | |
| rs17464221 | | | 2 | | | | 188278203 | | | | | CALCRL | | | | | C | | | | T | | | 0.711 | | | 0.012 | | | | | | 0.002 | | | | | 8.7E-09 | | | | | | 1.34E-04 | | | | | | 33 | | | |
| rs115319174 | | | 2 | | | | 207066474 | | | | | GPR1 | | | | | G | | | | C | | | 0.943 | | | -0.041 | | | | | | 0.004 | | | | | 1.3E-22 | | | | | | 3.89E-04 | | | | | | 96 | | | |
| rs2594989 | | | 3 | | | | 11316143 | | | | | ATG7 | | | | | C | | | | T | | | 0.178 | | | 0.019 | | | | | | 0.003 | | | | | 1.8E-13 | | | | | | 2.20E-04 | | | | | | 54 | | | |
| rs754635 | | | 3 | | | | 42305131 | | | | | CCK | | | | | C | | | | G | | | 0.113 | | | -0.017 | | | | | | 0.003 | | | | | 4.8E-08 | | | | | | 1.21E-04 | | | | | | 30 | | | |
| rs2034963 | | | 3 | | | | 48170802 | | | | | CDC25A | | | | | G | | | | C | | | 0.351 | | | 0.013 | | | | | | 0.002 | | | | | 5.1E-11 | | | | | | 1.75E-04 | | | | | | 43 | | | |
| rs2629881 | | | 3 | | | | 59778271 | | | | | FHIT | | | | | C | | | | T | | | 0.221 | | | -0.013 | | | | | | 0.002 | | | | | 1.6E-08 | | | | | | 1.29E-04 | | | | | | 32 | | | |
| rs79569013 | | | 3 | | | | 61218295 | | | | | FHIT | | | | | T | | | | G | | | 0.847 | | | 0.020 | | | | | | 0.003 | | | | | 8.6E-14 | | | | | | 2.26E-04 | | | | | | 56 | | | |
| rs818219 | | | 3 | | | | 85374589 | | | | | CADM2 | | | | | T | | | | C | | | 0.541 | | | -0.013 | | | | | | 0.002 | | | | | 6.5E-12 | | | | | | 1.91E-04 | | | | | | 47 | | | |
| rs2735556 | | | 3 | | | | 88105360 | | | | | CGGBP1 | | | | | T | | | | C | | | 0.885 | | | 0.018 | | | | | | 0.003 | | | | | 1.9E-09 | | | | | | 1.46E-04 | | | | | | 36 | | | |
| rs1199328 | | | 3 | | | | 138112107 | | | | | MRAS | | | | | G | | | | A | | | 0.191 | | | 0.015 | | | | | | 0.002 | | | | | 3.2E-10 | | | | | | 1.60E-04 | | | | | | 40 | | | |
| rs76152047 | | | 3 | | | | 141183792 | | | | | ZBTB38 | | | | | A | | | | G | | | 0.934 | | | -0.025 | | | | | | 0.004 | | | | | 1.3E-10 | | | | | | 1.67E-04 | | | | | | 41 | | | |
| rs7656673 | | | 4 | | | | 30840331 | | | | | PCDH7 | | | | | A | | | | G | | | 0.598 | | | -0.014 | | | | | | 0.002 | | | | | 3.5E-12 | | | | | | 1.96E-04 | | | | | | 48 | | | |
| rs12641981 | | | 4 | | | | 45179883 | | | | | GNPDA2 | | | | | C | | | | T | | | 0.566 | | | -0.023 | | | | | | 0.002 | | | | | 1.2E-32 | | | | | | 5.74E-04 | | | | | | 142 | | | |
| rs1349641 | | | 4 | | | | 82212652 | | | | | PRKG2 | | | | | T | | | | G | | | 0.603 | | | 0.012 | | | | | | 0.002 | | | | | 2.9E-10 | | | | | | 1.61E-04 | | | | | | 40 | | | |
| rs7377083 | | | 4 | | | | 102708997 | | | | | BANK1 | | | | | C | | | | A | | | 0.570 | | | -0.015 | | | | | | 0.002 | | | | | 4.4E-15 | | | | | | 2.49E-04 | | | | | | 62 | | | |
| rs3936511 | | | 5 | | | | 55860781 | | | | | AC022431.2 | | | | | A | | | | G | | | 0.808 | | | 0.015 | | | | | | 0.002 | | | | | 4E-10 | | | | | | 1.59E-04 | | | | | | 39 | | | |
| rs10050620 | | | 5 | | | | 63927239 | | | | | RGS7BP | | | | | C | | | | T | | | 0.675 | | | 0.016 | | | | | | 0.002 | | | | | 1.3E-14 | | | | | | 2.41E-04 | | | | | | 59 | | | |
| rs13190020 | | | 5 | | | | 65012526 | | | | | SGTB | | | | | G | | | | A | | | 0.654 | | | -0.011 | | | | | | 0.002 | | | | | 1.7E-08 | | | | | | 1.29E-04 | | | | | | 32 | | | |
| rs9293494 | | | 5 | | | | 87204121 | | | | | TMEM161B | | | | | T | | | | G | | | 0.764 | | | 0.015 | | | | | | 0.002 | | | | | 3.3E-11 | | | | | | 1.78E-04 | | | | | | 44 | | | |
| rs4235642 | | | 5 | | | | 103818412 | | | | | NUDT12 | | | | | A | | | | G | | | 0.621 | | | 0.012 | | | | | | 0.002 | | | | | 5.8E-10 | | | | | | 1.56E-04 | | | | | | 38 | | | |
| rs6860760 | | | 5 | | | | 142868379 | | | | | NR3C1 | | | | | A | | | | G | | | 0.434 | | | 0.011 | | | | | | 0.002 | | | | | 3.6E-08 | | | | | | 1.23E-04 | | | | | | 30 | | | |
| rs815610 | | | 5 | | | | 153517178 | | | | | MFAP3 | | | | | C | | | | G | | | 0.443 | | | 0.015 | | | | | | 0.002 | | | | | 4.7E-15 | | | | | | 2.49E-04 | | | | | | 61 | | | |
| rs12214497 | | | 6 | | | | 10015908 | | | | | OFCC1 | | | | | G | | | | T | | | 0.656 | | | 0.014 | | | | | | 0.002 | | | | | 2.7E-11 | | | | | | 1.80E-04 | | | | | | 44 | | | |
| rs75782365 | | | 6 | | | | 26408551 | | | | | BTN3A1 | | | | | T | | | | G | | | 0.893 | | | -0.025 | | | | | | 0.003 | | | | | 7.6E-16 | | | | | | 2.63E-04 | | | | | | 65 | | | |
| rs34196306 | | | 6 | | | | 27425644 | | | | | ZNF184 | | | | | G | | | | C | | | 0.893 | | | -0.026 | | | | | | 0.003 | | | | | 9.6E-17 | | | | | | 2.80E-04 | | | | | | 69 | | | |
| rs3749971 | | | 6 | | | | 29342775 | | | | | OR5V1 | | | | | G | | | | A | | | 0.878 | | | -0.024 | | | | | | 0.003 | | | | | 7.9E-16 | | | | | | 2.63E-04 | | | | | | 65 | | | |
| rs3131934 | | | 6 | | | | 30931844 | | | | | DPCR1 | | | | | T | | | | C | | | 0.831 | | | -0.023 | | | | | | 0.003 | | | | | 1.4E-18 | | | | | | 3.14E-04 | | | | | | 77 | | | |
| rs9268235 | | | 6 | | | | 32290208 | | | | | C6orf10 | | | | | C | | | | T | | | 0.869 | | | -0.021 | | | | | | 0.003 | | | | | 1.7E-13 | | | | | | 2.20E-04 | | | | | | 54 | | | |
| rs141127771 | | | 6 | | | | 32632200 | | | | | HLA-DQB1 | | | | | G | | | | A | | | 0.741 | | | -0.016 | | | | | | 0.003 | | | | | 1.8E-09 | | | | | | 1.47E-04 | | | | | | 36 | | | |
| rs3798544 | | | 6 | | | | 34520267 | | | | | SPDEF | | | | | G | | | | A | | | 0.867 | | | -0.016 | | | | | | 0.003 | | | | | 1.7E-08 | | | | | | 1.29E-04 | | | | | | 32 | | | |
| rs2206277 | | | 6 | | | | 50798526 | | | | | TFAP2B | | | | | C | | | | T | | | 0.820 | | | -0.027 | | | | | | 0.003 | | | | | 1.4E-26 | | | | | | 4.61E-04 | | | | | | 114 | | | |
| rs1775255 | | | 6 | | | | 51243035 | | | | | PKHD1 | | | | | G | | | | T | | | 0.523 | | | -0.014 | | | | | | 0.002 | | | | | 1.9E-12 | | | | | | 2.01E-04 | | | | | | 50 | | | |
| rs12110721 | | | 6 | | | | 55190480 | | | | | GFRAL | | | | | G | | | | A | | | 0.825 | | | -0.019 | | | | | | 0.003 | | | | | 1.5E-13 | | | | | | 2.21E-04 | | | | | | 55 | | | |
| rs34260097 | | | 6 | | | | 100727703 | | | | | SIM1 | | | | | T | | | | G | | | 0.776 | | | -0.025 | | | | | | 0.002 | | | | | 1.3E-27 | | | | | | 4.80E-04 | | | | | | 119 | | | |
| rs7759938 | | | 6 | | | | 105378954 | | | | | LIN28B | | | | | C | | | | T | | | 0.322 | | | -0.012 | | | | | | 0.002 | | | | | 2.2E-08 | | | | | | 1.27E-04 | | | | | | 31 | | | |
| rs796915 | | | 6 | | | | 154304628 | | | | | OPRM1 | | | | | C | | | | G | | | 0.306 | | | -0.016 | | | | | | 0.002 | | | | | 2.6E-14 | | | | | | 2.35E-04 | | | | | | 58 | | | |
| rs62425122 | | | 6 | | | | 166311987 | | | | | PDE10A | | | | | G | | | | A | | | 0.703 | | | 0.012 | | | | | | 0.002 | | | | | 4.5E-09 | | | | | | 1.39E-04 | | | | | | 34 | | | |
| rs983949 | | | 7 | | | | 24299013 | | | | | NPY | | | | | T | | | | G | | | 0.715 | | | -0.013 | | | | | | 0.002 | | | | | 1.3E-09 | | | | | | 1.49E-04 | | | | | | 37 | | | |
| rs7808296 | | | 7 | | | | 103127620 | | | | | RELN | | | | | C | | | | T | | | 0.685 | | | -0.012 | | | | | | 0.002 | | | | | 2E-08 | | | | | | 1.28E-04 | | | | | | 31 | | | |
| rs6979832 | | | 7 | | | | 127856276 | | | | | LEP | | | | | A | | | | G | | | 0.503 | | | -0.011 | | | | | | 0.002 | | | | | 5.7E-09 | | | | | | 1.38E-04 | | | | | | 34 | | | |
| rs13233916 | | | 7 | | | | 138874416 | | | | | TTC26 | | | | | C | | | | G | | | 0.911 | | | 0.021 | | | | | | 0.003 | | | | | 5.8E-10 | | | | | | 1.56E-04 | | | | | | 38 | | | |
| rs7005216 | | | 8 | | | | 8547110 | | | | | CLDN23 | | | | | G | | | | C | | | 0.522 | | | 0.011 | | | | | | 0.002 | | | | | 8.4E-09 | | | | | | 1.35E-04 | | | | | | 33 | | | |
| rs351776 | | | 8 | | | | 28191306 | | | | | PNOC | | | | | A | | | | C | | | 0.452 | | | -0.012 | | | | | | 0.002 | | | | | 2E-09 | | | | | | 1.46E-04 | | | | | | 36 | | | |
| rs62515439 | | | 8 | | | | 57165417 | | | | | CHCHD7 | | | | | C | | | | T | | | 0.633 | | | 0.013 | | | | | | 0.002 | | | | | 2.5E-10 | | | | | | 1.62E-04 | | | | | | 40 | | | |
| rs13254613 | | | 8 | | | | 64804804 | | | | | YTHDF3 | | | | | A | | | | C | | | 0.654 | | | -0.017 | | | | | | 0.002 | | | | | 3E-16 | | | | | | 2.71E-04 | | | | | | 67 | | | |
| rs2126474 | | | 8 | | | | 76878957 | | | | | HNF4G | | | | | G | | | | T | | | 0.586 | | | 0.020 | | | | | | 0.002 | | | | | 1.9E-23 | | | | | | 4.04E-04 | | | | | | 100 | | | |
| rs10821163 | | | 9 | | | | 96343060 | | | | | PHF2 | | | | | G | | | | C | | | 0.659 | | | -0.018 | | | | | | 0.002 | | | | | 2.8E-19 | | | | | | 3.27E-04 | | | | | | 81 | | | |
| rs957512 | | | 9 | | | | 120405705 | | | | | TLR4 | | | | | T | | | | C | | | 0.669 | | | 0.012 | | | | | | 0.002 | | | | | 2.2E-08 | | | | | | 1.27E-04 | | | | | | 31 | | | |
| rs2275241 | | | 9 | | | | 129370576 | | | | | LMX1B | | | | | G | | | | A | | | 0.628 | | | -0.014 | | | | | | 0.002 | | | | | 5.5E-12 | | | | | | 1.93E-04 | | | | | | 48 | | | |
| rs7084503 | | | 10 | | | | 2666859 | | | | | PFKP | | | | | T | | | | C | | | 0.492 | | | 0.015 | | | | | | 0.002 | | | | | 2E-15 | | | | | | 2.56E-04 | | | | | | 63 | | | |
| rs76971642 | | | 10 | | | | 87575243 | | | | | GRID1 | | | | | T | | | | C | | | 0.953 | | | -0.031 | | | | | | 0.005 | | | | | 1.6E-11 | | | | | | 1.84E-04 | | | | | | 45 | | | |
| rs962369 | | | 11 | | | | 27734420 | | | | | BDNF | | | | | T | | | | C | | | 0.694 | | | -0.015 | | | | | | 0.002 | | | | | 3.5E-13 | | | | | | 2.15E-04 | | | | | | 53 | | | |
| rs661878 | | | 11 | | | | 29188691 | | | | | METTL15 | | | | | A | | | | G | | | 0.867 | | | 0.019 | | | | | | 0.003 | | | | | 3.4E-11 | | | | | | 1.78E-04 | | | | | | 44 | | | |
| rs11039307 | | | 11 | | | | 47611152 | | | | | C1QTNF4 | | | | | C | | | | T | | | 0.591 | | | -0.016 | | | | | | 0.002 | | | | | 1.7E-15 | | | | | | 2.57E-04 | | | | | | 63 | | | |
| rs678653 | | | 11 | | | | 69466737 | | | | | CCND1 | | | | | C | | | | G | | | 0.364 | | | -0.012 | | | | | | 0.002 | | | | | 1.3E-09 | | | | | | 1.49E-04 | | | | | | 37 | | | |
| rs11215403 | | | 11 | | | | 115058585 | | | | | CADM1 | | | | | G | | | | A | | | 0.757 | | | 0.016 | | | | | | 0.002 | | | | | 7.6E-13 | | | | | | 2.08E-04 | | | | | | 51 | | | |
| rs11611246 | | | 12 | | | | 939480 | | | | | WNK1 | | | | | G | | | | T | | | 0.787 | | | -0.017 | | | | | | 0.002 | | | | | 2.3E-12 | | | | | | 2.00E-04 | | | | | | 49 | | | |
| rs2187642 | | | 12 | | | | 11855624 | | | | | ETV6 | | | | | A | | | | C | | | 0.376 | | | -0.012 | | | | | | 0.002 | | | | | 2.6E-09 | | | | | | 1.44E-04 | | | | | | 35 | | | |
| rs10876457 | | | 12 | | | | 39453689 | | | | | CPNE8 | | | | | G | | | | A | | | 0.775 | | | 0.013 | | | | | | 0.002 | | | | | 3.2E-08 | | | | | | 1.24E-04 | | | | | | 31 | | | |
| rs10783302 | | | 12 | | | | 49498130 | | | | | LMBR1L | | | | | G | | | | T | | | 0.635 | | | -0.013 | | | | | | 0.002 | | | | | 4.7E-11 | | | | | | 1.76E-04 | | | | | | 43 | | | |
| rs7132908 | | | 12 | | | | 50263148 | | | | | FAIM2 | | | | | G | | | | A | | | 0.616 | | | -0.031 | | | | | | 0.002 | | | | | 8E-56 | | | | | | 1.00E-03 | | | | | | 248 | | | |
| rs76919525 | | | 12 | | | | 50541733 | | | | | CERS5 | | | | | T | | | | A | | | 0.342 | | | -0.011 | | | | | | 0.002 | | | | | 4.6E-08 | | | | | | 1.21E-04 | | | | | | 30 | | | |
| rs78607331 | | | 12 | | | | 57648644 | | | | | R3HDM2 | | | | | C | | | | T | | | 0.955 | | | -0.029 | | | | | | 0.005 | | | | | 6.2E-10 | | | | | | 1.55E-04 | | | | | | 38 | | | |
| rs10784514 | | | 12 | | | | 66452879 | | | | | LLPH | | | | | C | | | | T | | | 0.320 | | | -0.012 | | | | | | 0.002 | | | | | 2.4E-08 | | | | | | 1.26E-04 | | | | | | 31 | | | |
| rs2364232 | | | 12 | | | | 93994827 | | | | | SOCS2 | | | | | A | | | | C | | | 0.741 | | | 0.012 | | | | | | 0.002 | | | | | 2.8E-08 | | | | | | 1.25E-04 | | | | | | 31 | | | |
| rs12309017 | | | 12 | | | | 99677307 | | | | | ANKS1B | | | | | G | | | | T | | | 0.747 | | | -0.012 | | | | | | 0.002 | | | | | 2.9E-08 | | | | | | 1.25E-04 | | | | | | 31 | | | |
| rs11111647 | | | 12 | | | | 103945224 | | | | | STAB2 | | | | | G | | | | A | | | 0.792 | | | 0.013 | | | | | | 0.002 | | | | | 4.6E-08 | | | | | | 1.21E-04 | | | | | | 30 | | | |
| rs7305424 | | | 12 | | | | 118399491 | | | | | KSR2 | | | | | A | | | | T | | | 0.658 | | | -0.012 | | | | | | 0.002 | | | | | 3.9E-09 | | | | | | 1.41E-04 | | | | | | 35 | | | |
| rs35202265 | | | 13 | | | | 27986069 | | | | | GTF3A | | | | | C | | | | T | | | 0.378 | | | -0.012 | | | | | | 0.002 | | | | | 7.1E-09 | | | | | | 1.36E-04 | | | | | | 33 | | | |
| rs9551428 | | | 13 | | | | 28618462 | | | | | FLT3 | | | | | C | | | | T | | | 0.374 | | | 0.015 | | | | | | 0.002 | | | | | 2.1E-14 | | | | | | 2.37E-04 | | | | | | 58 | | | |
| rs1336486 | | | 13 | | | | 40784814 | | | | | AL133318.1 | | | | | T | | | | G | | | 0.671 | | | -0.015 | | | | | | 0.002 | | | | | 9.3E-13 | | | | | | 2.07E-04 | | | | | | 51 | | | |
| rs4477562 | | | 13 | | | | 54104968 | | | | | OLFM4 | | | | | C | | | | T | | | 0.872 | | | -0.017 | | | | | | 0.003 | | | | | 3.7E-09 | | | | | | 1.41E-04 | | | | | | 35 | | | |
| rs9317002 | | | 13 | | | | 59175727 | | | | | PCDH17 | | | | | C | | | | A | | | 0.485 | | | -0.014 | | | | | | 0.002 | | | | | 1.2E-12 | | | | | | 2.04E-04 | | | | | | 50 | | | |
| rs58681688 | | | 13 | | | | 62467001 | | | | | PCDH20 | | | | | C | | | | G | | | 0.813 | | | -0.014 | | | | | | 0.002 | | | | | 4.7E-08 | | | | | | 1.21E-04 | | | | | | 30 | | | |
| rs9540493 | | | 13 | | | | 66205704 | | | | | PCDH9 | | | | | A | | | | G | | | 0.454 | | | 0.011 | | | | | | 0.002 | | | | | 2E-08 | | | | | | 1.28E-04 | | | | | | 31 | | | |
| rs1576655 | | | 13 | | | | 79587841 | | | | | RBM26 | | | | | A | | | | C | | | 0.404 | | | -0.012 | | | | | | 0.002 | | | | | 5.8E-09 | | | | | | 1.37E-04 | | | | | | 34 | | | |
| rs61980008 | | | 14 | | | | 30464716 | | | | | PRKD1 | | | | | G | | | | A | | | 0.961 | | | -0.037 | | | | | | 0.005 | | | | | 2.9E-13 | | | | | | 2.16E-04 | | | | | | 53 | | | |
| rs1865719 | | | 14 | | | | 79923667 | | | | | NRXN3 | | | | | A | | | | G | | | 0.367 | | | -0.013 | | | | | | 0.002 | | | | | 4.5E-10 | | | | | | 1.58E-04 | | | | | | 39 | | | |
| rs8030456 | | | 15 | | | | 68076856 | | | | | MAP2K5 | | | | | C | | | | T | | | 0.773 | | | 0.023 | | | | | | 0.002 | | | | | 4.2E-24 | | | | | | 4.16E-04 | | | | | | 103 | | | |
| rs4932430 | | | 15 | | | | 89363866 | | | | | ACAN | | | | | A | | | | C | | | 0.521 | | | 0.012 | | | | | | 0.002 | | | | | 1.5E-09 | | | | | | 1.48E-04 | | | | | | 37 | | | |
| rs2970356 | | | 15 | | | | 90623540 | | | | | ZNF710 | | | | | C | | | | G | | | 0.679 | | | -0.013 | | | | | | 0.002 | | | | | 1.2E-09 | | | | | | 1.50E-04 | | | | | | 37 | | | |
| rs72755233 | | | 15 | | | | 100692953 | | | | | ADAMTS17 | | | | | G | | | | A | | | 0.888 | | | -0.018 | | | | | | 0.003 | | | | | 7.2E-09 | | | | | | 1.36E-04 | | | | | | 33 | | | |
| rs2531991 | | | 16 | | | | 4023553 | | | | | ADCY9 | | | | | G | | | | A | | | 0.253 | | | -0.020 | | | | | | 0.002 | | | | | 2.1E-19 | | | | | | 3.29E-04 | | | | | | 81 | | | |
| rs148965598 | | | 16 | | | | 19975731 | | | | | GPR139 | | | | | A | | | | G | | | 0.861 | | | 0.032 | | | | | | 0.003 | | | | | 3.1E-30 | | | | | | 5.29E-04 | | | | | | 131 | | | |
| rs4432271 | | | 16 | | | | 20245283 | | | | | GP2 | | | | | C | | | | T | | | 0.131 | | | -0.022 | | | | | | 0.003 | | | | | 1.4E-13 | | | | | | 2.22E-04 | | | | | | 55 | | | |
| rs7189927 | | | 16 | | | | 28913787 | | | | | ATP2A1 | | | | | T | | | | C | | | 0.352 | | | 0.016 | | | | | | 0.002 | | | | | 2.1E-15 | | | | | | 2.56E-04 | | | | | | 63 | | | |
| rs4889630 | | | 16 | | | | 30877544 | | | | | BCL7C | | | | | T | | | | C | | | 0.200 | | | 0.016 | | | | | | 0.002 | | | | | 2.1E-11 | | | | | | 1.82E-04 | | | | | | 45 | | | |
| rs1421085 | | | 16 | | | | 53800954 | | | | | FTO | | | | | T | | | | C | | | 0.599 | | | -0.049 | | | | | | 0.002 | | | | | 1.9E-133 | | | | | | 2.45E-03 | | | | | | 604 | | | |
| rs11863799 | | | 16 | | | | 61933401 | | | | | CDH8 | | | | | C | | | | T | | | 0.675 | | | 0.011 | | | | | | 0.002 | | | | | 3.4E-08 | | | | | | 1.23E-04 | | | | | | 30 | | | |
| rs34229857 | | | 16 | | | | 67434917 | | | | | ZDHHC1 | | | | | C | | | | T | | | 0.971 | | | -0.045 | | | | | | 0.006 | | | | | 1E-14 | | | | | | 2.43E-04 | | | | | | 60 | | | |
| rs11642090 | | | 16 | | | | 81730582 | | | | | CMIP | | | | | T | | | | C | | | 0.627 | | | -0.013 | | | | | | 0.002 | | | | | 3.4E-11 | | | | | | 1.78E-04 | | | | | | 44 | | | |
| rs242922 | | | 17 | | | | 43946370 | | | | | SPPL2C | | | | | A | | | | C | | | 0.406 | | | -0.011 | | | | | | 0.002 | | | | | 3.6E-08 | | | | | | 1.23E-04 | | | | | | 30 | | | |
| rs999493 | | | 17 | | | | 46625519 | | | | | HOXB3 | | | | | G | | | | A | | | 0.378 | | | -0.011 | | | | | | 0.002 | | | | | 2.2E-08 | | | | | | 1.27E-04 | | | | | | 31 | | | |
| rs12185242 | | | 17 | | | | 47407071 | | | | | ZNF652 | | | | | A | | | | C | | | 0.544 | | | -0.011 | | | | | | 0.002 | | | | | 1.5E-08 | | | | | | 1.30E-04 | | | | | | 32 | | | |
| rs11150745 | | | 17 | | | | 78757626 | | | | | RPTOR | | | | | A | | | | G | | | 0.682 | | | 0.012 | | | | | | 0.002 | | | | | 7.3E-09 | | | | | | 1.36E-04 | | | | | | 33 | | | |
| rs1013737 | | | 18 | | | | 937050 | | | | | ADCYAP1 | | | | | G | | | | C | | | 0.507 | | | -0.011 | | | | | | 0.002 | | | | | 2.2E-08 | | | | | | 1.27E-04 | | | | | | 31 | | | |
| rs303753 | | | 18 | | | | 21074922 | | | | | RIOK3 | | | | | G | | | | A | | | 0.655 | | | -0.012 | | | | | | 0.002 | | | | | 1.9E-08 | | | | | | 1.28E-04 | | | | | | 32 | | | |
| rs7239114 | | | 18 | | | | 45921214 | | | | | ZBTB7C | | | | | G | | | | A | | | 0.458 | | | -0.014 | | | | | | 0.002 | | | | | 5E-13 | | | | | | 2.12E-04 | | | | | | 52 | | | |
| rs12606230 | | | 18 | | | | 52492252 | | | | | RAB27B | | | | | T | | | | C | | | 0.767 | | | -0.013 | | | | | | 0.002 | | | | | 3.5E-08 | | | | | | 1.23E-04 | | | | | | 30 | | | |
| rs2168711 | | | 18 | | | | 57848531 | | | | | MC4R | | | | | T | | | | C | | | 0.767 | | | -0.035 | | | | | | 0.002 | | | | | 3.6E-54 | | | | | | 9.73E-04 | | | | | | 240 | | | |
| rs17066856 | | | 18 | | | | 58049656 | | | | | MC4R | | | | | T | | | | C | | | 0.909 | | | 0.028 | | | | | | 0.003 | | | | | 6.8E-17 | | | | | | 2.83E-04 | | | | | | 70 | | | |
| rs16982345 | | | 19 | | | | 18500722 | | | | | GDF15 | | | | | G | | | | A | | | 0.750 | | | -0.012 | | | | | | 0.002 | | | | | 2.7E-08 | | | | | | 1.25E-04 | | | | | | 31 | | | |
| rs3810304 | | | 19 | | | | 30861683 | | | | | ZNF536 | | | | | A | | | | G | | | 0.225 | | | 0.014 | | | | | | 0.002 | | | | | 6.7E-10 | | | | | | 1.55E-04 | | | | | | 38 | | | |
| rs4805881 | | | 19 | | | | 33896432 | | | | | PEPD | | | | | A | | | | C | | | 0.334 | | | -0.012 | | | | | | 0.002 | | | | | 1.2E-08 | | | | | | 1.32E-04 | | | | | | 32 | | | |
| rs3810291 | | | 19 | | | | 47569003 | | | | | ZC3H4 | | | | | G | | | | A | | | 0.324 | | | -0.015 | | | | | | 0.002 | | | | | 1E-12 | | | | | | 2.06E-04 | | | | | | 51 | | | |
| rs633372 | | | 19 | | | | 49209226 | | | | | FUT2 | | | | | G | | | | A | | | 0.466 | | | 0.011 | | | | | | 0.002 | | | | | 1.1E-08 | | | | | | 1.33E-04 | | | | | | 33 | | | |
| rs994308 | | | 20 | | | | 6603622 | | | | | BMP2 | | | | | C | | | | T | | | 0.596 | | | -0.012 | | | | | | 0.002 | | | | | 3.8E-10 | | | | | | 1.59E-04 | | | | | | 39 | | | |
| rs7268466 | | | 20 | | | | 15810676 | | | | | MACROD2 | | | | | C | | | | T | | | 0.856 | | | -0.020 | | | | | | 0.003 | | | | | 4.9E-13 | | | | | | 2.12E-04 | | | | | | 52 | | | |
| rs947088 | | | 20 | | | | 17171373 | | | | | PCSK2 | | | | | G | | | | T | | | 0.282 | | | -0.013 | | | | | | 0.002 | | | | | 6.7E-09 | | | | | | 1.36E-04 | | | | | | 34 | | | |
| rs66469746 | | | 20 | | | | 54374063 | | | | | CBLN4 | | | | | C | | | | A | | | 0.714 | | | 0.014 | | | | | | 0.002 | | | | | 2.6E-11 | | | | | | 1.80E-04 | | | | | | 44 | | | |
| rs4817973 | | | 21 | | | | 40309592 | | | | | ETS2 | | | | | G | | | | A | | | 0.640 | | | 0.014 | | | | | | 0.002 | | | | | 1.8E-11 | | | | | | 1.83E-04 | | | | | | 45 | | | |
| rs6001872 | | | 22 | | | | 40703245 | | | | | TNRC6B | | | | | A | | | | G | | | 0.651 | | | 0.014 | | | | | | 0.002 | | | | | 8.3E-12 | | | | | | 1.89E-04 | | | | | | 47 | | | |
| Genome-wide significant SNPs for adult body size in UK Biobank (overall) | | | | | | | | | | | | | | | | | | | | | | | | | | | | | | | | | | | | | | | | | | | | | | | | | | | | | | |
| SNP | | | Chr | | Position | | | | | | Closest gene | | | EA | | | | | | | | OA | | | EAF | | | | | Beta | | | | | | SE | | | | | | pvalue | | | | R^2^ | | | | | | F statistic | | |
| rs4648450 | | | 1 | | 2723214 | | | | | | TTC34 | | | C | | | | | | | | A | | | 0.533 | | | | | 0.010 | | | | | | 0.001 | | | | | | 1.2E-12 | | | | 1.11E-04 | | | | | | 50 | | |
| rs2076363 | | | 1 | | 6684906 | | | | | | THAP3 | | | C | | | | | | | | G | | | 0.663 | | | | | -0.008 | | | | | | 0.001 | | | | | | 3.1E-08 | | | | 6.76E-05 | | | | | | 31 | | |
| rs4908677 | | | 1 | | 7738180 | | | | | | CAMTA1 | | | C | | | | | | | | T | | | 0.520 | | | | | -0.008 | | | | | | 0.001 | | | | | | 2.7E-08 | | | | 6.83E-05 | | | | | | 31 | | |
| rs78886584 | | | 1 | | 16859325 | | | | | | FAM231B | | | A | | | | | | | | G | | | 0.509 | | | | | -0.008 | | | | | | 0.001 | | | | | | 8.7E-10 | | | | 8.30E-05 | | | | | | 38 | | |
| rs10799778 | | | 1 | | 23313353 | | | | | | LACTBL1 | | | T | | | | | | | | G | | | 0.166 | | | | | 0.013 | | | | | | 0.002 | | | | | | 7.8E-12 | | | | 1.03E-04 | | | | | | 47 | | |
| rs7511698 | | | 1 | | 25015638 | | | | | | SRRM1 | | | C | | | | | | | | T | | | 0.694 | | | | | 0.008 | | | | | | 0.001 | | | | | | 3.2E-08 | | | | 6.74E-05 | | | | | | 31 | | |
| rs945211 | | | 1 | | 32191798 | | | | | | BAI2 | | | G | | | | | | | | C | | | 0.384 | | | | | -0.008 | | | | | | 0.001 | | | | | | 1.6E-09 | | | | 8.03E-05 | | | | | | 36 | | |
| rs3737992 | | | 1 | | 33234128 | | | | | | KIAA1522 | | | G | | | | | | | | A | | | 0.831 | | | | | 0.014 | | | | | | 0.002 | | | | | | 5.3E-14 | | | | 1.25E-04 | | | | | | 57 | | |
| rs12031634 | | | 1 | | 34584393 | | | | | | CSMD2 | | | G | | | | | | | | A | | | 0.703 | | | | | 0.009 | | | | | | 0.002 | | | | | | 4.8E-09 | | | | 7.56E-05 | | | | | | 34 | | |
| rs116195355 | | | 1 | | 39941508 | | | | | | MACF1 | | | C | | | | | | | | A | | | 0.969 | | | | | 0.026 | | | | | | 0.004 | | | | | | 5.9E-11 | | | | 9.46E-05 | | | | | | 43 | | |
| rs2744801 | | | 1 | | 41155486 | | | | | | NFYC | | | C | | | | | | | | T | | | 0.663 | | | | | 0.008 | | | | | | 0.001 | | | | | | 4.3E-08 | | | | 6.62E-05 | | | | | | 30 | | |
| rs4660586 | | | 1 | | 42407229 | | | | | | HIVEP3 | | | C | | | | | | | | T | | | 0.261 | | | | | 0.009 | | | | | | 0.002 | | | | | | 1E-08 | | | | 7.25E-05 | | | | | | 33 | | |
| rs6669341 | | | 1 | | 47678458 | | | | | | TAL1 | | | A | | | | | | | | G | | | 0.417 | | | | | 0.011 | | | | | | 0.001 | | | | | | 1.3E-14 | | | | 1.31E-04 | | | | | | 59 | | |
| rs1167311 | | | 1 | | 49996959 | | | | | | AGBL4 | | | G | | | | | | | | A | | | 0.319 | | | | | 0.012 | | | | | | 0.001 | | | | | | 1.2E-16 | | | | 1.51E-04 | | | | | | 69 | | |
| rs630602 | | | 1 | | 54728864 | | | | | | SSBP3 | | | G | | | | | | | | C | | | 0.393 | | | | | -0.008 | | | | | | 0.001 | | | | | | 4.1E-08 | | | | 6.64E-05 | | | | | | 30 | | |
| rs12140153 | | | 1 | | 62579891 | | | | | | INADL | | | G | | | | | | | | T | | | 0.906 | | | | | 0.021 | | | | | | 0.002 | | | | | | 4.2E-19 | | | | 1.76E-04 | | | | | | 80 | | |
| rs11208659 | | | 1 | | 65979280 | | | | | | LEPR | | | T | | | | | | | | C | | | 0.917 | | | | | -0.014 | | | | | | 0.002 | | | | | | 2.1E-08 | | | | 6.93E-05 | | | | | | 31 | | |
| rs7519259 | | | 1 | | 66434743 | | | | | | PDE4B | | | G | | | | | | | | A | | | 0.472 | | | | | -0.009 | | | | | | 0.001 | | | | | | 3.2E-11 | | | | 9.73E-05 | | | | | | 44 | | |
| rs2613499 | | | 1 | | 72751552 | | | | | | NEGR1 | | | A | | | | | | | | G | | | 0.809 | | | | | 0.019 | | | | | | 0.002 | | | | | | 1.2E-26 | | | | 2.52E-04 | | | | | | 114 | | |
| rs7553158 | | | 1 | | 75005238 | | | | | | TNNI3K | | | G | | | | | | | | A | | | 0.438 | | | | | 0.011 | | | | | | 0.001 | | | | | | 2E-14 | | | | 1.29E-04 | | | | | | 58 | | |
| rs115778101 | | | 1 | | 78198554 | | | | | | USP33 | | | T | | | | | | | | C | | | 0.952 | | | | | 0.018 | | | | | | 0.003 | | | | | | 3.4E-08 | | | | 6.72E-05 | | | | | | 30 | | |
| rs34517439 | | | 1 | | 78450517 | | | | | | DNAJB4 | | | C | | | | | | | | A | | | 0.878 | | | | | -0.025 | | | | | | 0.002 | | | | | | 1.9E-31 | | | | 3.00E-04 | | | | | | 136 | | |
| rs651533 | | | 1 | | 82375561 | | | | | | LPHN2 | | | T | | | | | | | | A | | | 0.244 | | | | | 0.009 | | | | | | 0.002 | | | | | | 5.6E-09 | | | | 7.50E-05 | | | | | | 34 | | |
| rs28726372 | | | 1 | | 84353839 | | | | | | TTLL7 | | | T | | | | | | | | C | | | 0.693 | | | | | -0.009 | | | | | | 0.001 | | | | | | 9.4E-10 | | | | 8.26E-05 | | | | | | 37 | | |
| rs7548936 | | | 1 | | 91207757 | | | | | | BARHL2 | | | G | | | | | | | | C | | | 0.627 | | | | | -0.008 | | | | | | 0.001 | | | | | | 2.1E-09 | | | | 7.92E-05 | | | | | | 36 | | |
| rs6679458 | | | 1 | | 96946253 | | | | | | PTBP2 | | | G | | | | | | | | T | | | 0.409 | | | | | -0.012 | | | | | | 0.001 | | | | | | 9.1E-17 | | | | 1.53E-04 | | | | | | 69 | | |
| rs12072739 | | | 1 | | 98315893 | | | | | | DPYD | | | A | | | | | | | | G | | | 0.776 | | | | | -0.012 | | | | | | 0.002 | | | | | | 1.8E-12 | | | | 1.10E-04 | | | | | | 50 | | |
| rs41279738 | | | 1 | | 110082551 | | | | | | GPR61 | | | T | | | | | | | | G | | | 0.974 | | | | | -0.043 | | | | | | 0.004 | | | | | | 3.9E-23 | | | | 2.16E-04 | | | | | | 98 | | |
| rs12033257 | | | 1 | | 112318484 | | | | | | KCND3 | | | A | | | | | | | | G | | | 0.618 | | | | | 0.010 | | | | | | 0.001 | | | | | | 4.9E-12 | | | | 1.05E-04 | | | | | | 48 | | |
| rs7549358 | | | 1 | | 115252609 | | | | | | NRAS | | | G | | | | | | | | C | | | 0.356 | | | | | 0.009 | | | | | | 0.001 | | | | | | 6.5E-10 | | | | 8.42E-05 | | | | | | 38 | | |
| rs1409158 | | | 1 | | 119538890 | | | | | | TBX15 | | | C | | | | | | | | T | | | 0.237 | | | | | 0.009 | | | | | | 0.002 | | | | | | 1E-08 | | | | 7.25E-05 | | | | | | 33 | | |
| rs142315514 | | | 1 | | 147050816 | | | | | | BCL9 | | | C | | | | | | | | A | | | 0.965 | | | | | -0.021 | | | | | | 0.004 | | | | | | 1.7E-08 | | | | 7.03E-05 | | | | | | 32 | | |
| rs10749659 | | | 1 | | 151033979 | | | | | | MLLT11 | | | C | | | | | | | | T | | | 0.228 | | | | | 0.010 | | | | | | 0.002 | | | | | | 4.4E-10 | | | | 8.59E-05 | | | | | | 39 | | |
| rs3753639 | | | 1 | | 154986091 | | | | | | ZBTB7B | | | T | | | | | | | | C | | | 0.756 | | | | | -0.011 | | | | | | 0.002 | | | | | | 7.3E-13 | | | | 1.14E-04 | | | | | | 51 | | |
| rs61813324 | | | 1 | | 156049877 | | | | | | MEX3A | | | C | | | | | | | | T | | | 0.864 | | | | | -0.018 | | | | | | 0.002 | | | | | | 1.8E-19 | | | | 1.80E-04 | | | | | | 81 | | |
| rs1778830 | | | 1 | | 156489974 | | | | | | IQGAP3 | | | G | | | | | | | | A | | | 0.638 | | | | | -0.010 | | | | | | 0.001 | | | | | | 3.8E-12 | | | | 1.06E-04 | | | | | | 48 | | |
| rs4916229 | | | 1 | | 171443368 | | | | | | PRRC2C | | | C | | | | | | | | G | | | 0.904 | | | | | -0.015 | | | | | | 0.002 | | | | | | 8.9E-11 | | | | 9.28E-05 | | | | | | 42 | | |
| rs148137538 | | | 1 | | 173399677 | | | | | | PRDX6 | | | A | | | | | | | | G | | | 0.977 | | | | | 0.025 | | | | | | 0.005 | | | | | | 4.4E-08 | | | | 6.61E-05 | | | | | | 30 | | |
| rs77560793 | | | 1 | | 175001179 | | | | | | MRPS14 | | | G | | | | | | | | A | | | 0.969 | | | | | 0.025 | | | | | | 0.004 | | | | | | 7.8E-10 | | | | 8.34E-05 | | | | | | 38 | | |
| rs539515 | | | 1 | | 177889025 | | | | | | SEC16B | | | A | | | | | | | | C | | | 0.795 | | | | | -0.030 | | | | | | 0.002 | | | | | | 8.4E-71 | | | | 6.98E-04 | | | | | | 316 | | |
| rs9425634 | | | 1 | | 184663581 | | | | | | EDEM3 | | | T | | | | | | | | C | | | 0.542 | | | | | -0.008 | | | | | | 0.001 | | | | | | 5.7E-09 | | | | 7.49E-05 | | | | | | 34 | | |
| rs815163 | | | 1 | | 190294726 | | | | | | BRINP3 | | | T | | | | | | | | C | | | 0.437 | | | | | 0.011 | | | | | | 0.001 | | | | | | 6.1E-15 | | | | 1.34E-04 | | | | | | 61 | | |
| rs76702514 | | | 1 | | 195148296 | | | | | | KCNT2 | | | C | | | | | | | | G | | | 0.789 | | | | | 0.010 | | | | | | 0.002 | | | | | | 2E-09 | | | | 7.95E-05 | | | | | | 36 | | |
| rs2678204 | | | 1 | | 201800511 | | | | | | IPO9 | | | T | | | | | | | | G | | | 0.660 | | | | | -0.015 | | | | | | 0.001 | | | | | | 2.7E-25 | | | | 2.38E-04 | | | | | | 108 | | |
| rs4971239 | | | 1 | | 203491150 | | | | | | OPTC | | | G | | | | | | | | A | | | 0.831 | | | | | -0.011 | | | | | | 0.002 | | | | | | 4.5E-10 | | | | 8.58E-05 | | | | | | 39 | | |
| rs7539903 | | | 1 | | 209208033 | | | | | | CAMK1G | | | T | | | | | | | | A | | | 0.385 | | | | | 0.008 | | | | | | 0.001 | | | | | | 4.6E-08 | | | | 6.59E-05 | | | | | | 30 | | |
| rs78508049 | | | 1 | | 210344884 | | | | | | SYT14 | | | T | | | | | | | | C | | | 0.812 | | | | | -0.011 | | | | | | 0.002 | | | | | | 2.1E-10 | | | | 8.91E-05 | | | | | | 40 | | |
| rs12037905 | | | 1 | | 219628036 | | | | | | SLC30A10 | | | C | | | | | | | | T | | | 0.581 | | | | | 0.008 | | | | | | 0.001 | | | | | | 1.1E-08 | | | | 7.20E-05 | | | | | | 33 | | |
| rs7518221 | | | 1 | | 225561346 | | | | | | DNAH14 | | | T | | | | | | | | C | | | 0.353 | | | | | 0.008 | | | | | | 0.001 | | | | | | 2.8E-08 | | | | 6.80E-05 | | | | | | 31 | | |
| rs10779835 | | | 1 | | 230299949 | | | | | | GALNT2 | | | T | | | | | | | | C | | | 0.387 | | | | | 0.008 | | | | | | 0.001 | | | | | | 1.2E-08 | | | | 7.16E-05 | | | | | | 32 | | |
| rs10927006 | | | 1 | | 243557659 | | | | | | SDCCAG8 | | | T | | | | | | | | C | | | 0.856 | | | | | 0.012 | | | | | | 0.002 | | | | | | 8.3E-10 | | | | 8.32E-05 | | | | | | 38 | | |
| rs4658403 | | | 1 | | 243832560 | | | | | | AKT3 | | | C | | | | | | | | T | | | 0.166 | | | | | 0.014 | | | | | | 0.002 | | | | | | 1.2E-13 | | | | 1.21E-04 | | | | | | 55 | | |
| rs6752378 | | | 2 | | 25150116 | | | | | | ADCY3 | | | C | | | | | | | | A | | | 0.514 | | | | | -0.020 | | | | | | 0.001 | | | | | | 1.8E-48 | | | | 4.72E-04 | | | | | | 214 | | |
| rs1631026 | | | 2 | | 26953850 | | | | | | KCNK3 | | | C | | | | | | | | T | | | 0.527 | | | | | -0.011 | | | | | | 0.001 | | | | | | 5.9E-15 | | | | 1.34E-04 | | | | | | 61 | | |
| rs10204994 | | | 2 | | 35443726 | | | | | | CRIM1 | | | G | | | | | | | | A | | | 0.771 | | | | | 0.010 | | | | | | 0.002 | | | | | | 5.3E-10 | | | | 8.51E-05 | | | | | | 39 | | |
| rs10185199 | | | 2 | | 40282202 | | | | | | SLC8A1 | | | G | | | | | | | | A | | | 0.719 | | | | | 0.010 | | | | | | 0.002 | | | | | | 1.6E-10 | | | | 9.01E-05 | | | | | | 41 | | |
| rs10169594 | | | 2 | | 41637688 | | | | | | C2orf91 | | | T | | | | | | | | C | | | 0.637 | | | | | -0.008 | | | | | | 0.001 | | | | | | 3.3E-08 | | | | 6.74E-05 | | | | | | 31 | | |
| rs35809007 | | | 2 | | 47019521 | | | | | | LINC01118 | | | G | | | | | | | | A | | | 0.637 | | | | | 0.011 | | | | | | 0.001 | | | | | | 1.2E-13 | | | | 1.22E-04 | | | | | | 55 | | |
| rs72618637 | | | 2 | | 48953979 | | | | | | GTF2A1L | | | T | | | | | | | | A | | | 0.811 | | | | | 0.010 | | | | | | 0.002 | | | | | | 1.8E-08 | | | | 6.99E-05 | | | | | | 32 | | |
| rs6761463 | | | 2 | | 50201547 | | | | | | NRXN1 | | | G | | | | | | | | C | | | 0.162 | | | | | 0.013 | | | | | | 0.002 | | | | | | 1.3E-11 | | | | 1.01E-04 | | | | | | 46 | | |
| rs59428052 | | | 2 | | 53861389 | | | | | | GPR75-ASB3 | | | A | | | | | | | | G | | | 0.851 | | | | | 0.011 | | | | | | 0.002 | | | | | | 9.8E-09 | | | | 7.25E-05 | | | | | | 33 | | |
| rs7601895 | | | 2 | | 55281901 | | | | | | RTN4 | | | C | | | | | | | | G | | | 0.692 | | | | | 0.010 | | | | | | 0.001 | | | | | | 4.1E-12 | | | | 1.06E-04 | | | | | | 48 | | |
| rs4671328 | | | 2 | | 58935282 | | | | | | FANCL | | | T | | | | | | | | G | | | 0.449 | | | | | 0.013 | | | | | | 0.001 | | | | | | 2.1E-21 | | | | 1.99E-04 | | | | | | 90 | | |
| rs4672338 | | | 2 | | 60217457 | | | | | | BCL11A | | | C | | | | | | | | T | | | 0.664 | | | | | -0.008 | | | | | | 0.001 | | | | | | 8E-09 | | | | 7.34E-05 | | | | | | 33 | | |
| rs12477088 | | | 2 | | 67841326 | | | | | | ETAA1 | | | T | | | | | | | | C | | | 0.590 | | | | | 0.010 | | | | | | 0.001 | | | | | | 4.7E-14 | | | | 1.25E-04 | | | | | | 57 | | |
| rs6752979 | | | 2 | | 81741750 | | | | | | CTNNA2 | | | G | | | | | | | | A | | | 0.683 | | | | | -0.009 | | | | | | 0.001 | | | | | | 1.1E-09 | | | | 8.19E-05 | | | | | | 37 | | |
| rs396354 | | | 2 | | 86850022 | | | | | | CHMP3 | | | T | | | | | | | | C | | | 0.284 | | | | | 0.010 | | | | | | 0.002 | | | | | | 8E-12 | | | | 1.03E-04 | | | | | | 47 | | |
| rs11691869 | | | 2 | | 100805996 | | | | | | AFF3 | | | C | | | | | | | | A | | | 0.638 | | | | | 0.011 | | | | | | 0.001 | | | | | | 3.5E-15 | | | | 1.37E-04 | | | | | | 62 | | |
| rs1451533 | | | 2 | | 105466005 | | | | | | POU3F3 | | | G | | | | | | | | A | | | 0.726 | | | | | -0.011 | | | | | | 0.002 | | | | | | 1.3E-12 | | | | 1.11E-04 | | | | | | 50 | | |
| rs72851476 | | | 2 | | 142874787 | | | | | | LRP1B | | | A | | | | | | | | C | | | 0.844 | | | | | 0.011 | | | | | | 0.002 | | | | | | 2.8E-08 | | | | 6.80E-05 | | | | | | 31 | | |
| rs62171698 | | | 2 | | 143959096 | | | | | | ARHGAP15 | | | C | | | | | | | | A | | | 0.859 | | | | | -0.012 | | | | | | 0.002 | | | | | | 8.8E-10 | | | | 8.29E-05 | | | | | | 38 | | |
| rs75706763 | | | 2 | | 145669168 | | | | | | ZEB2 | | | A | | | | | | | | G | | | 0.948 | | | | | -0.018 | | | | | | 0.003 | | | | | | 1.7E-08 | | | | 7.03E-05 | | | | | | 32 | | |
| rs409696 | | | 2 | | 147900651 | | | | | | ACVR2A | | | G | | | | | | | | A | | | 0.424 | | | | | 0.011 | | | | | | 0.001 | | | | | | 1.8E-16 | | | | 1.50E-04 | | | | | | 68 | | |
| rs12692596 | | | 2 | | 161265910 | | | | | | RBMS1 | | | C | | | | | | | | T | | | 0.628 | | | | | -0.008 | | | | | | 0.001 | | | | | | 8.1E-09 | | | | 7.34E-05 | | | | | | 33 | | |
| rs12477385 | | | 2 | | 166144850 | | | | | | SCN2A | | | G | | | | | | | | T | | | 0.775 | | | | | 0.009 | | | | | | 0.002 | | | | | | 1.8E-08 | | | | 7.00E-05 | | | | | | 32 | | |
| rs788163 | | | 2 | | 172931559 | | | | | | METAP1D | | | A | | | | | | | | C | | | 0.724 | | | | | -0.010 | | | | | | 0.002 | | | | | | 5.9E-11 | | | | 9.45E-05 | | | | | | 43 | | |
| rs72917533 | | | 2 | | 175238924 | | | | | | CIR1 | | | T | | | | | | | | C | | | 0.814 | | | | | 0.011 | | | | | | 0.002 | | | | | | 8.2E-11 | | | | 9.31E-05 | | | | | | 42 | | |
| rs7570446 | | | 2 | | 193801010 | | | | | | TMEFF2 | | | C | | | | | | | | A | | | 0.456 | | | | | -0.008 | | | | | | 0.001 | | | | | | 1.3E-08 | | | | 7.13E-05 | | | | | | 32 | | |
| rs1704190 | | | 2 | | 200760629 | | | | | | C2orf69 | | | G | | | | | | | | A | | | 0.364 | | | | | -0.008 | | | | | | 0.001 | | | | | | 4.5E-09 | | | | 7.59E-05 | | | | | | 34 | | |
| rs4482463 | | | 2 | | 205375909 | | | | | | PARD3B | | | C | | | | | | | | A | | | 0.077 | | | | | 0.019 | | | | | | 0.003 | | | | | | 2.3E-13 | | | | 1.19E-04 | | | | | | 54 | | |
| rs79675564 | | | 2 | | 211286896 | | | | | | LANCL1 | | | C | | | | | | | | A | | | 0.922 | | | | | -0.015 | | | | | | 0.003 | | | | | | 3.4E-09 | | | | 7.70E-05 | | | | | | 35 | | |
| rs13427822 | | | 2 | | 213414265 | | | | | | ERBB4 | | | A | | | | | | | | G | | | 0.729 | | | | | 0.009 | | | | | | 0.002 | | | | | | 1.3E-09 | | | | 8.14E-05 | | | | | | 37 | | |
| rs55658481 | | | 2 | | 219284215 | | | | | | VIL1 | | | G | | | | | | | | A | | | 0.661 | | | | | -0.009 | | | | | | 0.001 | | | | | | 5.3E-10 | | | | 8.51E-05 | | | | | | 39 | | |
| rs2433733 | | | 2 | | 230816703 | | | | | | FBXO36 | | | G | | | | | | | | A | | | 0.322 | | | | | 0.011 | | | | | | 0.001 | | | | | | 7.3E-13 | | | | 1.14E-04 | | | | | | 51 | | |
| rs4663213 | | | 2 | | 236807893 | | | | | | AGAP1 | | | G | | | | | | | | A | | | 0.222 | | | | | 0.009 | | | | | | 0.002 | | | | | | 1.2E-08 | | | | 7.17E-05 | | | | | | 33 | | |
| rs112380819 | | | 3 | | 9498519 | | | | | | SETD5 | | | G | | | | | | | | A | | | 0.898 | | | | | -0.014 | | | | | | 0.002 | | | | | | 1.3E-09 | | | | 8.11E-05 | | | | | | 37 | | |
| rs34373881 | | | 3 | | 20432033 | | | | | | SGOL1 | | | G | | | | | | | | A | | | 0.725 | | | | | 0.009 | | | | | | 0.002 | | | | | | 9E-09 | | | | 7.29E-05 | | | | | | 33 | | |
| rs7619139 | | | 3 | | 25110415 | | | | | | RARB | | | T | | | | | | | | A | | | 0.411 | | | | | -0.009 | | | | | | 0.001 | | | | | | 2.4E-10 | | | | 8.86E-05 | | | | | | 40 | | |
| rs80082536 | | | 3 | | 35195311 | | | | | | ARPP21 | | | A | | | | | | | | G | | | 0.880 | | | | | -0.013 | | | | | | 0.002 | | | | | | 1.3E-09 | | | | 8.13E-05 | | | | | | 37 | | |
| rs111768603 | | | 3 | | 42329113 | | | | | | CCK | | | G | | | | | | | | T | | | 0.890 | | | | | 0.016 | | | | | | 0.002 | | | | | | 3.8E-13 | | | | 1.16E-04 | | | | | | 53 | | |
| rs28350 | | | 3 | | 42418446 | | | | | | LYZL4 | | | A | | | | | | | | G | | | 0.179 | | | | | 0.013 | | | | | | 0.002 | | | | | | 1.7E-12 | | | | 1.10E-04 | | | | | | 50 | | |
| rs78517245 | | | 3 | | 42587865 | | | | | | SEC22C | | | T | | | | | | | | C | | | 0.985 | | | | | -0.034 | | | | | | 0.006 | | | | | | 4.1E-09 | | | | 7.63E-05 | | | | | | 35 | | |
| rs113706999 | | | 3 | | 44159156 | | | | | | TOPAZ1 | | | T | | | | | | | | A | | | 0.977 | | | | | -0.029 | | | | | | 0.005 | | | | | | 1.1E-09 | | | | 8.18E-05 | | | | | | 37 | | |
| rs9852062 | | | 3 | | 45373442 | | | | | | LARS2 | | | T | | | | | | | | A | | | 0.443 | | | | | 0.008 | | | | | | 0.001 | | | | | | 3.5E-09 | | | | 7.70E-05 | | | | | | 35 | | |
| rs113569731 | | | 3 | | 47093206 | | | | | | SETD2 | | | C | | | | | | | | A | | | 0.906 | | | | | -0.015 | | | | | | 0.002 | | | | | | 3.1E-10 | | | | 8.73E-05 | | | | | | 40 | | |
| rs9843653 | | | 3 | | 49920571 | | | | | | MST1R | | | T | | | | | | | | C | | | 0.488 | | | | | -0.018 | | | | | | 0.001 | | | | | | 1.5E-37 | | | | 3.62E-04 | | | | | | 164 | | |
| rs62259692 | | | 3 | | 51847709 | | | | | | IQCF3 | | | G | | | | | | | | A | | | 0.939 | | | | | -0.018 | | | | | | 0.003 | | | | | | 1.4E-09 | | | | 8.10E-05 | | | | | | 37 | | |
| rs6798941 | | | 3 | | 52893465 | | | | | | TMEM110 | | | C | | | | | | | | T | | | 0.704 | | | | | -0.011 | | | | | | 0.002 | | | | | | 2.7E-13 | | | | 1.18E-04 | | | | | | 53 | | |
| rs6445198 | | | 3 | | 61219865 | | | | | | FHIT | | | G | | | | | | | | T | | | 0.584 | | | | | 0.011 | | | | | | 0.001 | | | | | | 3.5E-15 | | | | 1.37E-04 | | | | | | 62 | | |
| rs6445258 | | | 3 | | 62112198 | | | | | | PTPRG | | | T | | | | | | | | C | | | 0.208 | | | | | 0.010 | | | | | | 0.002 | | | | | | 8.6E-09 | | | | 7.31E-05 | | | | | | 33 | | |
| rs76824303 | | | 3 | | 62459819 | | | | | | CADPS | | | A | | | | | | | | C | | | 0.900 | | | | | 0.015 | | | | | | 0.002 | | | | | | 9.2E-11 | | | | 9.26E-05 | | | | | | 42 | | |
| rs557951 | | | 3 | | 62713263 | | | | | | CADPS | | | T | | | | | | | | G | | | 0.687 | | | | | -0.008 | | | | | | 0.001 | | | | | | 3.9E-08 | | | | 6.67E-05 | | | | | | 30 | | |
| rs11708540 | | | 3 | | 70593081 | | | | | | FOXP1 | | | G | | | | | | | | A | | | 0.842 | | | | | -0.011 | | | | | | 0.002 | | | | | | 2E-08 | | | | 6.96E-05 | | | | | | 32 | | |
| rs1598121 | | | 3 | | 82694710 | | | | | | GBE1 | | | A | | | | | | | | G | | | 0.637 | | | | | -0.009 | | | | | | 0.001 | | | | | | 3E-10 | | | | 8.76E-05 | | | | | | 40 | | |
| rs114593013 | | | 3 | | 84113491 | | | | | | CADM2 | | | A | | | | | | | | G | | | 0.942 | | | | | 0.020 | | | | | | 0.003 | | | | | | 2.1E-11 | | | | 9.89E-05 | | | | | | 45 | | |
| rs11915747 | | | 3 | | 85699040 | | | | | | CADM2 | | | C | | | | | | | | G | | | 0.647 | | | | | 0.011 | | | | | | 0.001 | | | | | | 5E-14 | | | | 1.25E-04 | | | | | | 57 | | |
| rs4858940 | | | 3 | | 88254820 | | | | | | C3orf38 | | | T | | | | | | | | C | | | 0.114 | | | | | -0.013 | | | | | | 0.002 | | | | | | 6E-10 | | | | 8.45E-05 | | | | | | 38 | | |
| rs1454687 | | | 3 | | 94038085 | | | | | | NSUN3 | | | C | | | | | | | | G | | | 0.485 | | | | | 0.012 | | | | | | 0.001 | | | | | | 3E-19 | | | | 1.77E-04 | | | | | | 80 | | |
| rs1436348 | | | 3 | | 104612668 | | | | | | ALCAM | | | A | | | | | | | | G | | | 0.417 | | | | | -0.010 | | | | | | 0.001 | | | | | | 3.5E-12 | | | | 1.07E-04 | | | | | | 48 | | |
| rs36131051 | | | 3 | | 107888841 | | | | | | IFT57 | | | T | | | | | | | | G | | | 0.795 | | | | | 0.011 | | | | | | 0.002 | | | | | | 2.9E-10 | | | | 8.78E-05 | | | | | | 40 | | |
| rs9814758 | | | 3 | | 123062657 | | | | | | ADCY5 | | | T | | | | | | | | G | | | 0.644 | | | | | 0.008 | | | | | | 0.001 | | | | | | 5.4E-09 | | | | 7.51E-05 | | | | | | 34 | | |
| rs1320903 | | | 3 | | 131758077 | | | | | | CPNE4 | | | G | | | | | | | | A | | | 0.680 | | | | | -0.015 | | | | | | 0.001 | | | | | | 2.6E-23 | | | | 2.18E-04 | | | | | | 99 | | |
| rs10935143 | | | 3 | | 134665159 | | | | | | EPHB1 | | | G | | | | | | | | A | | | 0.551 | | | | | 0.008 | | | | | | 0.001 | | | | | | 1.1E-09 | | | | 8.18E-05 | | | | | | 37 | | |
| rs2343681 | | | 3 | | 136535024 | | | | | | SLC35G2 | | | G | | | | | | | | A | | | 0.211 | | | | | -0.012 | | | | | | 0.002 | | | | | | 1.8E-13 | | | | 1.20E-04 | | | | | | 54 | | |
| rs2035936 | | | 3 | | 141298124 | | | | | | RASA2 | | | G | | | | | | | | T | | | 0.944 | | | | | -0.022 | | | | | | 0.003 | | | | | | 2.3E-13 | | | | 1.19E-04 | | | | | | 54 | | |
| rs12634936 | | | 3 | | 147716498 | | | | | | ZIC1 | | | T | | | | | | | | C | | | 0.943 | | | | | -0.018 | | | | | | 0.003 | | | | | | 8.8E-09 | | | | 7.30E-05 | | | | | | 33 | | |
| rs1568488 | | | 3 | | 153657951 | | | | | | ARHGEF26 | | | G | | | | | | | | C | | | 0.405 | | | | | -0.011 | | | | | | 0.001 | | | | | | 4.4E-15 | | | | 1.36E-04 | | | | | | 61 | | |
| rs9834519 | | | 3 | | 156379637 | | | | | | TIPARP | | | C | | | | | | | | T | | | 0.919 | | | | | 0.016 | | | | | | 0.003 | | | | | | 8.4E-10 | | | | 8.31E-05 | | | | | | 38 | | |
| rs12630209 | | | 3 | | 156881392 | | | | | | CCNL1 | | | T | | | | | | | | G | | | 0.747 | | | | | -0.009 | | | | | | 0.002 | | | | | | 3E-08 | | | | 6.77E-05 | | | | | | 31 | | |
| rs8192675 | | | 3 | | 170724883 | | | | | | SLC2A2 | | | T | | | | | | | | C | | | 0.711 | | | | | -0.012 | | | | | | 0.002 | | | | | | 8.7E-15 | | | | 1.33E-04 | | | | | | 60 | | |
| rs529200 | | | 3 | | 173114305 | | | | | | NLGN1 | | | A | | | | | | | | G | | | 0.472 | | | | | -0.010 | | | | | | 0.001 | | | | | | 5.8E-14 | | | | 1.25E-04 | | | | | | 56 | | |
| rs13061117 | | | 3 | | 181186466 | | | | | | SOX2 | | | T | | | | | | | | C | | | 0.910 | | | | | -0.015 | | | | | | 0.002 | | | | | | 1.3E-09 | | | | 8.13E-05 | | | | | | 37 | | |
| rs262956 | | | 3 | | 183486117 | | | | | | YEATS2 | | | T | | | | | | | | G | | | 0.353 | | | | | 0.009 | | | | | | 0.001 | | | | | | 2.2E-10 | | | | 8.89E-05 | | | | | | 40 | | |
| rs869400 | | | 3 | | 185826740 | | | | | | ETV5 | | | T | | | | | | | | G | | | 0.185 | | | | | -0.018 | | | | | | 0.002 | | | | | | 3.5E-25 | | | | 2.37E-04 | | | | | | 107 | | |
| rs80236973 | | | 3 | | 188001014 | | | | | | LPP | | | C | | | | | | | | T | | | 0.865 | | | | | 0.012 | | | | | | 0.002 | | | | | | 1.4E-09 | | | | 8.09E-05 | | | | | | 37 | | |
| rs4677813 | | | 3 | | 194863860 | | | | | | XXYLT1 | | | T | | | | | | | | C | | | 0.750 | | | | | 0.009 | | | | | | 0.002 | | | | | | 1.6E-08 | | | | 7.04E-05 | | | | | | 32 | | |
| rs6583310 | | | 3 | | 196170985 | | | | | | UBXN7 | | | G | | | | | | | | C | | | 0.561 | | | | | -0.009 | | | | | | 0.001 | | | | | | 2.2E-10 | | | | 8.89E-05 | | | | | | 40 | | |
| rs2051559 | | | 4 | | 3298800 | | | | | | RGS12 | | | T | | | | | | | | C | | | 0.868 | | | | | -0.014 | | | | | | 0.002 | | | | | | 8.3E-12 | | | | 1.03E-04 | | | | | | 47 | | |
| rs35852935 | | | 4 | | 17991522 | | | | | | LCORL | | | A | | | | | | | | C | | | 0.964 | | | | | -0.022 | | | | | | 0.004 | | | | | | 4.9E-09 | | | | 7.55E-05 | | | | | | 34 | | |
| rs1477890 | | | 4 | | 18511738 | | | | | | LCORL | | | A | | | | | | | | G | | | 0.507 | | | | | -0.010 | | | | | | 0.001 | | | | | | 1.1E-12 | | | | 1.12E-04 | | | | | | 51 | | |
| rs34811474 | | | 4 | | 25408838 | | | | | | ANAPC4 | | | G | | | | | | | | A | | | 0.769 | | | | | 0.017 | | | | | | 0.002 | | | | | | 2E-24 | | | | 2.30E-04 | | | | | | 104 | | |
| rs73213484 | | | 4 | | 28489339 | | | | | | RP11-180C1.1 | | | A | | | | | | | | T | | | 0.859 | | | | | 0.015 | | | | | | 0.002 | | | | | | 1.1E-13 | | | | 1.22E-04 | | | | | | 55 | | |
| rs4527444 | | | 4 | | 30842780 | | | | | | PCDH7 | | | A | | | | | | | | G | | | 0.459 | | | | | -0.009 | | | | | | 0.001 | | | | | | 9E-12 | | | | 1.03E-04 | | | | | | 47 | | |
| rs36023504 | | | 4 | | 38698924 | | | | | | KLF3 | | | C | | | | | | | | T | | | 0.617 | | | | | 0.008 | | | | | | 0.001 | | | | | | 4.6E-08 | | | | 6.59E-05 | | | | | | 30 | | |
| rs12507026 | | | 4 | | 45181334 | | | | | | GNPDA2 | | | A | | | | | | | | T | | | 0.565 | | | | | -0.019 | | | | | | 0.001 | | | | | | 1.8E-41 | | | | 4.01E-04 | | | | | | 182 | | |
| rs2237025 | | | 4 | | 55541879 | | | | | | KIT | | | T | | | | | | | | C | | | 0.445 | | | | | 0.010 | | | | | | 0.001 | | | | | | 1.8E-13 | | | | 1.20E-04 | | | | | | 54 | | |
| rs28462076 | | | 4 | | 65696174 | | | | | | TECRL | | | A | | | | | | | | G | | | 0.762 | | | | | 0.009 | | | | | | 0.002 | | | | | | 6.7E-09 | | | | 7.42E-05 | | | | | | 34 | | |
| rs2164300 | | | 4 | | 67813017 | | | | | | CENPC | | | C | | | | | | | | T | | | 0.481 | | | | | 0.008 | | | | | | 0.001 | | | | | | 4.2E-08 | | | | 6.64E-05 | | | | | | 30 | | |
| rs13104584 | | | 4 | | 80811227 | | | | | | ANTXR2 | | | G | | | | | | | | A | | | 0.592 | | | | | -0.009 | | | | | | 0.001 | | | | | | 6.2E-10 | | | | 8.44E-05 | | | | | | 38 | | |
| rs4148155 | | | 4 | | 89054667 | | | | | | ABCG2 | | | A | | | | | | | | G | | | 0.887 | | | | | 0.014 | | | | | | 0.002 | | | | | | 2.9E-11 | | | | 9.76E-05 | | | | | | 44 | | |
| rs4419475 | | | 4 | | 96150044 | | | | | | UNC5C | | | A | | | | | | | | T | | | 0.593 | | | | | -0.008 | | | | | | 0.001 | | | | | | 6.5E-09 | | | | 7.43E-05 | | | | | | 34 | | |
| rs1229984 | | | 4 | | 100239319 | | | | | | ADH1B | | | T | | | | | | | | C | | | 0.027 | | | | | -0.023 | | | | | | 0.004 | | | | | | 3.2E-08 | | | | 6.75E-05 | | | | | | 31 | | |
| rs2583410 | | | 4 | | 102182199 | | | | | | PPP3CA | | | A | | | | | | | | C | | | 0.853 | | | | | -0.013 | | | | | | 0.002 | | | | | | 5.3E-12 | | | | 1.05E-04 | | | | | | 48 | | |
| rs13107325 | | | 4 | | 103188709 | | | | | | SLC39A8 | | | C | | | | | | | | T | | | 0.925 | | | | | -0.029 | | | | | | 0.003 | | | | | | 3.1E-28 | | | | 2.68E-04 | | | | | | 121 | | |
| rs1381010 | | | 4 | | 112677085 | | | | | | C4orf32 | | | G | | | | | | | | A | | | 0.695 | | | | | 0.008 | | | | | | 0.001 | | | | | | 3E-08 | | | | 6.77E-05 | | | | | | 31 | | |
| rs2952863 | | | 4 | | 130759647 | | | | | | C4orf33 | | | T | | | | | | | | G | | | 0.291 | | | | | 0.010 | | | | | | 0.002 | | | | | | 1.6E-10 | | | | 9.02E-05 | | | | | | 41 | | |
| rs1296328 | | | 4 | | 137083193 | | | | | | PCDH18 | | | A | | | | | | | | C | | | 0.441 | | | | | 0.012 | | | | | | 0.001 | | | | | | 2.6E-17 | | | | 1.58E-04 | | | | | | 72 | | |
| rs809955 | | | 4 | | 140874760 | | | | | | MAML3 | | | G | | | | | | | | A | | | 0.634 | | | | | 0.010 | | | | | | 0.001 | | | | | | 1.4E-12 | | | | 1.11E-04 | | | | | | 50 | | |
| rs35390852 | | | 4 | | 143067054 | | | | | | INPP4B | | | G | | | | | | | | A | | | 0.876 | | | | | -0.011 | | | | | | 0.002 | | | | | | 4.8E-08 | | | | 6.57E-05 | | | | | | 30 | | |
| rs12644329 | | | 4 | | 143634746 | | | | | | INPP4B | | | G | | | | | | | | A | | | 0.372 | | | | | 0.008 | | | | | | 0.001 | | | | | | 2.5E-08 | | | | 6.86E-05 | | | | | | 31 | | |
| rs6823268 | | | 4 | | 145982563 | | | | | | ANAPC10 | | | A | | | | | | | | G | | | 0.629 | | | | | -0.008 | | | | | | 0.001 | | | | | | 8.2E-09 | | | | 7.33E-05 | | | | | | 33 | | |
| rs113079574 | | | 4 | | 147354089 | | | | | | SLC10A7 | | | C | | | | | | | | T | | | 0.807 | | | | | 0.010 | | | | | | 0.002 | | | | | | 3.3E-08 | | | | 6.74E-05 | | | | | | 31 | | |
| rs6843852 | | | 4 | | 162132758 | | | | | | FSTL5 | | | C | | | | | | | | T | | | 0.492 | | | | | -0.009 | | | | | | 0.001 | | | | | | 2E-10 | | | | 8.92E-05 | | | | | | 40 | | |
| rs698147 | | | 5 | | 3513485 | | | | | | IRX1 | | | A | | | | | | | | G | | | 0.456 | | | | | 0.009 | | | | | | 0.001 | | | | | | 1.8E-10 | | | | 8.97E-05 | | | | | | 41 | | |
| rs67913249 | | | 5 | | 43204126 | | | | | | NIM1K | | | C | | | | | | | | G | | | 0.658 | | | | | 0.009 | | | | | | 0.001 | | | | | | 1.2E-10 | | | | 9.17E-05 | | | | | | 42 | | |
| rs114263339 | | | 5 | | 50932343 | | | | | | ISL1 | | | C | | | | | | | | T | | | 0.974 | | | | | -0.025 | | | | | | 0.004 | | | | | | 5.2E-09 | | | | 7.53E-05 | | | | | | 34 | | |
| rs10805383 | | | 5 | | 63034606 | | | | | | HTR1A | | | G | | | | | | | | A | | | 0.519 | | | | | -0.010 | | | | | | 0.001 | | | | | | 2.6E-14 | | | | 1.28E-04 | | | | | | 58 | | |
| rs9291822 | | | 5 | | 64076515 | | | | | | CWC27 | | | C | | | | | | | | T | | | 0.485 | | | | | 0.008 | | | | | | 0.001 | | | | | | 1.1E-08 | | | | 7.20E-05 | | | | | | 33 | | |
| rs27215 | | | 5 | | 66219318 | | | | | | MAST4 | | | C | | | | | | | | A | | | 0.275 | | | | | -0.010 | | | | | | 0.002 | | | | | | 5.1E-11 | | | | 9.52E-05 | | | | | | 43 | | |
| rs2307111 | | | 5 | | 75003678 | | | | | | POC5 | | | T | | | | | | | | C | | | 0.605 | | | | | 0.017 | | | | | | 0.001 | | | | | | 6.9E-33 | | | | 3.15E-04 | | | | | | 143 | | |
| rs252749 | | | 5 | | 77389973 | | | | | | AP3B1 | | | G | | | | | | | | A | | | 0.754 | | | | | 0.010 | | | | | | 0.002 | | | | | | 2.1E-09 | | | | 7.91E-05 | | | | | | 36 | | |
| rs59893724 | | | 5 | | 80830788 | | | | | | SSBP2 | | | A | | | | | | | | G | | | 0.756 | | | | | 0.011 | | | | | | 0.002 | | | | | | 8.7E-13 | | | | 1.13E-04 | | | | | | 51 | | |
| rs79236537 | | | 5 | | 86727690 | | | | | | CCNH | | | G | | | | | | | | T | | | 0.980 | | | | | -0.030 | | | | | | 0.005 | | | | | | 1E-09 | | | | 8.23E-05 | | | | | | 37 | | |
| rs6870983 | | | 5 | | 87697533 | | | | | | TMEM161B | | | C | | | | | | | | T | | | 0.786 | | | | | 0.014 | | | | | | 0.002 | | | | | | 1.6E-16 | | | | 1.50E-04 | | | | | | 68 | | |
| rs1477290 | | | 5 | | 87988934 | | | | | | MEF2C | | | T | | | | | | | | C | | | 0.863 | | | | | -0.020 | | | | | | 0.002 | | | | | | 3.5E-23 | | | | 2.17E-04 | | | | | | 98 | | |
| rs142503704 | | | 5 | | 92622421 | | | | | | NR2F1 | | | G | | | | | | | | A | | | 0.977 | | | | | -0.026 | | | | | | 0.005 | | | | | | 1.9E-08 | | | | 6.97E-05 | | | | | | 32 | | |
| rs62379271 | | | 5 | | 105870033 | | | | | | EFNA5 | | | T | | | | | | | | G | | | 0.421 | | | | | -0.008 | | | | | | 0.001 | | | | | | 2E-08 | | | | 6.94E-05 | | | | | | 31 | | |
| rs149457 | | | 5 | | 107438057 | | | | | | FBXL17 | | | C | | | | | | | | T | | | 0.830 | | | | | 0.015 | | | | | | 0.002 | | | | | | 2.1E-16 | | | | 1.49E-04 | | | | | | 67 | | |
| rs12517187 | | | 5 | | 112444682 | | | | | | MCC | | | C | | | | | | | | T | | | 0.565 | | | | | -0.008 | | | | | | 0.001 | | | | | | 2.8E-09 | | | | 7.79E-05 | | | | | | 35 | | |
| rs347551 | | | 5 | | 119389031 | | | | | | PRR16 | | | C | | | | | | | | G | | | 0.528 | | | | | -0.009 | | | | | | 0.001 | | | | | | 1.8E-10 | | | | 8.97E-05 | | | | | | 41 | | |
| rs1582931 | | | 5 | | 122657199 | | | | | | CEP120 | | | G | | | | | | | | A | | | 0.527 | | | | | 0.010 | | | | | | 0.001 | | | | | | 3E-12 | | | | 1.07E-04 | | | | | | 49 | | |
| rs4836133 | | | 5 | | 124332103 | | | | | | ZNF608 | | | C | | | | | | | | A | | | 0.510 | | | | | -0.008 | | | | | | 0.001 | | | | | | 3.1E-09 | | | | 7.75E-05 | | | | | | 35 | | |
| rs329118 | | | 5 | | 133861663 | | | | | | JADE2 | | | C | | | | | | | | T | | | 0.581 | | | | | 0.009 | | | | | | 0.001 | | | | | | 8.8E-12 | | | | 1.03E-04 | | | | | | 47 | | |
| rs13174863 | | | 5 | | 139080745 | | | | | | CXXC5 | | | A | | | | | | | | G | | | 0.852 | | | | | -0.014 | | | | | | 0.002 | | | | | | 2.4E-12 | | | | 1.08E-04 | | | | | | 49 | | |
| rs7719067 | | | 5 | | 153538241 | | | | | | MFAP3 | | | A | | | | | | | | G | | | 0.428 | | | | | 0.009 | | | | | | 0.001 | | | | | | 1.7E-11 | | | | 9.99E-05 | | | | | | 45 | | |
| rs11134512 | | | 5 | | 167847460 | | | | | | WWC1 | | | T | | | | | | | | G | | | 0.677 | | | | | 0.008 | | | | | | 0.001 | | | | | | 1.9E-08 | | | | 6.96E-05 | | | | | | 32 | | |
| rs11134679 | | | 5 | | 170623391 | | | | | | RANBP17 | | | A | | | | | | | | G | | | 0.315 | | | | | -0.012 | | | | | | 0.001 | | | | | | 5.2E-17 | | | | 1.55E-04 | | | | | | 70 | | |
| rs4467770 | | | 6 | | 12086826 | | | | | | HIVEP1 | | | G | | | | | | | | A | | | 0.269 | | | | | -0.010 | | | | | | 0.002 | | | | | | 2E-10 | | | | 8.92E-05 | | | | | | 40 | | |
| rs9395520 | | | 6 | | 13183523 | | | | | | PHACTR1 | | | C | | | | | | | | T | | | 0.696 | | | | | 0.010 | | | | | | 0.001 | | | | | | 5.9E-11 | | | | 9.46E-05 | | | | | | 43 | | |
| rs3806114 | | | 6 | | 20482335 | | | | | | E2F3 | | | G | | | | | | | | A | | | 0.332 | | | | | 0.009 | | | | | | 0.001 | | | | | | 2.3E-09 | | | | 7.87E-05 | | | | | | 36 | | |
| rs75499503 | | | 6 | | 26145217 | | | | | | HIST1H2AC | | | C | | | | | | | | T | | | 0.780 | | | | | 0.011 | | | | | | 0.002 | | | | | | 8E-12 | | | | 1.03E-04 | | | | | | 47 | | |
| rs34388845 | | | 6 | | 28578286 | | | | | | SCAND3 | | | A | | | | | | | | G | | | 0.786 | | | | | -0.012 | | | | | | 0.002 | | | | | | 1.9E-13 | | | | 1.19E-04 | | | | | | 54 | | |
| rs2260051 | | | 6 | | 31591918 | | | | | | PRRC2A | | | A | | | | | | | | T | | | 0.438 | | | | | -0.012 | | | | | | 0.001 | | | | | | 1.6E-17 | | | | 1.60E-04 | | | | | | 73 | | |
| rs9277992 | | | 6 | | 33312455 | | | | | | DAXX | | | G | | | | | | | | A | | | 0.810 | | | | | -0.013 | | | | | | 0.002 | | | | | | 5.7E-14 | | | | 1.25E-04 | | | | | | 56 | | |
| rs9366863 | | | 6 | | 34688946 | | | | | | C6orf106 | | | T | | | | | | | | C | | | 0.328 | | | | | 0.017 | | | | | | 0.001 | | | | | | 8.5E-33 | | | | 3.14E-04 | | | | | | 142 | | |
| rs34298980 | | | 6 | | 40409243 | | | | | | LRFN2 | | | T | | | | | | | | C | | | 0.491 | | | | | 0.013 | | | | | | 0.001 | | | | | | 4.4E-20 | | | | 1.86E-04 | | | | | | 84 | | |
| rs9462670 | | | 6 | | 41014309 | | | | | | OARD1 | | | G | | | | | | | | C | | | 0.769 | | | | | -0.009 | | | | | | 0.002 | | | | | | 7.1E-09 | | | | 7.39E-05 | | | | | | 34 | | |
| rs72892910 | | | 6 | | 50816887 | | | | | | TFAP2B | | | G | | | | | | | | T | | | 0.828 | | | | | -0.025 | | | | | | 0.002 | | | | | | 5.7E-44 | | | | 4.27E-04 | | | | | | 193 | | |
| rs1327259 | | | 6 | | 51177811 | | | | | | PKHD1 | | | A | | | | | | | | G | | | 0.612 | | | | | 0.010 | | | | | | 0.001 | | | | | | 1.3E-13 | | | | 1.21E-04 | | | | | | 55 | | |
| rs1547026 | | | 6 | | 51825622 | | | | | | PKHD1 | | | T | | | | | | | | C | | | 0.709 | | | | | -0.010 | | | | | | 0.002 | | | | | | 6.8E-12 | | | | 1.04E-04 | | | | | | 47 | | |
| rs72910629 | | | 6 | | 69761994 | | | | | | BAI3 | | | A | | | | | | | | G | | | 0.864 | | | | | -0.013 | | | | | | 0.002 | | | | | | 4.3E-11 | | | | 9.59E-05 | | | | | | 43 | | |
| rs1040046 | | | 6 | | 83473573 | | | | | | UBE3D | | | C | | | | | | | | A | | | 0.851 | | | | | -0.011 | | | | | | 0.002 | | | | | | 4.6E-09 | | | | 7.58E-05 | | | | | | 34 | | |
| rs1324110 | | | 6 | | 93913200 | | | | | | EPHA7 | | | G | | | | | | | | C | | | 0.557 | | | | | 0.008 | | | | | | 0.001 | | | | | | 2E-08 | | | | 6.95E-05 | | | | | | 32 | | |
| rs10499014 | | | 6 | | 97947755 | | | | | | MMS22L | | | C | | | | | | | | G | | | 0.731 | | | | | 0.010 | | | | | | 0.002 | | | | | | 1.1E-10 | | | | 9.20E-05 | | | | | | 42 | | |
| rs6938973 | | | 6 | | 98421721 | | | | | | MMS22L | | | T | | | | | | | | C | | | 0.399 | | | | | -0.012 | | | | | | 0.001 | | | | | | 3.5E-17 | | | | 1.57E-04 | | | | | | 71 | | |
| rs9496567 | | | 6 | | 100602753 | | | | | | MCHR2 | | | G | | | | | | | | A | | | 0.757 | | | | | -0.009 | | | | | | 0.002 | | | | | | 4.4E-09 | | | | 7.60E-05 | | | | | | 34 | | |
| rs156126 | | | 6 | | 104810083 | | | | | | HACE1 | | | T | | | | | | | | C | | | 0.196 | | | | | -0.010 | | | | | | 0.002 | | | | | | 2.7E-09 | | | | 7.82E-05 | | | | | | 35 | | |
| rs2253310 | | | 6 | | 108888593 | | | | | | FOXO3 | | | C | | | | | | | | G | | | 0.374 | | | | | -0.011 | | | | | | 0.001 | | | | | | 9E-14 | | | | 1.23E-04 | | | | | | 56 | | |
| rs13218383 | | | 6 | | 120173501 | | | | | | MAN1A1 | | | C | | | | | | | | G | | | 0.665 | | | | | 0.009 | | | | | | 0.001 | | | | | | 1.4E-10 | | | | 9.09E-05 | | | | | | 41 | | |
| rs2875762 | | | 6 | | 124925032 | | | | | | NKAIN2 | | | G | | | | | | | | C | | | 0.757 | | | | | -0.011 | | | | | | 0.002 | | | | | | 6E-12 | | | | 1.04E-04 | | | | | | 47 | | |
| rs10457469 | | | 6 | | 126083658 | | | | | | HEY2 | | | G | | | | | | | | A | | | 0.476 | | | | | -0.008 | | | | | | 0.001 | | | | | | 2.6E-08 | | | | 6.83E-05 | | | | | | 31 | | |
| rs12213441 | | | 6 | | 143208838 | | | | | | HIVEP2 | | | C | | | | | | | | T | | | 0.771 | | | | | -0.011 | | | | | | 0.002 | | | | | | 3.3E-10 | | | | 8.71E-05 | | | | | | 39 | | |
| rs7749708 | | | 6 | | 153375907 | | | | | | RGS17 | | | C | | | | | | | | T | | | 0.706 | | | | | -0.010 | | | | | | 0.002 | | | | | | 3.7E-11 | | | | 9.66E-05 | | | | | | 44 | | |
| rs9478496 | | | 6 | | 154333183 | | | | | | OPRM1 | | | T | | | | | | | | C | | | 0.836 | | | | | -0.012 | | | | | | 0.002 | | | | | | 3.8E-10 | | | | 8.65E-05 | | | | | | 39 | | |
| rs9480184 | | | 6 | | 155987788 | | | | | | NOX3 | | | C | | | | | | | | T | | | 0.789 | | | | | -0.010 | | | | | | 0.002 | | | | | | 4.9E-09 | | | | 7.55E-05 | | | | | | 34 | | |
| rs36007635 | | | 6 | | 163009335 | | | | | | PARK2 | | | G | | | | | | | | A | | | 0.862 | | | | | 0.014 | | | | | | 0.002 | | | | | | 5.6E-12 | | | | 1.05E-04 | | | | | | 47 | | |
| rs6950388 | | | 7 | | 1270699 | | | | | | UNCX | | | G | | | | | | | | A | | | 0.205 | | | | | -0.009 | | | | | | 0.002 | | | | | | 2.6E-08 | | | | 6.83E-05 | | | | | | 31 | | |
| rs2056477 | | | 7 | | 2079744 | | | | | | MAD1L1 | | | G | | | | | | | | C | | | 0.772 | | | | | 0.012 | | | | | | 0.002 | | | | | | 1.5E-12 | | | | 1.10E-04 | | | | | | 50 | | |
| rs4307239 | | | 7 | | 24354300 | | | | | | NPY | | | A | | | | | | | | G | | | 0.541 | | | | | -0.008 | | | | | | 0.001 | | | | | | 2.4E-08 | | | | 6.86E-05 | | | | | | 31 | | |
| rs215634 | | | 7 | | 32369148 | | | | | | PDE1C | | | A | | | | | | | | G | | | 0.388 | | | | | 0.010 | | | | | | 0.001 | | | | | | 1.4E-13 | | | | 1.21E-04 | | | | | | 55 | | |
| rs2237402 | | | 7 | | 39449768 | | | | | | POU6F2 | | | G | | | | | | | | A | | | 0.661 | | | | | 0.009 | | | | | | 0.001 | | | | | | 1.8E-10 | | | | 8.98E-05 | | | | | | 41 | | |
| rs2289379 | | | 7 | | 44804225 | | | | | | ZMIZ2 | | | C | | | | | | | | T | | | 0.604 | | | | | 0.010 | | | | | | 0.001 | | | | | | 4.1E-12 | | | | 1.06E-04 | | | | | | 48 | | |
| rs3823674 | | | 7 | | 50571996 | | | | | | DDC | | | C | | | | | | | | T | | | 0.572 | | | | | 0.008 | | | | | | 0.001 | | | | | | 3.5E-08 | | | | 6.71E-05 | | | | | | 30 | | |
| rs11765062 | | | 7 | | 54417515 | | | | | | VSTM2A | | | T | | | | | | | | C | | | 0.459 | | | | | 0.008 | | | | | | 0.001 | | | | | | 4.6E-08 | | | | 6.59E-05 | | | | | | 30 | | |
| rs2866720 | | | 7 | | 70106310 | | | | | | AUTS2 | | | C | | | | | | | | T | | | 0.615 | | | | | -0.009 | | | | | | 0.001 | | | | | | 1.2E-10 | | | | 9.14E-05 | | | | | | 41 | | |
| rs1852006 | | | 7 | | 77829768 | | | | | | MAGI2 | | | G | | | | | | | | A | | | 0.643 | | | | | 0.009 | | | | | | 0.001 | | | | | | 6.7E-10 | | | | 8.41E-05 | | | | | | 38 | | |
| rs6963840 | | | 7 | | 78144371 | | | | | | MAGI2 | | | C | | | | | | | | T | | | 0.844 | | | | | -0.013 | | | | | | 0.002 | | | | | | 8.5E-12 | | | | 1.03E-04 | | | | | | 47 | | |
| rs12538826 | | | 7 | | 99030228 | | | | | | PTCD1 | | | T | | | | | | | | C | | | 0.886 | | | | | 0.017 | | | | | | 0.002 | | | | | | 7.2E-15 | | | | 1.34E-04 | | | | | | 61 | | |
| rs2074686 | | | 7 | | 100800635 | | | | | | AP1S1 | | | G | | | | | | | | A | | | 0.410 | | | | | 0.008 | | | | | | 0.001 | | | | | | 2.9E-09 | | | | 7.78E-05 | | | | | | 35 | | |
| rs11496125 | | | 7 | | 103417557 | | | | | | RELN | | | C | | | | | | | | T | | | 0.575 | | | | | -0.011 | | | | | | 0.001 | | | | | | 4.8E-15 | | | | 1.35E-04 | | | | | | 61 | | |
| rs2396625 | | | 7 | | 113028634 | | | | | | TSRM | | | T | | | | | | | | A | | | 0.579 | | | | | 0.010 | | | | | | 0.001 | | | | | | 1.1E-12 | | | | 1.12E-04 | | | | | | 51 | | |
| rs12705894 | | | 7 | | 113351252 | | | | | | PPP1R3A | | | G | | | | | | | | A | | | 0.448 | | | | | 0.008 | | | | | | 0.001 | | | | | | 1.6E-08 | | | | 7.03E-05 | | | | | | 32 | | |
| rs1840660 | | | 7 | | 114352615 | | | | | | FOXP2 | | | G | | | | | | | | A | | | 0.612 | | | | | -0.010 | | | | | | 0.001 | | | | | | 2E-12 | | | | 1.09E-04 | | | | | | 50 | | |
| rs1899689 | | | 7 | | 121964349 | | | | | | CADPS2 | | | C | | | | | | | | T | | | 0.610 | | | | | -0.008 | | | | | | 0.001 | | | | | | 3.7E-08 | | | | 6.69E-05 | | | | | | 30 | | |
| rs35775580 | | | 7 | | 130420740 | | | | | | KLF14 | | | A | | | | | | | | G | | | 0.953 | | | | | 0.018 | | | | | | 0.003 | | | | | | 2.7E-08 | | | | 6.82E-05 | | | | | | 31 | | |
| rs11976084 | | | 7 | | 137437156 | | | | | | DGKI | | | C | | | | | | | | T | | | 0.712 | | | | | -0.009 | | | | | | 0.002 | | | | | | 1.7E-08 | | | | 7.02E-05 | | | | | | 32 | | |
| rs11525873 | | | 7 | | 138817193 | | | | | | TTC26 | | | T | | | | | | | | C | | | 0.902 | | | | | 0.015 | | | | | | 0.002 | | | | | | 1.4E-10 | | | | 9.07E-05 | | | | | | 41 | | |
| rs1805123 | | | 7 | | 150645534 | | | | | | KCNH2 | | | T | | | | | | | | G | | | 0.755 | | | | | 0.011 | | | | | | 0.002 | | | | | | 4.1E-12 | | | | 1.06E-04 | | | | | | 48 | | |
| rs7827182 | | | 8 | | 8380471 | | | | | | SGK223 | | | G | | | | | | | | C | | | 0.511 | | | | | -0.011 | | | | | | 0.001 | | | | | | 2.9E-16 | | | | 1.48E-04 | | | | | | 67 | | |
| rs6601451 | | | 8 | | 10243681 | | | | | | MSRA | | | C | | | | | | | | G | | | 0.485 | | | | | 0.012 | | | | | | 0.001 | | | | | | 3.7E-17 | | | | 1.56E-04 | | | | | | 71 | | |
| rs55896564 | | | 8 | | 11447093 | | | | | | BLK | | | G | | | | | | | | A | | | 0.465 | | | | | 0.012 | | | | | | 0.001 | | | | | | 2.6E-17 | | | | 1.58E-04 | | | | | | 72 | | |
| rs6530737 | | | 8 | | 14095763 | | | | | | SGCZ | | | A | | | | | | | | G | | | 0.349 | | | | | 0.009 | | | | | | 0.001 | | | | | | 3.5E-10 | | | | 8.69E-05 | | | | | | 39 | | |
| rs2616143 | | | 8 | | 20632022 | | | | | | LZTS1 | | | G | | | | | | | | A | | | 0.680 | | | | | 0.009 | | | | | | 0.001 | | | | | | 1.9E-09 | | | | 7.95E-05 | | | | | | 36 | | |
| rs117176448 | | | 8 | | 27261138 | | | | | | PTK2B | | | C | | | | | | | | G | | | 0.904 | | | | | -0.015 | | | | | | 0.002 | | | | | | 1.2E-10 | | | | 9.16E-05 | | | | | | 42 | | |
| rs4266606 | | | 8 | | 28043673 | | | | | | ELP3 | | | C | | | | | | | | T | | | 0.143 | | | | | 0.011 | | | | | | 0.002 | | | | | | 4.1E-08 | | | | 6.64E-05 | | | | | | 30 | | |
| rs2725371 | | | 8 | | 30854033 | | | | | | PURG | | | A | | | | | | | | G | | | 0.304 | | | | | 0.010 | | | | | | 0.001 | | | | | | 2E-11 | | | | 9.92E-05 | | | | | | 45 | | |
| rs4739558 | | | 8 | | 38337264 | | | | | | FGFR1 | | | A | | | | | | | | G | | | 0.399 | | | | | 0.008 | | | | | | 0.001 | | | | | | 7.4E-09 | | | | 7.38E-05 | | | | | | 33 | | |
| rs35894137 | | | 8 | | 43071838 | | | | | | HGSNAT | | | C | | | | | | | | T | | | 0.919 | | | | | 0.014 | | | | | | 0.003 | | | | | | 1.6E-08 | | | | 7.03E-05 | | | | | | 32 | | |
| rs143662847 | | | 8 | | 48804722 | | | | | | PRKDC | | | C | | | | | | | | T | | | 0.960 | | | | | 0.019 | | | | | | 0.004 | | | | | | 4.6E-08 | | | | 6.59E-05 | | | | | | 30 | | |
| rs473837 | | | 8 | | 60906881 | | | | | | CA8 | | | G | | | | | | | | T | | | 0.648 | | | | | 0.009 | | | | | | 0.001 | | | | | | 1.8E-09 | | | | 7.97E-05 | | | | | | 36 | | |
| rs12681792 | | | 8 | | 62054463 | | | | | | CLVS1 | | | C | | | | | | | | A | | | 0.807 | | | | | -0.010 | | | | | | 0.002 | | | | | | 4.3E-08 | | | | 6.62E-05 | | | | | | 30 | | |
| rs4737188 | | | 8 | | 64756657 | | | | | | YTHDF3 | | | A | | | | | | | | T | | | 0.526 | | | | | 0.008 | | | | | | 0.001 | | | | | | 1.5E-09 | | | | 8.06E-05 | | | | | | 37 | | |
| rs35957544 | | | 8 | | 73440371 | | | | | | KCNB2 | | | G | | | | | | | | T | | | 0.426 | | | | | 0.013 | | | | | | 0.001 | | | | | | 7.4E-20 | | | | 1.84E-04 | | | | | | 83 | | |
| rs2941432 | | | 8 | | 76532219 | | | | | | HNF4G | | | T | | | | | | | | A | | | 0.528 | | | | | -0.008 | | | | | | 0.001 | | | | | | 1.3E-08 | | | | 7.13E-05 | | | | | | 32 | | |
| rs78565420 | | | 8 | | 85703065 | | | | | | RALYL | | | C | | | | | | | | T | | | 0.948 | | | | | -0.019 | | | | | | 0.003 | | | | | | 2.7E-09 | | | | 7.81E-05 | | | | | | 35 | | |
| rs17619860 | | | 8 | | 87779603 | | | | | | CNGB3 | | | T | | | | | | | | C | | | 0.837 | | | | | -0.010 | | | | | | 0.002 | | | | | | 2.2E-08 | | | | 6.90E-05 | | | | | | 31 | | |
| rs1905616 | | | 8 | | 93235675 | | | | | | RUNX1T1 | | | G | | | | | | | | A | | | 0.669 | | | | | 0.008 | | | | | | 0.001 | | | | | | 1.8E-08 | | | | 6.99E-05 | | | | | | 32 | | |
| rs2114210 | | | 8 | | 95595162 | | | | | | KIAA1429 | | | G | | | | | | | | A | | | 0.664 | | | | | -0.010 | | | | | | 0.001 | | | | | | 1.2E-11 | | | | 1.02E-04 | | | | | | 46 | | |
| rs17716502 | | | 8 | | 116659731 | | | | | | TRPS1 | | | C | | | | | | | | T | | | 0.796 | | | | | 0.016 | | | | | | 0.002 | | | | | | 2.7E-21 | | | | 1.98E-04 | | | | | | 90 | | |
| rs72673947 | | | 8 | | 118884379 | | | | | | EXT1 | | | A | | | | | | | | G | | | 0.893 | | | | | -0.014 | | | | | | 0.002 | | | | | | 3.3E-10 | | | | 8.72E-05 | | | | | | 40 | | |
| rs112875651 | | | 8 | | 126506694 | | | | | | TRIB1 | | | G | | | | | | | | A | | | 0.609 | | | | | -0.008 | | | | | | 0.001 | | | | | | 4.3E-09 | | | | 7.60E-05 | | | | | | 34 | | |
| rs11782074 | | | 8 | | 142617096 | | | | | | AC138647.1 | | | G | | | | | | | | T | | | 0.616 | | | | | -0.010 | | | | | | 0.001 | | | | | | 1.2E-11 | | | | 1.01E-04 | | | | | | 46 | | |
| rs1865341 | | | 9 | | 8845911 | | | | | | PTPRD | | | C | | | | | | | | T | | | 0.238 | | | | | -0.009 | | | | | | 0.002 | | | | | | 2.5E-08 | | | | 6.85E-05 | | | | | | 31 | | |
| rs10960276 | | | 9 | | 11819686 | | | | | | TYRP1 | | | C | | | | | | | | A | | | 0.645 | | | | | 0.008 | | | | | | 0.001 | | | | | | 1.6E-08 | | | | 7.05E-05 | | | | | | 32 | | |
| rs7020196 | | | 9 | | 12289527 | | | | | | TYRP1 | | | C | | | | | | | | T | | | 0.420 | | | | | 0.008 | | | | | | 0.001 | | | | | | 3.6E-08 | | | | 6.70E-05 | | | | | | 30 | | |
| rs13292699 | | | 9 | | 15910044 | | | | | | CCDC171 | | | A | | | | | | | | C | | | 0.566 | | | | | 0.013 | | | | | | 0.001 | | | | | | 1.5E-19 | | | | 1.80E-04 | | | | | | 82 | | |
| rs1411432 | | | 9 | | 16728532 | | | | | | BNC2 | | | A | | | | | | | | C | | | 0.814 | | | | | -0.012 | | | | | | 0.002 | | | | | | 2E-12 | | | | 1.09E-04 | | | | | | 49 | | |
| rs17770336 | | | 9 | | 28414625 | | | | | | LINGO2 | | | C | | | | | | | | T | | | 0.678 | | | | | -0.016 | | | | | | 0.001 | | | | | | 3.1E-26 | | | | 2.48E-04 | | | | | | 112 | | |
| rs10969334 | | | 9 | | 29717279 | | | | | | LINGO2 | | | C | | | | | | | | A | | | 0.606 | | | | | 0.009 | | | | | | 0.001 | | | | | | 6.9E-10 | | | | 8.40E-05 | | | | | | 38 | | |
| rs10973159 | | | 9 | | 36992547 | | | | | | PAX5 | | | G | | | | | | | | T | | | 0.380 | | | | | -0.008 | | | | | | 0.001 | | | | | | 1.2E-08 | | | | 7.16E-05 | | | | | | 32 | | |
| rs7038966 | | | 9 | | 73777777 | | | | | | TRPM3 | | | C | | | | | | | | T | | | 0.589 | | | | | -0.009 | | | | | | 0.001 | | | | | | 1.4E-11 | | | | 1.01E-04 | | | | | | 46 | | |
| rs1547205 | | | 9 | | 98815145 | | | | | | ERCC6L2 | | | G | | | | | | | | C | | | 0.900 | | | | | 0.013 | | | | | | 0.002 | | | | | | 1.3E-08 | | | | 7.14E-05 | | | | | | 32 | | |
| rs4989244 | | | 9 | | 102100348 | | | | | | SEC61B | | | G | | | | | | | | A | | | 0.568 | | | | | 0.008 | | | | | | 0.001 | | | | | | 3.6E-08 | | | | 6.70E-05 | | | | | | 30 | | |
| rs2135745 | | | 9 | | 109150784 | | | | | | ZNF462 | | | C | | | | | | | | G | | | 0.249 | | | | | 0.009 | | | | | | 0.002 | | | | | | 1.1E-08 | | | | 7.22E-05 | | | | | | 33 | | |
| rs2417998 | | | 9 | | 111958746 | | | | | | EPB41L4B | | | C | | | | | | | | G | | | 0.292 | | | | | 0.008 | | | | | | 0.002 | | | | | | 2.8E-08 | | | | 6.80E-05 | | | | | | 31 | | |
| rs12376870 | | | 9 | | 117890567 | | | | | | TNC | | | G | | | | | | | | A | | | 0.762 | | | | | 0.009 | | | | | | 0.002 | | | | | | 4.1E-08 | | | | 6.64E-05 | | | | | | 30 | | |
| rs7038943 | | | 9 | | 120377178 | | | | | | TLR4 | | | T | | | | | | | | C | | | 0.661 | | | | | 0.009 | | | | | | 0.001 | | | | | | 1.6E-10 | | | | 9.04E-05 | | | | | | 41 | | |
| rs6478538 | | | 9 | | 124627012 | | | | | | TTLL11 | | | A | | | | | | | | G | | | 0.323 | | | | | 0.009 | | | | | | 0.001 | | | | | | 1.9E-09 | | | | 7.96E-05 | | | | | | 36 | | |
| rs10760277 | | | 9 | | 126093999 | | | | | | CRB2 | | | C | | | | | | | | T | | | 0.615 | | | | | -0.009 | | | | | | 0.001 | | | | | | 3.6E-10 | | | | 8.68E-05 | | | | | | 39 | | |
| rs7030609 | | | 9 | | 129415775 | | | | | | LMX1B | | | A | | | | | | | | G | | | 0.910 | | | | | -0.015 | | | | | | 0.002 | | | | | | 3.5E-10 | | | | 8.69E-05 | | | | | | 39 | | |
| rs113132247 | | | 9 | | 131026108 | | | | | | GOLGA2 | | | G | | | | | | | | A | | | 0.847 | | | | | -0.012 | | | | | | 0.002 | | | | | | 1.1E-10 | | | | 9.18E-05 | | | | | | 42 | | |
| rs7913496 | | | 10 | | 10257277 | | | | | | CELF2 | | | C | | | | | | | | T | | | 0.180 | | | | | 0.010 | | | | | | 0.002 | | | | | | 2E-08 | | | | 6.96E-05 | | | | | | 32 | | |
| rs7893571 | | | 10 | | 16750129 | | | | | | RSU1 | | | G | | | | | | | | T | | | 0.334 | | | | | -0.010 | | | | | | 0.001 | | | | | | 2.9E-12 | | | | 1.08E-04 | | | | | | 49 | | |
| rs12253527 | | | 10 | | 21819824 | | | | | | MLLT10 | | | G | | | | | | | | A | | | 0.678 | | | | | -0.014 | | | | | | 0.001 | | | | | | 2E-20 | | | | 1.89E-04 | | | | | | 86 | | |
| rs71495049 | | | 10 | | 34014435 | | | | | | PARD3 | | | G | | | | | | | | A | | | 0.916 | | | | | -0.017 | | | | | | 0.002 | | | | | | 5.7E-12 | | | | 1.05E-04 | | | | | | 47 | | |
| rs3125326 | | | 10 | | 63053788 | | | | | | TMEM26 | | | A | | | | | | | | C | | | 0.392 | | | | | -0.008 | | | | | | 0.001 | | | | | | 2.7E-08 | | | | 6.82E-05 | | | | | | 31 | | |
| rs7924036 | | | 10 | | 65191645 | | | | | | JMJD1C | | | G | | | | | | | | T | | | 0.497 | | | | | 0.010 | | | | | | 0.001 | | | | | | 2.2E-12 | | | | 1.09E-04 | | | | | | 49 | | |
| rs11000993 | | | 10 | | 76084111 | | | | | | ADK | | | T | | | | | | | | C | | | 0.876 | | | | | -0.014 | | | | | | 0.002 | | | | | | 3.1E-11 | | | | 9.74E-05 | | | | | | 44 | | |
| rs1250597 | | | 10 | | 81010250 | | | | | | ZMIZ1 | | | A | | | | | | | | G | | | 0.405 | | | | | -0.009 | | | | | | 0.001 | | | | | | 1E-09 | | | | 8.22E-05 | | | | | | 37 | | |
| rs17399739 | | | 10 | | 87490850 | | | | | | GRID1 | | | A | | | | | | | | G | | | 0.931 | | | | | -0.018 | | | | | | 0.003 | | | | | | 5.9E-11 | | | | 9.45E-05 | | | | | | 43 | | |
| rs2450444 | | | 10 | | 93010383 | | | | | | PCGF5 | | | G | | | | | | | | A | | | 0.651 | | | | | 0.008 | | | | | | 0.001 | | | | | | 2E-08 | | | | 6.96E-05 | | | | | | 32 | | |
| rs41310284 | | | 10 | | 102447647 | | | | | | PAX2 | | | C | | | | | | | | A | | | 0.899 | | | | | 0.018 | | | | | | 0.002 | | | | | | 9.8E-15 | | | | 1.32E-04 | | | | | | 60 | | |
| rs10736156 | | | 10 | | 104019447 | | | | | | GBF1 | | | C | | | | | | | | A | | | 0.156 | | | | | -0.012 | | | | | | 0.002 | | | | | | 3.8E-10 | | | | 8.65E-05 | | | | | | 39 | | |
| rs7086898 | | | 10 | | 104386152 | | | | | | SUFU | | | A | | | | | | | | G | | | 0.920 | | | | | -0.014 | | | | | | 0.003 | | | | | | 4.3E-08 | | | | 6.62E-05 | | | | | | 30 | | |
| rs4575195 | | | 10 | | 114765747 | | | | | | TCF7L2 | | | C | | | | | | | | A | | | 0.687 | | | | | 0.010 | | | | | | 0.001 | | | | | | 9E-11 | | | | 9.28E-05 | | | | | | 42 | | |
| rs9421249 | | | 10 | | 118623322 | | | | | | ENO4 | | | C | | | | | | | | T | | | 0.739 | | | | | -0.010 | | | | | | 0.002 | | | | | | 2.5E-11 | | | | 9.83E-05 | | | | | | 45 | | |
| rs845084 | | | 10 | | 125220036 | | | | | | GPR26 | | | G | | | | | | | | A | | | 0.742 | | | | | -0.010 | | | | | | 0.002 | | | | | | 3.7E-10 | | | | 8.66E-05 | | | | | | 39 | | |
| rs4962725 | | | 10 | | 126733321 | | | | | | CTBP2 | | | T | | | | | | | | C | | | 0.572 | | | | | -0.010 | | | | | | 0.001 | | | | | | 6.6E-13 | | | | 1.14E-04 | | | | | | 52 | | |
| rs2542615 | | | 10 | | 131128952 | | | | | | MGMT | | | C | | | | | | | | T | | | 0.342 | | | | | 0.008 | | | | | | 0.001 | | | | | | 7.3E-09 | | | | 7.38E-05 | | | | | | 33 | | |
| rs2035806 | | | 10 | | 133984916 | | | | | | JAKMIP3 | | | G | | | | | | | | A | | | 0.435 | | | | | 0.010 | | | | | | 0.001 | | | | | | 5.7E-12 | | | | 1.05E-04 | | | | | | 47 | | |
| rs67257872 | | | 11 | | 8530218 | | | | | | STK33 | | | A | | | | | | | | G | | | 0.553 | | | | | 0.011 | | | | | | 0.001 | | | | | | 1.5E-14 | | | | 1.30E-04 | | | | | | 59 | | |
| rs28711392 | | | 11 | | 13349559 | | | | | | ARNTL | | | T | | | | | | | | C | | | 0.633 | | | | | 0.012 | | | | | | 0.001 | | | | | | 2.1E-16 | | | | 1.49E-04 | | | | | | 67 | | |
| rs6265 | | | 11 | | 27679916 | | | | | | BDNF | | | C | | | | | | | | T | | | 0.812 | | | | | 0.025 | | | | | | 0.002 | | | | | | 1.5E-44 | | | | 4.32E-04 | | | | | | 196 | | |
| rs10835498 | | | 11 | | 29258947 | | | | | | KCNA4 | | | G | | | | | | | | A | | | 0.569 | | | | | -0.008 | | | | | | 0.001 | | | | | | 8.9E-09 | | | | 7.29E-05 | | | | | | 33 | | |
| rs1222216 | | | 11 | | 30346052 | | | | | | ARL14EP | | | C | | | | | | | | T | | | 0.774 | | | | | 0.012 | | | | | | 0.002 | | | | | | 1.1E-13 | | | | 1.22E-04 | | | | | | 55 | | |
| rs59227842 | | | 11 | | 43692423 | | | | | | HSD17B12 | | | A | | | | | | | | G | | | 0.688 | | | | | -0.015 | | | | | | 0.001 | | | | | | 1.8E-24 | | | | 2.30E-04 | | | | | | 104 | | |
| rs868784 | | | 11 | | 43944388 | | | | | | C11orf96 | | | G | | | | | | | | A | | | 0.619 | | | | | 0.008 | | | | | | 0.001 | | | | | | 2.7E-08 | | | | 6.81E-05 | | | | | | 31 | | |
| rs6416134 | | | 11 | | 45352781 | | | | | | SYT13 | | | G | | | | | | | | C | | | 0.181 | | | | | -0.010 | | | | | | 0.002 | | | | | | 4.6E-08 | | | | 6.59E-05 | | | | | | 30 | | |
| rs12798028 | | | 11 | | 47604639 | | | | | | NDUFS3 | | | C | | | | | | | | T | | | 0.591 | | | | | -0.015 | | | | | | 0.001 | | | | | | 3.4E-27 | | | | 2.57E-04 | | | | | | 117 | | |
| rs12363672 | | | 11 | | 55684028 | | | | | | OR5W2 | | | A | | | | | | | | C | | | 0.974 | | | | | -0.024 | | | | | | 0.004 | | | | | | 2.8E-08 | | | | 6.81E-05 | | | | | | 31 | | |
| rs34292685 | | | 11 | | 64049021 | | | | | | GPR137 | | | C | | | | | | | | T | | | 0.838 | | | | | 0.013 | | | | | | 0.002 | | | | | | 7.9E-12 | | | | 1.03E-04 | | | | | | 47 | | |
| rs2234458 | | | 11 | | 65639374 | | | | | | EFEMP2 | | | C | | | | | | | | T | | | 0.360 | | | | | 0.013 | | | | | | 0.001 | | | | | | 2.5E-19 | | | | 1.78E-04 | | | | | | 81 | | |
| rs667515 | | | 11 | | 69449076 | | | | | | CCND1 | | | G | | | | | | | | C | | | 0.614 | | | | | 0.009 | | | | | | 0.001 | | | | | | 8.4E-10 | | | | 8.31E-05 | | | | | | 38 | | |
| rs10160769 | | | 11 | | 76474827 | | | | | | TSKU | | | G | | | | | | | | C | | | 0.783 | | | | | 0.009 | | | | | | 0.002 | | | | | | 4.6E-08 | | | | 6.59E-05 | | | | | | 30 | | |
| rs7102934 | | | 11 | | 84648068 | | | | | | DLG2 | | | T | | | | | | | | C | | | 0.688 | | | | | -0.009 | | | | | | 0.001 | | | | | | 1.2E-09 | | | | 8.15E-05 | | | | | | 37 | | |
| rs61903695 | | | 11 | | 89922417 | | | | | | NAALAD2 | | | A | | | | | | | | G | | | 0.745 | | | | | -0.011 | | | | | | 0.002 | | | | | | 6.6E-13 | | | | 1.14E-04 | | | | | | 52 | | |
| rs2658797 | | | 11 | | 93212254 | | | | | | SMCO4 | | | C | | | | | | | | T | | | 0.518 | | | | | 0.008 | | | | | | 0.001 | | | | | | 1.3E-08 | | | | 7.12E-05 | | | | | | 32 | | |
| rs680071 | | | 11 | | 103088414 | | | | | | DYNC2H1 | | | T | | | | | | | | C | | | 0.119 | | | | | -0.012 | | | | | | 0.002 | | | | | | 2.8E-08 | | | | 6.80E-05 | | | | | | 31 | | |
| rs719802 | | | 11 | | 113234679 | | | | | | TTC12 | | | T | | | | | | | | C | | | 0.386 | | | | | 0.008 | | | | | | 0.001 | | | | | | 5.7E-09 | | | | 7.49E-05 | | | | | | 34 | | |
| rs11607476 | | | 11 | | 115037061 | | | | | | CADM1 | | | A | | | | | | | | C | | | 0.513 | | | | | -0.010 | | | | | | 0.001 | | | | | | 9.2E-13 | | | | 1.13E-04 | | | | | | 51 | | |
| rs7928320 | | | 11 | | 116942753 | | | | | | SIK3 | | | C | | | | | | | | G | | | 0.943 | | | | | -0.017 | | | | | | 0.003 | | | | | | 6.1E-09 | | | | 7.46E-05 | | | | | | 34 | | |
| rs12281009 | | | 11 | | 117032959 | | | | | | PAFAH1B2 | | | A | | | | | | | | G | | | 0.942 | | | | | -0.018 | | | | | | 0.003 | | | | | | 1.3E-09 | | | | 8.11E-05 | | | | | | 37 | | |
| rs7925100 | | | 11 | | 118941596 | | | | | | VPS11 | | | G | | | | | | | | A | | | 0.604 | | | | | -0.009 | | | | | | 0.001 | | | | | | 6.3E-11 | | | | 9.43E-05 | | | | | | 43 | | |
| rs11218510 | | | 11 | | 121922587 | | | | | | BLID | | | G | | | | | | | | A | | | 0.600 | | | | | 0.008 | | | | | | 0.001 | | | | | | 7.8E-09 | | | | 7.36E-05 | | | | | | 33 | | |
| rs10791113 | | | 11 | | 130873165 | | | | | | SNX19 | | | A | | | | | | | | G | | | 0.494 | | | | | -0.009 | | | | | | 0.001 | | | | | | 4.6E-10 | | | | 8.57E-05 | | | | | | 39 | | |
| rs12788343 | | | 11 | | 131452912 | | | | | | NTM | | | T | | | | | | | | C | | | 0.591 | | | | | -0.010 | | | | | | 0.001 | | | | | | 4.3E-12 | | | | 1.06E-04 | | | | | | 48 | | |
| rs11223204 | | | 11 | | 132652554 | | | | | | OPCML | | | A | | | | | | | | G | | | 0.566 | | | | | -0.009 | | | | | | 0.001 | | | | | | 3E-10 | | | | 8.76E-05 | | | | | | 40 | | |
| rs329651 | | | 11 | | 133767622 | | | | | | IGSF9B | | | G | | | | | | | | T | | | 0.196 | | | | | -0.011 | | | | | | 0.002 | | | | | | 1.7E-10 | | | | 9.01E-05 | | | | | | 41 | | |
| rs61909165 | | | 11 | | 134589355 | | | | | | AP003062.1 | | | T | | | | | | | | A | | | 0.826 | | | | | -0.012 | | | | | | 0.002 | | | | | | 1.6E-11 | | | | 1.00E-04 | | | | | | 45 | | |
| rs55726687 | | | 12 | | 991306 | | | | | | WNK1 | | | G | | | | | | | | A | | | 0.790 | | | | | -0.014 | | | | | | 0.002 | | | | | | 3E-16 | | | | 1.47E-04 | | | | | | 67 | | |
| rs10774018 | | | 12 | | 2157925 | | | | | | CACNA1C | | | G | | | | | | | | C | | | 0.780 | | | | | -0.009 | | | | | | 0.002 | | | | | | 1.1E-08 | | | | 7.19E-05 | | | | | | 33 | | |
| rs1799507 | | | 12 | | 16427314 | | | | | | SLC15A5 | | | G | | | | | | | | A | | | 0.856 | | | | | -0.011 | | | | | | 0.002 | | | | | | 3.1E-08 | | | | 6.77E-05 | | | | | | 31 | | |
| rs10505836 | | | 12 | | 19288508 | | | | | | PLEKHA5 | | | A | | | | | | | | C | | | 0.140 | | | | | -0.012 | | | | | | 0.002 | | | | | | 1.1E-09 | | | | 8.19E-05 | | | | | | 37 | | |
| rs10842231 | | | 12 | | 23986029 | | | | | | SOX5 | | | A | | | | | | | | T | | | 0.917 | | | | | -0.014 | | | | | | 0.003 | | | | | | 8.4E-09 | | | | 7.32E-05 | | | | | | 33 | | |
| rs1458156 | | | 12 | | 41887940 | | | | | | PDZRN4 | | | C | | | | | | | | T | | | 0.512 | | | | | -0.009 | | | | | | 0.001 | | | | | | 1.3E-11 | | | | 1.01E-04 | | | | | | 46 | | |
| rs1126930 | | | 12 | | 49399132 | | | | | | PRKAG1 | | | G | | | | | | | | C | | | 0.965 | | | | | -0.021 | | | | | | 0.004 | | | | | | 8.6E-09 | | | | 7.31E-05 | | | | | | 33 | | |
| rs7132908 | | | 12 | | 50263148 | | | | | | FAIM2 | | | G | | | | | | | | A | | | 0.616 | | | | | -0.019 | | | | | | 0.001 | | | | | | 8.4E-40 | | | | 3.85E-04 | | | | | | 174 | | |
| rs4077093 | | | 12 | | 51593616 | | | | | | POU6F1 | | | T | | | | | | | | G | | | 0.216 | | | | | 0.010 | | | | | | 0.002 | | | | | | 7.9E-09 | | | | 7.35E-05 | | | | | | 33 | | |
| rs4759073 | | | 12 | | 54653258 | | | | | | CBX5 | | | G | | | | | | | | A | | | 0.590 | | | | | 0.009 | | | | | | 0.001 | | | | | | 1.9E-11 | | | | 9.95E-05 | | | | | | 45 | | |
| rs4759228 | | | 12 | | 56508409 | | | | | | PA2G4 | | | G | | | | | | | | C | | | 0.705 | | | | | 0.012 | | | | | | 0.002 | | | | | | 1.3E-14 | | | | 1.31E-04 | | | | | | 59 | | |
| rs12821416 | | | 12 | | 58580570 | | | | | | XRCC6BP1 | | | C | | | | | | | | T | | | 0.864 | | | | | -0.011 | | | | | | 0.002 | | | | | | 1.2E-08 | | | | 7.16E-05 | | | | | | 32 | | |
| rs61754230 | | | 12 | | 72179446 | | | | | | RAB21 | | | C | | | | | | | | T | | | 0.980 | | | | | -0.027 | | | | | | 0.005 | | | | | | 2.8E-08 | | | | 6.81E-05 | | | | | | 31 | | |
| rs12427047 | | | 12 | | 90213070 | | | | | | ATP2B1 | | | C | | | | | | | | T | | | 0.757 | | | | | 0.011 | | | | | | 0.002 | | | | | | 8.9E-12 | | | | 1.03E-04 | | | | | | 47 | | |
| rs2712667 | | | 12 | | 99588917 | | | | | | ANKS1B | | | G | | | | | | | | C | | | 0.356 | | | | | 0.010 | | | | | | 0.001 | | | | | | 2.1E-11 | | | | 9.89E-05 | | | | | | 45 | | |
| rs4764949 | | | 12 | | 103658096 | | | | | | C12orf42 | | | A | | | | | | | | G | | | 0.674 | | | | | 0.012 | | | | | | 0.001 | | | | | | 3.8E-16 | | | | 1.46E-04 | | | | | | 66 | | |
| rs6606686 | | | 12 | | 110903380 | | | | | | GPN3 | | | G | | | | | | | | C | | | 0.319 | | | | | 0.010 | | | | | | 0.001 | | | | | | 3.4E-11 | | | | 9.69E-05 | | | | | | 44 | | |
| rs11513729 | | | 12 | | 112273499 | | | | | | MAPKAPK5 | | | C | | | | | | | | T | | | 0.587 | | | | | 0.008 | | | | | | 0.001 | | | | | | 1.5E-08 | | | | 7.08E-05 | | | | | | 32 | | |
| rs181617194 | | | 12 | | 122011598 | | | | | | KDM2B | | | T | | | | | | | | C | | | 0.959 | | | | | 0.023 | | | | | | 0.004 | | | | | | 1.7E-09 | | | | 8.00E-05 | | | | | | 36 | | |
| rs147730268 | | | 12 | | 123024476 | | | | | | KNTC1 | | | G | | | | | | | | T | | | 0.913 | | | | | 0.023 | | | | | | 0.002 | | | | | | 4E-20 | | | | 1.86E-04 | | | | | | 84 | | |
| rs9579775 | | | 13 | | 20616557 | | | | | | ZMYM2 | | | A | | | | | | | | C | | | 0.864 | | | | | -0.015 | | | | | | 0.002 | | | | | | 2.1E-12 | | | | 1.09E-04 | | | | | | 49 | | |
| rs9507791 | | | 13 | | 27352678 | | | | | | GPR12 | | | G | | | | | | | | A | | | 0.213 | | | | | 0.010 | | | | | | 0.002 | | | | | | 4.3E-09 | | | | 7.61E-05 | | | | | | 34 | | |
| rs1967772 | | | 13 | | 28036062 | | | | | | MTIF3 | | | G | | | | | | | | A | | | 0.715 | | | | | 0.011 | | | | | | 0.002 | | | | | | 8.2E-13 | | | | 1.13E-04 | | | | | | 51 | | |
| rs35193668 | | | 13 | | 33092929 | | | | | | N4BP2L2 | | | C | | | | | | | | T | | | 0.639 | | | | | 0.010 | | | | | | 0.001 | | | | | | 2.5E-13 | | | | 1.18E-04 | | | | | | 54 | | |
| rs61954177 | | | 13 | | 40787036 | | | | | | AL133318.1 | | | G | | | | | | | | C | | | 0.673 | | | | | -0.009 | | | | | | 0.001 | | | | | | 2.7E-09 | | | | 7.81E-05 | | | | | | 35 | | |
| rs12429545 | | | 13 | | 54102206 | | | | | | OLFM4 | | | G | | | | | | | | A | | | 0.871 | | | | | -0.020 | | | | | | 0.002 | | | | | | 2.6E-22 | | | | 2.08E-04 | | | | | | 94 | | |
| rs7321285 | | | 13 | | 54319327 | | | | | | OLFM4 | | | A | | | | | | | | C | | | 0.201 | | | | | 0.011 | | | | | | 0.002 | | | | | | 4.1E-11 | | | | 9.61E-05 | | | | | | 44 | | |
| rs2576135 | | | 13 | | 54691442 | | | | | | OLFM4 | | | T | | | | | | | | A | | | 0.093 | | | | | 0.013 | | | | | | 0.002 | | | | | | 3.5E-08 | | | | 6.71E-05 | | | | | | 30 | | |
| rs9317002 | | | 13 | | 59175727 | | | | | | PCDH17 | | | C | | | | | | | | A | | | 0.485 | | | | | -0.011 | | | | | | 0.001 | | | | | | 1.9E-16 | | | | 1.49E-04 | | | | | | 68 | | |
| rs9529148 | | | 13 | | 67419495 | | | | | | PCDH9 | | | G | | | | | | | | A | | | 0.377 | | | | | -0.008 | | | | | | 0.001 | | | | | | 1.9E-08 | | | | 6.97E-05 | | | | | | 32 | | |
| rs1576655 | | | 13 | | 79587841 | | | | | | RBM26 | | | A | | | | | | | | C | | | 0.404 | | | | | -0.011 | | | | | | 0.001 | | | | | | 1.9E-15 | | | | 1.39E-04 | | | | | | 63 | | |
| rs61971082 | | | 13 | | 86494667 | | | | | | SLITRK6 | | | T | | | | | | | | G | | | 0.716 | | | | | -0.010 | | | | | | 0.002 | | | | | | 3E-11 | | | | 9.75E-05 | | | | | | 44 | | |
| rs7331420 | | | 13 | | 99236471 | | | | | | STK24 | | | G | | | | | | | | A | | | 0.715 | | | | | 0.009 | | | | | | 0.002 | | | | | | 2.1E-09 | | | | 7.92E-05 | | | | | | 36 | | |
| rs9888533 | | | 13 | | 107854612 | | | | | | FAM155A | | | C | | | | | | | | T | | | 0.462 | | | | | -0.008 | | | | | | 0.001 | | | | | | 4.5E-08 | | | | 6.60E-05 | | | | | | 30 | | |
| rs9522180 | | | 13 | | 111970212 | | | | | | TEX29 | | | C | | | | | | | | T | | | 0.447 | | | | | 0.009 | | | | | | 0.001 | | | | | | 2.4E-11 | | | | 9.85E-05 | | | | | | 45 | | |
| rs9515446 | | | 13 | | 112217108 | | | | | | RP11-65D24.2 | | | A | | | | | | | | G | | | 0.552 | | | | | -0.010 | | | | | | 0.001 | | | | | | 2.2E-12 | | | | 1.09E-04 | | | | | | 49 | | |
| rs8015400 | | | 14 | | 25930988 | | | | | | STXBP6 | | | C | | | | | | | | A | | | 0.323 | | | | | -0.013 | | | | | | 0.001 | | | | | | 6.9E-18 | | | | 1.64E-04 | | | | | | 74 | | |
| rs9788550 | | | 14 | | 29681138 | | | | | | PRKD1 | | | G | | | | | | | | C | | | 0.753 | | | | | 0.014 | | | | | | 0.002 | | | | | | 5.6E-18 | | | | 1.65E-04 | | | | | | 75 | | |
| rs12883788 | | | 14 | | 33303540 | | | | | | AKAP6 | | | C | | | | | | | | T | | | 0.540 | | | | | -0.012 | | | | | | 0.001 | | | | | | 2.3E-19 | | | | 1.79E-04 | | | | | | 81 | | |
| rs7141912 | | | 14 | | 35649431 | | | | | | KIAA0391 | | | A | | | | | | | | T | | | 0.874 | | | | | 0.012 | | | | | | 0.002 | | | | | | 1.1E-08 | | | | 7.21E-05 | | | | | | 33 | | |
| rs8011566 | | | 14 | | 42939471 | | | | | | LRFN5 | | | T | | | | | | | | A | | | 0.574 | | | | | -0.009 | | | | | | 0.001 | | | | | | 3.5E-10 | | | | 8.69E-05 | | | | | | 39 | | |
| rs724623 | | | 14 | | 47303577 | | | | | | MDGA2 | | | A | | | | | | | | C | | | 0.492 | | | | | 0.010 | | | | | | 0.001 | | | | | | 9E-14 | | | | 1.23E-04 | | | | | | 56 | | |
| rs217672 | | | 14 | | 62361021 | | | | | | SYT16 | | | A | | | | | | | | C | | | 0.728 | | | | | -0.012 | | | | | | 0.002 | | | | | | 2.3E-14 | | | | 1.29E-04 | | | | | | 58 | | |
| rs3902951 | | | 14 | | 69789755 | | | | | | GALNT16 | | | T | | | | | | | | G | | | 0.763 | | | | | -0.011 | | | | | | 0.002 | | | | | | 1.1E-10 | | | | 9.18E-05 | | | | | | 42 | | |
| rs61986330 | | | 14 | | 73314450 | | | | | | DPF3 | | | C | | | | | | | | A | | | 0.726 | | | | | 0.009 | | | | | | 0.002 | | | | | | 1.4E-08 | | | | 7.10E-05 | | | | | | 32 | | |
| rs10146997 | | | 14 | | 79945162 | | | | | | NRXN3 | | | A | | | | | | | | G | | | 0.778 | | | | | -0.016 | | | | | | 0.002 | | | | | | 3.2E-22 | | | | 2.07E-04 | | | | | | 94 | | |
| rs8008772 | | | 14 | | 88321884 | | | | | | GALC | | | A | | | | | | | | T | | | 0.749 | | | | | -0.009 | | | | | | 0.002 | | | | | | 5.7E-09 | | | | 7.49E-05 | | | | | | 34 | | |
| rs1286138 | | | 14 | | 91485445 | | | | | | RPS6KA5 | | | T | | | | | | | | G | | | 0.325 | | | | | -0.009 | | | | | | 0.001 | | | | | | 8.4E-10 | | | | 8.31E-05 | | | | | | 38 | | |
| rs6575340 | | | 14 | | 94023972 | | | | | | UNC79 | | | G | | | | | | | | A | | | 0.364 | | | | | -0.013 | | | | | | 0.001 | | | | | | 1.2E-20 | | | | 1.92E-04 | | | | | | 87 | | |
| rs12885251 | | | 14 | | 99670791 | | | | | | BCL11B | | | G | | | | | | | | A | | | 0.542 | | | | | 0.008 | | | | | | 0.001 | | | | | | 4.1E-08 | | | | 6.64E-05 | | | | | | 30 | | |
| rs12147845 | | | 14 | | 101144596 | | | | | | DLK1 | | | C | | | | | | | | T | | | 0.884 | | | | | -0.014 | | | | | | 0.002 | | | | | | 2.6E-10 | | | | 8.81E-05 | | | | | | 40 | | |
| rs61992671 | | | 14 | | 101531854 | | | | | | AL117190.3 | | | A | | | | | | | | G | | | 0.508 | | | | | 0.010 | | | | | | 0.001 | | | | | | 5.7E-12 | | | | 1.05E-04 | | | | | | 47 | | |
| rs7145882 | | | 14 | | 103255461 | | | | | | TRAF3 | | | T | | | | | | | | C | | | 0.342 | | | | | 0.011 | | | | | | 0.001 | | | | | | 2.2E-15 | | | | 1.39E-04 | | | | | | 63 | | |
| rs3759584 | | | 14 | | 103990799 | | | | | | CKB | | | T | | | | | | | | C | | | 0.637 | | | | | 0.010 | | | | | | 0.001 | | | | | | 1.1E-11 | | | | 1.02E-04 | | | | | | 46 | | |
| rs76520838 | | | 15 | | 47916618 | | | | | | SEMA6D | | | C | | | | | | | | T | | | 0.967 | | | | | -0.023 | | | | | | 0.004 | | | | | | 1.9E-09 | | | | 7.95E-05 | | | | | | 36 | | |
| rs7182917 | | | 15 | | 52080803 | | | | | | TMOD2 | | | T | | | | | | | | C | | | 0.550 | | | | | 0.009 | | | | | | 0.001 | | | | | | 2.8E-10 | | | | 8.78E-05 | | | | | | 40 | | |
| rs2247401 | | | 15 | | 53156672 | | | | | | ONECUT1 | | | G | | | | | | | | A | | | 0.733 | | | | | -0.009 | | | | | | 0.002 | | | | | | 4.7E-08 | | | | 6.58E-05 | | | | | | 30 | | |
| rs28465175 | | | 15 | | 53427155 | | | | | | ONECUT1 | | | A | | | | | | | | G | | | 0.930 | | | | | 0.015 | | | | | | 0.003 | | | | | | 2.6E-08 | | | | 6.84E-05 | | | | | | 31 | | |
| rs7175642 | | | 15 | | 59450079 | | | | | | MYO1E | | | T | | | | | | | | G | | | 0.342 | | | | | 0.009 | | | | | | 0.001 | | | | | | 5.2E-09 | | | | 7.52E-05 | | | | | | 34 | | |
| rs28408562 | | | 15 | | 60917079 | | | | | | RORA | | | C | | | | | | | | G | | | 0.553 | | | | | -0.008 | | | | | | 0.001 | | | | | | 1.3E-08 | | | | 7.13E-05 | | | | | | 32 | | |
| rs1369159 | | | 15 | | 66360842 | | | | | | MEGF11 | | | C | | | | | | | | T | | | 0.420 | | | | | 0.008 | | | | | | 0.001 | | | | | | 1.7E-08 | | | | 7.02E-05 | | | | | | 32 | | |
| rs111584879 | | | 15 | | 66678173 | | | | | | TIPIN | | | T | | | | | | | | C | | | 0.761 | | | | | 0.009 | | | | | | 0.002 | | | | | | 4.8E-08 | | | | 6.57E-05 | | | | | | 30 | | |
| rs2241420 | | | 15 | | 68082816 | | | | | | MAP2K5 | | | G | | | | | | | | A | | | 0.774 | | | | | 0.018 | | | | | | 0.002 | | | | | | 1.9E-29 | | | | 2.80E-04 | | | | | | 127 | | |
| rs62004865 | | | 15 | | 74207695 | | | | | | LOXL1 | | | T | | | | | | | | A | | | 0.894 | | | | | -0.014 | | | | | | 0.002 | | | | | | 1.4E-09 | | | | 8.08E-05 | | | | | | 37 | | |
| rs11856579 | | | 15 | | 78012688 | | | | | | LINGO1 | | | G | | | | | | | | A | | | 0.732 | | | | | 0.009 | | | | | | 0.002 | | | | | | 3.4E-09 | | | | 7.71E-05 | | | | | | 35 | | |
| rs57488047 | | | 15 | | 79403002 | | | | | | RASGRF1 | | | T | | | | | | | | C | | | 0.532 | | | | | 0.010 | | | | | | 0.001 | | | | | | 1.1E-12 | | | | 1.12E-04 | | | | | | 51 | | |
| rs34994596 | | | 15 | | 80991447 | | | | | | ABHD17C | | | T | | | | | | | | C | | | 0.703 | | | | | 0.011 | | | | | | 0.002 | | | | | | 1.1E-13 | | | | 1.22E-04 | | | | | | 55 | | |
| rs7498044 | | | 15 | | 92573639 | | | | | | SLCO3A1 | | | G | | | | | | | | A | | | 0.783 | | | | | 0.010 | | | | | | 0.002 | | | | | | 3.2E-09 | | | | 7.73E-05 | | | | | | 35 | | |
| rs8038574 | | | 15 | | 95275890 | | | | | | MCTP2 | | | T | | | | | | | | C | | | 0.346 | | | | | 0.009 | | | | | | 0.001 | | | | | | 4E-10 | | | | 8.63E-05 | | | | | | 39 | | |
| rs56803094 | | | 15 | | 99222509 | | | | | | IGF1R | | | A | | | | | | | | G | | | 0.773 | | | | | 0.010 | | | | | | 0.002 | | | | | | 5.8E-09 | | | | 7.48E-05 | | | | | | 34 | | |
| rs412243 | | | 16 | | 339672 | | | | | | AXIN1 | | | T | | | | | | | | C | | | 0.621 | | | | | 0.010 | | | | | | 0.001 | | | | | | 1.4E-13 | | | | 1.21E-04 | | | | | | 55 | | |
| rs2516726 | | | 16 | | 2095065 | | | | | | NTHL1 | | | T | | | | | | | | C | | | 0.772 | | | | | 0.010 | | | | | | 0.002 | | | | | | 1.7E-09 | | | | 8.01E-05 | | | | | | 36 | | |
| rs879620 | | | 16 | | 4015729 | | | | | | ADCY9 | | | C | | | | | | | | T | | | 0.387 | | | | | -0.014 | | | | | | 0.001 | | | | | | 5.9E-24 | | | | 2.25E-04 | | | | | | 102 | | |
| rs2660241 | | | 16 | | 4940023 | | | | | | PPL | | | T | | | | | | | | C | | | 0.635 | | | | | -0.008 | | | | | | 0.001 | | | | | | 1.2E-08 | | | | 7.16E-05 | | | | | | 32 | | |
| rs11642387 | | | 16 | | 6753239 | | | | | | RBFOX1 | | | A | | | | | | | | G | | | 0.899 | | | | | 0.013 | | | | | | 0.002 | | | | | | 2.1E-08 | | | | 6.94E-05 | | | | | | 31 | | |
| rs39674 | | | 16 | | 9413210 | | | | | | C16orf72 | | | C | | | | | | | | G | | | 0.304 | | | | | -0.009 | | | | | | 0.001 | | | | | | 6E-09 | | | | 7.47E-05 | | | | | | 34 | | |
| rs12927792 | | | 16 | | 9713194 | | | | | | RP11-297M9.1 | | | C | | | | | | | | T | | | 0.574 | | | | | -0.009 | | | | | | 0.001 | | | | | | 5.2E-10 | | | | 8.52E-05 | | | | | | 39 | | |
| rs8054082 | | | 16 | | 19975418 | | | | | | GPR139 | | | C | | | | | | | | T | | | 0.859 | | | | | 0.017 | | | | | | 0.002 | | | | | | 2.9E-18 | | | | 1.68E-04 | | | | | | 76 | | |
| rs4432271 | | | 16 | | 20245283 | | | | | | GP2 | | | C | | | | | | | | T | | | 0.131 | | | | | -0.012 | | | | | | 0.002 | | | | | | 2.1E-08 | | | | 6.92E-05 | | | | | | 31 | | |
| rs11864909 | | | 16 | | 20400839 | | | | | | PDILT | | | C | | | | | | | | T | | | 0.714 | | | | | -0.009 | | | | | | 0.002 | | | | | | 5.1E-09 | | | | 7.54E-05 | | | | | | 34 | | |
| rs9922288 | | | 16 | | 24550930 | | | | | | RBBP6 | | | A | | | | | | | | G | | | 0.236 | | | | | 0.009 | | | | | | 0.002 | | | | | | 9.8E-09 | | | | 7.25E-05 | | | | | | 33 | | |
| rs7498665 | | | 16 | | 28883241 | | | | | | SH2B1 | | | A | | | | | | | | G | | | 0.600 | | | | | -0.017 | | | | | | 0.001 | | | | | | 5.9E-35 | | | | 3.36E-04 | | | | | | 152 | | |
| rs3814883 | | | 16 | | 29994922 | | | | | | TAOK2 | | | C | | | | | | | | T | | | 0.518 | | | | | -0.015 | | | | | | 0.001 | | | | | | 8.1E-27 | | | | 2.54E-04 | | | | | | 115 | | |
| rs34898535 | | | 16 | | 31025641 | | | | | | STX1B | | | C | | | | | | | | T | | | 0.622 | | | | | 0.014 | | | | | | 0.001 | | | | | | 4.8E-24 | | | | 2.26E-04 | | | | | | 102 | | |
| rs56094641 | | | 16 | | 53806453 | | | | | | FTO | | | A | | | | | | | | G | | | 0.595 | | | | | -0.047 | | | | | | 0.001 | | | | | | 4E-243 | | | | 2.44E-03 | | | | | | 1109 | | |
| rs862320 | | | 16 | | 69651866 | | | | | | NFAT5 | | | C | | | | | | | | T | | | 0.590 | | | | | 0.014 | | | | | | 0.001 | | | | | | 6.2E-25 | | | | 2.35E-04 | | | | | | 106 | | |
| rs12149660 | | | 16 | | 70309237 | | | | | | AARS | | | G | | | | | | | | A | | | 0.885 | | | | | 0.016 | | | | | | 0.002 | | | | | | 6.2E-14 | | | | 1.24E-04 | | | | | | 56 | | |
| rs811054 | | | 16 | | 72251132 | | | | | | PMFBP1 | | | C | | | | | | | | T | | | 0.463 | | | | | -0.009 | | | | | | 0.001 | | | | | | 4.4E-10 | | | | 8.59E-05 | | | | | | 39 | | |
| rs4500770 | | | 16 | | 74658430 | | | | | | RFWD3 | | | A | | | | | | | | T | | | 0.637 | | | | | 0.008 | | | | | | 0.001 | | | | | | 4.3E-08 | | | | 6.62E-05 | | | | | | 30 | | |
| rs9673839 | | | 16 | | 76895693 | | | | | | RP11-58C22.1 | | | A | | | | | | | | G | | | 0.509 | | | | | -0.008 | | | | | | 0.001 | | | | | | 1.7E-09 | | | | 8.00E-05 | | | | | | 36 | | |
| rs12926506 | | | 16 | | 81722413 | | | | | | CMIP | | | C | | | | | | | | T | | | 0.830 | | | | | 0.011 | | | | | | 0.002 | | | | | | 1.6E-09 | | | | 8.03E-05 | | | | | | 36 | | |
| rs11150462 | | | 16 | | 82451679 | | | | | | CDH13 | | | T | | | | | | | | A | | | 0.368 | | | | | 0.008 | | | | | | 0.001 | | | | | | 3.1E-09 | | | | 7.75E-05 | | | | | | 35 | | |
| rs7206608 | | | 16 | | 82872628 | | | | | | CDH13 | | | C | | | | | | | | G | | | 0.678 | | | | | -0.010 | | | | | | 0.001 | | | | | | 2.6E-11 | | | | 9.80E-05 | | | | | | 44 | | |
| rs4790292 | | | 17 | | 1824305 | | | | | | RTN4RL1 | | | C | | | | | | | | A | | | 0.846 | | | | | 0.016 | | | | | | 0.002 | | | | | | 1.3E-17 | | | | 1.61E-04 | | | | | | 73 | | |
| rs58351927 | | | 17 | | 5297038 | | | | | | NUP88 | | | A | | | | | | | | G | | | 0.698 | | | | | -0.010 | | | | | | 0.001 | | | | | | 1.2E-11 | | | | 1.01E-04 | | | | | | 46 | | |
| rs4792716 | | | 17 | | 15943144 | | | | | | NCOR1 | | | A | | | | | | | | G | | | 0.438 | | | | | -0.008 | | | | | | 0.001 | | | | | | 4.8E-09 | | | | 7.57E-05 | | | | | | 34 | | |
| rs1320251 | | | 17 | | 21264396 | | | | | | KCNJ12 | | | C | | | | | | | | T | | | 0.545 | | | | | 0.012 | | | | | | 0.001 | | | | | | 3.2E-19 | | | | 1.77E-04 | | | | | | 80 | | |
| rs1017529 | | | 17 | | 27912415 | | | | | | GIT1 | | | C | | | | | | | | A | | | 0.825 | | | | | -0.011 | | | | | | 0.002 | | | | | | 7.7E-09 | | | | 7.36E-05 | | | | | | 33 | | |
| rs73982435 | | | 17 | | 31473455 | | | | | | ASIC2 | | | C | | | | | | | | T | | | 0.784 | | | | | 0.009 | | | | | | 0.002 | | | | | | 1.6E-08 | | | | 7.05E-05 | | | | | | 32 | | |
| rs113962925 | | | 17 | | 46044446 | | | | | | CDK5RAP3 | | | C | | | | | | | | T | | | 0.926 | | | | | -0.017 | | | | | | 0.003 | | | | | | 8.6E-11 | | | | 9.29E-05 | | | | | | 42 | | |
| rs11079849 | | | 17 | | 47090785 | | | | | | IGF2BP1 | | | C | | | | | | | | T | | | 0.671 | | | | | 0.012 | | | | | | 0.001 | | | | | | 6.4E-17 | | | | 1.54E-04 | | | | | | 70 | | |
| rs78369934 | | | 17 | | 61739101 | | | | | | MAP3K3 | | | T | | | | | | | | C | | | 0.945 | | | | | 0.020 | | | | | | 0.003 | | | | | | 6E-11 | | | | 9.45E-05 | | | | | | 43 | | |
| rs11150745 | | | 17 | | 78757626 | | | | | | RPTOR | | | A | | | | | | | | G | | | 0.682 | | | | | 0.014 | | | | | | 0.001 | | | | | | 9.2E-21 | | | | 1.93E-04 | | | | | | 87 | | |
| rs2083323 | | | 18 | | 1856272 | | | | | | METTL4 | | | G | | | | | | | | A | | | 0.822 | | | | | -0.010 | | | | | | 0.002 | | | | | | 7.3E-09 | | | | 7.38E-05 | | | | | | 33 | | |
| rs512121 | | | 18 | | 7548501 | | | | | | PTPRM | | | T | | | | | | | | C | | | 0.808 | | | | | 0.011 | | | | | | 0.002 | | | | | | 1.5E-09 | | | | 8.06E-05 | | | | | | 37 | | |
| rs1788808 | | | 18 | | 21090023 | | | | | | NPC1 | | | A | | | | | | | | G | | | 0.505 | | | | | 0.013 | | | | | | 0.001 | | | | | | 1.9E-20 | | | | 1.89E-04 | | | | | | 86 | | |
| rs16940823 | | | 18 | | 22137319 | | | | | | HRH4 | | | C | | | | | | | | A | | | 0.815 | | | | | 0.011 | | | | | | 0.002 | | | | | | 1.6E-09 | | | | 8.03E-05 | | | | | | 36 | | |
| rs6507054 | | | 18 | | 31248323 | | | | | | ASXL3 | | | T | | | | | | | | C | | | 0.416 | | | | | -0.009 | | | | | | 0.001 | | | | | | 2.7E-11 | | | | 9.80E-05 | | | | | | 44 | | |
| rs559231 | | | 18 | | 39644247 | | | | | | PIK3C3 | | | G | | | | | | | | T | | | 0.607 | | | | | -0.010 | | | | | | 0.001 | | | | | | 1.5E-11 | | | | 1.00E-04 | | | | | | 46 | | |
| rs1834144 | | | 18 | | 40744790 | | | | | | RIT2 | | | C | | | | | | | | A | | | 0.627 | | | | | 0.008 | | | | | | 0.001 | | | | | | 2.5E-08 | | | | 6.86E-05 | | | | | | 31 | | |
| rs7230240 | | | 18 | | 42597978 | | | | | | SETBP1 | | | C | | | | | | | | T | | | 0.705 | | | | | 0.009 | | | | | | 0.002 | | | | | | 4.2E-10 | | | | 8.61E-05 | | | | | | 39 | | |
| rs58243949 | | | 18 | | 52509833 | | | | | | RAB27B | | | C | | | | | | | | T | | | 0.767 | | | | | -0.012 | | | | | | 0.002 | | | | | | 1.3E-13 | | | | 1.21E-04 | | | | | | 55 | | |
| rs11659764 | | | 18 | | 53335512 | | | | | | TCF4 | | | T | | | | | | | | A | | | 0.947 | | | | | 0.018 | | | | | | 0.003 | | | | | | 1.9E-09 | | | | 7.96E-05 | | | | | | 36 | | |
| rs1517037 | | | 18 | | 56878274 | | | | | | GRP | | | C | | | | | | | | T | | | 0.812 | | | | | 0.010 | | | | | | 0.002 | | | | | | 1.6E-08 | | | | 7.03E-05 | | | | | | 32 | | |
| rs58084604 | | | 18 | | 57849429 | | | | | | MC4R | | | C | | | | | | | | T | | | 0.767 | | | | | -0.035 | | | | | | 0.002 | | | | | | 2.6E-104 | | | | 1.04E-03 | | | | | | 470 | | |
| rs17773370 | | | 18 | | 57951433 | | | | | | MC4R | | | G | | | | | | | | A | | | 0.934 | | | | | -0.016 | | | | | | 0.003 | | | | | | 8.7E-09 | | | | 7.31E-05 | | | | | | 33 | | |
| rs57636386 | | | 18 | | 58048295 | | | | | | MC4R | | | T | | | | | | | | C | | | 0.916 | | | | | 0.025 | | | | | | 0.002 | | | | | | 2.7E-23 | | | | 2.18E-04 | | | | | | 99 | | |
| rs12454712 | | | 18 | | 60845884 | | | | | | BCL2 | | | T | | | | | | | | C | | | 0.623 | | | | | -0.008 | | | | | | 0.001 | | | | | | 1E-08 | | | | 7.24E-05 | | | | | | 33 | | |
| rs1373349 | | | 18 | | 63282992 | | | | | | CDH7 | | | C | | | | | | | | T | | | 0.316 | | | | | 0.010 | | | | | | 0.001 | | | | | | 3.5E-11 | | | | 9.68E-05 | | | | | | 44 | | |
| rs8089514 | | | 18 | | 69224478 | | | | | | RP11-723G8.2 | | | T | | | | | | | | A | | | 0.631 | | | | | -0.008 | | | | | | 0.001 | | | | | | 1.4E-08 | | | | 7.11E-05 | | | | | | 32 | | |
| rs45521740 | | | 19 | | 2245622 | | | | | | SF3A2 | | | G | | | | | | | | A | | | 0.943 | | | | | -0.018 | | | | | | 0.003 | | | | | | 1E-09 | | | | 8.23E-05 | | | | | | 37 | | |
| rs72976986 | | | 19 | | 4050424 | | | | | | ZBTB7A | | | G | | | | | | | | A | | | 0.810 | | | | | 0.014 | | | | | | 0.002 | | | | | | 4.4E-15 | | | | 1.36E-04 | | | | | | 62 | | |
| rs75957461 | | | 19 | | 11166163 | | | | | | SMARCA4 | | | C | | | | | | | | T | | | 0.946 | | | | | -0.018 | | | | | | 0.003 | | | | | | 6.6E-09 | | | | 7.43E-05 | | | | | | 34 | | |
| rs6511826 | | | 19 | | 12706991 | | | | | | ZNF490 | | | G | | | | | | | | A | | | 0.087 | | | | | 0.014 | | | | | | 0.002 | | | | | | 1.3E-08 | | | | 7.15E-05 | | | | | | 32 | | |
| rs273505 | | | 19 | | 18217147 | | | | | | MAST3 | | | T | | | | | | | | C | | | 0.578 | | | | | -0.010 | | | | | | 0.001 | | | | | | 3.1E-13 | | | | 1.17E-04 | | | | | | 53 | | |
| rs113230003 | | | 19 | | 18460956 | | | | | | PGPEP1 | | | G | | | | | | | | A | | | 0.739 | | | | | 0.013 | | | | | | 0.002 | | | | | | 1.2E-15 | | | | 1.41E-04 | | | | | | 64 | | |
| rs10404726 | | | 19 | | 18834514 | | | | | | CRTC1 | | | C | | | | | | | | T | | | 0.535 | | | | | 0.012 | | | | | | 0.001 | | | | | | 4.7E-19 | | | | 1.76E-04 | | | | | | 80 | | |
| rs112253053 | | | 19 | | 19425145 | | | | | | SUGP1 | | | T | | | | | | | | A | | | 0.839 | | | | | 0.014 | | | | | | 0.002 | | | | | | 1.8E-13 | | | | 1.20E-04 | | | | | | 54 | | |
| rs12462975 | | | 19 | | 30272202 | | | | | | CCNE1 | | | G | | | | | | | | A | | | 0.670 | | | | | -0.011 | | | | | | 0.001 | | | | | | 5.9E-15 | | | | 1.34E-04 | | | | | | 61 | | |
| rs73026723 | | | 19 | | 31017177 | | | | | | ZNF536 | | | C | | | | | | | | T | | | 0.846 | | | | | 0.014 | | | | | | 0.002 | | | | | | 1.3E-13 | | | | 1.21E-04 | | | | | | 55 | | |
| rs7255223 | | | 19 | | 32824310 | | | | | | ZNF507 | | | C | | | | | | | | A | | | 0.735 | | | | | 0.009 | | | | | | 0.002 | | | | | | 8.5E-09 | | | | 7.32E-05 | | | | | | 33 | | |
| rs429358 | | | 19 | | 45411941 | | | | | | APOE | | | T | | | | | | | | C | | | 0.846 | | | | | 0.016 | | | | | | 0.002 | | | | | | 2.9E-17 | | | | 1.58E-04 | | | | | | 71 | | |
| rs12971645 | | | 19 | | 45807945 | | | | | | MARK4 | | | G | | | | | | | | A | | | 0.725 | | | | | 0.009 | | | | | | 0.002 | | | | | | 2.6E-08 | | | | 6.84E-05 | | | | | | 31 | | |
| rs10423928 | | | 19 | | 46182304 | | | | | | GIPR | | | T | | | | | | | | A | | | 0.806 | | | | | 0.021 | | | | | | 0.002 | | | | | | 4.2E-33 | | | | 3.17E-04 | | | | | | 144 | | |
| rs3810291 | | | 19 | | 47569003 | | | | | | ZC3H4 | | | G | | | | | | | | A | | | 0.325 | | | | | -0.016 | | | | | | 0.001 | | | | | | 4.1E-27 | | | | 2.57E-04 | | | | | | 116 | | |
| rs4545921 | | | 19 | | 49646006 | | | | | | PPFIA3 | | | A | | | | | | | | G | | | 0.400 | | | | | 0.008 | | | | | | 0.001 | | | | | | 4.3E-08 | | | | 6.62E-05 | | | | | | 30 | | |
| rs61746970 | | | 19 | | 51132746 | | | | | | SYT3 | | | G | | | | | | | | A | | | 0.961 | | | | | -0.021 | | | | | | 0.004 | | | | | | 3.8E-09 | | | | 7.66E-05 | | | | | | 35 | | |
| rs8111074 | | | 19 | | 51776117 | | | | | | SIGLECL1 | | | G | | | | | | | | T | | | 0.714 | | | | | 0.008 | | | | | | 0.002 | | | | | | 3.2E-08 | | | | 6.74E-05 | | | | | | 31 | | |
| rs6075658 | | | 20 | | 2094078 | | | | | | STK35 | | | T | | | | | | | | C | | | 0.531 | | | | | 0.008 | | | | | | 0.001 | | | | | | 6.8E-10 | | | | 8.40E-05 | | | | | | 38 | | |
| rs2206925 | | | 20 | | 6634895 | | | | | | BMP2 | | | T | | | | | | | | C | | | 0.364 | | | | | -0.012 | | | | | | 0.001 | | | | | | 2E-16 | | | | 1.49E-04 | | | | | | 68 | | |
| rs4813224 | | | 20 | | 16546846 | | | | | | KIF16B | | | T | | | | | | | | C | | | 0.262 | | | | | -0.009 | | | | | | 0.002 | | | | | | 5.4E-09 | | | | 7.51E-05 | | | | | | 34 | | |
| rs8124896 | | | 20 | | 21385659 | | | | | | NKX2-4 | | | T | | | | | | | | C | | | 0.899 | | | | | -0.013 | | | | | | 0.002 | | | | | | 8.1E-09 | | | | 7.34E-05 | | | | | | 33 | | |
| rs6050446 | | | 20 | | 25195509 | | | | | | ENTPD6 | | | A | | | | | | | | G | | | 0.033 | | | | | -0.026 | | | | | | 0.004 | | | | | | 1.2E-11 | | | | 1.02E-04 | | | | | | 46 | | |
| rs201475383 | | | 20 | | 26273991 | | | | | | FAM182B | | | G | | | | | | | | A | | | 0.966 | | | | | 0.024 | | | | | | 0.004 | | | | | | 8.8E-10 | | | | 8.29E-05 | | | | | | 38 | | |
| rs1987960 | | | 20 | | 30649834 | | | | | | HCK | | | T | | | | | | | | C | | | 0.048 | | | | | -0.019 | | | | | | 0.003 | | | | | | 1.1E-08 | | | | 7.21E-05 | | | | | | 33 | | |
| rs4911382 | | | 20 | | 32553095 | | | | | | RALY | | | C | | | | | | | | T | | | 0.415 | | | | | -0.008 | | | | | | 0.001 | | | | | | 2.6E-09 | | | | 7.83E-05 | | | | | | 35 | | |
| rs6029180 | | | 20 | | 39178923 | | | | | | MAFB | | | A | | | | | | | | G | | | 0.674 | | | | | -0.008 | | | | | | 0.001 | | | | | | 3.1E-08 | | | | 6.76E-05 | | | | | | 31 | | |
| rs6030803 | | | 20 | | 41986507 | | | | | | SRSF6 | | | T | | | | | | | | C | | | 0.873 | | | | | 0.013 | | | | | | 0.002 | | | | | | 4.5E-10 | | | | 8.58E-05 | | | | | | 39 | | |
| rs2425856 | | | 20 | | 44911954 | | | | | | CDH22 | | | A | | | | | | | | G | | | 0.444 | | | | | 0.008 | | | | | | 0.001 | | | | | | 1.1E-09 | | | | 8.20E-05 | | | | | | 37 | | |
| rs112852122 | | | 20 | | 47498117 | | | | | | ARFGEF2 | | | G | | | | | | | | A | | | 0.842 | | | | | 0.013 | | | | | | 0.002 | | | | | | 2.7E-12 | | | | 1.08E-04 | | | | | | 49 | | |
| rs66460909 | | | 20 | | 51195387 | | | | | | ZFP64 | | | G | | | | | | | | A | | | 0.808 | | | | | 0.016 | | | | | | 0.002 | | | | | | 2.9E-19 | | | | 1.78E-04 | | | | | | 81 | | |
| rs559390 | | | 20 | | 54157546 | | | | | | CBLN4 | | | A | | | | | | | | T | | | 0.658 | | | | | -0.008 | | | | | | 0.001 | | | | | | 7.9E-09 | | | | 7.35E-05 | | | | | | 33 | | |
| rs8134638 | | | 21 | | 40644170 | | | | | | BRWD1 | | | T | | | | | | | | C | | | 0.624 | | | | | -0.008 | | | | | | 0.001 | | | | | | 4E-08 | | | | 6.65E-05 | | | | | | 30 | | |
| rs2837398 | | | 21 | | 41427168 | | | | | | DSCAM | | | A | | | | | | | | C | | | 0.596 | | | | | -0.008 | | | | | | 0.001 | | | | | | 4.8E-09 | | | | 7.56E-05 | | | | | | 34 | | |
| rs1964926 | | | 21 | | 42653121 | | | | | | BACE2 | | | A | | | | | | | | G | | | 0.354 | | | | | -0.008 | | | | | | 0.001 | | | | | | 1.1E-08 | | | | 7.21E-05 | | | | | | 33 | | |
| rs403694 | | | 21 | | 46567625 | | | | | | ADARB1 | | | C | | | | | | | | T | | | 0.463 | | | | | -0.012 | | | | | | 0.001 | | | | | | 1.3E-18 | | | | 1.71E-04 | | | | | | 78 | | |
| rs1296685 | | | 22 | | 18230964 | | | | | | BID | | | A | | | | | | | | G | | | 0.789 | | | | | -0.009 | | | | | | 0.002 | | | | | | 2.2E-08 | | | | 6.90E-05 | | | | | | 31 | | |
| rs12484438 | | | 22 | | 40558064 | | | | | | TNRC6B | | | T | | | | | | | | C | | | 0.661 | | | | | 0.013 | | | | | | 0.001 | | | | | | 6.7E-19 | | | | 1.74E-04 | | | | | | 79 | | |
| rs738140 | | | 22 | | 41884954 | | | | | | ACO2 | | | A | | | | | | | | G | | | 0.690 | | | | | 0.009 | | | | | | 0.001 | | | | | | 2.6E-09 | | | | 7.82E-05 | | | | | | 35 | | |
| rs9615723 | | | 22 | | 48386670 | | | | | | FAM19A5 | | | C | | | | | | | | T | | | 0.441 | | | | | 0.008 | | | | | | 0.001 | | | | | | 3.8E-08 | | | | 6.67E-05 | | | | | | 30 | | |
| rs34778589 | | | 22 | | 50709957 | | | | | | MAPK11 | | | A | | | | | | | | C | | | 0.917 | | | | | -0.014 | | | | | | 0.003 | | | | | | 9.8E-09 | | | | 7.26E-05 | | | | | | 33 | | |
| Genome-wide significant SNPs for adult body size in UK Biobank (men only) | | | | | | | | | | | | | | | | | | | | | | | | | | | | | | | | | | | | | | | | | | | | | | | | | | | | | | |
| SNP | | | Chr | | Position | | | | | | Closest gene | | | EA | | | | OA | | | | | EAF | | | | | | Beta | | | | | | SE | | | | | | pvalue | | | | | | | R^2^ | | | | F statistic | | |
| rs3762444 | | | 1 | | 2427712 | | | | | | PLCH2 | | | C | | | | T | | | | | 0.546 | | | | | | 0.012 | | | | | | 0.002 | | | | | | 2.1E-10 | | | | | | | 1.95E-04 | | | | 40 | | |
| rs1284373 | | | 1 | | 33355923 | | | | | | HPCA | | | C | | | | T | | | | | 0.801 | | | | | | 0.014 | | | | | | 0.002 | | | | | | 6.9E-09 | | | | | | | 1.62E-04 | | | | 34 | | |
| rs34052145 | | | 1 | | 38824262 | | | | | | POU3F1 | | | A | | | | G | | | | | 0.647 | | | | | | -0.011 | | | | | | 0.002 | | | | | | 1.4E-08 | | | | | | | 1.56E-04 | | | | 32 | | |
| rs1167311 | | | 1 | | 49996959 | | | | | | AGBL4 | | | G | | | | A | | | | | 0.319 | | | | | | 0.012 | | | | | | 0.002 | | | | | | 7E-09 | | | | | | | 1.62E-04 | | | | 34 | | |
| rs12140153 | | | 1 | | 62579891 | | | | | | INADL | | | G | | | | T | | | | | 0.905 | | | | | | 0.024 | | | | | | 0.003 | | | | | | 1.1E-12 | | | | | | | 2.45E-04 | | | | 51 | | |
| rs11208779 | | | 1 | | 66417145 | | | | | | PDE4B | | | G | | | | C | | | | | 0.471 | | | | | | -0.012 | | | | | | 0.002 | | | | | | 1.9E-09 | | | | | | | 1.75E-04 | | | | 36 | | |
| rs61765650 | | | 1 | | 72753112 | | | | | | NEGR1 | | | A | | | | G | | | | | 0.810 | | | | | | 0.022 | | | | | | 0.002 | | | | | | 2.8E-19 | | | | | | | 3.90E-04 | | | | 81 | | |
| rs34517439 | | | 1 | | 78450517 | | | | | | DNAJB4 | | | C | | | | A | | | | | 0.879 | | | | | | -0.024 | | | | | | 0.003 | | | | | | 3.5E-16 | | | | | | | 3.22E-04 | | | | 67 | | |
| rs2181375 | | | 1 | | 96940119 | | | | | | PTBP2 | | | A | | | | G | | | | | 0.403 | | | | | | -0.012 | | | | | | 0.002 | | | | | | 2.6E-10 | | | | | | | 1.93E-04 | | | | 40 | | |
| rs17024258 | | | 1 | | 110147321 | | | | | | GNAT2 | | | C | | | | T | | | | | 0.974 | | | | | | -0.047 | | | | | | 0.006 | | | | | | 1.4E-14 | | | | | | | 2.86E-04 | | | | 59 | | |
| rs984225 | | | 1 | | 119504284 | | | | | | TBX15 | | | G | | | | A | | | | | 0.389 | | | | | | 0.011 | | | | | | 0.002 | | | | | | 3.4E-08 | | | | | | | 1.47E-04 | | | | 30 | | |
| rs61813324 | | | 1 | | 156049877 | | | | | | MEX3A | | | C | | | | T | | | | | 0.864 | | | | | | -0.016 | | | | | | 0.003 | | | | | | 2.4E-08 | | | | | | | 1.51E-04 | | | | 31 | | |
| rs543874 | | | 1 | | 177889480 | | | | | | SEC16B | | | A | | | | G | | | | | 0.794 | | | | | | -0.022 | | | | | | 0.002 | | | | | | 1.8E-20 | | | | | | | 4.16E-04 | | | | 86 | | |
| rs2125232 | | | 1 | | 243759210 | | | | | | AKT3 | | | C | | | | T | | | | | 0.318 | | | | | | 0.012 | | | | | | 0.002 | | | | | | 2.7E-09 | | | | | | | 1.71E-04 | | | | 35 | | |
| rs1529897 | | | 2 | | 25086827 | | | | | | ADCY3 | | | T | | | | G | | | | | 0.569 | | | | | | -0.016 | | | | | | 0.002 | | | | | | 1E-16 | | | | | | | 3.34E-04 | | | | 69 | | |
| rs935166 | | | 2 | | 26949366 | | | | | | KCNK3 | | | G | | | | A | | | | | 0.493 | | | | | | 0.013 | | | | | | 0.002 | | | | | | 2.5E-11 | | | | | | | 2.16E-04 | | | | 45 | | |
| rs1861410 | | | 2 | | 58933591 | | | | | | FANCL | | | C | | | | T | | | | | 0.446 | | | | | | 0.013 | | | | | | 0.002 | | | | | | 1.5E-11 | | | | | | | 2.20E-04 | | | | 46 | | |
| rs3552 | | | 2 | | 69698158 | | | | | | AAK1 | | | G | | | | A | | | | | 0.458 | | | | | | -0.012 | | | | | | 0.002 | | | | | | 2E-09 | | | | | | | 1.74E-04 | | | | 36 | | |
| rs396354 | | | 2 | | 86850022 | | | | | | CHMP3 | | | T | | | | C | | | | | 0.285 | | | | | | 0.012 | | | | | | 0.002 | | | | | | 3.1E-08 | | | | | | | 1.48E-04 | | | | 31 | | |
| rs6753397 | | | 2 | | 105466651 | | | | | | POU3F3 | | | C | | | | T | | | | | 0.725 | | | | | | -0.012 | | | | | | 0.002 | | | | | | 3E-08 | | | | | | | 1.49E-04 | | | | 31 | | |
| rs13405033 | | | 2 | | 113994595 | | | | | | PAX8 | | | C | | | | T | | | | | 0.846 | | | | | | -0.018 | | | | | | 0.003 | | | | | | 5.6E-12 | | | | | | | 2.30E-04 | | | | 47 | | |
| rs1451077 | | | 2 | | 147901207 | | | | | | ACVR2A | | | G | | | | A | | | | | 0.414 | | | | | | 0.013 | | | | | | 0.002 | | | | | | 2.7E-11 | | | | | | | 2.15E-04 | | | | 44 | | |
| rs7581907 | | | 2 | | 205385454 | | | | | | PARD3B | | | A | | | | G | | | | | 0.127 | | | | | | 0.016 | | | | | | 0.003 | | | | | | 2E-08 | | | | | | | 1.52E-04 | | | | 31 | | |
| rs6436661 | | | 2 | | 228056670 | | | | | | COL4A3 | | | C | | | | T | | | | | 0.113 | | | | | | -0.018 | | | | | | 0.003 | | | | | | 2.6E-08 | | | | | | | 1.50E-04 | | | | 31 | | |
| rs7619139 | | | 3 | | 25110415 | | | | | | RARB | | | T | | | | A | | | | | 0.411 | | | | | | -0.012 | | | | | | 0.002 | | | | | | 9.2E-10 | | | | | | | 1.81E-04 | | | | 37 | | |
| rs2526389 | | | 3 | | 50192826 | | | | | | SEMA3F | | | C | | | | T | | | | | 0.573 | | | | | | -0.019 | | | | | | 0.002 | | | | | | 1E-22 | | | | | | | 4.66E-04 | | | | 96 | | |
| rs2336558 | | | 3 | | 52886240 | | | | | | TMEM110 | | | C | | | | T | | | | | 0.384 | | | | | | 0.012 | | | | | | 0.002 | | | | | | 3.8E-10 | | | | | | | 1.90E-04 | | | | 39 | | |
| rs11708540 | | | 3 | | 70593081 | | | | | | FOXP1 | | | G | | | | A | | | | | 0.843 | | | | | | -0.015 | | | | | | 0.003 | | | | | | 1.6E-08 | | | | | | | 1.55E-04 | | | | 32 | | |
| rs55782528 | | | 3 | | 85893825 | | | | | | CADM2 | | | C | | | | A | | | | | 0.646 | | | | | | 0.013 | | | | | | 0.002 | | | | | | 1.2E-10 | | | | | | | 2.00E-04 | | | | 41 | | |
| rs9861443 | | | 3 | | 88196211 | | | | | | CGGBP1 | | | A | | | | C | | | | | 0.285 | | | | | | -0.013 | | | | | | 0.002 | | | | | | 2.8E-09 | | | | | | | 1.71E-04 | | | | 35 | | |
| rs13066686 | | | 3 | | 94075026 | | | | | | NSUN3 | | | C | | | | A | | | | | 0.594 | | | | | | 0.013 | | | | | | 0.002 | | | | | | 1.2E-11 | | | | | | | 2.22E-04 | | | | 46 | | |
| rs2918217 | | | 3 | | 115546380 | | | | | | LSAMP | | | C | | | | T | | | | | 0.859 | | | | | | -0.018 | | | | | | 0.003 | | | | | | 5.7E-11 | | | | | | | 2.08E-04 | | | | 43 | | |
| rs73875019 | | | 3 | | 131521276 | | | | | | CPNE4 | | | T | | | | A | | | | | 0.883 | | | | | | 0.019 | | | | | | 0.003 | | | | | | 1.1E-10 | | | | | | | 2.02E-04 | | | | 42 | | |
| rs1320903 | | | 3 | | 131758077 | | | | | | CPNE4 | | | G | | | | A | | | | | 0.681 | | | | | | -0.016 | | | | | | 0.002 | | | | | | 1.2E-14 | | | | | | | 2.88E-04 | | | | 60 | | |
| rs10935143 | | | 3 | | 134665159 | | | | | | EPHB1 | | | G | | | | A | | | | | 0.551 | | | | | | 0.011 | | | | | | 0.002 | | | | | | 2.2E-08 | | | | | | | 1.52E-04 | | | | 31 | | |
| rs61789562 | | | 3 | | 135926784 | | | | | | MSL2 | | | T | | | | C | | | | | 0.882 | | | | | | 0.017 | | | | | | 0.003 | | | | | | 1.5E-08 | | | | | | | 1.55E-04 | | | | 32 | | |
| rs1568488 | | | 3 | | 153657951 | | | | | | ARHGEF26 | | | G | | | | C | | | | | 0.405 | | | | | | -0.012 | | | | | | 0.002 | | | | | | 2.7E-09 | | | | | | | 1.71E-04 | | | | 35 | | |
| rs8192675 | | | 3 | | 170724883 | | | | | | SLC2A2 | | | T | | | | C | | | | | 0.712 | | | | | | -0.014 | | | | | | 0.002 | | | | | | 1.7E-10 | | | | | | | 1.98E-04 | | | | 41 | | |
| rs894000 | | | 3 | | 183536063 | | | | | | MAP6D1 | | | T | | | | C | | | | | 0.372 | | | | | | 0.013 | | | | | | 0.002 | | | | | | 3.7E-11 | | | | | | | 2.12E-04 | | | | 44 | | |
| rs55742087 | | | 3 | | 185830488 | | | | | | DGKG | | | C | | | | T | | | | | 0.815 | | | | | | 0.019 | | | | | | 0.002 | | | | | | 1.5E-14 | | | | | | | 2.86E-04 | | | | 59 | | |
| rs10938398 | | | 4 | | 45186139 | | | | | | GNPDA2 | | | G | | | | A | | | | | 0.565 | | | | | | -0.021 | | | | | | 0.002 | | | | | | 2.3E-28 | | | | | | | 5.90E-04 | | | | 122 | | |
| rs2537860 | | | 4 | | 55484718 | | | | | | KIT | | | A | | | | C | | | | | 0.437 | | | | | | 0.011 | | | | | | 0.002 | | | | | | 6E-09 | | | | | | | 1.64E-04 | | | | 34 | | |
| rs13107325 | | | 4 | | 103188709 | | | | | | SLC39A8 | | | C | | | | T | | | | | 0.924 | | | | | | -0.029 | | | | | | 0.004 | | | | | | 4.7E-16 | | | | | | | 3.19E-04 | | | | 66 | | |
| rs1296328 | | | 4 | | 137083193 | | | | | | PCDH18 | | | A | | | | C | | | | | 0.440 | | | | | | 0.013 | | | | | | 0.002 | | | | | | 7E-12 | | | | | | | 2.28E-04 | | | | 47 | | |
| rs2307111 | | | 5 | | 75003678 | | | | | | POC5 | | | T | | | | C | | | | | 0.605 | | | | | | 0.013 | | | | | | 0.002 | | | | | | 3.7E-11 | | | | | | | 2.12E-04 | | | | 44 | | |
| rs7703782 | | | 5 | | 87938557 | | | | | | MEF2C | | | T | | | | A | | | | | 0.868 | | | | | | -0.024 | | | | | | 0.003 | | | | | | 7.4E-17 | | | | | | | 3.37E-04 | | | | 70 | | |
| rs1459843 | | | 5 | | 95867223 | | | | | | CAST | | | C | | | | A | | | | | 0.392 | | | | | | 0.012 | | | | | | 0.002 | | | | | | 4.2E-09 | | | | | | | 1.67E-04 | | | | 35 | | |
| rs2591496 | | | 5 | | 170516965 | | | | | | RANBP17 | | | G | | | | A | | | | | 0.270 | | | | | | -0.013 | | | | | | 0.002 | | | | | | 2.4E-09 | | | | | | | 1.72E-04 | | | | 36 | | |
| rs9379829 | | | 6 | | 26172219 | | | | | | HIST1H2BD | | | C | | | | T | | | | | 0.782 | | | | | | 0.013 | | | | | | 0.002 | | | | | | 3.1E-08 | | | | | | | 1.48E-04 | | | | 31 | | |
| rs2260051 | | | 6 | | 31591918 | | | | | | PRRC2A | | | A | | | | T | | | | | 0.439 | | | | | | -0.012 | | | | | | 0.002 | | | | | | 1.4E-09 | | | | | | | 1.77E-04 | | | | 37 | | |
| rs9277992 | | | 6 | | 33312455 | | | | | | DAXX | | | G | | | | A | | | | | 0.810 | | | | | | -0.016 | | | | | | 0.002 | | | | | | 6.8E-11 | | | | | | | 2.06E-04 | | | | 43 | | |
| rs9469899 | | | 6 | | 34793124 | | | | | | UHRF1BP1 | | | G | | | | A | | | | | 0.640 | | | | | | -0.013 | | | | | | 0.002 | | | | | | 8.2E-11 | | | | | | | 2.04E-04 | | | | 42 | | |
| rs9471333 | | | 6 | | 40362023 | | | | | | LRFN2 | | | C | | | | T | | | | | 0.447 | | | | | | 0.012 | | | | | | 0.002 | | | | | | 3.8E-10 | | | | | | | 1.90E-04 | | | | 39 | | |
| rs3798519 | | | 6 | | 50788778 | | | | | | TFAP2B | | | A | | | | C | | | | | 0.820 | | | | | | -0.024 | | | | | | 0.003 | | | | | | 3.8E-21 | | | | | | | 4.31E-04 | | | | 89 | | |
| rs74621225 | | | 6 | | 97804399 | | | | | | MMS22L | | | A | | | | G | | | | | 0.866 | | | | | | 0.016 | | | | | | 0.003 | | | | | | 8.9E-09 | | | | | | | 1.60E-04 | | | | 33 | | |
| rs9320823 | | | 6 | | 98429337 | | | | | | MMS22L | | | T | | | | C | | | | | 0.397 | | | | | | -0.013 | | | | | | 0.002 | | | | | | 1.3E-11 | | | | | | | 2.21E-04 | | | | 46 | | |
| rs9478671 | | | 6 | | 155987825 | | | | | | NOX3 | | | A | | | | G | | | | | 0.789 | | | | | | -0.014 | | | | | | 0.002 | | | | | | 3.1E-09 | | | | | | | 1.70E-04 | | | | 35 | | |
| rs34714518 | | | 6 | | 163080778 | | | | | | PARK2 | | | G | | | | A | | | | | 0.877 | | | | | | 0.016 | | | | | | 0.003 | | | | | | 2.6E-08 | | | | | | | 1.50E-04 | | | | 31 | | |
| rs12666574 | | | 7 | | 26526960 | | | | | | KIAA0087 | | | G | | | | A | | | | | 0.327 | | | | | | -0.012 | | | | | | 0.002 | | | | | | 1.7E-08 | | | | | | | 1.54E-04 | | | | 32 | | |
| rs62457529 | | | 7 | | 32349219 | | | | | | PDE1C | | | A | | | | G | | | | | 0.891 | | | | | | -0.019 | | | | | | 0.003 | | | | | | 9.4E-10 | | | | | | | 1.81E-04 | | | | 37 | | |
| rs6962280 | | | 7 | | 44788657 | | | | | | ZMIZ2 | | | A | | | | G | | | | | 0.442 | | | | | | -0.012 | | | | | | 0.002 | | | | | | 3.8E-10 | | | | | | | 1.90E-04 | | | | 39 | | |
| rs17145600 | | | 7 | | 73994733 | | | | | | GTF2IRD1 | | | C | | | | T | | | | | 0.949 | | | | | | -0.025 | | | | | | 0.004 | | | | | | 2E-08 | | | | | | | 1.52E-04 | | | | 31 | | |
| rs12538826 | | | 7 | | 99030228 | | | | | | PTCD1 | | | T | | | | C | | | | | 0.885 | | | | | | 0.017 | | | | | | 0.003 | | | | | | 2E-08 | | | | | | | 1.52E-04 | | | | 31 | | |
| rs10236214 | | | 7 | | 150668070 | | | | | | KCNH2 | | | C | | | | T | | | | | 0.358 | | | | | | -0.011 | | | | | | 0.002 | | | | | | 2.1E-08 | | | | | | | 1.52E-04 | | | | 31 | | |
| rs4840941 | | | 8 | | 8216986 | | | | | | SGK223 | | | A | | | | G | | | | | 0.470 | | | | | | -0.014 | | | | | | 0.002 | | | | | | 2.1E-12 | | | | | | | 2.39E-04 | | | | 49 | | |
| rs9329197 | | | 8 | | 9259568 | | | | | | TNKS | | | A | | | | T | | | | | 0.380 | | | | | | 0.013 | | | | | | 0.002 | | | | | | 1.8E-10 | | | | | | | 1.97E-04 | | | | 41 | | |
| rs3750310 | | | 8 | | 10283426 | | | | | | MSRA | | | G | | | | A | | | | | 0.488 | | | | | | 0.012 | | | | | | 0.002 | | | | | | 3.6E-10 | | | | | | | 1.90E-04 | | | | 39 | | |
| rs11780420 | | | 8 | | 11446421 | | | | | | BLK | | | G | | | | A | | | | | 0.415 | | | | | | 0.014 | | | | | | 0.002 | | | | | | 3.6E-12 | | | | | | | 2.34E-04 | | | | 48 | | |
| rs7827210 | | | 8 | | 30871656 | | | | | | PURG | | | G | | | | A | | | | | 0.604 | | | | | | -0.011 | | | | | | 0.002 | | | | | | 1.7E-08 | | | | | | | 1.54E-04 | | | | 32 | | |
| rs35732620 | | | 8 | | 73445881 | | | | | | KCNB2 | | | G | | | | T | | | | | 0.425 | | | | | | 0.012 | | | | | | 0.002 | | | | | | 2.6E-09 | | | | | | | 1.71E-04 | | | | 35 | | |
| rs1812736 | | | 8 | | 76299138 | | | | | | HNF4G | | | G | | | | A | | | | | 0.174 | | | | | | -0.014 | | | | | | 0.003 | | | | | | 2.3E-08 | | | | | | | 1.51E-04 | | | | 31 | | |
| rs72674843 | | | 8 | | 95533000 | | | | | | KIAA1429 | | | T | | | | C | | | | | 0.761 | | | | | | 0.015 | | | | | | 0.002 | | | | | | 2.8E-11 | | | | | | | 2.14E-04 | | | | 44 | | |
| rs800526 | | | 8 | | 116845729 | | | | | | TRPS1 | | | A | | | | C | | | | | 0.223 | | | | | | -0.014 | | | | | | 0.002 | | | | | | 7.1E-10 | | | | | | | 1.84E-04 | | | | 38 | | |
| rs1412239 | | | 9 | | 28425515 | | | | | | LINGO2 | | | C | | | | G | | | | | 0.678 | | | | | | -0.015 | | | | | | 0.002 | | | | | | 4.2E-14 | | | | | | | 2.76E-04 | | | | 57 | | |
| rs10828247 | | | 10 | | 21822856 | | | | | | MLLT10 | | | A | | | | G | | | | | 0.657 | | | | | | -0.011 | | | | | | 0.002 | | | | | | 2.7E-08 | | | | | | | 1.49E-04 | | | | 31 | | |
| rs10824218 | | | 10 | | 76421216 | | | | | | ADK | | | A | | | | T | | | | | 0.563 | | | | | | 0.011 | | | | | | 0.002 | | | | | | 4.7E-08 | | | | | | | 1.44E-04 | | | | 30 | | |
| rs10883026 | | | 10 | | 99793865 | | | | | | CRTAC1 | | | C | | | | T | | | | | 0.479 | | | | | | 0.013 | | | | | | 0.002 | | | | | | 1E-10 | | | | | | | 2.02E-04 | | | | 42 | | |
| rs117597828 | | | 10 | | 102416055 | | | | | | PAX2 | | | C | | | | T | | | | | 0.782 | | | | | | -0.014 | | | | | | 0.002 | | | | | | 1E-09 | | | | | | | 1.80E-04 | | | | 37 | | |
| rs4962671 | | | 10 | | 126305434 | | | | | | LHPP | | | T | | | | C | | | | | 0.532 | | | | | | 0.011 | | | | | | 0.002 | | | | | | 2.7E-08 | | | | | | | 1.49E-04 | | | | 31 | | |
| rs72867447 | | | 11 | | 13301875 | | | | | | ARNTL | | | C | | | | G | | | | | 0.428 | | | | | | -0.011 | | | | | | 0.002 | | | | | | 9.5E-09 | | | | | | | 1.59E-04 | | | | 33 | | |
| rs6265 | | | 11 | | 27679916 | | | | | | BDNF | | | C | | | | T | | | | | 0.811 | | | | | | 0.028 | | | | | | 0.002 | | | | | | 3E-31 | | | | | | | 6.54E-04 | | | | 135 | | |
| rs1222216 | | | 11 | | 30346052 | | | | | | ARL14EP | | | C | | | | T | | | | | 0.773 | | | | | | 0.014 | | | | | | 0.002 | | | | | | 2.6E-09 | | | | | | | 1.71E-04 | | | | 35 | | |
| rs4755726 | | | 11 | | 43642130 | | | | | | HSD17B12 | | | T | | | | G | | | | | 0.310 | | | | | | 0.014 | | | | | | 0.002 | | | | | | 2.5E-11 | | | | | | | 2.15E-04 | | | | 45 | | |
| rs12798028 | | | 11 | | 47604639 | | | | | | NDUFS3 | | | C | | | | T | | | | | 0.592 | | | | | | -0.015 | | | | | | 0.002 | | | | | | 9.6E-15 | | | | | | | 2.90E-04 | | | | 60 | | |
| rs7940691 | | | 11 | | 65640906 | | | | | | EFEMP2 | | | C | | | | T | | | | | 0.361 | | | | | | 0.013 | | | | | | 0.002 | | | | | | 1.8E-11 | | | | | | | 2.19E-04 | | | | 45 | | |
| rs10898317 | | | 11 | | 84613479 | | | | | | DLG2 | | | C | | | | T | | | | | 0.487 | | | | | | 0.011 | | | | | | 0.002 | | | | | | 3E-08 | | | | | | | 1.49E-04 | | | | 31 | | |
| rs55726687 | | | 12 | | 991306 | | | | | | WNK1 | | | G | | | | A | | | | | 0.792 | | | | | | -0.013 | | | | | | 0.002 | | | | | | 4.7E-08 | | | | | | | 1.44E-04 | | | | 30 | | |
| rs76895963 | | | 12 | | 4384844 | | | | | | CCND2 | | | T | | | | G | | | | | 0.979 | | | | | | -0.042 | | | | | | 0.007 | | | | | | 1.3E-08 | | | | | | | 1.57E-04 | | | | 32 | | |
| rs7132908 | | | 12 | | 50263148 | | | | | | FAIM2 | | | G | | | | A | | | | | 0.615 | | | | | | -0.019 | | | | | | 0.002 | | | | | | 5.3E-22 | | | | | | | 4.50E-04 | | | | 93 | | |
| rs4759228 | | | 12 | | 56508409 | | | | | | PA2G4 | | | G | | | | C | | | | | 0.703 | | | | | | 0.012 | | | | | | 0.002 | | | | | | 2.5E-08 | | | | | | | 1.50E-04 | | | | 31 | | |
| rs7308188 | | | 12 | | 103701537 | | | | | | C12orf42 | | | T | | | | C | | | | | 0.745 | | | | | | 0.014 | | | | | | 0.002 | | | | | | 5.3E-10 | | | | | | | 1.87E-04 | | | | 39 | | |
| rs6490030 | | | 12 | | 116106755 | | | | | | MED13L | | | C | | | | A | | | | | 0.626 | | | | | | -0.011 | | | | | | 0.002 | | | | | | 3.8E-08 | | | | | | | 1.46E-04 | | | | 30 | | |
| rs147730268 | | | 12 | | 123024476 | | | | | | KNTC1 | | | G | | | | T | | | | | 0.913 | | | | | | 0.022 | | | | | | 0.003 | | | | | | 6.1E-10 | | | | | | | 1.85E-04 | | | | 38 | | |
| rs11619722 | | | 13 | | 27998600 | | | | | | GTF3A | | | T | | | | C | | | | | 0.699 | | | | | | 0.012 | | | | | | 0.002 | | | | | | 7.1E-09 | | | | | | | 1.62E-04 | | | | 34 | | |
| rs61954177 | | | 13 | | 40787036 | | | | | | AL133318.1 | | | G | | | | C | | | | | 0.673 | | | | | | -0.012 | | | | | | 0.002 | | | | | | 2E-08 | | | | | | | 1.53E-04 | | | | 32 | | |
| rs4477562 | | | 13 | | 54104968 | | | | | | OLFM4 | | | C | | | | T | | | | | 0.871 | | | | | | -0.021 | | | | | | 0.003 | | | | | | 8E-13 | | | | | | | 2.48E-04 | | | | 51 | | |
| rs9317002 | | | 13 | | 59175727 | | | | | | PCDH17 | | | C | | | | A | | | | | 0.486 | | | | | | -0.015 | | | | | | 0.002 | | | | | | 3E-14 | | | | | | | 2.79E-04 | | | | 58 | | |
| rs7983454 | | | 13 | | 111850539 | | | | | | ARHGEF7 | | | T | | | | C | | | | | 0.477 | | | | | | 0.011 | | | | | | 0.002 | | | | | | 1.2E-08 | | | | | | | 1.57E-04 | | | | 33 | | |
| rs9522279 | | | 13 | | 112221296 | | | | | | RP11-65D24.2 | | | C | | | | T | | | | | 0.577 | | | | | | -0.013 | | | | | | 0.002 | | | | | | 7.3E-11 | | | | | | | 2.05E-04 | | | | 42 | | |
| rs10132280 | | | 14 | | 25928179 | | | | | | STXBP6 | | | C | | | | A | | | | | 0.698 | | | | | | 0.016 | | | | | | 0.002 | | | | | | 9.9E-15 | | | | | | | 2.90E-04 | | | | 60 | | |
| rs9788550 | | | 14 | | 29681138 | | | | | | PRKD1 | | | G | | | | C | | | | | 0.753 | | | | | | 0.015 | | | | | | 0.002 | | | | | | 2E-11 | | | | | | | 2.18E-04 | | | | 45 | | |
| rs2143975 | | | 14 | | 33297398 | | | | | | AKAP6 | | | C | | | | G | | | | | 0.466 | | | | | | 0.013 | | | | | | 0.002 | | | | | | 4.6E-12 | | | | | | | 2.31E-04 | | | | 48 | | |
| rs10131761 | | | 14 | | 40752651 | | | | | | FBXO33 | | | T | | | | A | | | | | 0.812 | | | | | | 0.014 | | | | | | 0.002 | | | | | | 4E-08 | | | | | | | 1.46E-04 | | | | 30 | | |
| rs4898556 | | | 14 | | 47299082 | | | | | | MDGA2 | | | A | | | | C | | | | | 0.492 | | | | | | 0.013 | | | | | | 0.002 | | | | | | 1.9E-11 | | | | | | | 2.18E-04 | | | | 45 | | |
| rs217669 | | | 14 | | 62360075 | | | | | | SYT16 | | | T | | | | C | | | | | 0.728 | | | | | | -0.014 | | | | | | 0.002 | | | | | | 3.4E-10 | | | | | | | 1.91E-04 | | | | 39 | | |
| rs8008910 | | | 14 | | 79944099 | | | | | | NRXN3 | | | G | | | | A | | | | | 0.778 | | | | | | -0.017 | | | | | | 0.002 | | | | | | 2.3E-13 | | | | | | | 2.60E-04 | | | | 54 | | |
| rs8008772 | | | 14 | | 88321884 | | | | | | GALC | | | A | | | | T | | | | | 0.750 | | | | | | -0.013 | | | | | | 0.002 | | | | | | 1.6E-08 | | | | | | | 1.54E-04 | | | | 32 | | |
| rs1887197 | | | 14 | | 94187832 | | | | | | PRIMA1 | | | C | | | | T | | | | | 0.347 | | | | | | -0.014 | | | | | | 0.002 | | | | | | 2.3E-11 | | | | | | | 2.16E-04 | | | | 45 | | |
| rs61992671 | | | 14 | | 101531854 | | | | | | AL117190.3 | | | A | | | | G | | | | | 0.508 | | | | | | 0.011 | | | | | | 0.002 | | | | | | 4.9E-08 | | | | | | | 1.44E-04 | | | | 30 | | |
| rs11631651 | | | 15 | | 73646487 | | | | | | HCN4 | | | A | | | | C | | | | | 0.927 | | | | | | 0.022 | | | | | | 0.004 | | | | | | 1.3E-09 | | | | | | | 1.78E-04 | | | | 37 | | |
| rs57488047 | | | 15 | | 79403002 | | | | | | RASGRF1 | | | T | | | | C | | | | | 0.532 | | | | | | 0.012 | | | | | | 0.002 | | | | | | 3E-09 | | | | | | | 1.70E-04 | | | | 35 | | |
| rs2238435 | | | 16 | | 4014282 | | | | | | ADCY9 | | | C | | | | G | | | | | 0.386 | | | | | | -0.011 | | | | | | 0.002 | | | | | | 2.5E-08 | | | | | | | 1.50E-04 | | | | 31 | | |
| rs1990573 | | | 16 | | 9713688 | | | | | | RP11-297M9.1 | | | A | | | | G | | | | | 0.306 | | | | | | -0.013 | | | | | | 0.002 | | | | | | 1.7E-09 | | | | | | | 1.76E-04 | | | | 36 | | |
| rs8054079 | | | 16 | | 19975407 | | | | | | GPR139 | | | C | | | | T | | | | | 0.860 | | | | | | 0.016 | | | | | | 0.003 | | | | | | 8.8E-09 | | | | | | | 1.60E-04 | | | | 33 | | |
| rs27741 | | | 16 | | 28504181 | | | | | | CLN3 | | | G | | | | A | | | | | 0.584 | | | | | | -0.017 | | | | | | 0.002 | | | | | | 8E-19 | | | | | | | 3.80E-04 | | | | 78 | | |
| rs62048402 | | | 16 | | 53803223 | | | | | | FTO | | | G | | | | A | | | | | 0.594 | | | | | | -0.047 | | | | | | 0.002 | | | | | | 6.9E-130 | | | | | | | 2.84E-03 | | | | 588 | | |
| rs12923231 | | | 16 | | 69572892 | | | | | | NFAT5 | | | C | | | | T | | | | | 0.590 | | | | | | 0.014 | | | | | | 0.002 | | | | | | 3.9E-13 | | | | | | | 2.55E-04 | | | | 53 | | |
| rs12149660 | | | 16 | | 70309237 | | | | | | AARS | | | G | | | | A | | | | | 0.885 | | | | | | 0.020 | | | | | | 0.003 | | | | | | 3E-11 | | | | | | | 2.14E-04 | | | | 44 | | |
| rs3923783 | | | 17 | | 1843189 | | | | | | RTN4RL1 | | | C | | | | A | | | | | 0.815 | | | | | | 0.016 | | | | | | 0.002 | | | | | | 1E-10 | | | | | | | 2.02E-04 | | | | 42 | | |
| rs9901404 | | | 17 | | 21213782 | | | | | | MAP2K3 | | | A | | | | G | | | | | 0.473 | | | | | | 0.016 | | | | | | 0.002 | | | | | | 1.1E-13 | | | | | | | 2.67E-04 | | | | 55 | | |
| rs12941009 | | | 17 | | 34961051 | | | | | | MRM1 | | | C | | | | T | | | | | 0.598 | | | | | | 0.011 | | | | | | 0.002 | | | | | | 1.2E-08 | | | | | | | 1.57E-04 | | | | 32 | | |
| rs11079849 | | | 17 | | 47090785 | | | | | | IGF2BP1 | | | C | | | | T | | | | | 0.672 | | | | | | 0.012 | | | | | | 0.002 | | | | | | 6.4E-09 | | | | | | | 1.63E-04 | | | | 34 | | |
| rs11150745 | | | 17 | | 78757626 | | | | | | RPTOR | | | A | | | | G | | | | | 0.683 | | | | | | 0.013 | | | | | | 0.002 | | | | | | 6.8E-11 | | | | | | | 2.06E-04 | | | | 43 | | |
| rs1652376 | | | 18 | | 21109466 | | | | | | NPC1 | | | G | | | | T | | | | | 0.538 | | | | | | 0.015 | | | | | | 0.002 | | | | | | 2.6E-14 | | | | | | | 2.80E-04 | | | | 58 | | |
| rs7232171 | | | 18 | | 31251221 | | | | | | ASXL3 | | | G | | | | T | | | | | 0.418 | | | | | | -0.011 | | | | | | 0.002 | | | | | | 2.2E-08 | | | | | | | 1.51E-04 | | | | 31 | | |
| rs58243949 | | | 18 | | 52509833 | | | | | | RAB27B | | | C | | | | T | | | | | 0.767 | | | | | | -0.014 | | | | | | 0.002 | | | | | | 2.3E-09 | | | | | | | 1.73E-04 | | | | 36 | | |
| rs7240682 | | | 18 | | 57824038 | | | | | | MC4R | | | C | | | | G | | | | | 0.772 | | | | | | -0.031 | | | | | | 0.002 | | | | | | 8.7E-42 | | | | | | | 8.87E-04 | | | | 183 | | |
| rs8112818 | | | 19 | | 18812785 | | | | | | CRTC1 | | | A | | | | G | | | | | 0.601 | | | | | | 0.012 | | | | | | 0.002 | | | | | | 4.6E-09 | | | | | | | 1.66E-04 | | | | 34 | | |
| rs10423928 | | | 19 | | 46182304 | | | | | | GIPR | | | T | | | | A | | | | | 0.806 | | | | | | 0.019 | | | | | | 0.002 | | | | | | 1.2E-14 | | | | | | | 2.88E-04 | | | | 60 | | |
| rs3810291 | | | 19 | | 47569003 | | | | | | ZC3H4 | | | G | | | | A | | | | | 0.326 | | | | | | -0.016 | | | | | | 0.002 | | | | | | 6.3E-15 | | | | | | | 2.94E-04 | | | | 61 | | |
| rs6054427 | | | 20 | | 6635266 | | | | | | BMP2 | | | G | | | | A | | | | | 0.378 | | | | | | -0.014 | | | | | | 0.002 | | | | | | 8.9E-12 | | | | | | | 2.25E-04 | | | | 47 | | |
| rs6096886 | | | 20 | | 50951298 | | | | | | ZFP64 | | | A | | | | G | | | | | 0.810 | | | | | | 0.016 | | | | | | 0.002 | | | | | | 2.4E-10 | | | | | | | 1.94E-04 | | | | 40 | | |
| rs9977825 | | | 21 | | 46494995 | | | | | | ADARB1 | | | T | | | | C | | | | | 0.361 | | | | | | 0.012 | | | | | | 0.002 | | | | | | 5.8E-09 | | | | | | | 1.64E-04 | | | | 34 | | |
| rs17421586 | | | 22 | | 40591312 | | | | | | TNRC6B | | | T | | | | A | | | | | 0.660 | | | | | | 0.012 | | | | | | 0.002 | | | | | | 3.4E-09 | | | | | | | 1.69E-04 | | | | 35 | | |
| Genome-wide significant SNPs for adult body size in UK Biobank (women only) | | | | | | | | | | | | | | | | | | | | | | | | | | | | | | | | | | | | | | | | | | | | | | | | | | | | | | |
| SNP | | Chr | | | Position | | | | Closest gene | | | | EA | | | | | | | OA | | | | EAF | | | | Beta | | | | | | SE | | | | | | pvalue | | | | | | | R^2^ | | | | F statistic | | | |
| rs74892851 | | 1 | | | 1563789 | | | | MIB2 | | | | C | | | | | | | A | | | | 0.623 | | | | 0.012 | | | | | | 0.002 | | | | | | 1.9E-08 | | | | | | | 1.28E-04 | | | | 32 | | | |
| rs78886584 | | 1 | | | 16859325 | | | | FAM231B | | | | A | | | | | | | G | | | | 0.509 | | | | -0.013 | | | | | | 0.002 | | | | | | 2.6E-11 | | | | | | | 1.80E-04 | | | | 44 | | | |
| rs72660086 | | 1 | | | 39571992 | | | | MACF1 | | | | T | | | | | | | G | | | | 0.789 | | | | -0.013 | | | | | | 0.002 | | | | | | 3.2E-08 | | | | | | | 1.24E-04 | | | | 31 | | | |
| rs12144626 | | 1 | | | 47670525 | | | | TAL1 | | | | T | | | | | | | C | | | | 0.417 | | | | 0.012 | | | | | | 0.002 | | | | | | 4.4E-09 | | | | | | | 1.40E-04 | | | | 34 | | | |
| rs1494461 | | 1 | | | 49787196 | | | | AGBL4 | | | | C | | | | | | | T | | | | 0.321 | | | | 0.013 | | | | | | 0.002 | | | | | | 1.4E-09 | | | | | | | 1.49E-04 | | | | 37 | | | |
| rs12140153 | | 1 | | | 62579891 | | | | INADL | | | | G | | | | | | | T | | | | 0.906 | | | | 0.019 | | | | | | 0.003 | | | | | | 2.3E-08 | | | | | | | 1.27E-04 | | | | 31 | | | |
| rs2815757 | | 1 | | | 72764289 | | | | NEGR1 | | | | C | | | | | | | T | | | | 0.191 | | | | -0.017 | | | | | | 0.002 | | | | | | 2E-11 | | | | | | | 1.82E-04 | | | | 45 | | | |
| rs1514173 | | 1 | | | 74995110 | | | | TNNI3K | | | | C | | | | | | | T | | | | 0.598 | | | | -0.013 | | | | | | 0.002 | | | | | | 1.9E-11 | | | | | | | 1.83E-04 | | | | 45 | | | |
| rs34517439 | | 1 | | | 78450517 | | | | DNAJB4 | | | | C | | | | | | | A | | | | 0.878 | | | | -0.025 | | | | | | 0.003 | | | | | | 8.4E-17 | | | | | | | 2.81E-04 | | | | 69 | | | |
| rs10922911 | | 1 | | | 91205831 | | | | BARHL2 | | | | C | | | | | | | T | | | | 0.629 | | | | -0.014 | | | | | | 0.002 | | | | | | 1.1E-11 | | | | | | | 1.87E-04 | | | | 46 | | | |
| rs653958 | | 1 | | | 96884006 | | | | PTBP2 | | | | A | | | | | | | G | | | | 0.629 | | | | -0.012 | | | | | | 0.002 | | | | | | 1.1E-08 | | | | | | | 1.33E-04 | | | | 33 | | | |
| rs75641275 | | 1 | | | 98327133 | | | | DPYD | | | | A | | | | | | | C | | | | 0.857 | | | | -0.018 | | | | | | 0.003 | | | | | | 4.7E-11 | | | | | | | 1.76E-04 | | | | 43 | | | |
| rs41279738 | | 1 | | | 110082551 | | | | GPR61 | | | | T | | | | | | | G | | | | 0.974 | | | | -0.041 | | | | | | 0.006 | | | | | | 3.4E-11 | | | | | | | 1.78E-04 | | | | 44 | | | |
| rs12033257 | | 1 | | | 112318484 | | | | KCND3 | | | | A | | | | | | | G | | | | 0.618 | | | | 0.012 | | | | | | 0.002 | | | | | | 1.9E-09 | | | | | | | 1.46E-04 | | | | 36 | | | |
| rs3753639 | | 1 | | | 154986091 | | | | ZBTB7B | | | | T | | | | | | | C | | | | 0.756 | | | | -0.013 | | | | | | 0.002 | | | | | | 3.7E-08 | | | | | | | 1.23E-04 | | | | 30 | | | |
| rs61813324 | | 1 | | | 156049877 | | | | MEX3A | | | | C | | | | | | | T | | | | 0.865 | | | | -0.021 | | | | | | 0.003 | | | | | | 7.6E-13 | | | | | | | 2.08E-04 | | | | 51 | | | |
| rs539515 | | 1 | | | 177889025 | | | | SEC16B | | | | A | | | | | | | C | | | | 0.795 | | | | -0.038 | | | | | | 0.002 | | | | | | 9.6E-54 | | | | | | | 9.65E-04 | | | | 238 | | | |
| rs815163 | | 1 | | | 190294726 | | | | BRINP3 | | | | T | | | | | | | C | | | | 0.438 | | | | 0.013 | | | | | | 0.002 | | | | | | 2.4E-11 | | | | | | | 1.81E-04 | | | | 45 | | | |
| rs2678204 | | 1 | | | 201800511 | | | | IPO9 | | | | T | | | | | | | G | | | | 0.659 | | | | -0.013 | | | | | | 0.002 | | | | | | 1.2E-10 | | | | | | | 1.68E-04 | | | | 42 | | | |
| rs2994320 | | 1 | | | 243641247 | | | | SDCCAG8 | | | | A | | | | | | | G | | | | 0.804 | | | | 0.017 | | | | | | 0.002 | | | | | | 2.4E-11 | | | | | | | 1.81E-04 | | | | 45 | | | |
| rs62106258 | | 2 | | | 417167 | | | | FAM150B | | | | T | | | | | | | C | | | | 0.951 | | | | 0.067 | | | | | | 0.005 | | | | | | 2E-49 | | | | | | | 8.85E-04 | | | | 218 | | | |
| rs6548237 | | 2 | | | 621461 | | | | TMEM18 | | | | A | | | | | | | C | | | | 0.173 | | | | -0.036 | | | | | | 0.003 | | | | | | 3.4E-44 | | | | | | | 7.88E-04 | | | | 194 | | | |
| rs6749422 | | 2 | | | 25150011 | | | | ADCY3 | | | | C | | | | | | | G | | | | 0.514 | | | | -0.024 | | | | | | 0.002 | | | | | | 1.2E-34 | | | | | | | 6.11E-04 | | | | 151 | | | |
| rs34606703 | | 2 | | | 47014522 | | | | SOCS5 | | | | G | | | | | | | A | | | | 0.642 | | | | 0.012 | | | | | | 0.002 | | | | | | 1.1E-08 | | | | | | | 1.32E-04 | | | | 33 | | | |
| rs13420048 | | 2 | | | 50751414 | | | | NRXN1 | | | | C | | | | | | | A | | | | 0.635 | | | | 0.012 | | | | | | 0.002 | | | | | | 4.4E-09 | | | | | | | 1.40E-04 | | | | 34 | | | |
| rs6545468 | | 2 | | | 55277641 | | | | RTN4 | | | | C | | | | | | | G | | | | 0.587 | | | | 0.012 | | | | | | 0.002 | | | | | | 2.2E-09 | | | | | | | 1.45E-04 | | | | 36 | | | |
| rs4671328 | | 2 | | | 58935282 | | | | FANCL | | | | T | | | | | | | G | | | | 0.448 | | | | 0.014 | | | | | | 0.002 | | | | | | 6.5E-12 | | | | | | | 1.91E-04 | | | | 47 | | | |
| rs13416992 | | 2 | | | 59298298 | | | | FANCL | | | | A | | | | | | | C | | | | 0.396 | | | | 0.015 | | | | | | 0.002 | | | | | | 1.9E-13 | | | | | | | 2.19E-04 | | | | 54 | | | |
| rs10192894 | | 2 | | | 62838936 | | | | EHBP1 | | | | A | | | | | | | G | | | | 0.562 | | | | -0.011 | | | | | | 0.002 | | | | | | 2.5E-08 | | | | | | | 1.26E-04 | | | | 31 | | | |
| rs12477088 | | 2 | | | 67841326 | | | | ETAA1 | | | | T | | | | | | | C | | | | 0.590 | | | | 0.012 | | | | | | 0.002 | | | | | | 2.7E-09 | | | | | | | 1.43E-04 | | | | 35 | | | |
| rs11691869 | | 2 | | | 100805996 | | | | AFF3 | | | | C | | | | | | | A | | | | 0.637 | | | | 0.016 | | | | | | 0.002 | | | | | | 3.3E-14 | | | | | | | 2.33E-04 | | | | 58 | | | |
| rs7602120 | | 2 | | | 144033069 | | | | ARHGAP15 | | | | C | | | | | | | T | | | | 0.534 | | | | -0.013 | | | | | | 0.002 | | | | | | 1.2E-11 | | | | | | | 1.87E-04 | | | | 46 | | | |
| rs1083472 | | 2 | | | 147873492 | | | | ACVR2A | | | | C | | | | | | | G | | | | 0.386 | | | | 0.011 | | | | | | 0.002 | | | | | | 2.1E-08 | | | | | | | 1.27E-04 | | | | 31 | | | |
| rs4482463 | | 2 | | | 205375909 | | | | PARD3B | | | | C | | | | | | | A | | | | 0.077 | | | | 0.022 | | | | | | 0.004 | | | | | | 3.7E-09 | | | | | | | 1.41E-04 | | | | 35 | | | |
| rs4673553 | | 2 | | | 211608379 | | | | CPS1 | | | | T | | | | | | | G | | | | 0.552 | | | | -0.012 | | | | | | 0.002 | | | | | | 3.2E-09 | | | | | | | 1.42E-04 | | | | 35 | | | |
| rs2433733 | | 2 | | | 230816703 | | | | FBXO36 | | | | G | | | | | | | A | | | | 0.322 | | | | 0.012 | | | | | | 0.002 | | | | | | 2.3E-08 | | | | | | | 1.27E-04 | | | | 31 | | | |
| rs113706999 | | 3 | | | 44159156 | | | | TOPAZ1 | | | | T | | | | | | | A | | | | 0.976 | | | | -0.039 | | | | | | 0.007 | | | | | | 1E-08 | | | | | | | 1.33E-04 | | | | 33 | | | |
| rs72906474 | | 3 | | | 47817007 | | | | SMARCC1 | | | | G | | | | | | | T | | | | 0.416 | | | | 0.012 | | | | | | 0.002 | | | | | | 1.1E-08 | | | | | | | 1.33E-04 | | | | 33 | | | |
| rs9843653 | | 3 | | | 49920571 | | | | MST1R | | | | T | | | | | | | C | | | | 0.490 | | | | -0.018 | | | | | | 0.002 | | | | | | 4.2E-19 | | | | | | | 3.24E-04 | | | | 80 | | | |
| rs6774533 | | 3 | | | 62471086 | | | | CADPS | | | | C | | | | | | | T | | | | 0.296 | | | | -0.012 | | | | | | 0.002 | | | | | | 2.8E-08 | | | | | | | 1.25E-04 | | | | 31 | | | |
| rs13066308 | | 3 | | | 82713150 | | | | GBE1 | | | | C | | | | | | | G | | | | 0.639 | | | | -0.012 | | | | | | 0.002 | | | | | | 3.7E-09 | | | | | | | 1.41E-04 | | | | 35 | | | |
| rs1454687 | | 3 | | | 94038085 | | | | NSUN3 | | | | C | | | | | | | G | | | | 0.484 | | | | 0.012 | | | | | | 0.002 | | | | | | 4.1E-10 | | | | | | | 1.59E-04 | | | | 39 | | | |
| rs13081671 | | 3 | | | 135876549 | | | | MSL2 | | | | C | | | | | | | T | | | | 0.728 | | | | -0.013 | | | | | | 0.002 | | | | | | 1.2E-09 | | | | | | | 1.50E-04 | | | | 37 | | | |
| rs2035936 | | 3 | | | 141298124 | | | | RASA2 | | | | G | | | | | | | T | | | | 0.944 | | | | -0.027 | | | | | | 0.004 | | | | | | 4.4E-10 | | | | | | | 1.58E-04 | | | | 39 | | | |
| rs529200 | | 3 | | | 173114305 | | | | NLGN1 | | | | A | | | | | | | G | | | | 0.473 | | | | -0.012 | | | | | | 0.002 | | | | | | 4E-09 | | | | | | | 1.40E-04 | | | | 35 | | | |
| rs73052033 | | 3 | | | 185828465 | | | | DGKG | | | | T | | | | | | | C | | | | 0.815 | | | | 0.017 | | | | | | 0.003 | | | | | | 1.1E-11 | | | | | | | 1.87E-04 | | | | 46 | | | |
| rs61218008 | | 3 | | | 194881130 | | | | XXYLT1 | | | | A | | | | | | | G | | | | 0.721 | | | | 0.012 | | | | | | 0.002 | | | | | | 4.7E-08 | | | | | | | 1.21E-04 | | | | 30 | | | |
| rs2643450 | | 4 | | | 18547417 | | | | LCORL | | | | A | | | | | | | G | | | | 0.459 | | | | -0.011 | | | | | | 0.002 | | | | | | 4.5E-08 | | | | | | | 1.21E-04 | | | | 30 | | | |
| rs9684942 | | 4 | | | 20233035 | | | | SLIT2 | | | | G | | | | | | | A | | | | 0.853 | | | | -0.015 | | | | | | 0.003 | | | | | | 3.8E-08 | | | | | | | 1.23E-04 | | | | 30 | | | |
| rs34811474 | | 4 | | | 25408838 | | | | ANAPC4 | | | | G | | | | | | | A | | | | 0.770 | | | | 0.020 | | | | | | 0.002 | | | | | | 3.2E-17 | | | | | | | 2.89E-04 | | | | 71 | | | |
| rs73213484 | | 4 | | | 28489339 | | | | RP11-180C1.1 | | | | A | | | | | | | T | | | | 0.858 | | | | 0.019 | | | | | | 0.003 | | | | | | 2.3E-11 | | | | | | | 1.81E-04 | | | | 45 | | | |
| rs4527444 | | 4 | | | 30842780 | | | | PCDH7 | | | | A | | | | | | | G | | | | 0.458 | | | | -0.012 | | | | | | 0.002 | | | | | | 3.3E-10 | | | | | | | 1.60E-04 | | | | 39 | | | |
| rs12641981 | | 4 | | | 45179883 | | | | GNPDA2 | | | | C | | | | | | | T | | | | 0.566 | | | | -0.017 | | | | | | 0.002 | | | | | | 2.8E-17 | | | | | | | 2.90E-04 | | | | 71 | | | |
| rs148712344 | | 4 | | | 55476318 | | | | KIT | | | | G | | | | | | | T | | | | 0.960 | | | | 0.030 | | | | | | 0.005 | | | | | | 5.1E-09 | | | | | | | 1.38E-04 | | | | 34 | | | |
| rs925422 | | 4 | | | 60254101 | | | | LPHN3 | | | | T | | | | | | | G | | | | 0.257 | | | | 0.013 | | | | | | 0.002 | | | | | | 2.7E-08 | | | | | | | 1.25E-04 | | | | 31 | | | |
| rs1603179 | | 4 | | | 67805347 | | | | CENPC | | | | A | | | | | | | C | | | | 0.356 | | | | 0.011 | | | | | | 0.002 | | | | | | 2.9E-08 | | | | | | | 1.25E-04 | | | | 31 | | | |
| rs11098965 | | 4 | | | 80888040 | | | | ANTXR2 | | | | C | | | | | | | T | | | | 0.293 | | | | 0.014 | | | | | | 0.002 | | | | | | 1.6E-09 | | | | | | | 1.48E-04 | | | | 36 | | | |
| rs4148155 | | 4 | | | 89054667 | | | | ABCG2 | | | | A | | | | | | | G | | | | 0.887 | | | | 0.018 | | | | | | 0.003 | | | | | | 3.8E-09 | | | | | | | 1.41E-04 | | | | 35 | | | |
| rs13107325 | | 4 | | | 103188709 | | | | SLC39A8 | | | | C | | | | | | | T | | | | 0.926 | | | | -0.029 | | | | | | 0.004 | | | | | | 1.9E-14 | | | | | | | 2.38E-04 | | | | 59 | | | |
| rs769668 | | 4 | | | 140858717 | | | | MAML3 | | | | T | | | | | | | C | | | | 0.653 | | | | 0.014 | | | | | | 0.002 | | | | | | 1.8E-11 | | | | | | | 1.83E-04 | | | | 45 | | | |
| rs35390852 | | 4 | | | 143067054 | | | | INPP4B | | | | G | | | | | | | A | | | | 0.876 | | | | -0.017 | | | | | | 0.003 | | | | | | 2.4E-08 | | | | | | | 1.26E-04 | | | | 31 | | | |
| rs828550 | | 5 | | | 3539923 | | | | IRX1 | | | | C | | | | | | | T | | | | 0.355 | | | | -0.012 | | | | | | 0.002 | | | | | | 1.5E-08 | | | | | | | 1.30E-04 | | | | 32 | | | |
| rs10514963 | | 5 | | | 63027870 | | | | HTR1A | | | | G | | | | | | | A | | | | 0.519 | | | | -0.014 | | | | | | 0.002 | | | | | | 3E-12 | | | | | | | 1.97E-04 | | | | 49 | | | |
| rs34341 | | 5 | | | 74934009 | | | | ANKDD1B | | | | A | | | | | | | T | | | | 0.425 | | | | -0.020 | | | | | | 0.002 | | | | | | 1.1E-23 | | | | | | | 4.08E-04 | | | | 101 | | | |
| rs59893724 | | 5 | | | 80830788 | | | | SSBP2 | | | | A | | | | | | | G | | | | 0.756 | | | | 0.013 | | | | | | 0.002 | | | | | | 2.4E-08 | | | | | | | 1.26E-04 | | | | 31 | | | |
| rs7442885 | | 5 | | | 87682877 | | | | TMEM161B | | | | C | | | | | | | G | | | | 0.786 | | | | 0.017 | | | | | | 0.002 | | | | | | 1.5E-12 | | | | | | | 2.03E-04 | | | | 50 | | | |
| rs1477290 | | 5 | | | 87988934 | | | | MEF2C | | | | T | | | | | | | C | | | | 0.863 | | | | -0.017 | | | | | | 0.003 | | | | | | 1.4E-09 | | | | | | | 1.49E-04 | | | | 37 | | | |
| rs10038055 | | 5 | | | 88783421 | | | | MEF2C | | | | G | | | | | | | T | | | | 0.632 | | | | -0.012 | | | | | | 0.002 | | | | | | 8.3E-09 | | | | | | | 1.35E-04 | | | | 33 | | | |
| rs288187 | | 5 | | | 107344426 | | | | FBXL17 | | | | C | | | | | | | T | | | | 0.827 | | | | 0.020 | | | | | | 0.003 | | | | | | 4E-14 | | | | | | | 2.32E-04 | | | | 57 | | | |
| rs1366334 | | 5 | | | 122683163 | | | | CEP120 | | | | C | | | | | | | G | | | | 0.293 | | | | 0.012 | | | | | | 0.002 | | | | | | 4.7E-08 | | | | | | | 1.21E-04 | | | | 30 | | | |
| rs13174863 | | 5 | | | 139080745 | | | | CXXC5 | | | | A | | | | | | | G | | | | 0.852 | | | | -0.019 | | | | | | 0.003 | | | | | | 1.8E-11 | | | | | | | 1.83E-04 | | | | 45 | | | |
| rs251353 | | 5 | | | 140228164 | | | | PCDHA1 | | | | C | | | | | | | A | | | | 0.471 | | | | 0.012 | | | | | | 0.002 | | | | | | 1.1E-08 | | | | | | | 1.33E-04 | | | | 33 | | | |
| rs11134679 | | 5 | | | 170623391 | | | | RANBP17 | | | | A | | | | | | | G | | | | 0.315 | | | | -0.013 | | | | | | 0.002 | | | | | | 8E-10 | | | | | | | 1.53E-04 | | | | 38 | | | |
| rs9395520 | | 6 | | | 13183523 | | | | PHACTR1 | | | | C | | | | | | | T | | | | 0.695 | | | | 0.013 | | | | | | 0.002 | | | | | | 4.6E-09 | | | | | | | 1.39E-04 | | | | 34 | | | |
| rs35778344 | | 6 | | | 28599105 | | | | SCAND3 | | | | G | | | | | | | A | | | | 0.803 | | | | -0.014 | | | | | | 0.002 | | | | | | 1.8E-08 | | | | | | | 1.29E-04 | | | | 32 | | | |
| rs3130048 | | 6 | | | 31613739 | | | | BAG6 | | | | T | | | | | | | C | | | | 0.720 | | | | -0.016 | | | | | | 0.002 | | | | | | 4.6E-13 | | | | | | | 2.12E-04 | | | | 52 | | | |
| rs34298980 | | 6 | | | 40409243 | | | | LRFN2 | | | | T | | | | | | | C | | | | 0.492 | | | | 0.014 | | | | | | 0.002 | | | | | | 8.8E-12 | | | | | | | 1.89E-04 | | | | 47 | | | |
| rs72892910 | | 6 | | | 50816887 | | | | TFAP2B | | | | G | | | | | | | T | | | | 0.828 | | | | -0.026 | | | | | | 0.003 | | | | | | 9.7E-24 | | | | | | | 4.09E-04 | | | | 101 | | | |
| rs1547026 | | 6 | | | 51825622 | | | | PKHD1 | | | | T | | | | | | | C | | | | 0.709 | | | | -0.012 | | | | | | 0.002 | | | | | | 1.6E-08 | | | | | | | 1.29E-04 | | | | 32 | | | |
| rs2253310 | | 6 | | | 108888593 | | | | FOXO3 | | | | C | | | | | | | G | | | | 0.374 | | | | -0.012 | | | | | | 0.002 | | | | | | 6.1E-09 | | | | | | | 1.37E-04 | | | | 34 | | | |
| rs9387640 | | 6 | | | 119508871 | | | | MAN1A1 | | | | C | | | | | | | T | | | | 0.637 | | | | 0.012 | | | | | | 0.002 | | | | | | 9.2E-09 | | | | | | | 1.34E-04 | | | | 33 | | | |
| rs73046311 | | 7 | | | 1854159 | | | | MAD1L1 | | | | C | | | | | | | G | | | | 0.839 | | | | 0.016 | | | | | | 0.003 | | | | | | 5.7E-09 | | | | | | | 1.38E-04 | | | | 34 | | | |
| rs2866720 | | 7 | | | 70106310 | | | | AUTS2 | | | | C | | | | | | | T | | | | 0.615 | | | | -0.012 | | | | | | 0.002 | | | | | | 7E-09 | | | | | | | 1.36E-04 | | | | 34 | | | |
| rs11976018 | | 7 | | | 99122437 | | | | ZKSCAN5 | | | | G | | | | | | | A | | | | 0.846 | | | | 0.015 | | | | | | 0.003 | | | | | | 2.8E-08 | | | | | | | 1.25E-04 | | | | 31 | | | |
| rs12375196 | | 7 | | | 103416541 | | | | RELN | | | | C | | | | | | | A | | | | 0.576 | | | | -0.012 | | | | | | 0.002 | | | | | | 5.5E-09 | | | | | | | 1.38E-04 | | | | 34 | | | |
| rs2396625 | | 7 | | | 113028634 | | | | TSRM | | | | T | | | | | | | A | | | | 0.578 | | | | 0.012 | | | | | | 0.002 | | | | | | 3.4E-09 | | | | | | | 1.42E-04 | | | | 35 | | | |
| rs1840661 | | 7 | | | 114352682 | | | | FOXP2 | | | | T | | | | | | | A | | | | 0.575 | | | | -0.012 | | | | | | 0.002 | | | | | | 1.2E-09 | | | | | | | 1.50E-04 | | | | 37 | | | |
| rs7853 | | 8 | | | 8890814 | | | | ERI1 | | | | A | | | | | | | G | | | | 0.553 | | | | -0.012 | | | | | | 0.002 | | | | | | 1.3E-09 | | | | | | | 1.50E-04 | | | | 37 | | | |
| rs11250094 | | 8 | | | 10802001 | | | | XKR6 | | | | G | | | | | | | C | | | | 0.453 | | | | 0.012 | | | | | | 0.002 | | | | | | 1.1E-09 | | | | | | | 1.51E-04 | | | | 37 | | | |
| rs6557829 | | 8 | | | 21973970 | | | | HR | | | | C | | | | | | | A | | | | 0.595 | | | | -0.012 | | | | | | 0.002 | | | | | | 6.9E-09 | | | | | | | 1.36E-04 | | | | 34 | | | |
| rs117176448 | | 8 | | | 27261138 | | | | PTK2B | | | | C | | | | | | | G | | | | 0.904 | | | | -0.019 | | | | | | 0.003 | | | | | | 2E-08 | | | | | | | 1.28E-04 | | | | 32 | | | |
| rs10957605 | | 8 | | | 73433886 | | | | KCNB2 | | | | C | | | | | | | T | | | | 0.318 | | | | 0.016 | | | | | | 0.002 | | | | | | 9.8E-14 | | | | | | | 2.25E-04 | | | | 55 | | | |
| rs17716502 | | 8 | | | 116659731 | | | | TRPS1 | | | | C | | | | | | | T | | | | 0.795 | | | | 0.018 | | | | | | 0.002 | | | | | | 3.5E-13 | | | | | | | 2.15E-04 | | | | 53 | | | |
| rs4740442 | | 9 | | | 10153245 | | | | PTPRD | | | | C | | | | | | | T | | | | 0.715 | | | | -0.012 | | | | | | 0.002 | | | | | | 2.6E-08 | | | | | | | 1.26E-04 | | | | 31 | | | |
| rs13292699 | | 9 | | | 15910044 | | | | CCDC171 | | | | A | | | | | | | C | | | | 0.567 | | | | 0.015 | | | | | | 0.002 | | | | | | 4.3E-14 | | | | | | | 2.31E-04 | | | | 57 | | | |
| rs17770336 | | 9 | | | 28414625 | | | | LINGO2 | | | | C | | | | | | | T | | | | 0.676 | | | | -0.016 | | | | | | 0.002 | | | | | | 2.5E-14 | | | | | | | 2.36E-04 | | | | 58 | | | |
| rs2398851 | | 9 | | | 96398508 | | | | PHF2 | | | | A | | | | | | | G | | | | 0.320 | | | | 0.012 | | | | | | 0.002 | | | | | | 8E-09 | | | | | | | 1.35E-04 | | | | 33 | | | |
| rs7047694 | | 9 | | | 103141037 | | | | TEX10 | | | | G | | | | | | | A | | | | 0.681 | | | | -0.012 | | | | | | 0.002 | | | | | | 3.7E-09 | | | | | | | 1.41E-04 | | | | 35 | | | |
| rs6478538 | | 9 | | | 124627012 | | | | TTLL11 | | | | A | | | | | | | G | | | | 0.323 | | | | 0.012 | | | | | | 0.002 | | | | | | 4.6E-08 | | | | | | | 1.21E-04 | | | | 30 | | | |
| rs777676 | | 9 | | | 129691913 | | | | RALGPS1 | | | | T | | | | | | | A | | | | 0.511 | | | | 0.011 | | | | | | 0.002 | | | | | | 3.7E-08 | | | | | | | 1.23E-04 | | | | 30 | | | |
| rs3003578 | | 9 | | | 130994179 | | | | DNM1 | | | | C | | | | | | | T | | | | 0.441 | | | | 0.011 | | | | | | 0.002 | | | | | | 1.8E-08 | | | | | | | 1.29E-04 | | | | 32 | | | |
| rs1270799 | | 10 | | | 21907423 | | | | MLLT10 | | | | T | | | | | | | G | | | | 0.696 | | | | -0.017 | | | | | | 0.002 | | | | | | 1.2E-14 | | | | | | | 2.42E-04 | | | | 60 | | | |
| rs113585475 | | 10 | | | 33985434 | | | | NRP1 | | | | C | | | | | | | T | | | | 0.900 | | | | 0.022 | | | | | | 0.003 | | | | | | 3.6E-11 | | | | | | | 1.78E-04 | | | | 44 | | | |
| rs3125326 | | 10 | | | 63053788 | | | | TMEM26 | | | | A | | | | | | | C | | | | 0.391 | | | | -0.011 | | | | | | 0.002 | | | | | | 4.7E-08 | | | | | | | 1.21E-04 | | | | 30 | | | |
| rs7090758 | | 10 | | | 65335315 | | | | REEP3 | | | | T | | | | | | | C | | | | 0.526 | | | | -0.013 | | | | | | 0.002 | | | | | | 5.7E-11 | | | | | | | 1.74E-04 | | | | 43 | | | |
| rs11000942 | | 10 | | | 76025256 | | | | ADK | | | | G | | | | | | | A | | | | 0.876 | | | | -0.016 | | | | | | 0.003 | | | | | | 4E-08 | | | | | | | 1.22E-04 | | | | 30 | | | |
| rs1250535 | | 10 | | | 81016112 | | | | ZMIZ1 | | | | C | | | | | | | G | | | | 0.325 | | | | -0.012 | | | | | | 0.002 | | | | | | 1.8E-08 | | | | | | | 1.28E-04 | | | | 32 | | | |
| rs17399739 | | 10 | | | 87490850 | | | | GRID1 | | | | A | | | | | | | G | | | | 0.931 | | | | -0.023 | | | | | | 0.004 | | | | | | 6.1E-09 | | | | | | | 1.37E-04 | | | | 34 | | | |
| rs10510025 | | 10 | | | 118650996 | | | | KIAA1598 | | | | C | | | | | | | T | | | | 0.753 | | | | -0.013 | | | | | | 0.002 | | | | | | 6.9E-09 | | | | | | | 1.36E-04 | | | | 34 | | | |
| rs4962725 | | 10 | | | 126733321 | | | | CTBP2 | | | | T | | | | | | | C | | | | 0.572 | | | | -0.011 | | | | | | 0.002 | | | | | | 2E-08 | | | | | | | 1.28E-04 | | | | 31 | | | |
| rs11146233 | | 10 | | | 134000962 | | | | DPYSL4 | | | | G | | | | | | | A | | | | 0.439 | | | | 0.011 | | | | | | 0.002 | | | | | | 9.4E-09 | | | | | | | 1.34E-04 | | | | 33 | | | |
| rs7950166 | | 11 | | | 8642218 | | | | TRIM66 | | | | C | | | | | | | T | | | | 0.354 | | | | -0.014 | | | | | | 0.002 | | | | | | 3E-12 | | | | | | | 1.97E-04 | | | | 49 | | | |
| rs11022766 | | 11 | | | 13348249 | | | | ARNTL | | | | T | | | | | | | G | | | | 0.641 | | | | 0.014 | | | | | | 0.002 | | | | | | 2.5E-11 | | | | | | | 1.81E-04 | | | | 45 | | | |
| rs1013402 | | 11 | | | 27712381 | | | | BDNF | | | | A | | | | | | | G | | | | 0.681 | | | | -0.021 | | | | | | 0.002 | | | | | | 6E-24 | | | | | | | 4.13E-04 | | | | 102 | | | |
| rs34292685 | | 11 | | | 64049021 | | | | GPR137 | | | | C | | | | | | | T | | | | 0.839 | | | | 0.018 | | | | | | 0.003 | | | | | | 1.6E-11 | | | | | | | 1.84E-04 | | | | 45 | | | |
| rs10896012 | | 11 | | | 65278461 | | | | SCYL1 | | | | T | | | | | | | C | | | | 0.784 | | | | -0.016 | | | | | | 0.002 | | | | | | 3.7E-11 | | | | | | | 1.77E-04 | | | | 44 | | | |
| rs3802858 | | 11 | | | 115078492 | | | | CADM1 | | | | T | | | | | | | C | | | | 0.573 | | | | 0.012 | | | | | | 0.002 | | | | | | 1.1E-09 | | | | | | | 1.51E-04 | | | | 37 | | | |
| rs11218510 | | 11 | | | 121922587 | | | | BLID | | | | G | | | | | | | A | | | | 0.599 | | | | 0.011 | | | | | | 0.002 | | | | | | 2.9E-08 | | | | | | | 1.25E-04 | | | | 31 | | | |
| rs2512884 | | 11 | | | 131467856 | | | | NTM | | | | C | | | | | | | A | | | | 0.484 | | | | -0.012 | | | | | | 0.002 | | | | | | 3.3E-10 | | | | | | | 1.60E-04 | | | | 40 | | | |
| rs11223204 | | 11 | | | 132652554 | | | | OPCML | | | | A | | | | | | | G | | | | 0.565 | | | | -0.011 | | | | | | 0.002 | | | | | | 1.1E-08 | | | | | | | 1.32E-04 | | | | 33 | | | |
| rs12364470 | | 11 | | | 134601012 | | | | AP003062.1 | | | | T | | | | | | | G | | | | 0.836 | | | | -0.015 | | | | | | 0.003 | | | | | | 2.9E-08 | | | | | | | 1.25E-04 | | | | 31 | | | |
| rs55726687 | | 12 | | | 991306 | | | | WNK1 | | | | G | | | | | | | A | | | | 0.789 | | | | -0.015 | | | | | | 0.002 | | | | | | 1.3E-09 | | | | | | | 1.49E-04 | | | | 37 | | | |
| rs7976757 | | 12 | | | 19207948 | | | | PLEKHA5 | | | | T | | | | | | | C | | | | 0.173 | | | | -0.015 | | | | | | 0.003 | | | | | | 6.1E-09 | | | | | | | 1.37E-04 | | | | 34 | | | |
| rs7132908 | | 12 | | | 50263148 | | | | FAIM2 | | | | G | | | | | | | A | | | | 0.616 | | | | -0.019 | | | | | | 0.002 | | | | | | 8.2E-21 | | | | | | | 3.55E-04 | | | | 88 | | | |
| rs2292238 | | 12 | | | 56493822 | | | | ERBB3 | | | | A | | | | | | | C | | | | 0.593 | | | | 0.012 | | | | | | 0.002 | | | | | | 1.3E-09 | | | | | | | 1.50E-04 | | | | 37 | | | |
| rs770082 | | 12 | | | 89776485 | | | | DUSP6 | | | | G | | | | | | | A | | | | 0.563 | | | | -0.013 | | | | | | 0.002 | | | | | | 2.1E-11 | | | | | | | 1.82E-04 | | | | 45 | | | |
| rs10849900 | | 12 | | | 110974890 | | | | PPTC7 | | | | T | | | | | | | C | | | | 0.690 | | | | 0.012 | | | | | | 0.002 | | | | | | 1.6E-08 | | | | | | | 1.29E-04 | | | | 32 | | | |
| rs111828690 | | 12 | | | 117576767 | | | | FBXO21 | | | | C | | | | | | | T | | | | 0.779 | | | | -0.014 | | | | | | 0.002 | | | | | | 1E-08 | | | | | | | 1.33E-04 | | | | 33 | | | |
| rs181617194 | | 12 | | | 122011598 | | | | KDM2B | | | | T | | | | | | | C | | | | 0.959 | | | | 0.032 | | | | | | 0.005 | | | | | | 4.2E-09 | | | | | | | 1.40E-04 | | | | 35 | | | |
| rs3803005 | | 12 | | | 123110654 | | | | KNTC1 | | | | T | | | | | | | C | | | | 0.270 | | | | -0.017 | | | | | | 0.002 | | | | | | 3.6E-14 | | | | | | | 2.33E-04 | | | | 57 | | | |
| rs9579775 | | 13 | | | 20616557 | | | | ZMYM2 | | | | A | | | | | | | C | | | | 0.864 | | | | -0.019 | | | | | | 0.003 | | | | | | 1.4E-10 | | | | | | | 1.67E-04 | | | | 41 | | | |
| rs1933440 | | 13 | | | 28676971 | | | | FLT3 | | | | A | | | | | | | C | | | | 0.837 | | | | -0.015 | | | | | | 0.003 | | | | | | 1.2E-08 | | | | | | | 1.32E-04 | | | | 33 | | | |
| rs2761366 | | 13 | | | 33011872 | | | | N4BP2L2 | | | | C | | | | | | | T | | | | 0.358 | | | | -0.011 | | | | | | 0.002 | | | | | | 3.2E-08 | | | | | | | 1.24E-04 | | | | 31 | | | |
| rs9568867 | | 13 | | | 54107352 | | | | OLFM4 | | | | G | | | | | | | A | | | | 0.871 | | | | -0.019 | | | | | | 0.003 | | | | | | 6.8E-11 | | | | | | | 1.73E-04 | | | | 43 | | | |
| rs12866691 | | 13 | | | 58623783 | | | | PCDH17 | | | | A | | | | | | | T | | | | 0.773 | | | | 0.016 | | | | | | 0.002 | | | | | | 3.9E-11 | | | | | | | 1.77E-04 | | | | 44 | | | |
| rs1576655 | | 13 | | | 79587841 | | | | RBM26 | | | | A | | | | | | | C | | | | 0.404 | | | | -0.012 | | | | | | 0.002 | | | | | | 2.4E-09 | | | | | | | 1.45E-04 | | | | 36 | | | |
| rs7331420 | | 13 | | | 99236471 | | | | STK24 | | | | G | | | | | | | A | | | | 0.715 | | | | 0.012 | | | | | | 0.002 | | | | | | 4.8E-08 | | | | | | | 1.21E-04 | | | | 30 | | | |
| rs9522180 | | 13 | | | 111970212 | | | | TEX29 | | | | C | | | | | | | T | | | | 0.447 | | | | 0.011 | | | | | | 0.002 | | | | | | 4.4E-08 | | | | | | | 1.21E-04 | | | | 30 | | | |
| rs10142359 | | 14 | | | 73884540 | | | | NUMB | | | | A | | | | | | | G | | | | 0.518 | | | | -0.011 | | | | | | 0.002 | | | | | | 1.7E-08 | | | | | | | 1.29E-04 | | | | 32 | | | |
| rs8022132 | | 14 | | | 79955864 | | | | NRXN3 | | | | A | | | | | | | T | | | | 0.697 | | | | -0.015 | | | | | | 0.002 | | | | | | 1E-11 | | | | | | | 1.88E-04 | | | | 46 | | | |
| rs6575340 | | 14 | | | 94023972 | | | | UNC79 | | | | G | | | | | | | A | | | | 0.365 | | | | -0.015 | | | | | | 0.002 | | | | | | 1.1E-12 | | | | | | | 2.05E-04 | | | | 51 | | | |
| rs7145882 | | 14 | | | 103255461 | | | | TRAF3 | | | | T | | | | | | | C | | | | 0.342 | | | | 0.012 | | | | | | 0.002 | | | | | | 6E-09 | | | | | | | 1.37E-04 | | | | 34 | | | |
| rs12891477 | | 14 | | | 104332759 | | | | PPP1R13B | | | | C | | | | | | | T | | | | 0.631 | | | | -0.013 | | | | | | 0.002 | | | | | | 1.8E-10 | | | | | | | 1.65E-04 | | | | 41 | | | |
| rs1466276 | | 15 | | | 52025950 | | | | LYSMD2 | | | | C | | | | | | | G | | | | 0.524 | | | | 0.011 | | | | | | 0.002 | | | | | | 1.9E-08 | | | | | | | 1.28E-04 | | | | 32 | | | |
| rs4776985 | | 15 | | | 68123021 | | | | SKOR1 | | | | T | | | | | | | G | | | | 0.774 | | | | 0.020 | | | | | | 0.002 | | | | | | 8.9E-17 | | | | | | | 2.81E-04 | | | | 69 | | | |
| rs67962220 | | 15 | | | 74188926 | | | | TBC1D21 | | | | T | | | | | | | G | | | | 0.831 | | | | -0.015 | | | | | | 0.003 | | | | | | 9.8E-09 | | | | | | | 1.33E-04 | | | | 33 | | | |
| rs715724 | | 15 | | | 80984293 | | | | ABHD17C | | | | A | | | | | | | G | | | | 0.649 | | | | 0.012 | | | | | | 0.002 | | | | | | 1.4E-08 | | | | | | | 1.30E-04 | | | | 32 | | | |
| rs939624 | | 15 | | | 99480551 | | | | IGF1R | | | | C | | | | | | | T | | | | 0.538 | | | | -0.012 | | | | | | 0.002 | | | | | | 4.7E-10 | | | | | | | 1.57E-04 | | | | 39 | | | |
| rs7200589 | | 16 | | | 349331 | | | | AXIN1 | | | | G | | | | | | | A | | | | 0.727 | | | | 0.016 | | | | | | 0.002 | | | | | | 2E-12 | | | | | | | 2.01E-04 | | | | 49 | | | |
| rs879620 | | 16 | | | 4015729 | | | | ADCY9 | | | | C | | | | | | | T | | | | 0.387 | | | | -0.017 | | | | | | 0.002 | | | | | | 9.7E-18 | | | | | | | 2.98E-04 | | | | 74 | | | |
| rs57790054 | | 16 | | | 20006986 | | | | GPR139 | | | | A | | | | | | | G | | | | 0.739 | | | | -0.012 | | | | | | 0.002 | | | | | | 3.7E-08 | | | | | | | 1.23E-04 | | | | 30 | | | |
| rs11074452 | | 16 | | | 20370168 | | | | PDILT | | | | C | | | | | | | G | | | | 0.492 | | | | 0.015 | | | | | | 0.002 | | | | | | 8.3E-14 | | | | | | | 2.26E-04 | | | | 56 | | | |
| rs62031562 | | 16 | | | 28609329 | | | | SULT1A2 | | | | A | | | | | | | T | | | | 0.626 | | | | -0.018 | | | | | | 0.002 | | | | | | 1.3E-19 | | | | | | | 3.33E-04 | | | | 82 | | | |
| rs3814883 | | 16 | | | 29994922 | | | | TAOK2 | | | | C | | | | | | | T | | | | 0.517 | | | | -0.015 | | | | | | 0.002 | | | | | | 3.5E-14 | | | | | | | 2.33E-04 | | | | 57 | | | |
| rs34898535 | | 16 | | | 31025641 | | | | STX1B | | | | C | | | | | | | T | | | | 0.623 | | | | 0.019 | | | | | | 0.002 | | | | | | 2.8E-21 | | | | | | | 3.64E-04 | | | | 90 | | | |
| rs56094641 | | 16 | | | 53806453 | | | | FTO | | | | A | | | | | | | G | | | | 0.597 | | | | -0.046 | | | | | | 0.002 | | | | | | 4.3E-114 | | | | | | | 2.09E-03 | | | | 515 | | | |
| rs3751859 | | 16 | | | 81735012 | | | | CMIP | | | | G | | | | | | | A | | | | 0.843 | | | | 0.017 | | | | | | 0.003 | | | | | | 2.4E-10 | | | | | | | 1.63E-04 | | | | 40 | | | |
| rs4790292 | | 17 | | | 1824305 | | | | RTN4RL1 | | | | C | | | | | | | A | | | | 0.846 | | | | 0.017 | | | | | | 0.003 | | | | | | 1.8E-10 | | | | | | | 1.65E-04 | | | | 41 | | | |
| rs1914889 | | 17 | | | 21267590 | | | | KCNJ12 | | | | G | | | | | | | A | | | | 0.547 | | | | 0.012 | | | | | | 0.002 | | | | | | 7.8E-10 | | | | | | | 1.53E-04 | | | | 38 | | | |
| rs2306593 | | 17 | | | 34866546 | | | | MYO19 | | | | C | | | | | | | T | | | | 0.510 | | | | 0.011 | | | | | | 0.002 | | | | | | 7.2E-09 | | | | | | | 1.36E-04 | | | | 33 | | | |
| rs11079849 | | 17 | | | 47090785 | | | | IGF2BP1 | | | | C | | | | | | | T | | | | 0.671 | | | | 0.013 | | | | | | 0.002 | | | | | | 1.6E-09 | | | | | | | 1.48E-04 | | | | 36 | | | |
| rs77706698 | | 17 | | | 65953348 | | | | BPTF | | | | G | | | | | | | A | | | | 0.868 | | | | -0.017 | | | | | | 0.003 | | | | | | 4.2E-09 | | | | | | | 1.40E-04 | | | | 35 | | | |
| rs2619976 | | 17 | | | 71754545 | | | | SDK2 | | | | C | | | | | | | T | | | | 0.587 | | | | -0.012 | | | | | | 0.002 | | | | | | 5.4E-09 | | | | | | | 1.38E-04 | | | | 34 | | | |
| rs11150745 | | 17 | | | 78757626 | | | | RPTOR | | | | A | | | | | | | G | | | | 0.682 | | | | 0.014 | | | | | | 0.002 | | | | | | 1.5E-11 | | | | | | | 1.85E-04 | | | | 46 | | | |
| rs891386 | | 18 | | | 21103971 | | | | NPC1 | | | | T | | | | | | | G | | | | 0.542 | | | | 0.012 | | | | | | 0.002 | | | | | | 3.4E-09 | | | | | | | 1.42E-04 | | | | 35 | | | |
| rs11660335 | | 18 | | | 22154235 | | | | HRH4 | | | | T | | | | | | | C | | | | 0.810 | | | | 0.016 | | | | | | 0.003 | | | | | | 2.5E-10 | | | | | | | 1.62E-04 | | | | 40 | | | |
| rs784257 | | 18 | | | 53397199 | | | | TCF4 | | | | T | | | | | | | C | | | | 0.188 | | | | -0.014 | | | | | | 0.003 | | | | | | 2.9E-08 | | | | | | | 1.25E-04 | | | | 31 | | | |
| rs66922415 | | 18 | | | 57848651 | | | | MC4R | | | | A | | | | | | | G | | | | 0.767 | | | | -0.039 | | | | | | 0.002 | | | | | | 7.4E-62 | | | | | | | 1.12E-03 | | | | 275 | | | |
| rs17066856 | | 18 | | | 58049656 | | | | MC4R | | | | T | | | | | | | C | | | | 0.909 | | | | 0.028 | | | | | | 0.003 | | | | | | 2.9E-16 | | | | | | | 2.71E-04 | | | | 67 | | | |
| rs9962947 | | 18 | | | 72903636 | | | | ZADH2 | | | | C | | | | | | | T | | | | 0.362 | | | | -0.011 | | | | | | 0.002 | | | | | | 4.6E-08 | | | | | | | 1.21E-04 | | | | 30 | | | |
| rs12974664 | | 19 | | | 1856186 | | | | KLF16 | | | | G | | | | | | | A | | | | 0.464 | | | | -0.012 | | | | | | 0.002 | | | | | | 6E-10 | | | | | | | 1.55E-04 | | | | 38 | | | |
| rs350832 | | 19 | | | 4069426 | | | | ZBTB7A | | | | G | | | | | | | A | | | | 0.229 | | | | -0.014 | | | | | | 0.002 | | | | | | 6.5E-09 | | | | | | | 1.37E-04 | | | | 34 | | | |
| rs12986231 | | 19 | | | 18469017 | | | | PGPEP1 | | | | T | | | | | | | C | | | | 0.732 | | | | 0.015 | | | | | | 0.002 | | | | | | 6.2E-11 | | | | | | | 1.73E-04 | | | | 43 | | | |
| rs10404726 | | 19 | | | 18834514 | | | | CRTC1 | | | | C | | | | | | | T | | | | 0.534 | | | | 0.014 | | | | | | 0.002 | | | | | | 2.5E-12 | | | | | | | 1.99E-04 | | | | 49 | | | |
| rs56212061 | | 19 | | | 19394640 | | | | SUGP1 | | | | C | | | | | | | T | | | | 0.849 | | | | 0.015 | | | | | | 0.003 | | | | | | 3.9E-08 | | | | | | | 1.23E-04 | | | | 30 | | | |
| rs111640872 | | 19 | | | 30290357 | | | | CCNE1 | | | | G | | | | | | | C | | | | 0.669 | | | | -0.013 | | | | | | 0.002 | | | | | | 1.8E-09 | | | | | | | 1.47E-04 | | | | 36 | | | |
| rs11880064 | | 19 | | | 33964181 | | | | PEPD | | | | T | | | | | | | C | | | | 0.636 | | | | -0.011 | | | | | | 0.002 | | | | | | 4.8E-08 | | | | | | | 1.21E-04 | | | | 30 | | | |
| rs429358 | | 19 | | | 45411941 | | | | APOE | | | | T | | | | | | | C | | | | 0.846 | | | | 0.018 | | | | | | 0.003 | | | | | | 1.4E-10 | | | | | | | 1.67E-04 | | | | 41 | | | |
| rs12971645 | | 19 | | | 45807945 | | | | MARK4 | | | | G | | | | | | | A | | | | 0.725 | | | | 0.012 | | | | | | 0.002 | | | | | | 4.3E-08 | | | | | | | 1.22E-04 | | | | 30 | | | |
| rs1800437 | | 19 | | | 46181392 | | | | GIPR | | | | G | | | | | | | C | | | | 0.805 | | | | 0.023 | | | | | | 0.002 | | | | | | 1.8E-20 | | | | | | | 3.49E-04 | | | | 86 | | | |
| rs3810291 | | 19 | | | 47569003 | | | | ZC3H4 | | | | G | | | | | | | A | | | | 0.324 | | | | -0.015 | | | | | | 0.002 | | | | | | 4.7E-13 | | | | | | | 2.12E-04 | | | | 52 | | | |
| rs8124896 | | 20 | | | 21385659 | | | | NKX2-4 | | | | T | | | | | | | C | | | | 0.899 | | | | -0.020 | | | | | | 0.003 | | | | | | 6.9E-10 | | | | | | | 1.54E-04 | | | | 38 | | | |
| rs116948922 | | 20 | | | 25534854 | | | | NINL | | | | C | | | | | | | T | | | | 0.967 | | | | 0.034 | | | | | | 0.006 | | | | | | 1.5E-09 | | | | | | | 1.48E-04 | | | | 37 | | | |
| rs34966255 | | 20 | | | 51193862 | | | | ZFP64 | | | | T | | | | | | | C | | | | 0.808 | | | | 0.016 | | | | | | 0.003 | | | | | | 6.2E-11 | | | | | | | 1.73E-04 | | | | 43 | | | |
| rs915814 | | 21 | | | 46493003 | | | | AP001579.1 | | | | G | | | | | | | A | | | | 0.761 | | | | 0.013 | | | | | | 0.002 | | | | | | 1.5E-08 | | | | | | | 1.30E-04 | | | | 32 | | | |
| rs400997 | | 21 | | | 46564154 | | | | ADARB1 | | | | T | | | | | | | A | | | | 0.441 | | | | -0.014 | | | | | | 0.002 | | | | | | 8E-13 | | | | | | | 2.08E-04 | | | | 51 | | | |
| rs738140 | | 22 | | | 41884954 | | | | ACO2 | | | | A | | | | | | | G | | | | 0.689 | | | | 0.012 | | | | | | 0.002 | | | | | | 1.80E-08 | | | | | | | 1.28E-04 | | | | 32 | | | |
| Abbreviations: Chr Chromosome; EA effect allele; EAF effect allele frequency; OA other allele; SE standard error | | | | | | | | | | | | | | | | | | | | | | | | | | | | | | | | | | | | | | | | | | | | | | | | | | | | | | |

| **Supplementary table 2: Summary information on colorectal cancer risk for the SNPs used in the analysis** | | | | | | | | | | | | | | | | | | | | | | | | | | | | | | | | | |
| --- | --- | --- | --- | --- | --- | --- | --- | --- | --- | --- | --- | --- | --- | --- | --- | --- | --- | --- | --- | --- | --- | --- | --- | --- | --- | --- | --- | --- | --- | --- | --- | --- | --- |
| Early life body size SNPs (overall) | | | | | | | | | | | | | | | | | | | | | | | | | | | | | | | | | |
| SNP | EA | | | OA | | | beta_ca | | se_ca | | | | | beta_co | | se_co | | | | | | beta_prox | | se_prox | | beta_dist | | se_dist | | beta_re | | se_re | |
| rs2229330 | T | | | G | | | -0.018 | | 0.021 | | | | | -0.024 | | 0.025 | | | | | | -0.029 | | 0.031 | | -0.019 | | 0.032 | | -0.019 | | 0.033 | |
| rs2175171 | G | | | C | | | 0.005 | | 0.010 | | | | | 0.000 | | 0.012 | | | | | | -0.001 | | 0.015 | | -0.006 | | 0.015 | | -0.001 | | 0.015 | |
| rs6577497 | A | | | T | | | -0.007 | | 0.010 | | | | | -0.004 | | 0.012 | | | | | | 0.009 | | 0.015 | | -0.012 | | 0.016 | | -0.014 | | 0.016 | |
| rs12045879 | C | | | T | | | -0.018 | | 0.010 | | | | | -0.014 | | 0.013 | | | | | | -0.014 | | 0.016 | | -0.012 | | 0.016 | | -0.014 | | 0.016 | |
| rs212517 | T | | | A | | | 0.009 | | 0.010 | | | | | 0.003 | | 0.012 | | | | | | 0.013 | | 0.015 | | -0.019 | | 0.015 | | 0.011 | | 0.015 | |
| rs2356864 | G | | | A | | | 0.009 | | 0.010 | | | | | 0.022 | | 0.012 | | | | | | 0.035 | | 0.015 | | 0.014 | | 0.015 | | -0.003 | | 0.015 | |
| rs630602 | G | | | C | | | -0.004 | | 0.010 | | | | | -0.014 | | 0.012 | | | | | | -0.007 | | 0.015 | | -0.017 | | 0.016 | | 0.015 | | 0.016 | |
| rs12140153 | G | | | T | | | 0.015 | | 0.019 | | | | | 0.026 | | 0.022 | | | | | | 0.041 | | 0.028 | | -0.003 | | 0.029 | | -0.024 | | 0.029 | |
| rs2767486 | A | | | G | | | -0.013 | | 0.012 | | | | | -0.016 | | 0.014 | | | | | | -0.004 | | 0.018 | | -0.029 | | 0.018 | | -0.022 | | 0.019 | |
| rs7522014 | A | | | G | | | -0.008 | | 0.011 | | | | | 0.007 | | 0.013 | | | | | | -0.011 | | 0.016 | | 0.025 | | 0.016 | | -0.015 | | 0.016 | |
| rs2755253 | C | | | T | | | 0.033 | | 0.011 | | | | | 0.029 | | 0.013 | | | | | | 0.026 | | 0.016 | | 0.038 | | 0.017 | | 0.042 | | 0.017 | |
| rs11209943 | A | | | G | | | 0.017 | | 0.010 | | | | | 0.011 | | 0.012 | | | | | | 0.009 | | 0.015 | | 0.005 | | 0.016 | | 0.035 | | 0.016 | |
| rs12042908 | A | | | G | | | 0.001 | | 0.010 | | | | | 0.011 | | 0.012 | | | | | | 0.010 | | 0.015 | | 0.019 | | 0.015 | | 0.006 | | 0.015 | |
| rs34517439 | C | | | A | | | -0.007 | | 0.017 | | | | | -0.016 | | 0.020 | | | | | | -0.011 | | 0.025 | | -0.023 | | 0.026 | | -0.015 | | 0.026 | |
| rs11165687 | C | | | T | | | -0.014 | | 0.010 | | | | | -0.016 | | 0.012 | | | | | | -0.006 | | 0.015 | | -0.024 | | 0.015 | | -0.015 | | 0.015 | |
| rs7550711 | C | | | T | | | 0.003 | | 0.029 | | | | | -0.012 | | 0.034 | | | | | | -0.014 | | 0.044 | | -0.004 | | 0.045 | | -0.004 | | 0.044 | |
| rs3013431 | C | | | T | | | -0.008 | | 0.010 | | | | | -0.003 | | 0.012 | | | | | | -0.020 | | 0.015 | | 0.013 | | 0.015 | | -0.031 | | 0.016 | |
| rs12132598 | A | | | G | | | -0.017 | | 0.010 | | | | | -0.015 | | 0.012 | | | | | | -0.005 | | 0.015 | | -0.021 | | 0.016 | | -0.028 | | 0.016 | |
| rs7536458 | T | | | G | | | 0.031 | | 0.011 | | | | | 0.030 | | 0.013 | | | | | | 0.042 | | 0.017 | | 0.012 | | 0.017 | | 0.042 | | 0.017 | |
| rs11205303 | T | | | C | | | 0.011 | | 0.010 | | | | | 0.031 | | 0.012 | | | | | | 0.017 | | 0.016 | | 0.037 | | 0.016 | | 0.004 | | 0.016 | |
| rs35588936 | C | | | T | | | -0.005 | | 0.019 | | | | | -0.002 | | 0.022 | | | | | | 0.021 | | 0.028 | | -0.026 | | 0.028 | | 0.016 | | 0.029 | |
| rs12748436 | C | | | G | | | -0.027 | | 0.018 | | | | | -0.006 | | 0.021 | | | | | | -0.030 | | 0.027 | | 0.007 | | 0.028 | | -0.030 | | 0.028 | |
| rs543874 | A | | | G | | | -0.003 | | 0.012 | | | | | 0.002 | | 0.015 | | | | | | -0.005 | | 0.019 | | 0.006 | | 0.019 | | 0.002 | | 0.019 | |
| rs78444298 | G | | | A | | | 0.014 | | 0.042 | | | | | 0.038 | | 0.050 | | | | | | 0.072 | | 0.063 | | 0.008 | | 0.065 | | -0.009 | | 0.065 | |
| rs4074404 | T | | | A | | | -0.018 | | 0.013 | | | | | -0.015 | | 0.016 | | | | | | -0.014 | | 0.020 | | -0.019 | | 0.020 | | -0.025 | | 0.020 | |
| rs16839832 | G | | | T | | | 0.014 | | 0.018 | | | | | 0.009 | | 0.022 | | | | | | -0.005 | | 0.027 | | 0.029 | | 0.029 | | 0.001 | | 0.029 | |
| rs9438393 | A | | | G | | | 0.033 | | 0.010 | | | | | 0.033 | | 0.012 | | | | | | 0.021 | | 0.015 | | 0.043 | | 0.015 | | 0.040 | | 0.015 | |
| rs7354849 | A | | | G | | | -0.009 | | 0.010 | | | | | -0.005 | | 0.012 | | | | | | -0.003 | | 0.015 | | -0.008 | | 0.015 | | -0.017 | | 0.015 | |
| rs62106258 | T | | | C | | | 0.046 | | 0.026 | | | | | 0.050 | | 0.030 | | | | | | 0.047 | | 0.038 | | 0.068 | | 0.040 | | 0.043 | | 0.040 | |
| rs12992672 | G | | | A | | | -0.018 | | 0.013 | | | | | -0.024 | | 0.015 | | | | | | -0.022 | | 0.019 | | -0.024 | | 0.020 | | -0.023 | | 0.020 | |
| rs2867116 | C | | | A | | | -0.012 | | 0.015 | | | | | -0.015 | | 0.017 | | | | | | -0.014 | | 0.022 | | -0.027 | | 0.022 | | -0.029 | | 0.022 | |
| rs2141004 | A | | | C | | | 0.010 | | 0.011 | | | | | 0.009 | | 0.013 | | | | | | 0.001 | | 0.016 | | 0.016 | | 0.017 | | 0.015 | | 0.017 | |
| rs10182458 | A | | | G | | | -0.005 | | 0.010 | | | | | -0.011 | | 0.012 | | | | | | -0.018 | | 0.015 | | -0.009 | | 0.015 | | -0.013 | | 0.015 | |
| rs146910503 | G | | | A | | | 0.075 | | 0.037 | | | | | 0.082 | | 0.044 | | | | | | 0.110 | | 0.057 | | 0.039 | | 0.058 | | 0.011 | | 0.056 | |
| rs6719507 | G | | | A | | | -0.007 | | 0.010 | | | | | 0.005 | | 0.012 | | | | | | 0.016 | | 0.015 | | -0.002 | | 0.015 | | -0.018 | | 0.015 | |
| rs62134189 | A | | | G | | | -0.006 | | 0.017 | | | | | -0.007 | | 0.020 | | | | | | -0.012 | | 0.025 | | -0.005 | | 0.026 | | -0.044 | | 0.026 | |
| rs2902142 | C | | | T | | | -0.016 | | 0.010 | | | | | -0.007 | | 0.012 | | | | | | -0.002 | | 0.015 | | -0.010 | | 0.015 | | -0.006 | | 0.016 | |
| rs2539692 | T | | | A | | | 0.003 | | 0.010 | | | | | -0.003 | | 0.012 | | | | | | -0.001 | | 0.015 | | -0.002 | | 0.015 | | 0.002 | | 0.016 | |
| rs1177279 | A | | | G | | | 0.005 | | 0.011 | | | | | 0.027 | | 0.013 | | | | | | 0.011 | | 0.017 | | 0.042 | | 0.017 | | -0.017 | | 0.017 | |
| rs7565437 | T | | | C | | | 0.007 | | 0.010 | | | | | 0.006 | | 0.012 | | | | | | 0.006 | | 0.015 | | 0.006 | | 0.015 | | 0.019 | | 0.015 | |
| rs12713889 | T | | | C | | | -0.001 | | 0.010 | | | | | 0.002 | | 0.012 | | | | | | -0.002 | | 0.015 | | 0.005 | | 0.016 | | 0.001 | | 0.016 | |
| rs772175 | G | | | A | | | -0.004 | | 0.010 | | | | | -0.007 | | 0.012 | | | | | | -0.023 | | 0.016 | | 0.012 | | 0.016 | | -0.005 | | 0.016 | |
| rs1384660 | G | | | A | | | -0.015 | | 0.012 | | | | | -0.015 | | 0.015 | | | | | | 0.003 | | 0.019 | | -0.040 | | 0.019 | | -0.032 | | 0.020 | |
| rs62175963 | T | | | C | | | 0.014 | | 0.010 | | | | | 0.021 | | 0.012 | | | | | | 0.004 | | 0.015 | | 0.034 | | 0.016 | | 0.004 | | 0.016 | |
| rs115319174 | G | | | C | | | -0.025 | | 0.022 | | | | | -0.011 | | 0.026 | | | | | | 0.021 | | 0.033 | | -0.038 | | 0.033 | | -0.033 | | 0.034 | |
| rs11891707 | T | | | C | | | 0.008 | | 0.014 | | | | | 0.006 | | 0.017 | | | | | | -0.006 | | 0.021 | | 0.011 | | 0.022 | | 0.005 | | 0.022 | |
| rs3791478 | T | | | C | | | -0.006 | | 0.016 | | | | | -0.014 | | 0.019 | | | | | | -0.016 | | 0.024 | | -0.007 | | 0.025 | | -0.014 | | 0.025 | |
| rs1476698 | A | | | G | | | 0.010 | | 0.010 | | | | | 0.012 | | 0.012 | | | | | | 0.000 | | 0.015 | | 0.027 | | 0.016 | | 0.011 | | 0.016 | |
| rs2594994 | T | | | A | | | 0.007 | | 0.013 | | | | | 0.003 | | 0.015 | | | | | | -0.004 | | 0.019 | | 0.013 | | 0.019 | | 0.036 | | 0.020 | |
| rs7619139 | T | | | A | | | 0.001 | | 0.010 | | | | | 0.002 | | 0.012 | | | | | | -0.005 | | 0.015 | | 0.007 | | 0.015 | | -0.012 | | 0.015 | |
| rs1402989 | C | | | T | | | -0.001 | | 0.010 | | | | | 0.003 | | 0.012 | | | | | | 0.009 | | 0.015 | | -0.003 | | 0.015 | | -0.001 | | 0.015 | |
| rs2268762 | A | | | G | | | 0.014 | | 0.010 | | | | | 0.017 | | 0.012 | | | | | | 0.023 | | 0.015 | | 0.007 | | 0.015 | | -0.012 | | 0.016 | |
| rs754635 | C | | | G | | | -0.016 | | 0.015 | | | | | -0.021 | | 0.018 | | | | | | -0.012 | | 0.023 | | -0.026 | | 0.024 | | -0.029 | | 0.024 | |
| rs2034963 | G | | | C | | | -0.002 | | 0.010 | | | | | 0.001 | | 0.012 | | | | | | -0.006 | | 0.016 | | 0.018 | | 0.016 | | -0.012 | | 0.016 | |
| rs35926495 | C | | | T | | | -0.012 | | 0.010 | | | | | -0.014 | | 0.012 | | | | | | -0.014 | | 0.015 | | -0.011 | | 0.016 | | -0.014 | | 0.016 | |
| rs3774604 | C | | | T | | | -0.020 | | 0.010 | | | | | -0.015 | | 0.012 | | | | | | -0.018 | | 0.015 | | -0.013 | | 0.016 | | -0.026 | | 0.016 | |
| rs2629881 | C | | | T | | | 0.007 | | 0.012 | | | | | 0.010 | | 0.015 | | | | | | 0.009 | | 0.019 | | 0.011 | | 0.019 | | 0.013 | | 0.019 | |
| rs538579 | G | | | C | | | -0.014 | | 0.010 | | | | | -0.020 | | 0.012 | | | | | | -0.009 | | 0.016 | | -0.029 | | 0.016 | | -0.017 | | 0.016 | |
| rs115903965 | G | | | A | | | 0.031 | | 0.044 | | | | | 0.008 | | 0.052 | | | | | | -0.021 | | 0.064 | | 0.046 | | 0.069 | | 0.022 | | 0.069 | |
| rs4677156 | A | | | T | | | 0.033 | | 0.012 | | | | | 0.037 | | 0.014 | | | | | | 0.030 | | 0.018 | | 0.052 | | 0.018 | | 0.042 | | 0.018 | |
| rs1666132 | C | | | T | | | -0.010 | | 0.010 | | | | | -0.019 | | 0.012 | | | | | | -0.009 | | 0.015 | | -0.031 | | 0.015 | | -0.001 | | 0.015 | |
| rs1357798 | C | | | T | | | 0.000 | | 0.012 | | | | | -0.004 | | 0.014 | | | | | | -0.002 | | 0.018 | | -0.005 | | 0.018 | | 0.018 | | 0.018 | |
| rs6783281 | A | | | G | | | 0.005 | | 0.011 | | | | | 0.015 | | 0.013 | | | | | | 0.006 | | 0.016 | | 0.021 | | 0.017 | | 0.019 | | 0.017 | |
| rs7355953 | T | | | C | | | 0.020 | | 0.012 | | | | | 0.029 | | 0.014 | | | | | | 0.038 | | 0.018 | | 0.024 | | 0.018 | | 0.024 | | 0.018 | |
| rs2735556 | T | | | C | | | 0.005 | | 0.016 | | | | | -0.004 | | 0.019 | | | | | | -0.016 | | 0.024 | | 0.005 | | 0.025 | | 0.035 | | 0.026 | |
| rs11925138 | G | | | A | | | -0.001 | | 0.016 | | | | | 0.002 | | 0.019 | | | | | | -0.008 | | 0.024 | | 0.011 | | 0.025 | | -0.031 | | 0.025 | |
| rs7625768 | G | | | A | | | -0.002 | | 0.010 | | | | | 0.009 | | 0.013 | | | | | | -0.006 | | 0.016 | | 0.021 | | 0.016 | | -0.034 | | 0.016 | |
| rs1199333 | G | | | T | | | 0.018 | | 0.012 | | | | | 0.035 | | 0.015 | | | | | | 0.038 | | 0.018 | | 0.027 | | 0.019 | | -0.030 | | 0.019 | |
| rs59714050 | T | | | A | | | 0.006 | | 0.019 | | | | | 0.000 | | 0.024 | | | | | | -0.033 | | 0.030 | | 0.051 | | 0.031 | | 0.002 | | 0.031 | |
| rs355748 | G | | | T | | | 0.000 | | 0.010 | | | | | 0.004 | | 0.012 | | | | | | 0.004 | | 0.015 | | 0.013 | | 0.015 | | -0.009 | | 0.015 | |
| rs7633995 | A | | | G | | | -0.001 | | 0.017 | | | | | -0.026 | | 0.020 | | | | | | -0.027 | | 0.026 | | -0.023 | | 0.026 | | 0.037 | | 0.027 | |
| rs10937241 | A | | | G | | | 0.014 | | 0.013 | | | | | 0.020 | | 0.016 | | | | | | 0.031 | | 0.020 | | 0.012 | | 0.021 | | -0.011 | | 0.021 | |
| rs34811474 | G | | | A | | | 0.022 | | 0.013 | | | | | 0.038 | | 0.016 | | | | | | 0.033 | | 0.020 | | 0.043 | | 0.021 | | 0.016 | | 0.021 | |
| rs7656673 | A | | | G | | | -0.001 | | 0.010 | | | | | -0.006 | | 0.012 | | | | | | 0.003 | | 0.015 | | -0.014 | | 0.015 | | -0.003 | | 0.015 | |
| rs34722008 | G | | | A | | | 0.004 | | 0.011 | | | | | 0.009 | | 0.013 | | | | | | 0.018 | | 0.016 | | 0.002 | | 0.016 | | 0.015 | | 0.016 | |
| rs7439324 | C | | | T | | | 0.008 | | 0.013 | | | | | 0.034 | | 0.016 | | | | | | 0.033 | | 0.020 | | 0.042 | | 0.020 | | -0.011 | | 0.020 | |
| rs12641981 | C | | | T | | | 0.000 | | 0.010 | | | | | -0.005 | | 0.012 | | | | | | -0.008 | | 0.015 | | 0.002 | | 0.015 | | -0.001 | | 0.015 | |
| rs788858 | A | | | G | | | -0.008 | | 0.011 | | | | | -0.012 | | 0.013 | | | | | | -0.020 | | 0.016 | | -0.004 | | 0.017 | | 0.001 | | 0.017 | |
| rs7377083 | C | | | A | | | 0.000 | | 0.010 | | | | | -0.004 | | 0.012 | | | | | | -0.004 | | 0.015 | | -0.004 | | 0.016 | | 0.006 | | 0.016 | |
| rs72675820 | A | | | C | | | 0.000 | | 0.010 | | | | | -0.002 | | 0.012 | | | | | | 0.002 | | 0.015 | | -0.003 | | 0.016 | | 0.006 | | 0.016 | |
| rs35189091 | A | | | G | | | -0.009 | | 0.010 | | | | | -0.005 | | 0.012 | | | | | | -0.019 | | 0.015 | | 0.011 | | 0.015 | | -0.018 | | 0.016 | |
| rs11727676 | T | | | C | | | -0.078 | | 0.017 | | | | | -0.091 | | 0.020 | | | | | | -0.090 | | 0.025 | | -0.094 | | 0.026 | | -0.077 | | 0.026 | |
| rs3936511 | A | | | G | | | -0.009 | | 0.012 | | | | | -0.019 | | 0.015 | | | | | | -0.026 | | 0.019 | | -0.010 | | 0.019 | | 0.013 | | 0.019 | |
| rs6449532 | C | | | T | | | 0.006 | | 0.010 | | | | | 0.008 | | 0.012 | | | | | | -0.002 | | 0.015 | | 0.028 | | 0.016 | | 0.007 | | 0.016 | |
| rs9291816 | C | | | T | | | 0.006 | | 0.010 | | | | | 0.007 | | 0.012 | | | | | | 0.003 | | 0.016 | | 0.010 | | 0.016 | | 0.010 | | 0.016 | |
| rs39862 | T | | | C | | | 0.010 | | 0.011 | | | | | 0.003 | | 0.013 | | | | | | 0.000 | | 0.016 | | -0.001 | | 0.017 | | 0.009 | | 0.017 | |
| rs2307111 | T | | | C | | | 0.016 | | 0.010 | | | | | 0.008 | | 0.012 | | | | | | -0.003 | | 0.015 | | 0.018 | | 0.015 | | 0.009 | | 0.015 | |
| rs1422067 | C | | | T | | | -0.012 | | 0.012 | | | | | -0.001 | | 0.014 | | | | | | 0.014 | | 0.018 | | -0.028 | | 0.018 | | -0.030 | | 0.018 | |
| rs2115885 | G | | | A | | | -0.031 | | 0.012 | | | | | -0.036 | | 0.014 | | | | | | -0.034 | | 0.017 | | -0.040 | | 0.018 | | -0.011 | | 0.018 | |
| rs77960 | G | | | A | | | 0.017 | | 0.010 | | | | | 0.019 | | 0.012 | | | | | | 0.026 | | 0.016 | | 0.016 | | 0.016 | | 0.021 | | 0.016 | |
| rs4958568 | G | | | A | | | -0.002 | | 0.011 | | | | | -0.002 | | 0.013 | | | | | | -0.003 | | 0.016 | | -0.009 | | 0.017 | | -0.013 | | 0.017 | |
| rs7719067 | A | | | G | | | 0.012 | | 0.010 | | | | | 0.018 | | 0.012 | | | | | | 0.014 | | 0.015 | | 0.009 | | 0.015 | | -0.011 | | 0.015 | |
| rs7711823 | A | | | G | | | 0.017 | | 0.010 | | | | | 0.016 | | 0.012 | | | | | | 0.012 | | 0.015 | | 0.018 | | 0.016 | | 0.036 | | 0.016 | |
| rs918472 | G | | | A | | | 0.001 | | 0.011 | | | | | 0.000 | | 0.013 | | | | | | 0.000 | | 0.016 | | -0.002 | | 0.016 | | -0.017 | | 0.017 | |
| rs12214497 | G | | | T | | | 0.002 | | 0.010 | | | | | 0.006 | | 0.012 | | | | | | 0.031 | | 0.015 | | -0.019 | | 0.016 | | -0.027 | | 0.016 | |
| rs10498713 | G | | | T | | | -0.006 | | 0.014 | | | | | -0.004 | | 0.016 | | | | | | 0.026 | | 0.021 | | -0.033 | | 0.021 | | -0.012 | | 0.021 | |
| rs35162296 | C | | | T | | | -0.064 | | 0.019 | | | | | -0.073 | | 0.022 | | | | | | -0.098 | | 0.027 | | -0.062 | | 0.028 | | -0.089 | | 0.028 | |
| rs34196306 | G | | | C | | | -0.064 | | 0.019 | | | | | -0.073 | | 0.022 | | | | | | -0.107 | | 0.028 | | -0.043 | | 0.029 | | -0.087 | | 0.029 | |
| rs3131336 | C | | | T | | | -0.059 | | 0.018 | | | | | -0.069 | | 0.021 | | | | | | -0.107 | | 0.026 | | -0.037 | | 0.027 | | -0.075 | | 0.027 | |
| rs3129942 | G | | | T | | | -0.022 | | 0.011 | | | | | -0.020 | | 0.014 | | | | | | -0.024 | | 0.017 | | -0.012 | | 0.018 | | -0.031 | | 0.018 | |
| rs9366803 | T | | | C | | | -0.023 | | 0.016 | | | | | -0.018 | | 0.020 | | | | | | -0.010 | | 0.025 | | -0.028 | | 0.025 | | -0.051 | | 0.024 | |
| rs686431 | C | | | T | | | -0.113 | | 0.037 | | | | | -0.130 | | 0.043 | | | | | | -0.156 | | 0.053 | | -0.139 | | 0.055 | | -0.147 | | 0.057 | |
| rs73422097 | A | | | G | | | 0.012 | | 0.010 | | | | | 0.007 | | 0.013 | | | | | | 0.010 | | 0.016 | | 0.007 | | 0.016 | | 0.026 | | 0.016 | |
| rs76187039 | G | | | T | | | 0.012 | | 0.015 | | | | | 0.003 | | 0.017 | | | | | | 0.005 | | 0.022 | | -0.006 | | 0.023 | | 0.016 | | 0.023 | |
| rs3798519 | A | | | C | | | 0.009 | | 0.012 | | | | | 0.006 | | 0.015 | | | | | | 0.020 | | 0.019 | | -0.005 | | 0.019 | | 0.025 | | 0.020 | |
| rs1775255 | G | | | T | | | -0.011 | | 0.010 | | | | | -0.009 | | 0.012 | | | | | | -0.010 | | 0.015 | | -0.004 | | 0.015 | | -0.010 | | 0.015 | |
| rs1342831 | T | | | C | | | 0.009 | | 0.018 | | | | | 0.024 | | 0.023 | | | | | | 0.028 | | 0.029 | | 0.019 | | 0.030 | | 0.005 | | 0.030 | |
| rs12110721 | G | | | A | | | -0.030 | | 0.013 | | | | | -0.041 | | 0.016 | | | | | | -0.061 | | 0.020 | | -0.019 | | 0.021 | | -0.026 | | 0.021 | |
| rs9370527 | G | | | A | | | 0.007 | | 0.011 | | | | | 0.006 | | 0.014 | | | | | | 0.011 | | 0.017 | | 0.008 | | 0.018 | | 0.020 | | 0.018 | |
| rs435775 | A | | | G | | | 0.000 | | 0.011 | | | | | -0.010 | | 0.013 | | | | | | -0.018 | | 0.017 | | -0.009 | | 0.017 | | -0.020 | | 0.017 | |
| rs6931604 | C | | | T | | | 0.030 | | 0.010 | | | | | 0.039 | | 0.012 | | | | | | 0.014 | | 0.015 | | 0.059 | | 0.015 | | 0.032 | | 0.015 | |
| rs34260097 | T | | | G | | | -0.014 | | 0.012 | | | | | -0.011 | | 0.014 | | | | | | -0.017 | | 0.018 | | -0.014 | | 0.018 | | -0.007 | | 0.018 | |
| rs7759938 | C | | | T | | | 0.001 | | 0.010 | | | | | 0.000 | | 0.012 | | | | | | 0.000 | | 0.016 | | 0.000 | | 0.016 | | -0.003 | | 0.016 | |
| rs7753558 | C | | | A | | | -0.001 | | 0.010 | | | | | -0.008 | | 0.012 | | | | | | 0.003 | | 0.015 | | -0.014 | | 0.016 | | 0.017 | | 0.016 | |
| rs1452991 | G | | | A | | | 0.012 | | 0.010 | | | | | 0.009 | | 0.012 | | | | | | 0.017 | | 0.015 | | 0.008 | | 0.016 | | 0.020 | | 0.016 | |
| rs796915 | C | | | G | | | 0.016 | | 0.010 | | | | | 0.019 | | 0.012 | | | | | | 0.011 | | 0.016 | | 0.026 | | 0.016 | | 0.024 | | 0.016 | |
| rs62425398 | C | | | A | | | -0.007 | | 0.017 | | | | | 0.008 | | 0.020 | | | | | | 0.005 | | 0.025 | | 0.010 | | 0.026 | | -0.029 | | 0.026 | |
| rs2349179 | T | | | A | | | -0.026 | | 0.010 | | | | | -0.034 | | 0.012 | | | | | | -0.018 | | 0.015 | | -0.045 | | 0.016 | | -0.019 | | 0.016 | |
| rs2722406 | C | | | T | | | -0.002 | | 0.011 | | | | | -0.009 | | 0.013 | | | | | | -0.010 | | 0.016 | | -0.014 | | 0.016 | | 0.010 | | 0.017 | |
| rs4723263 | G | | | C | | | -0.001 | | 0.010 | | | | | -0.007 | | 0.012 | | | | | | -0.014 | | 0.015 | | -0.008 | | 0.015 | | 0.007 | | 0.015 | |
| rs10234366 | G | | | A | | | -0.016 | | 0.016 | | | | | -0.021 | | 0.018 | | | | | | 0.002 | | 0.023 | | -0.045 | | 0.024 | | -0.032 | | 0.024 | |
| rs1852006 | G | | | A | | | -0.011 | | 0.010 | | | | | -0.007 | | 0.012 | | | | | | -0.014 | | 0.015 | | 0.001 | | 0.016 | | -0.009 | | 0.016 | |
| rs6974282 | C | | | T | | | -0.035 | | 0.012 | | | | | -0.024 | | 0.015 | | | | | | -0.031 | | 0.018 | | -0.012 | | 0.019 | | -0.052 | | 0.019 | |
| rs7808296 | C | | | T | | | -0.002 | | 0.010 | | | | | 0.011 | | 0.013 | | | | | | 0.010 | | 0.016 | | 0.006 | | 0.016 | | -0.037 | | 0.016 | |
| rs262338 | G | | | T | | | -0.003 | | 0.010 | | | | | -0.002 | | 0.012 | | | | | | -0.001 | | 0.015 | | -0.004 | | 0.015 | | -0.030 | | 0.015 | |
| rs10953577 | T | | | C | | | 0.008 | | 0.010 | | | | | 0.011 | | 0.012 | | | | | | 0.014 | | 0.015 | | 0.020 | | 0.015 | | -0.011 | | 0.015 | |
| rs67679818 | C | | | T | | | -0.017 | | 0.010 | | | | | -0.024 | | 0.012 | | | | | | -0.039 | | 0.015 | | -0.009 | | 0.015 | | -0.004 | | 0.015 | |
| rs6979832 | A | | | G | | | 0.001 | | 0.010 | | | | | -0.006 | | 0.012 | | | | | | -0.003 | | 0.015 | | -0.013 | | 0.015 | | 0.020 | | 0.015 | |
| rs11525873 | T | | | C | | | 0.017 | | 0.016 | | | | | 0.015 | | 0.020 | | | | | | 0.008 | | 0.026 | | 0.028 | | 0.027 | | 0.043 | | 0.027 | |
| rs10503246 | A | | | G | | | -0.003 | | 0.011 | | | | | 0.001 | | 0.013 | | | | | | 0.006 | | 0.016 | | -0.003 | | 0.017 | | -0.001 | | 0.017 | |
| rs77976727 | C | | | T | | | -0.005 | | 0.016 | | | | | -0.017 | | 0.019 | | | | | | -0.013 | | 0.024 | | -0.031 | | 0.025 | | 0.012 | | 0.025 | |
| rs7814267 | A | | | G | | | -0.018 | | 0.012 | | | | | -0.021 | | 0.015 | | | | | | -0.030 | | 0.019 | | -0.015 | | 0.019 | | -0.019 | | 0.019 | |
| rs11777719 | A | | | G | | | -0.012 | | 0.011 | | | | | 0.002 | | 0.013 | | | | | | 0.023 | | 0.017 | | -0.024 | | 0.017 | | -0.041 | | 0.018 | |
| rs13256357 | C | | | T | | | -0.006 | | 0.012 | | | | | -0.010 | | 0.015 | | | | | | 0.001 | | 0.019 | | -0.017 | | 0.019 | | -0.001 | | 0.020 | |
| rs2409743 | C | | | G | | | -0.001 | | 0.011 | | | | | -0.017 | | 0.013 | | | | | | -0.032 | | 0.016 | | 0.004 | | 0.017 | | 0.017 | | 0.017 | |
| rs10503555 | A | | | G | | | -0.004 | | 0.010 | | | | | 0.002 | | 0.012 | | | | | | -0.003 | | 0.015 | | 0.008 | | 0.015 | | -0.025 | | 0.015 | |
| rs884152 | G | | | T | | | 0.008 | | 0.010 | | | | | 0.013 | | 0.012 | | | | | | 0.006 | | 0.015 | | 0.029 | | 0.016 | | 0.011 | | 0.016 | |
| rs7012648 | G | | | A | | | -0.001 | | 0.010 | | | | | 0.001 | | 0.012 | | | | | | -0.015 | | 0.015 | | 0.018 | | 0.015 | | 0.002 | | 0.015 | |
| rs4739558 | A | | | G | | | 0.007 | | 0.010 | | | | | 0.011 | | 0.012 | | | | | | 0.004 | | 0.015 | | 0.014 | | 0.015 | | 0.008 | | 0.016 | |
| rs10095724 | G | | | A | | | 0.015 | | 0.010 | | | | | 0.020 | | 0.012 | | | | | | 0.020 | | 0.015 | | 0.018 | | 0.016 | | 0.023 | | 0.016 | |
| rs10111937 | C | | | T | | | 0.004 | | 0.010 | | | | | 0.002 | | 0.012 | | | | | | 0.000 | | 0.016 | | 0.008 | | 0.016 | | 0.009 | | 0.016 | |
| rs7840305 | A | | | G | | | 0.002 | | 0.010 | | | | | 0.007 | | 0.012 | | | | | | 0.004 | | 0.015 | | 0.016 | | 0.016 | | -0.007 | | 0.016 | |
| rs13254613 | A | | | C | | | 0.001 | | 0.010 | | | | | 0.013 | | 0.012 | | | | | | 0.030 | | 0.015 | | -0.006 | | 0.016 | | 0.001 | | 0.016 | |
| rs7817581 | G | | | A | | | 0.016 | | 0.011 | | | | | 0.024 | | 0.014 | | | | | | 0.018 | | 0.017 | | 0.030 | | 0.018 | | 0.027 | | 0.018 | |
| rs10962279 | T | | | C | | | 0.004 | | 0.012 | | | | | 0.008 | | 0.015 | | | | | | 0.005 | | 0.019 | | -0.005 | | 0.019 | | -0.006 | | 0.019 | |
| rs3118252 | G | | | C | | | 0.001 | | 0.010 | | | | | -0.001 | | 0.012 | | | | | | -0.019 | | 0.015 | | 0.018 | | 0.015 | | -0.017 | | 0.016 | |
| rs1935354 | T | | | C | | | -0.010 | | 0.010 | | | | | -0.020 | | 0.012 | | | | | | -0.022 | | 0.015 | | -0.018 | | 0.015 | | -0.011 | | 0.015 | |
| rs1619120 | A | | | G | | | -0.001 | | 0.010 | | | | | 0.007 | | 0.012 | | | | | | 0.008 | | 0.015 | | 0.002 | | 0.015 | | -0.029 | | 0.015 | |
| rs4744246 | A | | | G | | | 0.015 | | 0.010 | | | | | 0.016 | | 0.012 | | | | | | 0.053 | | 0.015 | | -0.023 | | 0.016 | | -0.014 | | 0.016 | |
| rs7020564 | A | | | T | | | 0.010 | | 0.011 | | | | | 0.016 | | 0.013 | | | | | | 0.016 | | 0.016 | | 0.015 | | 0.016 | | 0.007 | | 0.017 | |
| rs957512 | T | | | C | | | 0.018 | | 0.010 | | | | | 0.028 | | 0.012 | | | | | | 0.023 | | 0.015 | | 0.034 | | 0.016 | | 0.026 | | 0.016 | |
| rs10116891 | G | | | A | | | 0.043 | | 0.017 | | | | | 0.032 | | 0.019 | | | | | | 0.054 | | 0.025 | | 0.006 | | 0.025 | | 0.056 | | 0.026 | |
| rs2275241 | G | | | A | | | -0.012 | | 0.010 | | | | | -0.025 | | 0.012 | | | | | | -0.026 | | 0.015 | | -0.021 | | 0.016 | | 0.007 | | 0.016 | |
| rs117911387 | G | | | A | | | 0.008 | | 0.026 | | | | | 0.021 | | 0.031 | | | | | | -0.010 | | 0.038 | | 0.062 | | 0.040 | | -0.025 | | 0.040 | |
| rs7084503 | T | | | C | | | 0.020 | | 0.010 | | | | | 0.018 | | 0.012 | | | | | | 0.010 | | 0.015 | | 0.024 | | 0.015 | | 0.037 | | 0.015 | |
| rs11256627 | G | | | A | | | 0.024 | | 0.011 | | | | | 0.020 | | 0.013 | | | | | | 0.028 | | 0.016 | | 0.015 | | 0.017 | | 0.024 | | 0.017 | |
| rs4572029 | A | | | G | | | -0.014 | | 0.011 | | | | | -0.019 | | 0.014 | | | | | | -0.021 | | 0.018 | | -0.026 | | 0.018 | | -0.013 | | 0.018 | |
| rs10823504 | G | | | A | | | -0.012 | | 0.019 | | | | | -0.016 | | 0.023 | | | | | | -0.034 | | 0.029 | | 0.010 | | 0.030 | | 0.019 | | 0.030 | |
| rs2242258 | T | | | C | | | -0.001 | | 0.011 | | | | | -0.012 | | 0.014 | | | | | | -0.015 | | 0.017 | | -0.002 | | 0.018 | | 0.008 | | 0.018 | |
| rs17399739 | A | | | G | | | 0.022 | | 0.019 | | | | | 0.034 | | 0.023 | | | | | | 0.048 | | 0.029 | | 0.033 | | 0.030 | | 0.031 | | 0.030 | |
| rs10887571 | C | | | T | | | -0.002 | | 0.010 | | | | | -0.007 | | 0.012 | | | | | | -0.019 | | 0.016 | | 0.007 | | 0.016 | | 0.005 | | 0.016 | |
| rs41310284 | C | | | A | | | -0.011 | | 0.017 | | | | | -0.006 | | 0.020 | | | | | | -0.013 | | 0.025 | | 0.006 | | 0.026 | | -0.010 | | 0.026 | |
| rs75387636 | G | | | A | | | -0.007 | | 0.023 | | | | | -0.014 | | 0.029 | | | | | | 0.051 | | 0.037 | | -0.068 | | 0.037 | | -0.044 | | 0.037 | |
| rs2939931 | T | | | C | | | 0.015 | | 0.010 | | | | | 0.008 | | 0.012 | | | | | | -0.003 | | 0.015 | | 0.018 | | 0.015 | | 0.008 | | 0.015 | |
| rs1061072 | G | | | A | | | -0.006 | | 0.016 | | | | | -0.003 | | 0.019 | | | | | | -0.003 | | 0.024 | | -0.003 | | 0.024 | | -0.023 | | 0.024 | |
| rs56133711 | G | | | A | | | 0.024 | | 0.012 | | | | | 0.021 | | 0.014 | | | | | | 0.012 | | 0.017 | | 0.026 | | 0.018 | | 0.033 | | 0.018 | |
| rs4267058 | T | | | C | | | 0.025 | | 0.010 | | | | | 0.014 | | 0.012 | | | | | | -0.003 | | 0.015 | | 0.033 | | 0.016 | | 0.047 | | 0.016 | |
| rs661878 | A | | | G | | | 0.003 | | 0.014 | | | | | -0.002 | | 0.016 | | | | | | -0.001 | | 0.021 | | -0.004 | | 0.021 | | 0.012 | | 0.022 | |
| rs3181269 | C | | | T | | | -0.014 | | 0.012 | | | | | -0.004 | | 0.014 | | | | | | -0.027 | | 0.017 | | 0.020 | | 0.018 | | -0.023 | | 0.018 | |
| rs7951870 | T | | | C | | | 0.017 | | 0.013 | | | | | 0.017 | | 0.016 | | | | | | 0.012 | | 0.020 | | 0.021 | | 0.020 | | 0.031 | | 0.021 | |
| rs12798028 | C | | | T | | | -0.008 | | 0.010 | | | | | -0.004 | | 0.012 | | | | | | -0.013 | | 0.015 | | 0.000 | | 0.015 | | -0.006 | | 0.015 | |
| rs3862342 | C | | | T | | | 0.012 | | 0.011 | | | | | 0.013 | | 0.013 | | | | | | 0.013 | | 0.016 | | 0.006 | | 0.017 | | 0.005 | | 0.017 | |
| rs2958542 | C | | | T | | | -0.005 | | 0.010 | | | | | -0.012 | | 0.012 | | | | | | -0.019 | | 0.015 | | -0.012 | | 0.016 | | -0.001 | | 0.016 | |
| rs10791902 | C | | | T | | | 0.005 | | 0.011 | | | | | 0.001 | | 0.013 | | | | | | -0.001 | | 0.016 | | 0.004 | | 0.017 | | 0.012 | | 0.017 | |
| rs10896348 | T | | | C | | | -0.012 | | 0.011 | | | | | -0.011 | | 0.013 | | | | | | -0.015 | | 0.016 | | -0.003 | | 0.017 | | -0.025 | | 0.017 | |
| rs10796828 | T | | | G | | | -0.013 | | 0.010 | | | | | -0.017 | | 0.012 | | | | | | -0.006 | | 0.016 | | -0.026 | | 0.016 | | 0.005 | | 0.016 | |
| rs11215403 | G | | | A | | | -0.018 | | 0.012 | | | | | -0.019 | | 0.014 | | | | | | -0.016 | | 0.017 | | -0.013 | | 0.018 | | -0.016 | | 0.018 | |
| rs7123283 | C | | | T | | | -0.008 | | 0.010 | | | | | -0.006 | | 0.012 | | | | | | -0.026 | | 0.015 | | 0.013 | | 0.015 | | -0.027 | | 0.015 | |
| rs10790809 | A | | | G | | | -0.005 | | 0.010 | | | | | 0.008 | | 0.012 | | | | | | 0.019 | | 0.016 | | 0.002 | | 0.016 | | -0.022 | | 0.016 | |
| rs55726687 | G | | | A | | | -0.015 | | 0.012 | | | | | -0.026 | | 0.014 | | | | | | -0.033 | | 0.018 | | -0.016 | | 0.019 | | 0.007 | | 0.019 | |
| rs2187642 | A | | | C | | | 0.000 | | 0.010 | | | | | 0.005 | | 0.012 | | | | | | 0.008 | | 0.015 | | 0.000 | | 0.016 | | -0.001 | | 0.016 | |
| rs10841379 | A | | | G | | | 0.018 | | 0.010 | | | | | 0.022 | | 0.012 | | | | | | 0.016 | | 0.016 | | 0.032 | | 0.016 | | 0.010 | | 0.016 | |
| rs10842356 | A | | | T | | | -0.018 | | 0.010 | | | | | -0.017 | | 0.012 | | | | | | -0.019 | | 0.015 | | -0.020 | | 0.015 | | -0.039 | | 0.015 | |
| rs61937656 | G | | | A | | | 0.001 | | 0.012 | | | | | 0.002 | | 0.014 | | | | | | 0.005 | | 0.017 | | -0.002 | | 0.018 | | -0.002 | | 0.018 | |
| rs7958241 | A | | | G | | | 0.034 | | 0.010 | | | | | 0.036 | | 0.012 | | | | | | 0.035 | | 0.016 | | 0.042 | | 0.016 | | 0.040 | | 0.016 | |
| rs7132908 | G | | | A | | | -0.012 | | 0.010 | | | | | -0.006 | | 0.012 | | | | | | 0.004 | | 0.015 | | -0.014 | | 0.015 | | -0.006 | | 0.016 | |
| rs836179 | A | | | G | | | 0.041 | | 0.010 | | | | | 0.038 | | 0.012 | | | | | | 0.030 | | 0.015 | | 0.050 | | 0.015 | | 0.053 | | 0.016 | |
| rs78607331 | C | | | T | | | 0.003 | | 0.028 | | | | | 0.029 | | 0.034 | | | | | | 0.029 | | 0.043 | | 0.026 | | 0.044 | | -0.016 | | 0.043 | |
| rs7306710 | T | | | C | | | -0.010 | | 0.010 | | | | | -0.007 | | 0.012 | | | | | | -0.026 | | 0.015 | | 0.015 | | 0.015 | | -0.014 | | 0.015 | |
| rs10860295 | T | | | C | | | 0.006 | | 0.010 | | | | | 0.016 | | 0.012 | | | | | | 0.011 | | 0.015 | | 0.012 | | 0.015 | | -0.001 | | 0.015 | |
| rs1552759 | T | | | C | | | -0.005 | | 0.010 | | | | | -0.006 | | 0.012 | | | | | | -0.011 | | 0.015 | | -0.005 | | 0.016 | | -0.021 | | 0.016 | |
| rs12817542 | C | | | T | | | -0.017 | | 0.023 | | | | | -0.016 | | 0.027 | | | | | | 0.000 | | 0.034 | | -0.029 | | 0.036 | | -0.016 | | 0.036 | |
| rs61936936 | A | | | T | | | -0.027 | | 0.017 | | | | | -0.017 | | 0.020 | | | | | | -0.033 | | 0.025 | | 0.005 | | 0.026 | | -0.071 | | 0.026 | |
| rs7305424 | A | | | T | | | 0.002 | | 0.010 | | | | | 0.015 | | 0.012 | | | | | | 0.012 | | 0.015 | | 0.019 | | 0.016 | | -0.004 | | 0.016 | |
| rs12308065 | A | | | G | | | -0.011 | | 0.011 | | | | | -0.013 | | 0.013 | | | | | | -0.023 | | 0.016 | | 0.004 | | 0.016 | | -0.025 | | 0.016 | |
| rs28629903 | T | | | C | | | -0.006 | | 0.010 | | | | | -0.008 | | 0.012 | | | | | | -0.008 | | 0.015 | | -0.005 | | 0.015 | | 0.023 | | 0.015 | |
| rs7989098 | T | | | C | | | 0.007 | | 0.011 | | | | | 0.003 | | 0.014 | | | | | | 0.002 | | 0.017 | | -0.001 | | 0.018 | | 0.016 | | 0.018 | |
| rs9652090 | G | | | T | | | 0.023 | | 0.010 | | | | | 0.023 | | 0.012 | | | | | | 0.031 | | 0.015 | | 0.024 | | 0.015 | | 0.023 | | 0.015 | |
| rs1933437 | G | | | A | | | -0.011 | | 0.010 | | | | | -0.013 | | 0.012 | | | | | | 0.000 | | 0.015 | | -0.026 | | 0.015 | | 0.003 | | 0.015 | |
| rs9603697 | C | | | T | | | 0.011 | | 0.010 | | | | | 0.018 | | 0.012 | | | | | | 0.010 | | 0.016 | | 0.022 | | 0.016 | | 0.015 | | 0.016 | |
| rs9594686 | C | | | T | | | -0.001 | | 0.013 | | | | | 0.003 | | 0.016 | | | | | | 0.018 | | 0.020 | | -0.013 | | 0.020 | | -0.003 | | 0.020 | |
| rs12429545 | G | | | A | | | 0.005 | | 0.014 | | | | | 0.015 | | 0.017 | | | | | | 0.016 | | 0.022 | | 0.006 | | 0.022 | | 0.020 | | 0.023 | |
| rs9538141 | G | | | A | | | -0.009 | | 0.010 | | | | | -0.007 | | 0.012 | | | | | | -0.002 | | 0.015 | | -0.018 | | 0.015 | | -0.012 | | 0.015 | |
| rs1333010 | G | | | A | | | 0.017 | | 0.010 | | | | | 0.010 | | 0.012 | | | | | | 0.015 | | 0.015 | | 0.003 | | 0.016 | | 0.034 | | 0.016 | |
| rs1576655 | A | | | C | | | -0.017 | | 0.010 | | | | | -0.021 | | 0.012 | | | | | | -0.020 | | 0.015 | | -0.024 | | 0.016 | | -0.009 | | 0.016 | |
| rs61978655 | G | | | A | | | -0.029 | | 0.030 | | | | | -0.014 | | 0.030 | | | | | | 0.001 | | 0.038 | | -0.012 | | 0.039 | | -0.052 | | 0.038 | |
| rs7161424 | G | | | A | | | -0.008 | | 0.012 | | | | | -0.007 | | 0.012 | | | | | | -0.007 | | 0.015 | | -0.005 | | 0.015 | | -0.009 | | 0.015 | |
| rs1865719 | A | | | G | | | 0.011 | | 0.012 | | | | | 0.014 | | 0.012 | | | | | | 0.015 | | 0.015 | | 0.011 | | 0.016 | | 0.040 | | 0.016 | |
| rs10133279 | C | | | T | | | -0.003 | | 0.012 | | | | | 0.010 | | 0.012 | | | | | | 0.001 | | 0.015 | | 0.023 | | 0.016 | | -0.018 | | 0.016 | |
| rs7145052 | C | | | T | | | 0.004 | | 0.011 | | | | | 0.007 | | 0.012 | | | | | | -0.006 | | 0.015 | | 0.015 | | 0.015 | | 0.004 | | 0.015 | |
| rs7159126 | T | | | C | | | 0.013 | | 0.012 | | | | | -0.008 | | 0.013 | | | | | | -0.002 | | 0.016 | | -0.018 | | 0.017 | | 0.004 | | 0.017 | |
| rs78420139 | G | | | A | | | 0.047 | | 0.026 | | | | | 0.030 | | 0.027 | | | | | | 0.055 | | 0.034 | | 0.014 | | 0.035 | | 0.069 | | 0.035 | |
| rs12436513 | C | | | A | | | -0.009 | | 0.012 | | | | | 0.001 | | 0.012 | | | | | | 0.002 | | 0.015 | | 0.002 | | 0.016 | | -0.011 | | 0.016 | |
| rs824207 | A | | | G | | | 0.008 | | 0.010 | | | | | 0.006 | | 0.012 | | | | | | -0.004 | | 0.015 | | 0.012 | | 0.016 | | 0.008 | | 0.016 | |
| rs62048187 | G | | | C | | | -0.031 | | 0.011 | | | | | -0.035 | | 0.013 | | | | | | -0.036 | | 0.016 | | -0.037 | | 0.017 | | -0.029 | | 0.017 | |
| rs10519136 | C | | | T | | | -0.011 | | 0.010 | | | | | -0.020 | | 0.012 | | | | | | -0.018 | | 0.015 | | -0.025 | | 0.015 | | -0.004 | | 0.015 | |
| rs8030456 | C | | | T | | | 0.044 | | 0.011 | | | | | 0.044 | | 0.014 | | | | | | 0.058 | | 0.017 | | 0.028 | | 0.018 | | 0.011 | | 0.018 | |
| rs7162542 | C | | | G | | | -0.013 | | 0.010 | | | | | -0.017 | | 0.012 | | | | | | -0.017 | | 0.015 | | -0.014 | | 0.015 | | -0.012 | | 0.015 | |
| rs3817428 | C | | | G | | | -0.007 | | 0.011 | | | | | 0.001 | | 0.014 | | | | | | 0.011 | | 0.017 | | -0.011 | | 0.018 | | -0.019 | | 0.018 | |
| rs1000471 | C | | | T | | | 0.008 | | 0.012 | | | | | 0.004 | | 0.015 | | | | | | 0.005 | | 0.018 | | 0.003 | | 0.019 | | -0.003 | | 0.019 | |
| rs2970356 | C | | | G | | | -0.005 | | 0.011 | | | | | -0.016 | | 0.013 | | | | | | -0.001 | | 0.016 | | -0.022 | | 0.017 | | 0.009 | | 0.017 | |
| rs72755233 | G | | | A | | | 0.024 | | 0.018 | | | | | 0.030 | | 0.022 | | | | | | 0.043 | | 0.028 | | 0.025 | | 0.029 | | 0.032 | | 0.029 | |
| rs2238435 | C | | | G | | | -0.009 | | 0.010 | | | | | -0.004 | | 0.012 | | | | | | -0.004 | | 0.015 | | -0.012 | | 0.015 | | -0.011 | | 0.016 | |
| rs55880046 | T | | | G | | | -0.011 | | 0.014 | | | | | -0.018 | | 0.017 | | | | | | 0.002 | | 0.021 | | -0.040 | | 0.022 | | 0.013 | | 0.022 | |
| rs4432271 | C | | | T | | | -0.008 | | 0.014 | | | | | -0.004 | | 0.017 | | | | | | -0.006 | | 0.022 | | 0.014 | | 0.022 | | -0.001 | | 0.022 | |
| rs9922288 | A | | | G | | | -0.011 | | 0.012 | | | | | -0.020 | | 0.015 | | | | | | -0.016 | | 0.018 | | -0.018 | | 0.019 | | 0.018 | | 0.019 | |
| rs62037365 | C | | | G | | | -0.011 | | 0.010 | | | | | -0.017 | | 0.012 | | | | | | -0.022 | | 0.015 | | -0.016 | | 0.016 | | -0.007 | | 0.016 | |
| rs4889630 | T | | | C | | | 0.025 | | 0.012 | | | | | 0.019 | | 0.014 | | | | | | 0.021 | | 0.018 | | 0.009 | | 0.019 | | 0.024 | | 0.019 | |
| rs4783789 | T | | | C | | | -0.012 | | 0.011 | | | | | -0.008 | | 0.013 | | | | | | -0.017 | | 0.017 | | 0.004 | | 0.017 | | -0.017 | | 0.018 | |
| rs1421085 | T | | | C | | | 0.004 | | 0.010 | | | | | -0.003 | | 0.012 | | | | | | 0.000 | | 0.015 | | -0.008 | | 0.015 | | 0.008 | | 0.015 | |
| rs594585 | T | | | G | | | 0.020 | | 0.010 | | | | | 0.029 | | 0.012 | | | | | | 0.028 | | 0.015 | | 0.031 | | 0.015 | | 0.011 | | 0.015 | |
| rs117903946 | G | | | A | | | -0.001 | | 0.029 | | | | | 0.009 | | 0.034 | | | | | | -0.014 | | 0.043 | | 0.030 | | 0.044 | | -0.059 | | 0.043 | |
| rs7672 | C | | | G | | | -0.019 | | 0.011 | | | | | -0.013 | | 0.013 | | | | | | -0.004 | | 0.016 | | -0.010 | | 0.017 | | -0.011 | | 0.017 | |
| rs4985555 | A | | | G | | | 0.004 | | 0.010 | | | | | 0.008 | | 0.012 | | | | | | 0.014 | | 0.015 | | -0.003 | | 0.016 | | -0.001 | | 0.016 | |
| rs11642090 | T | | | C | | | 0.008 | | 0.010 | | | | | 0.004 | | 0.012 | | | | | | 0.010 | | 0.015 | | -0.006 | | 0.016 | | -0.002 | | 0.016 | |
| rs72819571 | G | | | T | | | 0.005 | | 0.010 | | | | | -0.001 | | 0.012 | | | | | | 0.011 | | 0.015 | | -0.004 | | 0.016 | | 0.006 | | 0.016 | |
| rs67603370 | G | | | A | | | 0.016 | | 0.019 | | | | | 0.023 | | 0.023 | | | | | | 0.025 | | 0.029 | | 0.030 | | 0.030 | | 0.042 | | 0.030 | |
| rs3815156 | A | | | G | | | -0.021 | | 0.013 | | | | | -0.032 | | 0.015 | | | | | | -0.032 | | 0.019 | | -0.039 | | 0.020 | | -0.018 | | 0.020 | |
| rs12601380 | A | | | C | | | 0.002 | | 0.010 | | | | | 0.003 | | 0.012 | | | | | | 0.017 | | 0.015 | | -0.011 | | 0.015 | | 0.016 | | 0.015 | |
| rs9299 | C | | | T | | | -0.003 | | 0.010 | | | | | -0.006 | | 0.012 | | | | | | -0.005 | | 0.015 | | -0.004 | | 0.016 | | 0.009 | | 0.016 | |
| rs17637472 | G | | | A | | | 0.028 | | 0.010 | | | | | 0.024 | | 0.012 | | | | | | 0.013 | | 0.015 | | 0.044 | | 0.015 | | 0.028 | | 0.015 | |
| rs7217460 | G | | | A | | | 0.017 | | 0.011 | | | | | 0.015 | | 0.014 | | | | | | -0.001 | | 0.017 | | 0.027 | | 0.018 | | 0.034 | | 0.018 | |
| rs12941038 | C | | | T | | | 0.012 | | 0.011 | | | | | 0.007 | | 0.014 | | | | | | 0.013 | | 0.017 | | 0.004 | | 0.018 | | 0.035 | | 0.018 | |
| rs2246623 | C | | | T | | | -0.026 | | 0.010 | | | | | -0.022 | | 0.012 | | | | | | -0.009 | | 0.015 | | -0.036 | | 0.016 | | -0.044 | | 0.016 | |
| rs11150745 | A | | | G | | | -0.007 | | 0.010 | | | | | -0.008 | | 0.012 | | | | | | 0.000 | | 0.016 | | -0.008 | | 0.016 | | 0.007 | | 0.016 | |
| rs7503580 | C | | | T | | | -0.005 | | 0.013 | | | | | -0.019 | | 0.016 | | | | | | -0.014 | | 0.020 | | -0.030 | | 0.021 | | 0.025 | | 0.021 | |
| rs1013737 | G | | | C | | | 0.001 | | 0.010 | | | | | -0.007 | | 0.012 | | | | | | 0.003 | | 0.015 | | -0.027 | | 0.015 | | -0.021 | | 0.015 | |
| rs1808579 | C | | | T | | | 0.020 | | 0.010 | | | | | 0.012 | | 0.012 | | | | | | 0.006 | | 0.015 | | 0.020 | | 0.015 | | 0.030 | | 0.015 | |
| rs7237444 | G | | | A | | | -0.017 | | 0.011 | | | | | -0.021 | | 0.013 | | | | | | -0.035 | | 0.017 | | -0.006 | | 0.017 | | -0.023 | | 0.017 | |
| rs7239114 | G | | | A | | | -0.001 | | 0.010 | | | | | -0.008 | | 0.012 | | | | | | -0.009 | | 0.015 | | -0.004 | | 0.015 | | 0.012 | | 0.015 | |
| rs68015088 | G | | | A | | | 0.007 | | 0.010 | | | | | 0.016 | | 0.012 | | | | | | 0.014 | | 0.015 | | 0.026 | | 0.016 | | -0.012 | | 0.016 | |
| rs12606230 | T | | | C | | | 0.002 | | 0.011 | | | | | 0.001 | | 0.014 | | | | | | -0.003 | | 0.018 | | 0.016 | | 0.018 | | 0.017 | | 0.018 | |
| rs663129 | G | | | A | | | -0.019 | | 0.011 | | | | | -0.010 | | 0.014 | | | | | | -0.007 | | 0.017 | | -0.010 | | 0.018 | | -0.025 | | 0.018 | |
| rs113728099 | G | | | A | | | 0.024 | | 0.032 | | | | | 0.022 | | 0.038 | | | | | | -0.011 | | 0.048 | | 0.038 | | 0.050 | | 0.028 | | 0.050 | |
| rs8096658 | C | | | G | | | -0.008 | | 0.011 | | | | | -0.021 | | 0.013 | | | | | | -0.002 | | 0.016 | | -0.032 | | 0.017 | | -0.012 | | 0.017 | |
| rs62621197 | C | | | T | | | -0.035 | | 0.029 | | | | | -0.025 | | 0.034 | | | | | | 0.022 | | 0.044 | | -0.079 | | 0.044 | | -0.020 | | 0.046 | |
| rs4545941 | T | | | C | | | 0.008 | | 0.015 | | | | | 0.005 | | 0.018 | | | | | | 0.023 | | 0.022 | | -0.021 | | 0.023 | | -0.008 | | 0.023 | |
| rs116399833 | C | | | A | | | -0.001 | | 0.012 | | | | | -0.005 | | 0.014 | | | | | | 0.012 | | 0.018 | | -0.014 | | 0.019 | | 0.011 | | 0.019 | |
| rs4808961 | C | | | G | | | -0.021 | | 0.010 | | | | | -0.016 | | 0.012 | | | | | | -0.014 | | 0.015 | | -0.019 | | 0.016 | | -0.022 | | 0.016 | |
| rs3810304 | A | | | G | | | -0.004 | | 0.012 | | | | | 0.003 | | 0.014 | | | | | | 0.013 | | 0.018 | | -0.011 | | 0.018 | | -0.004 | | 0.018 | |
| rs1800437 | G | | | C | | | -0.005 | | 0.012 | | | | | -0.002 | | 0.014 | | | | | | -0.002 | | 0.018 | | 0.001 | | 0.018 | | -0.007 | | 0.019 | |
| rs3810291 | G | | | A | | | 0.000 | | 0.010 | | | | | 0.003 | | 0.013 | | | | | | -0.008 | | 0.016 | | 0.017 | | 0.016 | | -0.005 | | 0.016 | |
| rs601338 | G | | | A | | | -0.018 | | 0.010 | | | | | -0.028 | | 0.012 | | | | | | -0.044 | | 0.015 | | -0.010 | | 0.015 | | -0.008 | | 0.015 | |
| rs16996644 | C | | | G | | | 0.009 | | 0.015 | | | | | 0.015 | | 0.018 | | | | | | 0.026 | | 0.023 | | 0.013 | | 0.023 | | 0.044 | | 0.023 | |
| rs947088 | G | | | T | | | -0.002 | | 0.011 | | | | | 0.001 | | 0.013 | | | | | | -0.007 | | 0.016 | | 0.012 | | 0.017 | | -0.010 | | 0.017 | |
| rs8117463 | G | | | A | | | 0.005 | | 0.011 | | | | | -0.002 | | 0.012 | | | | | | -0.016 | | 0.016 | | 0.011 | | 0.016 | | 0.025 | | 0.016 | |
| rs73085586 | G | | | A | | | 0.021 | | 0.012 | | | | | 0.026 | | 0.014 | | | | | | 0.021 | | 0.018 | | 0.034 | | 0.019 | | -0.006 | | 0.019 | |
| rs2281148 | T | | | C | | | -0.006 | | 0.011 | | | | | -0.014 | | 0.014 | | | | | | -0.024 | | 0.017 | | -0.004 | | 0.018 | | 0.014 | | 0.018 | |
| rs2207894 | C | | | T | | | -0.005 | | 0.012 | | | | | 0.002 | | 0.014 | | | | | | 0.000 | | 0.018 | | 0.002 | | 0.019 | | -0.025 | | 0.019 | |
| rs117455294 | C | | | A | | | 0.034 | | 0.026 | | | | | 0.056 | | 0.031 | | | | | | 0.039 | | 0.039 | | 0.080 | | 0.041 | | -0.017 | | 0.040 | |
| rs8130408 | A | | | C | | | -0.005 | | 0.012 | | | | | 0.007 | | 0.014 | | | | | | -0.006 | | 0.018 | | 0.027 | | 0.018 | | -0.026 | | 0.019 | |
| rs13047416 | C | | | G | | | -0.017 | | 0.010 | | | | | -0.003 | | 0.012 | | | | | | 0.015 | | 0.015 | | -0.019 | | 0.016 | | -0.038 | | 0.016 | |
| rs78907487 | A | | | C | | | -0.009 | | 0.014 | | | | | -0.016 | | 0.016 | | | | | | -0.005 | | 0.020 | | -0.033 | | 0.021 | | 0.038 | | 0.021 | |
| rs9610387 | G | | | A | | | 0.011 | | 0.018 | | | | | 0.015 | | 0.021 | | | | | | 0.040 | | 0.027 | | -0.007 | | 0.028 | | -0.006 | | 0.028 | |
| rs6001872 | A | | | G | | | 0.017 | | 0.010 | | | | | 0.008 | | 0.012 | | | | | | 0.004 | | 0.015 | | 0.012 | | 0.016 | | 0.021 | | 0.016 | |
| rs9611560 | T | | | C | | | -0.018 | | 0.011 | | | | | -0.023 | | 0.014 | | | | | | -0.022 | | 0.017 | | -0.015 | | 0.018 | | -0.032 | | 0.018 | |
| Early life body size SNPs (men only) | | | | | | | | | | | | | | | | | | | | | | | | | | | | | | | | |
| SNP | EA | | | OA | | beta_ca | | | se_ca | | | | beta_co | | se_co | | | | | | beta_prox | | se_prox | | beta_dist | | se_dist | | beta_re | | se_re | |
| rs12140153 | G | | | T | | 0.000 | | | 0.026 | | | | 0.012 | | 0.032 | | | | | | -0.008 | | 0.041 | | 0.027 | | 0.040 | | -0.034 | | 0.038 | |
| rs2012697 | T | | | C | | 0.025 | | | 0.014 | | | | 0.023 | | 0.017 | | | | | | 0.008 | | 0.022 | | 0.023 | | 0.022 | | 0.033 | | 0.021 | |
| rs4650277 | A | | | G | | 0.010 | | | 0.014 | | | | 0.023 | | 0.017 | | | | | | 0.028 | | 0.022 | | 0.022 | | 0.021 | | 0.016 | | 0.021 | |
| rs7550711 | C | | | T | | 0.002 | | | 0.040 | | | | -0.025 | | 0.049 | | | | | | -0.008 | | 0.063 | | 0.008 | | 0.062 | | -0.030 | | 0.059 | |
| rs539515 | A | | | C | | 0.013 | | | 0.017 | | | | 0.000 | | 0.022 | | | | | | -0.024 | | 0.028 | | 0.009 | | 0.027 | | 0.031 | | 0.026 | |
| rs78444298 | G | | | A | | -0.004 | | | 0.060 | | | | 0.023 | | 0.073 | | | | | | 0.055 | | 0.095 | | 0.037 | | 0.093 | | 0.009 | | 0.090 | |
| rs1772143 | T | | | A | | 0.036 | | | 0.014 | | | | 0.041 | | 0.017 | | | | | | 0.028 | | 0.022 | | 0.049 | | 0.021 | | 0.032 | | 0.021 | |
| rs77165542 | C | | | T | | 0.033 | | | 0.043 | | | | 0.007 | | 0.051 | | | | | | 0.046 | | 0.067 | | -0.015 | | 0.065 | | 0.051 | | 0.065 | |
| rs6749422 | C | | | G | | 0.002 | | | 0.014 | | | | -0.016 | | 0.017 | | | | | | -0.030 | | 0.021 | | -0.008 | | 0.021 | | 0.001 | | 0.020 | |
| rs2862874 | G | | | T | | 0.033 | | | 0.014 | | | | 0.028 | | 0.017 | | | | | | 0.001 | | 0.022 | | 0.052 | | 0.021 | | 0.019 | | 0.021 | |
| rs10496885 | G | | | A | | -0.007 | | | 0.018 | | | | 0.021 | | 0.022 | | | | | | 0.058 | | 0.028 | | -0.011 | | 0.027 | | -0.013 | | 0.026 | |
| rs115319174 | G | | | C | | -0.023 | | | 0.031 | | | | -0.018 | | 0.037 | | | | | | 0.007 | | 0.048 | | -0.022 | | 0.046 | | -0.046 | | 0.045 | |
| rs9880272 | T | | | G | | 0.007 | | | 0.016 | | | | 0.016 | | 0.019 | | | | | | 0.013 | | 0.025 | | 0.021 | | 0.024 | | 0.011 | | 0.024 | |
| rs34722008 | G | | | A | | -0.017 | | | 0.015 | | | | -0.006 | | 0.018 | | | | | | 0.007 | | 0.023 | | -0.007 | | 0.023 | | -0.001 | | 0.022 | |
| rs10938398 | G | | | A | | 0.017 | | | 0.014 | | | | 0.014 | | 0.017 | | | | | | 0.015 | | 0.022 | | 0.014 | | 0.021 | | 0.013 | | 0.020 | |
| rs13107325 | C | | | T | | -0.009 | | | 0.026 | | | | -0.003 | | 0.032 | | | | | | -0.012 | | 0.040 | | -0.009 | | 0.039 | | 0.000 | | 0.039 | |
| rs3212519 | A | | | G | | -0.010 | | | 0.026 | | | | -0.028 | | 0.032 | | | | | | -0.018 | | 0.041 | | -0.051 | | 0.039 | | 0.025 | | 0.039 | |
| rs75577466 | G | | | C | | 0.003 | | | 0.019 | | | | 0.017 | | 0.023 | | | | | | 0.014 | | 0.029 | | 0.029 | | 0.028 | | -0.034 | | 0.027 | |
| rs25842 | C | | | T | | 0.001 | | | 0.015 | | | | 0.003 | | 0.019 | | | | | | -0.004 | | 0.024 | | 0.000 | | 0.024 | | -0.011 | | 0.023 | |
| rs55654862 | A | | | G | | -0.015 | | | 0.017 | | | | -0.007 | | 0.020 | | | | | | 0.034 | | 0.026 | | -0.052 | | 0.025 | | -0.021 | | 0.024 | |
| rs4958361 | C | | | G | | 0.001 | | | 0.014 | | | | 0.010 | | 0.017 | | | | | | 0.015 | | 0.021 | | 0.002 | | 0.021 | | -0.004 | | 0.020 | |
| rs2240071 | C | | | G | | -0.012 | | | 0.016 | | | | -0.013 | | 0.020 | | | | | | -0.012 | | 0.025 | | -0.019 | | 0.025 | | 0.001 | | 0.024 | |
| rs62405422 | T | | | C | | 0.005 | | | 0.017 | | | | 0.006 | | 0.021 | | | | | | 0.018 | | 0.028 | | -0.006 | | 0.027 | | 0.005 | | 0.026 | |
| rs1775255 | G | | | T | | -0.030 | | | 0.014 | | | | -0.029 | | 0.017 | | | | | | -0.027 | | 0.022 | | -0.030 | | 0.021 | | -0.025 | | 0.020 | |
| rs115597956 | G | | | A | | 0.043 | | | 0.026 | | | | 0.049 | | 0.034 | | | | | | 0.064 | | 0.043 | | 0.045 | | 0.042 | | 0.075 | | 0.042 | |
| rs12110721 | G | | | A | | -0.049 | | | 0.019 | | | | -0.050 | | 0.023 | | | | | | -0.068 | | 0.030 | | -0.034 | | 0.029 | | -0.062 | | 0.028 | |
| rs2693560 | A | | | G | | 0.015 | | | 0.014 | | | | 0.007 | | 0.018 | | | | | | 0.018 | | 0.023 | | 0.005 | | 0.022 | | 0.016 | | 0.021 | |
| rs1452991 | G | | | A | | 0.003 | | | 0.014 | | | | -0.009 | | 0.017 | | | | | | -0.011 | | 0.022 | | -0.005 | | 0.022 | | 0.006 | | 0.021 | |
| rs16120 | A | | | G | | 0.002 | | | 0.014 | | | | 0.017 | | 0.017 | | | | | | 0.032 | | 0.021 | | 0.012 | | 0.021 | | -0.010 | | 0.020 | |
| rs7796922 | A | | | G | | -0.006 | | | 0.024 | | | | -0.011 | | 0.029 | | | | | | -0.011 | | 0.038 | | -0.002 | | 0.037 | | 0.011 | | 0.036 | |
| rs2979139 | A | | | G | | -0.016 | | | 0.015 | | | | -0.019 | | 0.018 | | | | | | -0.031 | | 0.024 | | -0.014 | | 0.023 | | -0.016 | | 0.022 | |
| rs12674871 | C | | | T | | 0.003 | | | 0.017 | | | | -0.012 | | 0.022 | | | | | | -0.011 | | 0.028 | | -0.006 | | 0.027 | | 0.026 | | 0.027 | |
| rs10504620 | T | | | C | | 0.008 | | | 0.015 | | | | -0.009 | | 0.018 | | | | | | -0.010 | | 0.024 | | -0.011 | | 0.023 | | 0.036 | | 0.022 | |
| rs10968101 | G | | | A | | -0.014 | | | 0.014 | | | | -0.025 | | 0.017 | | | | | | -0.029 | | 0.022 | | -0.016 | | 0.021 | | -0.007 | | 0.021 | |
| rs11790060 | T | | | C | | 0.015 | | | 0.014 | | | | 0.008 | | 0.018 | | | | | | 0.030 | | 0.023 | | -0.009 | | 0.022 | | -0.006 | | 0.022 | |
| rs41310284 | C | | | A | | -0.002 | | | 0.024 | | | | -0.002 | | 0.029 | | | | | | 0.001 | | 0.038 | | -0.010 | | 0.037 | | 0.005 | | 0.036 | |
| rs11030102 | C | | | G | | 0.022 | | | 0.016 | | | | 0.023 | | 0.020 | | | | | | 0.007 | | 0.025 | | 0.026 | | 0.025 | | 0.018 | | 0.024 | |
| rs3817334 | C | | | T | | -0.016 | | | 0.014 | | | | -0.007 | | 0.017 | | | | | | -0.010 | | 0.022 | | -0.013 | | 0.021 | | -0.013 | | 0.021 | |
| rs10896348 | T | | | C | | -0.008 | | | 0.015 | | | | -0.011 | | 0.019 | | | | | | -0.033 | | 0.024 | | 0.016 | | 0.024 | | -0.019 | | 0.023 | |
| rs11218734 | A | | | G | | 0.003 | | | 0.016 | | | | 0.006 | | 0.020 | | | | | | 0.031 | | 0.025 | | -0.013 | | 0.025 | | 0.003 | | 0.024 | |
| rs7978659 | G | | | T | | 0.021 | | | 0.015 | | | | 0.024 | | 0.018 | | | | | | 0.048 | | 0.023 | | 0.003 | | 0.022 | | 0.030 | | 0.022 | |
| rs7132908 | G | | | A | | 0.001 | | | 0.014 | | | | 0.005 | | 0.017 | | | | | | 0.006 | | 0.022 | | 0.004 | | 0.022 | | 0.018 | | 0.021 | |
| rs7306710 | T | | | C | | -0.010 | | | 0.014 | | | | -0.005 | | 0.017 | | | | | | -0.032 | | 0.022 | | 0.023 | | 0.021 | | -0.016 | | 0.021 | |
| rs7316962 | A | | | G | | 0.006 | | | 0.014 | | | | 0.002 | | 0.017 | | | | | | 0.005 | | 0.022 | | -0.005 | | 0.021 | | 0.022 | | 0.020 | |
| rs9568868 | G | | | T | | 0.009 | | | 0.020 | | | | 0.039 | | 0.025 | | | | | | 0.063 | | 0.032 | | 0.014 | | 0.031 | | -0.007 | | 0.030 | |
| rs1576655 | A | | | C | | -0.018 | | | 0.014 | | | | -0.016 | | 0.018 | | | | | | -0.014 | | 0.023 | | -0.021 | | 0.022 | | -0.018 | | 0.021 | |
| rs61978655 | G | | | A | | -0.022 | | | 0.036 | | | | -0.037 | | 0.051 | | | | | | -0.054 | | 0.066 | | -0.037 | | 0.066 | | -0.015 | | 0.066 | |
| rs2143975 | C | | | G | | 0.021 | | | 0.014 | | | | 0.033 | | 0.020 | | | | | | 0.042 | | 0.025 | | 0.025 | | 0.025 | | 0.037 | | 0.025 | |
| rs7159126 | T | | | C | | -0.002 | | | 0.015 | | | | -0.003 | | 0.018 | | | | | | 0.015 | | 0.024 | | -0.023 | | 0.023 | | -0.017 | | 0.022 | |
| rs3784710 | T | | | C | | 0.063 | | | 0.016 | | | | 0.061 | | 0.020 | | | | | | 0.074 | | 0.025 | | 0.061 | | 0.025 | | 0.022 | | 0.024 | |
| rs7190603 | T | | | C | | -0.015 | | | 0.020 | | | | -0.006 | | 0.024 | | | | | | 0.016 | | 0.031 | | -0.024 | | 0.030 | | -0.017 | | 0.029 | |
| rs56094641 | A | | | G | | 0.001 | | | 0.014 | | | | 0.002 | | 0.017 | | | | | | -0.005 | | 0.022 | | 0.010 | | 0.021 | | 0.003 | | 0.020 | |
| rs17637472 | G | | | A | | 0.006 | | | 0.014 | | | | 0.008 | | 0.017 | | | | | | 0.002 | | 0.022 | | 0.022 | | 0.021 | | 0.006 | | 0.021 | |
| rs2250081 | G | | | A | | -0.032 | | | 0.014 | | | | -0.037 | | 0.017 | | | | | | -0.032 | | 0.022 | | -0.043 | | 0.022 | | -0.032 | | 0.021 | |
| rs7239114 | G | | | A | | -0.014 | | | 0.014 | | | | -0.017 | | 0.017 | | | | | | -0.016 | | 0.022 | | -0.014 | | 0.021 | | -0.001 | | 0.021 | |
| rs3764516 | A | | | C | | 0.002 | | | 0.016 | | | | -0.002 | | 0.020 | | | | | | -0.004 | | 0.026 | | -0.009 | | 0.025 | | -0.019 | | 0.025 | |
| rs663129 | G | | | A | | -0.019 | | | 0.016 | | | | -0.020 | | 0.020 | | | | | | -0.021 | | 0.025 | | -0.012 | | 0.025 | | -0.018 | | 0.024 | |
| rs1532127 | G | | | A | | 0.020 | | | 0.015 | | | | 0.027 | | 0.018 | | | | | | 0.017 | | 0.023 | | 0.044 | | 0.023 | | 0.012 | | 0.022 | |
| rs1321434 | A | | | G | | 0.050 | | | 0.014 | | | | 0.055 | | 0.017 | | | | | | 0.032 | | 0.022 | | 0.075 | | 0.021 | | 0.058 | | 0.021 | |
| rs73898513 | C | | | T | | 0.052 | | | 0.022 | | | | 0.058 | | 0.027 | | | | | | 0.060 | | 0.034 | | 0.074 | | 0.034 | | 0.063 | | 0.032 | |
| Early life body size SNPs (women only) | | | | | | | | | | | | | | | | | | | | | | | | | | | | | | | | |
| SNP | EA | OA | | | beta_ca | | | | se_ca | | beta_co | | | | se_co | | | | beta_prox | | | | se_prox | | beta_dist | | se_dist | | beta_re | | se_re | |
| rs212540 | C | T | | | 0.004 | | | | 0.014 | | 0.005 | | | | 0.016 | | | | 0.016 | | | | 0.020 | | -0.024 | | 0.022 | | -0.005 | | 0.023 | |
| rs582220 | A | G | | | 0.002 | | | | 0.014 | | -0.007 | | | | 0.016 | | | | 0.001 | | | | 0.020 | | -0.018 | | 0.022 | | 0.034 | | 0.023 | |
| rs12140153 | G | T | | | 0.028 | | | | 0.027 | | 0.038 | | | | 0.031 | | | | 0.080 | | | | 0.039 | | -0.038 | | 0.042 | | -0.026 | | 0.044 | |
| rs2767486 | A | G | | | 0.005 | | | | 0.017 | | 0.011 | | | | 0.020 | | | | 0.013 | | | | 0.024 | | 0.021 | | 0.027 | | 0.008 | | 0.028 | |
| rs7522014 | A | G | | | -0.009 | | | | 0.015 | | 0.008 | | | | 0.018 | | | | 0.002 | | | | 0.022 | | 0.017 | | 0.024 | | -0.035 | | 0.025 | |
| rs11209943 | A | G | | | 0.006 | | | | 0.014 | | -0.003 | | | | 0.017 | | | | 0.009 | | | | 0.020 | | -0.019 | | 0.023 | | 0.023 | | 0.024 | |
| rs12042908 | A | G | | | -0.010 | | | | 0.014 | | -0.001 | | | | 0.016 | | | | -0.003 | | | | 0.020 | | 0.011 | | 0.022 | | -0.006 | | 0.023 | |
| rs41279738 | T | G | | | 0.002 | | | | 0.042 | | 0.002 | | | | 0.049 | | | | -0.015 | | | | 0.060 | | -0.020 | | 0.066 | | 0.029 | | 0.069 | |
| rs543874 | A | G | | | -0.017 | | | | 0.017 | | 0.003 | | | | 0.021 | | | | 0.006 | | | | 0.025 | | 0.007 | | 0.028 | | -0.038 | | 0.029 | |
| rs10798139 | C | T | | | 0.010 | | | | 0.016 | | 0.010 | | | | 0.020 | | | | -0.008 | | | | 0.024 | | 0.024 | | 0.027 | | -0.014 | | 0.028 | |
| rs815339 | T | A | | | 0.006 | | | | 0.014 | | 0.011 | | | | 0.016 | | | | 0.005 | | | | 0.020 | | 0.023 | | 0.022 | | -0.002 | | 0.023 | |
| rs4971239 | G | A | | | -0.001 | | | | 0.022 | | -0.009 | | | | 0.027 | | | | -0.017 | | | | 0.033 | | 0.019 | | 0.037 | | 0.021 | | 0.038 | |
| rs62106258 | T | C | | | 0.041 | | | | 0.036 | | 0.060 | | | | 0.042 | | | | 0.027 | | | | 0.051 | | 0.121 | | 0.058 | | 0.000 | | 0.059 | |
| rs12992672 | G | A | | | -0.008 | | | | 0.018 | | -0.006 | | | | 0.021 | | | | -0.030 | | | | 0.026 | | 0.023 | | 0.028 | | 0.001 | | 0.030 | |
| rs6738433 | G | C | | | -0.020 | | | | 0.014 | | -0.017 | | | | 0.016 | | | | -0.019 | | | | 0.020 | | -0.023 | | 0.022 | | -0.031 | | 0.023 | |
| rs146910503 | G | A | | | 0.119 | | | | 0.053 | | 0.114 | | | | 0.062 | | | | 0.103 | | | | 0.076 | | 0.114 | | 0.086 | | -0.010 | | 0.085 | |
| rs1446725 | T | G | | | -0.007 | | | | 0.014 | | -0.019 | | | | 0.016 | | | | -0.039 | | | | 0.020 | | -0.005 | | 0.022 | | 0.020 | | 0.023 | |
| rs1483153 | C | T | | | 0.018 | | | | 0.016 | | 0.027 | | | | 0.020 | | | | 0.010 | | | | 0.024 | | 0.057 | | 0.026 | | 0.037 | | 0.028 | |
| rs55959207 | A | C | | | 0.031 | | | | 0.014 | | 0.052 | | | | 0.017 | | | | 0.032 | | | | 0.020 | | 0.068 | | 0.023 | | 0.016 | | 0.024 | |
| rs17464221 | C | T | | | 0.009 | | | | 0.015 | | 0.001 | | | | 0.017 | | | | 0.017 | | | | 0.021 | | -0.011 | | 0.024 | | 0.018 | | 0.025 | |
| rs115319174 | G | C | | | -0.017 | | | | 0.031 | | -0.001 | | | | 0.036 | | | | 0.031 | | | | 0.044 | | -0.053 | | 0.048 | | -0.024 | | 0.051 | |
| rs2594989 | C | T | | | 0.003 | | | | 0.018 | | -0.002 | | | | 0.021 | | | | -0.006 | | | | 0.026 | | -0.005 | | 0.028 | | 0.030 | | 0.029 | |
| rs754635 | C | G | | | 0.004 | | | | 0.021 | | -0.007 | | | | 0.026 | | | | -0.020 | | | | 0.031 | | 0.001 | | 0.035 | | 0.018 | | 0.036 | |
| rs2034963 | G | C | | | 0.018 | | | | 0.015 | | 0.022 | | | | 0.017 | | | | 0.009 | | | | 0.021 | | 0.039 | | 0.023 | | 0.031 | | 0.025 | |
| rs2629881 | C | T | | | 0.005 | | | | 0.017 | | -0.003 | | | | 0.020 | | | | -0.012 | | | | 0.025 | | 0.002 | | 0.028 | | -0.014 | | 0.029 | |
| rs79569013 | T | G | | | -0.009 | | | | 0.020 | | -0.016 | | | | 0.023 | | | | -0.012 | | | | 0.028 | | -0.024 | | 0.031 | | 0.011 | | 0.033 | |
| rs818219 | T | C | | | -0.017 | | | | 0.014 | | -0.018 | | | | 0.016 | | | | 0.000 | | | | 0.020 | | -0.035 | | 0.022 | | 0.028 | | 0.023 | |
| rs2735556 | T | C | | | -0.031 | | | | 0.023 | | -0.025 | | | | 0.027 | | | | 0.001 | | | | 0.033 | | -0.060 | | 0.036 | | -0.019 | | 0.039 | |
| rs1199328 | G | A | | | 0.029 | | | | 0.017 | | 0.046 | | | | 0.020 | | | | 0.040 | | | | 0.024 | | 0.050 | | 0.027 | | -0.031 | | 0.029 | |
| rs76152047 | A | G | | | -0.025 | | | | 0.026 | | -0.024 | | | | 0.033 | | | | -0.077 | | | | 0.039 | | 0.051 | | 0.045 | | -0.081 | | 0.046 | |
| rs7656673 | A | G | | | 0.000 | | | | 0.014 | | -0.001 | | | | 0.017 | | | | 0.006 | | | | 0.020 | | -0.010 | | 0.022 | | -0.017 | | 0.023 | |
| rs12641981 | C | T | | | -0.019 | | | | 0.014 | | -0.025 | | | | 0.016 | | | | -0.027 | | | | 0.020 | | -0.013 | | 0.022 | | -0.011 | | 0.023 | |
| rs1349641 | T | G | | | -0.003 | | | | 0.014 | | -0.020 | | | | 0.017 | | | | -0.030 | | | | 0.020 | | -0.015 | | 0.022 | | 0.024 | | 0.023 | |
| rs7377083 | C | A | | | 0.014 | | | | 0.014 | | 0.005 | | | | 0.017 | | | | -0.002 | | | | 0.021 | | 0.016 | | 0.023 | | 0.027 | | 0.024 | |
| rs3936511 | A | G | | | -0.035 | | | | 0.018 | | -0.049 | | | | 0.021 | | | | -0.023 | | | | 0.025 | | -0.072 | | 0.027 | | 0.006 | | 0.029 | |
| rs10050620 | C | T | | | 0.002 | | | | 0.014 | | 0.003 | | | | 0.017 | | | | 0.000 | | | | 0.021 | | 0.004 | | 0.023 | | 0.014 | | 0.024 | |
| rs13190020 | G | A | | | -0.012 | | | | 0.015 | | -0.028 | | | | 0.017 | | | | -0.016 | | | | 0.021 | | -0.047 | | 0.023 | | -0.033 | | 0.024 | |
| rs9293494 | T | G | | | -0.037 | | | | 0.016 | | -0.039 | | | | 0.019 | | | | -0.045 | | | | 0.023 | | -0.036 | | 0.025 | | -0.025 | | 0.026 | |
| rs4235642 | A | G | | | 0.021 | | | | 0.014 | | 0.020 | | | | 0.017 | | | | 0.031 | | | | 0.020 | | 0.015 | | 0.022 | | 0.016 | | 0.024 | |
| rs6860760 | A | G | | | -0.003 | | | | 0.014 | | -0.007 | | | | 0.016 | | | | -0.020 | | | | 0.020 | | 0.012 | | 0.022 | | -0.017 | | 0.023 | |
| rs815610 | C | G | | | 0.015 | | | | 0.014 | | 0.021 | | | | 0.016 | | | | 0.011 | | | | 0.020 | | 0.015 | | 0.022 | | -0.028 | | 0.023 | |
| rs12214497 | G | T | | | 0.016 | | | | 0.014 | | 0.025 | | | | 0.017 | | | | 0.038 | | | | 0.021 | | -0.008 | | 0.023 | | -0.015 | | 0.024 | |
| rs75782365 | T | G | | | -0.092 | | | | 0.026 | | -0.094 | | | | 0.031 | | | | -0.142 | | | | 0.037 | | -0.034 | | 0.042 | | -0.119 | | 0.043 | |
| rs34196306 | G | C | | | -0.083 | | | | 0.027 | | -0.084 | | | | 0.031 | | | | -0.140 | | | | 0.037 | | -0.014 | | 0.042 | | -0.103 | | 0.043 | |
| rs3749971 | G | A | | | -0.093 | | | | 0.024 | | -0.091 | | | | 0.028 | | | | -0.133 | | | | 0.034 | | -0.043 | | 0.038 | | -0.112 | | 0.039 | |
| rs3131934 | T | C | | | -0.031 | | | | 0.020 | | -0.048 | | | | 0.023 | | | | -0.062 | | | | 0.028 | | -0.027 | | 0.032 | | -0.088 | | 0.033 | |
| rs9268235 | C | T | | | -0.043 | | | | 0.023 | | -0.041 | | | | 0.027 | | | | -0.055 | | | | 0.033 | | -0.021 | | 0.037 | | -0.107 | | 0.038 | |
| rs141127771 | G | A | | | -0.035 | | | | 0.025 | | -0.017 | | | | 0.030 | | | | 0.001 | | | | 0.037 | | -0.048 | | 0.040 | | -0.027 | | 0.041 | |
| rs3798544 | G | A | | | -0.003 | | | | 0.019 | | -0.014 | | | | 0.023 | | | | 0.004 | | | | 0.028 | | -0.035 | | 0.031 | | 0.012 | | 0.033 | |
| rs2206277 | C | T | | | 0.019 | | | | 0.017 | | 0.012 | | | | 0.021 | | | | 0.026 | | | | 0.026 | | -0.002 | | 0.028 | | 0.060 | | 0.030 | |
| rs1775255 | G | T | | | 0.005 | | | | 0.014 | | 0.005 | | | | 0.016 | | | | -0.001 | | | | 0.020 | | 0.020 | | 0.022 | | 0.007 | | 0.023 | |
| rs12110721 | G | A | | | -0.008 | | | | 0.019 | | -0.033 | | | | 0.022 | | | | -0.053 | | | | 0.027 | | 0.001 | | 0.030 | | 0.014 | | 0.032 | |
| rs34260097 | T | G | | | -0.031 | | | | 0.016 | | -0.035 | | | | 0.019 | | | | -0.035 | | | | 0.024 | | -0.045 | | 0.026 | | -0.017 | | 0.027 | |
| rs7759938 | C | T | | | 0.014 | | | | 0.015 | | 0.010 | | | | 0.017 | | | | 0.010 | | | | 0.021 | | 0.017 | | 0.023 | | -0.002 | | 0.025 | |
| rs796915 | C | G | | | 0.021 | | | | 0.015 | | 0.023 | | | | 0.017 | | | | 0.016 | | | | 0.021 | | 0.031 | | 0.023 | | 0.028 | | 0.025 | |
| rs62425122 | G | A | | | -0.022 | | | | 0.015 | | -0.039 | | | | 0.018 | | | | -0.037 | | | | 0.022 | | -0.048 | | 0.024 | | -0.051 | | 0.025 | |
| rs983949 | T | G | | | 0.009 | | | | 0.015 | | 0.006 | | | | 0.018 | | | | -0.002 | | | | 0.022 | | 0.013 | | 0.024 | | 0.035 | | 0.025 | |
| rs7808296 | C | T | | | -0.013 | | | | 0.015 | | -0.017 | | | | 0.017 | | | | -0.008 | | | | 0.021 | | -0.042 | | 0.024 | | -0.055 | | 0.025 | |
| rs6979832 | A | G | | | -0.006 | | | | 0.014 | | -0.016 | | | | 0.016 | | | | -0.029 | | | | 0.020 | | -0.006 | | 0.022 | | 0.023 | | 0.023 | |
| rs13233916 | C | G | | | 0.024 | | | | 0.025 | | 0.019 | | | | 0.031 | | | | 0.021 | | | | 0.038 | | 0.032 | | 0.043 | | 0.096 | | 0.045 | |
| rs7005216 | G | C | | | 0.019 | | | | 0.015 | | -0.001 | | | | 0.017 | | | | -0.030 | | | | 0.021 | | 0.045 | | 0.024 | | 0.041 | | 0.025 | |
| rs351776 | A | C | | | -0.034 | | | | 0.014 | | -0.043 | | | | 0.016 | | | | -0.050 | | | | 0.020 | | -0.046 | | 0.022 | | -0.065 | | 0.023 | |
| rs62515439 | C | T | | | -0.006 | | | | 0.014 | | 0.007 | | | | 0.017 | | | | -0.008 | | | | 0.021 | | 0.033 | | 0.023 | | -0.014 | | 0.024 | |
| rs13254613 | A | C | | | 0.011 | | | | 0.014 | | 0.043 | | | | 0.017 | | | | 0.065 | | | | 0.021 | | 0.021 | | 0.023 | | 0.000 | | 0.024 | |
| rs2126474 | G | T | | | 0.006 | | | | 0.014 | | 0.009 | | | | 0.016 | | | | 0.021 | | | | 0.020 | | -0.011 | | 0.022 | | 0.026 | | 0.023 | |
| rs10821163 | G | C | | | 0.009 | | | | 0.014 | | 0.021 | | | | 0.017 | | | | 0.062 | | | | 0.021 | | -0.025 | | 0.023 | | -0.023 | | 0.024 | |
| rs957512 | T | C | | | 0.003 | | | | 0.014 | | 0.013 | | | | 0.017 | | | | 0.004 | | | | 0.021 | | 0.019 | | 0.023 | | -0.011 | | 0.024 | |
| rs2275241 | G | A | | | -0.013 | | | | 0.015 | | -0.017 | | | | 0.017 | | | | -0.012 | | | | 0.021 | | -0.023 | | 0.023 | | 0.003 | | 0.024 | |
| rs7084503 | T | C | | | 0.025 | | | | 0.014 | | 0.018 | | | | 0.016 | | | | 0.001 | | | | 0.020 | | 0.043 | | 0.022 | | 0.048 | | 0.023 | |
| rs76971642 | T | C | | | 0.034 | | | | 0.033 | | 0.053 | | | | 0.039 | | | | 0.078 | | | | 0.048 | | 0.022 | | 0.053 | | 0.039 | | 0.055 | |
| rs962369 | T | C | | | 0.005 | | | | 0.015 | | -0.010 | | | | 0.018 | | | | -0.013 | | | | 0.022 | | -0.015 | | 0.024 | | 0.028 | | 0.026 | |
| rs661878 | A | G | | | -0.012 | | | | 0.019 | | -0.014 | | | | 0.023 | | | | -0.018 | | | | 0.028 | | -0.008 | | 0.031 | | -0.013 | | 0.033 | |
| rs11039307 | C | T | | | 0.002 | | | | 0.014 | | 0.001 | | | | 0.016 | | | | -0.011 | | | | 0.020 | | 0.011 | | 0.022 | | 0.001 | | 0.023 | |
| rs678653 | C | G | | | -0.011 | | | | 0.015 | | -0.009 | | | | 0.017 | | | | 0.003 | | | | 0.021 | | -0.021 | | 0.023 | | -0.013 | | 0.024 | |
| rs11215403 | G | A | | | -0.045 | | | | 0.016 | | -0.032 | | | | 0.019 | | | | -0.026 | | | | 0.024 | | -0.023 | | 0.026 | | -0.061 | | 0.027 | |
| rs11611246 | G | T | | | -0.017 | | | | 0.017 | | -0.028 | | | | 0.020 | | | | -0.043 | | | | 0.025 | | -0.004 | | 0.027 | | 0.040 | | 0.029 | |
| rs2187642 | A | C | | | 0.012 | | | | 0.014 | | 0.030 | | | | 0.017 | | | | 0.027 | | | | 0.021 | | 0.036 | | 0.023 | | 0.010 | | 0.024 | |
| rs10876457 | G | A | | | 0.004 | | | | 0.017 | | 0.016 | | | | 0.019 | | | | 0.014 | | | | 0.024 | | 0.016 | | 0.026 | | -0.005 | | 0.027 | |
| rs10783302 | G | T | | | 0.052 | | | | 0.014 | | 0.046 | | | | 0.017 | | | | 0.037 | | | | 0.021 | | 0.062 | | 0.023 | | 0.038 | | 0.024 | |
| rs7132908 | G | A | | | -0.021 | | | | 0.014 | | -0.015 | | | | 0.017 | | | | 0.001 | | | | 0.021 | | -0.032 | | 0.023 | | -0.033 | | 0.024 | |
| rs76919525 | T | A | | | -0.047 | | | | 0.015 | | -0.039 | | | | 0.017 | | | | -0.035 | | | | 0.021 | | -0.045 | | 0.023 | | -0.071 | | 0.025 | |
| rs78607331 | C | T | | | -0.016 | | | | 0.041 | | -0.008 | | | | 0.048 | | | | -0.041 | | | | 0.058 | | 0.028 | | 0.065 | | -0.028 | | 0.066 | |
| rs10784514 | C | T | | | 0.020 | | | | 0.016 | | 0.022 | | | | 0.018 | | | | 0.022 | | | | 0.023 | | 0.038 | | 0.025 | | 0.033 | | 0.026 | |
| rs2364232 | A | C | | | 0.005 | | | | 0.016 | | -0.015 | | | | 0.018 | | | | 0.010 | | | | 0.023 | | -0.039 | | 0.025 | | 0.020 | | 0.026 | |
| rs12309017 | G | T | | | -0.016 | | | | 0.016 | | -0.023 | | | | 0.019 | | | | -0.054 | | | | 0.023 | | 0.011 | | 0.026 | | 0.015 | | 0.027 | |
| rs11111647 | G | A | | | -0.007 | | | | 0.017 | | -0.018 | | | | 0.020 | | | | -0.016 | | | | 0.025 | | -0.020 | | 0.027 | | 0.000 | | 0.029 | |
| rs7305424 | A | T | | | 0.018 | | | | 0.014 | | 0.029 | | | | 0.017 | | | | 0.014 | | | | 0.021 | | 0.043 | | 0.023 | | -0.012 | | 0.024 | |
| rs35202265 | C | T | | | 0.010 | | | | 0.014 | | 0.003 | | | | 0.017 | | | | 0.020 | | | | 0.021 | | -0.008 | | 0.023 | | 0.045 | | 0.024 | |
| rs9551428 | C | T | | | -0.026 | | | | 0.014 | | -0.020 | | | | 0.016 | | | | -0.009 | | | | 0.020 | | -0.032 | | 0.022 | | -0.017 | | 0.023 | |
| rs1336486 | T | G | | | 0.015 | | | | 0.014 | | 0.015 | | | | 0.017 | | | | 0.002 | | | | 0.021 | | 0.028 | | 0.023 | | 0.024 | | 0.025 | |
| rs4477562 | C | T | | | 0.003 | | | | 0.020 | | -0.007 | | | | 0.024 | | | | -0.017 | | | | 0.029 | | -0.002 | | 0.032 | | 0.049 | | 0.034 | |
| rs9317002 | C | A | | | -0.009 | | | | 0.014 | | -0.008 | | | | 0.016 | | | | 0.002 | | | | 0.020 | | -0.023 | | 0.022 | | -0.006 | | 0.023 | |
| rs58681688 | C | G | | | -0.017 | | | | 0.017 | | -0.025 | | | | 0.021 | | | | -0.015 | | | | 0.026 | | -0.037 | | 0.028 | | -0.039 | | 0.030 | |
| rs9540493 | A | G | | | 0.019 | | | | 0.014 | | 0.017 | | | | 0.016 | | | | 0.020 | | | | 0.020 | | 0.012 | | 0.022 | | 0.050 | | 0.023 | |
| rs1576655 | A | C | | | -0.017 | | | | 0.014 | | -0.026 | | | | 0.017 | | | | -0.025 | | | | 0.021 | | -0.025 | | 0.023 | | 0.006 | | 0.024 | |
| rs61980008 | G | A | | | -0.032 | | | | 0.036 | | 0.011 | | | | 0.051 | | | | 0.048 | | | | 0.062 | | -0.030 | | 0.067 | | 0.008 | | 0.073 | |
| rs1865719 | A | G | | | 0.006 | | | | 0.014 | | 0.006 | | | | 0.018 | | | | 0.025 | | | | 0.024 | | 0.017 | | 0.027 | | 0.059 | | 0.029 | |
| rs8030456 | C | T | | | 0.030 | | | | 0.016 | | 0.039 | | | | 0.019 | | | | 0.052 | | | | 0.023 | | 0.006 | | 0.026 | | 0.001 | | 0.027 | |
| rs4932430 | A | C | | | 0.016 | | | | 0.014 | | 0.015 | | | | 0.017 | | | | 0.026 | | | | 0.020 | | 0.005 | | 0.023 | | 0.004 | | 0.024 | |
| rs2970356 | C | G | | | 0.002 | | | | 0.015 | | -0.006 | | | | 0.018 | | | | 0.008 | | | | 0.022 | | -0.010 | | 0.025 | | 0.019 | | 0.026 | |
| rs72755233 | G | A | | | 0.020 | | | | 0.026 | | 0.017 | | | | 0.030 | | | | 0.002 | | | | 0.037 | | 0.041 | | 0.042 | | 0.014 | | 0.043 | |
| rs2531991 | G | A | | | -0.026 | | | | 0.016 | | -0.024 | | | | 0.019 | | | | -0.015 | | | | 0.023 | | -0.043 | | 0.025 | | -0.040 | | 0.026 | |
| rs148965598 | A | G | | | -0.015 | | | | 0.021 | | -0.036 | | | | 0.024 | | | | -0.014 | | | | 0.030 | | -0.066 | | 0.033 | | 0.033 | | 0.035 | |
| rs4432271 | C | T | | | -0.018 | | | | 0.020 | | 0.000 | | | | 0.024 | | | | -0.004 | | | | 0.029 | | 0.023 | | 0.032 | | 0.007 | | 0.034 | |
| rs7189927 | T | C | | | 0.012 | | | | 0.015 | | 0.019 | | | | 0.017 | | | | 0.027 | | | | 0.021 | | 0.009 | | 0.023 | | 0.010 | | 0.024 | |
| rs4889630 | T | C | | | 0.049 | | | | 0.017 | | 0.045 | | | | 0.020 | | | | 0.049 | | | | 0.024 | | 0.034 | | 0.027 | | 0.031 | | 0.028 | |
| rs1421085 | T | C | | | 0.005 | | | | 0.014 | | -0.011 | | | | 0.016 | | | | 0.003 | | | | 0.020 | | -0.032 | | 0.022 | | 0.017 | | 0.023 | |
| rs11863799 | C | T | | | -0.001 | | | | 0.015 | | -0.016 | | | | 0.017 | | | | -0.003 | | | | 0.021 | | -0.036 | | 0.023 | | 0.003 | | 0.024 | |
| rs34229857 | C | T | | | -0.046 | | | | 0.045 | | -0.002 | | | | 0.052 | | | | -0.024 | | | | 0.063 | | 0.023 | | 0.072 | | -0.136 | | 0.070 | |
| rs11642090 | T | C | | | -0.001 | | | | 0.014 | | 0.005 | | | | 0.017 | | | | 0.012 | | | | 0.021 | | 0.003 | | 0.023 | | -0.008 | | 0.024 | |
| rs242922 | A | C | | | 0.009 | | | | 0.014 | | 0.011 | | | | 0.017 | | | | -0.013 | | | | 0.021 | | 0.031 | | 0.023 | | -0.004 | | 0.024 | |
| rs999493 | G | A | | | 0.008 | | | | 0.014 | | 0.017 | | | | 0.016 | | | | 0.026 | | | | 0.020 | | 0.016 | | 0.022 | | 0.009 | | 0.023 | |
| rs12185242 | A | C | | | 0.029 | | | | 0.014 | | 0.029 | | | | 0.016 | | | | 0.022 | | | | 0.020 | | 0.043 | | 0.022 | | 0.025 | | 0.023 | |
| rs11150745 | A | G | | | -0.022 | | | | 0.015 | | -0.020 | | | | 0.017 | | | | -0.008 | | | | 0.021 | | -0.032 | | 0.023 | | -0.018 | | 0.025 | |
| rs1013737 | G | C | | | 0.020 | | | | 0.014 | | 0.014 | | | | 0.016 | | | | 0.044 | | | | 0.020 | | -0.030 | | 0.022 | | 0.000 | | 0.023 | |
| rs303753 | G | A | | | 0.001 | | | | 0.015 | | -0.005 | | | | 0.017 | | | | -0.006 | | | | 0.021 | | -0.008 | | 0.023 | | 0.007 | | 0.024 | |
| rs7239114 | G | A | | | 0.010 | | | | 0.014 | | 0.001 | | | | 0.016 | | | | -0.002 | | | | 0.020 | | 0.006 | | 0.022 | | 0.029 | | 0.023 | |
| rs12606230 | T | C | | | 0.009 | | | | 0.016 | | 0.003 | | | | 0.019 | | | | -0.006 | | | | 0.024 | | 0.024 | | 0.026 | | 0.014 | | 0.028 | |
| rs2168711 | T | C | | | -0.022 | | | | 0.016 | | -0.005 | | | | 0.019 | | | | -0.002 | | | | 0.023 | | -0.008 | | 0.025 | | -0.036 | | 0.027 | |
| rs17066856 | T | C | | | -0.002 | | | | 0.023 | | 0.003 | | | | 0.028 | | | | -0.016 | | | | 0.034 | | 0.043 | | 0.038 | | -0.009 | | 0.039 | |
| rs16982345 | G | A | | | 0.008 | | | | 0.016 | | 0.007 | | | | 0.019 | | | | 0.038 | | | | 0.023 | | -0.019 | | 0.025 | | 0.006 | | 0.027 | |
| rs3810304 | A | G | | | -0.006 | | | | 0.016 | | -0.009 | | | | 0.020 | | | | 0.001 | | | | 0.024 | | -0.026 | | 0.027 | | -0.008 | | 0.028 | |
| rs4805881 | A | C | | | 0.020 | | | | 0.014 | | 0.032 | | | | 0.017 | | | | 0.039 | | | | 0.021 | | 0.012 | | 0.023 | | -0.010 | | 0.024 | |
| rs3810291 | G | A | | | -0.021 | | | | 0.015 | | -0.019 | | | | 0.018 | | | | -0.023 | | | | 0.021 | | -0.012 | | 0.024 | | -0.016 | | 0.025 | |
| rs633372 | G | A | | | -0.027 | | | | 0.014 | | -0.041 | | | | 0.016 | | | | -0.058 | | | | 0.020 | | -0.026 | | 0.022 | | -0.031 | | 0.023 | |
| rs994308 | C | T | | | 0.091 | | | | 0.014 | | 0.082 | | | | 0.016 | | | | 0.070 | | | | 0.020 | | 0.097 | | 0.022 | | 0.098 | | 0.023 | |
| rs7268466 | C | T | | | -0.009 | | | | 0.020 | | 0.006 | | | | 0.024 | | | | 0.034 | | | | 0.029 | | -0.023 | | 0.032 | | 0.026 | | 0.033 | |
| rs947088 | G | T | | | -0.007 | | | | 0.015 | | -0.005 | | | | 0.018 | | | | -0.023 | | | | 0.022 | | 0.021 | | 0.024 | | -0.011 | | 0.025 | |
| rs66469746 | C | A | | | -0.011 | | | | 0.015 | | -0.011 | | | | 0.018 | | | | -0.006 | | | | 0.022 | | -0.020 | | 0.024 | | -0.040 | | 0.025 | |
| rs4817973 | G | A | | | -0.010 | | | | 0.014 | | -0.003 | | | | 0.017 | | | | 0.022 | | | | 0.021 | | -0.033 | | 0.023 | | -0.019 | | 0.024 | |
| rs6001872 | A | G | | | 0.024 | | | | 0.014 | | 0.021 | | | | 0.017 | | | | 0.017 | | | | 0.021 | | 0.034 | | 0.023 | | 0.043 | | 0.024 | |
| Adult body size SNPs (overall) | | | | | | | | | | | | | | | | | | | | | | | | | | | | | | | | |
| SNP | EA | OA | | | beta_ca | | | | se_ca | | beta_co | | | | se_co | | | | beta_prox | | | | se_prox | | beta_dist | | se_dist | | beta_re | | se_re | |
| rs4648450 | C | A | | | 0.024 | | | | 0.010 | | 0.016 | | | | 0.012 | | | | 0.018 | | | | 0.015 | | 0.021 | | 0.015 | | 0.031 | | 0.015 | |
| rs2076363 | C | G | | | -0.024 | | | | 0.010 | | -0.022 | | | | 0.012 | | | | -0.026 | | | | 0.015 | | -0.024 | | 0.016 | | -0.027 | | 0.016 | |
| rs4908677 | C | T | | | -0.004 | | | | 0.010 | | -0.001 | | | | 0.012 | | | | -0.003 | | | | 0.015 | | 0.000 | | 0.015 | | -0.014 | | 0.015 | |
| rs78886584 | A | G | | | -0.010 | | | | 0.011 | | -0.009 | | | | 0.013 | | | | -0.008 | | | | 0.017 | | -0.008 | | 0.017 | | -0.006 | | 0.018 | |
| rs10799778 | T | G | | | 0.027 | | | | 0.013 | | 0.035 | | | | 0.015 | | | | 0.039 | | | | 0.019 | | 0.031 | | 0.020 | | 0.043 | | 0.020 | |
| rs7511698 | C | T | | | -0.019 | | | | 0.011 | | -0.031 | | | | 0.013 | | | | -0.034 | | | | 0.016 | | -0.029 | | 0.017 | | -0.013 | | 0.017 | |
| rs945211 | G | C | | | 0.004 | | | | 0.010 | | 0.011 | | | | 0.012 | | | | 0.026 | | | | 0.015 | | -0.011 | | 0.016 | | 0.009 | | 0.016 | |
| rs3737992 | G | A | | | 0.005 | | | | 0.012 | | 0.002 | | | | 0.015 | | | | -0.002 | | | | 0.019 | | 0.002 | | 0.019 | | -0.013 | | 0.020 | |
| rs12031634 | G | A | | | 0.016 | | | | 0.011 | | 0.009 | | | | 0.013 | | | | 0.030 | | | | 0.016 | | -0.010 | | 0.017 | | 0.009 | | 0.017 | |
| rs116195355 | C | A | | | 0.011 | | | | 0.032 | | 0.002 | | | | 0.037 | | | | -0.039 | | | | 0.046 | | 0.027 | | 0.049 | | 0.023 | | 0.049 | |
| rs2744801 | C | T | | | 0.011 | | | | 0.010 | | 0.012 | | | | 0.012 | | | | -0.009 | | | | 0.016 | | 0.029 | | 0.016 | | 0.013 | | 0.016 | |
| rs4660586 | C | T | | | -0.010 | | | | 0.011 | | -0.017 | | | | 0.013 | | | | -0.024 | | | | 0.017 | | -0.008 | | 0.017 | | -0.007 | | 0.017 | |
| rs6669341 | A | G | | | -0.012 | | | | 0.010 | | -0.018 | | | | 0.012 | | | | -0.017 | | | | 0.015 | | -0.014 | | 0.015 | | -0.033 | | 0.015 | |
| rs1167311 | G | A | | | 0.011 | | | | 0.011 | | 0.010 | | | | 0.013 | | | | 0.009 | | | | 0.016 | | 0.006 | | 0.017 | | 0.016 | | 0.017 | |
| rs630602 | G | C | | | -0.004 | | | | 0.010 | | -0.014 | | | | 0.012 | | | | -0.007 | | | | 0.015 | | -0.017 | | 0.016 | | 0.015 | | 0.016 | |
| rs12140153 | G | T | | | 0.015 | | | | 0.019 | | 0.026 | | | | 0.022 | | | | 0.041 | | | | 0.028 | | -0.003 | | 0.029 | | -0.024 | | 0.029 | |
| rs11208659 | T | C | | | 0.004 | | | | 0.016 | | -0.005 | | | | 0.019 | | | | -0.001 | | | | 0.025 | | 0.002 | | 0.025 | | 0.036 | | 0.026 | |
| rs7519259 | G | A | | | 0.010 | | | | 0.010 | | 0.005 | | | | 0.012 | | | | 0.009 | | | | 0.015 | | 0.004 | | 0.015 | | 0.001 | | 0.015 | |
| rs2613499 | A | G | | | 0.013 | | | | 0.013 | | 0.023 | | | | 0.015 | | | | 0.015 | | | | 0.019 | | 0.039 | | 0.020 | | 0.001 | | 0.020 | |
| rs7553158 | G | A | | | 0.001 | | | | 0.010 | | 0.011 | | | | 0.012 | | | | 0.009 | | | | 0.015 | | 0.019 | | 0.015 | | 0.006 | | 0.015 | |
| rs115778101 | T | C | | | 0.029 | | | | 0.024 | | 0.038 | | | | 0.029 | | | | 0.079 | | | | 0.037 | | -0.006 | | 0.037 | | 0.046 | | 0.038 | |
| rs34517439 | C | A | | | -0.007 | | | | 0.017 | | -0.016 | | | | 0.020 | | | | -0.011 | | | | 0.025 | | -0.023 | | 0.026 | | -0.015 | | 0.026 | |
| rs651533 | T | A | | | 0.011 | | | | 0.011 | | 0.023 | | | | 0.014 | | | | 0.020 | | | | 0.017 | | 0.017 | | 0.018 | | 0.011 | | 0.018 | |
| rs28726372 | T | C | | | 0.016 | | | | 0.011 | | 0.023 | | | | 0.013 | | | | 0.034 | | | | 0.017 | | 0.014 | | 0.017 | | 0.003 | | 0.017 | |
| rs7548936 | G | C | | | 0.001 | | | | 0.010 | | 0.003 | | | | 0.012 | | | | 0.008 | | | | 0.015 | | -0.004 | | 0.015 | | 0.025 | | 0.016 | |
| rs6679458 | G | T | | | 0.001 | | | | 0.010 | | -0.002 | | | | 0.012 | | | | 0.005 | | | | 0.015 | | -0.008 | | 0.015 | | 0.013 | | 0.015 | |
| rs12072739 | A | G | | | 0.008 | | | | 0.012 | | 0.008 | | | | 0.014 | | | | 0.015 | | | | 0.018 | | 0.005 | | 0.018 | | 0.014 | | 0.018 | |
| rs41279738 | T | G | | | 0.003 | | | | 0.029 | | -0.012 | | | | 0.034 | | | | -0.015 | | | | 0.044 | | -0.002 | | 0.045 | | -0.004 | | 0.045 | |
| rs12033257 | A | G | | | 0.015 | | | | 0.010 | | 0.011 | | | | 0.012 | | | | 0.016 | | | | 0.016 | | 0.001 | | 0.016 | | 0.016 | | 0.016 | |
| rs7549358 | G | C | | | 0.015 | | | | 0.010 | | 0.010 | | | | 0.012 | | | | 0.008 | | | | 0.015 | | 0.017 | | 0.016 | | 0.020 | | 0.016 | |
| rs1409158 | C | T | | | -0.015 | | | | 0.011 | | -0.018 | | | | 0.013 | | | | -0.015 | | | | 0.017 | | -0.022 | | 0.017 | | -0.011 | | 0.018 | |
| rs142315514 | C | A | | | 0.001 | | | | 0.030 | | -0.001 | | | | 0.036 | | | | 0.021 | | | | 0.045 | | -0.017 | | 0.046 | | 0.011 | | 0.047 | |
| rs10749659 | C | T | | | -0.022 | | | | 0.011 | | -0.024 | | | | 0.014 | | | | -0.035 | | | | 0.017 | | -0.020 | | 0.018 | | -0.020 | | 0.018 | |
| rs3753639 | T | C | | | -0.001 | | | | 0.012 | | -0.003 | | | | 0.014 | | | | 0.005 | | | | 0.017 | | -0.017 | | 0.018 | | -0.024 | | 0.018 | |
| rs61813324 | C | T | | | -0.047 | | | | 0.017 | | -0.045 | | | | 0.020 | | | | -0.041 | | | | 0.025 | | -0.060 | | 0.026 | | -0.047 | | 0.027 | |
| rs1778830 | G | A | | | -0.021 | | | | 0.010 | | -0.020 | | | | 0.012 | | | | -0.028 | | | | 0.015 | | -0.011 | | 0.016 | | -0.024 | | 0.016 | |
| rs4916229 | C | G | | | -0.022 | | | | 0.017 | | -0.023 | | | | 0.020 | | | | -0.049 | | | | 0.025 | | 0.002 | | 0.026 | | -0.025 | | 0.026 | |
| rs148137538 | A | G | | | -0.049 | | | | 0.036 | | -0.058 | | | | 0.042 | | | | -0.004 | | | | 0.054 | | -0.100 | | 0.055 | | -0.033 | | 0.056 | |
| rs77560793 | G | A | | | -0.027 | | | | 0.028 | | -0.005 | | | | 0.034 | | | | 0.009 | | | | 0.042 | | -0.041 | | 0.043 | | -0.105 | | 0.043 | |
| rs539515 | A | C | | | -0.003 | | | | 0.012 | | 0.002 | | | | 0.015 | | | | -0.004 | | | | 0.019 | | 0.005 | | 0.019 | | 0.002 | | 0.019 | |
| rs9425634 | T | C | | | 0.009 | | | | 0.010 | | 0.009 | | | | 0.012 | | | | 0.015 | | | | 0.015 | | 0.000 | | 0.015 | | 0.003 | | 0.015 | |
| rs815163 | T | C | | | -0.015 | | | | 0.010 | | -0.015 | | | | 0.012 | | | | -0.012 | | | | 0.015 | | -0.021 | | 0.015 | | -0.018 | | 0.015 | |
| rs76702514 | C | G | | | 0.016 | | | | 0.012 | | 0.023 | | | | 0.015 | | | | 0.035 | | | | 0.018 | | 0.011 | | 0.019 | | 0.007 | | 0.019 | |
| rs2678204 | T | G | | | -0.027 | | | | 0.010 | | -0.035 | | | | 0.012 | | | | -0.045 | | | | 0.016 | | -0.029 | | 0.016 | | -0.005 | | 0.016 | |
| rs4971239 | G | A | | | -0.002 | | | | 0.016 | | -0.008 | | | | 0.019 | | | | -0.047 | | | | 0.024 | | 0.044 | | 0.025 | | 0.033 | | 0.025 | |
| rs7539903 | T | A | | | 0.007 | | | | 0.010 | | -0.001 | | | | 0.012 | | | | 0.002 | | | | 0.015 | | -0.011 | | 0.015 | | 0.004 | | 0.016 | |
| rs78508049 | T | C | | | -0.016 | | | | 0.012 | | -0.018 | | | | 0.015 | | | | -0.010 | | | | 0.019 | | -0.032 | | 0.019 | | -0.027 | | 0.019 | |
| rs12037905 | C | T | | | 0.005 | | | | 0.010 | | 0.001 | | | | 0.012 | | | | -0.009 | | | | 0.015 | | 0.013 | | 0.015 | | 0.008 | | 0.015 | |
| rs7518221 | T | C | | | 0.009 | | | | 0.010 | | 0.012 | | | | 0.012 | | | | 0.017 | | | | 0.015 | | 0.005 | | 0.016 | | -0.020 | | 0.016 | |
| rs10779835 | T | C | | | 0.002 | | | | 0.010 | | -0.008 | | | | 0.012 | | | | 0.002 | | | | 0.015 | | -0.018 | | 0.015 | | 0.009 | | 0.015 | |
| rs10927006 | T | C | | | 0.000 | | | | 0.014 | | -0.003 | | | | 0.017 | | | | 0.008 | | | | 0.021 | | -0.015 | | 0.021 | | -0.002 | | 0.022 | |
| rs4658403 | C | T | | | -0.001 | | | | 0.013 | | 0.001 | | | | 0.015 | | | | 0.011 | | | | 0.019 | | -0.005 | | 0.020 | | 0.011 | | 0.020 | |
| rs6752378 | C | A | | | -0.004 | | | | 0.010 | | -0.010 | | | | 0.012 | | | | -0.016 | | | | 0.015 | | -0.007 | | 0.015 | | -0.013 | | 0.015 | |
| rs1631026 | C | T | | | -0.024 | | | | 0.010 | | -0.027 | | | | 0.012 | | | | -0.033 | | | | 0.015 | | -0.022 | | 0.015 | | -0.027 | | 0.015 | |
| rs10204994 | G | A | | | 0.007 | | | | 0.012 | | 0.012 | | | | 0.014 | | | | -0.002 | | | | 0.017 | | 0.022 | | 0.018 | | 0.010 | | 0.018 | |
| rs10185199 | G | A | | | -0.009 | | | | 0.011 | | -0.008 | | | | 0.013 | | | | -0.008 | | | | 0.017 | | 0.005 | | 0.017 | | -0.006 | | 0.017 | |
| rs10169594 | T | C | | | -0.006 | | | | 0.010 | | -0.006 | | | | 0.012 | | | | 0.007 | | | | 0.015 | | -0.022 | | 0.016 | | -0.008 | | 0.016 | |
| rs35809007 | G | A | | | -0.001 | | | | 0.010 | | -0.007 | | | | 0.012 | | | | -0.021 | | | | 0.015 | | 0.002 | | 0.016 | | -0.004 | | 0.016 | |
| rs72618637 | T | A | | | -0.008 | | | | 0.012 | | -0.023 | | | | 0.015 | | | | -0.023 | | | | 0.019 | | -0.021 | | 0.019 | | -0.025 | | 0.020 | |
| rs6761463 | G | C | | | 0.000 | | | | 0.013 | | 0.008 | | | | 0.016 | | | | -0.007 | | | | 0.020 | | 0.035 | | 0.021 | | -0.010 | | 0.021 | |
| rs59428052 | A | G | | | 0.015 | | | | 0.014 | | 0.018 | | | | 0.017 | | | | 0.011 | | | | 0.021 | | 0.021 | | 0.022 | | 0.010 | | 0.022 | |
| rs7601895 | C | G | | | 0.010 | | | | 0.011 | | 0.013 | | | | 0.013 | | | | 0.022 | | | | 0.016 | | 0.003 | | 0.017 | | 0.019 | | 0.017 | |
| rs4671328 | T | G | | | -0.007 | | | | 0.010 | | -0.007 | | | | 0.012 | | | | -0.006 | | | | 0.015 | | -0.008 | | 0.015 | | 0.013 | | 0.015 | |
| rs4672338 | C | T | | | 0.006 | | | | 0.010 | | 0.009 | | | | 0.012 | | | | 0.018 | | | | 0.016 | | 0.000 | | 0.016 | | -0.007 | | 0.016 | |
| rs12477088 | T | C | | | -0.010 | | | | 0.010 | | -0.016 | | | | 0.012 | | | | -0.028 | | | | 0.015 | | 0.002 | | 0.015 | | -0.031 | | 0.015 | |
| rs6752979 | G | A | | | 0.009 | | | | 0.010 | | 0.009 | | | | 0.012 | | | | -0.012 | | | | 0.016 | | 0.024 | | 0.016 | | -0.008 | | 0.016 | |
| rs396354 | T | C | | | 0.008 | | | | 0.011 | | -0.001 | | | | 0.013 | | | | 0.004 | | | | 0.016 | | -0.009 | | 0.017 | | 0.009 | | 0.017 | |
| rs11691869 | C | A | | | 0.026 | | | | 0.010 | | 0.025 | | | | 0.012 | | | | 0.012 | | | | 0.016 | | 0.038 | | 0.016 | | 0.033 | | 0.016 | |
| rs1451533 | G | A | | | 0.009 | | | | 0.011 | | 0.002 | | | | 0.013 | | | | -0.008 | | | | 0.016 | | 0.023 | | 0.017 | | 0.013 | | 0.017 | |
| rs72851476 | A | C | | | -0.006 | | | | 0.015 | | -0.014 | | | | 0.017 | | | | 0.000 | | | | 0.022 | | -0.032 | | 0.023 | | 0.009 | | 0.023 | |
| rs62171698 | C | A | | | -0.007 | | | | 0.014 | | -0.005 | | | | 0.016 | | | | 0.002 | | | | 0.021 | | -0.015 | | 0.021 | | -0.007 | | 0.022 | |
| rs75706763 | A | G | | | -0.016 | | | | 0.022 | | 0.004 | | | | 0.026 | | | | -0.014 | | | | 0.032 | | 0.002 | | 0.033 | | -0.070 | | 0.033 | |
| rs409696 | G | A | | | 0.014 | | | | 0.010 | | 0.018 | | | | 0.012 | | | | 0.013 | | | | 0.015 | | 0.022 | | 0.015 | | 0.006 | | 0.015 | |
| rs12692596 | C | T | | | -0.013 | | | | 0.010 | | -0.017 | | | | 0.012 | | | | 0.003 | | | | 0.015 | | -0.037 | | 0.016 | | -0.010 | | 0.016 | |
| rs12477385 | G | T | | | 0.003 | | | | 0.012 | | -0.004 | | | | 0.014 | | | | -0.008 | | | | 0.018 | | 0.003 | | 0.018 | | 0.017 | | 0.018 | |
| rs788163 | A | C | | | 0.017 | | | | 0.011 | | 0.012 | | | | 0.013 | | | | 0.004 | | | | 0.016 | | 0.017 | | 0.017 | | 0.006 | | 0.017 | |
| rs72917533 | T | C | | | 0.004 | | | | 0.013 | | -0.006 | | | | 0.015 | | | | 0.003 | | | | 0.019 | | -0.012 | | 0.020 | | 0.013 | | 0.020 | |
| rs7570446 | C | A | | | -0.002 | | | | 0.010 | | -0.003 | | | | 0.012 | | | | 0.012 | | | | 0.015 | | -0.018 | | 0.015 | | -0.004 | | 0.015 | |
| rs1704190 | G | A | | | 0.022 | | | | 0.010 | | 0.032 | | | | 0.012 | | | | 0.031 | | | | 0.015 | | 0.032 | | 0.016 | | 0.017 | | 0.016 | |
| rs4482463 | C | A | | | 0.001 | | | | 0.017 | | 0.006 | | | | 0.021 | | | | -0.006 | | | | 0.027 | | 0.009 | | 0.027 | | -0.018 | | 0.028 | |
| rs79675564 | C | A | | | -0.004 | | | | 0.020 | | -0.011 | | | | 0.024 | | | | -0.010 | | | | 0.030 | | -0.009 | | 0.031 | | -0.019 | | 0.031 | |
| rs13427822 | A | G | | | 0.024 | | | | 0.011 | | 0.030 | | | | 0.013 | | | | 0.030 | | | | 0.016 | | 0.035 | | 0.017 | | 0.014 | | 0.017 | |
| rs55658481 | G | A | | | 0.013 | | | | 0.010 | | 0.010 | | | | 0.012 | | | | 0.003 | | | | 0.016 | | 0.013 | | 0.016 | | 0.035 | | 0.016 | |
| rs2433733 | G | A | | | 0.000 | | | | 0.010 | | -0.001 | | | | 0.012 | | | | -0.001 | | | | 0.015 | | -0.007 | | 0.016 | | 0.003 | | 0.016 | |
| rs4663213 | G | A | | | 0.015 | | | | 0.012 | | 0.021 | | | | 0.014 | | | | 0.035 | | | | 0.017 | | 0.004 | | 0.018 | | 0.028 | | 0.018 | |
| rs112380819 | G | A | | | 0.007 | | | | 0.016 | | 0.011 | | | | 0.019 | | | | 0.017 | | | | 0.024 | | 0.009 | | 0.024 | | 0.004 | | 0.024 | |
| rs34373881 | G | A | | | 0.001 | | | | 0.011 | | -0.013 | | | | 0.013 | | | | 0.009 | | | | 0.017 | | -0.034 | | 0.017 | | -0.006 | | 0.017 | |
| rs7619139 | T | A | | | 0.001 | | | | 0.010 | | 0.002 | | | | 0.012 | | | | -0.005 | | | | 0.015 | | 0.007 | | 0.015 | | -0.012 | | 0.015 | |
| rs80082536 | A | G | | | 0.006 | | | | 0.016 | | 0.007 | | | | 0.019 | | | | -0.004 | | | | 0.024 | | 0.014 | | 0.025 | | 0.034 | | 0.025 | |
| rs111768603 | G | T | | | 0.012 | | | | 0.015 | | 0.019 | | | | 0.019 | | | | 0.008 | | | | 0.024 | | 0.026 | | 0.024 | | 0.026 | | 0.024 | |
| rs28350 | A | G | | | -0.004 | | | | 0.013 | | 0.007 | | | | 0.015 | | | | -0.012 | | | | 0.019 | | 0.037 | | 0.019 | | -0.025 | | 0.020 | |
| rs78517245 | T | C | | | 0.001 | | | | 0.047 | | 0.004 | | | | 0.056 | | | | 0.040 | | | | 0.072 | | -0.076 | | 0.074 | | -0.085 | | 0.073 | |
| rs113706999 | T | A | | | 0.002 | | | | 0.039 | | 0.024 | | | | 0.047 | | | | 0.000 | | | | 0.058 | | 0.068 | | 0.062 | | -0.002 | | 0.062 | |
| rs9852062 | T | A | | | -0.003 | | | | 0.010 | | 0.001 | | | | 0.012 | | | | 0.003 | | | | 0.015 | | -0.007 | | 0.015 | | 0.012 | | 0.015 | |
| rs113569731 | C | A | | | 0.024 | | | | 0.020 | | 0.036 | | | | 0.023 | | | | 0.036 | | | | 0.030 | | 0.056 | | 0.031 | | 0.014 | | 0.031 | |
| rs9843653 | T | C | | | -0.008 | | | | 0.010 | | -0.008 | | | | 0.012 | | | | 0.000 | | | | 0.015 | | -0.014 | | 0.015 | | -0.033 | | 0.015 | |
| rs62259692 | G | A | | | 0.027 | | | | 0.025 | | 0.021 | | | | 0.029 | | | | -0.004 | | | | 0.037 | | 0.060 | | 0.038 | | 0.033 | | 0.038 | |
| rs6798941 | C | T | | | -0.015 | | | | 0.010 | | -0.018 | | | | 0.013 | | | | -0.019 | | | | 0.016 | | -0.017 | | 0.016 | | -0.008 | | 0.017 | |
| rs6445198 | G | T | | | 0.015 | | | | 0.010 | | 0.005 | | | | 0.012 | | | | -0.005 | | | | 0.015 | | 0.013 | | 0.015 | | 0.024 | | 0.015 | |
| rs6445258 | T | C | | | 0.002 | | | | 0.013 | | 0.010 | | | | 0.015 | | | | 0.004 | | | | 0.019 | | 0.019 | | 0.020 | | -0.017 | | 0.020 | |
| rs76824303 | A | C | | | 0.008 | | | | 0.016 | | 0.016 | | | | 0.019 | | | | 0.021 | | | | 0.025 | | 0.005 | | 0.025 | | 0.025 | | 0.026 | |
| rs557951 | T | G | | | -0.015 | | | | 0.010 | | -0.020 | | | | 0.012 | | | | -0.009 | | | | 0.016 | | -0.030 | | 0.016 | | -0.017 | | 0.016 | |
| rs11708540 | G | A | | | -0.027 | | | | 0.014 | | -0.023 | | | | 0.016 | | | | -0.033 | | | | 0.021 | | -0.010 | | 0.021 | | -0.032 | | 0.021 | |
| rs1598121 | A | G | | | -0.005 | | | | 0.010 | | 0.001 | | | | 0.012 | | | | 0.005 | | | | 0.015 | | 0.000 | | 0.016 | | -0.003 | | 0.016 | |
| rs114593013 | A | G | | | -0.003 | | | | 0.021 | | -0.026 | | | | 0.025 | | | | -0.030 | | | | 0.032 | | -0.009 | | 0.033 | | -0.001 | | 0.033 | |
| rs11915747 | C | G | | | -0.015 | | | | 0.010 | | -0.026 | | | | 0.012 | | | | -0.021 | | | | 0.015 | | -0.035 | | 0.016 | | -0.021 | | 0.016 | |
| rs4858940 | T | C | | | -0.006 | | | | 0.017 | | 0.000 | | | | 0.020 | | | | 0.014 | | | | 0.025 | | -0.010 | | 0.026 | | -0.036 | | 0.026 | |
| rs1454687 | C | G | | | -0.012 | | | | 0.010 | | -0.012 | | | | 0.012 | | | | -0.011 | | | | 0.015 | | -0.014 | | 0.015 | | -0.005 | | 0.015 | |
| rs1436348 | A | G | | | -0.011 | | | | 0.010 | | -0.016 | | | | 0.012 | | | | -0.011 | | | | 0.015 | | -0.023 | | 0.015 | | 0.004 | | 0.015 | |
| rs36131051 | T | G | | | 0.013 | | | | 0.013 | | 0.013 | | | | 0.015 | | | | 0.013 | | | | 0.019 | | 0.022 | | 0.020 | | 0.023 | | 0.020 | |
| rs9814758 | T | G | | | 0.001 | | | | 0.010 | | -0.002 | | | | 0.012 | | | | -0.005 | | | | 0.015 | | 0.005 | | 0.016 | | 0.011 | | 0.016 | |
| rs1320903 | G | A | | | -0.002 | | | | 0.010 | | 0.009 | | | | 0.012 | | | | -0.006 | | | | 0.016 | | 0.021 | | 0.016 | | -0.033 | | 0.016 | |
| rs10935143 | G | A | | | 0.003 | | | | 0.010 | | -0.009 | | | | 0.012 | | | | -0.007 | | | | 0.015 | | -0.014 | | 0.015 | | -0.002 | | 0.015 | |
| rs2343681 | G | A | | | -0.018 | | | | 0.012 | | -0.021 | | | | 0.014 | | | | -0.014 | | | | 0.018 | | -0.018 | | 0.018 | | -0.016 | | 0.018 | |
| rs2035936 | G | T | | | -0.001 | | | | 0.020 | | -0.016 | | | | 0.026 | | | | -0.055 | | | | 0.033 | | 0.042 | | 0.035 | | -0.004 | | 0.034 | |
| rs12634936 | T | C | | | 0.012 | | | | 0.026 | | 0.037 | | | | 0.031 | | | | 0.001 | | | | 0.039 | | 0.097 | | 0.042 | | -0.018 | | 0.041 | |
| rs1568488 | G | C | | | 0.012 | | | | 0.010 | | 0.017 | | | | 0.012 | | | | 0.035 | | | | 0.015 | | 0.003 | | 0.016 | | 0.012 | | 0.016 | |
| rs9834519 | C | T | | | 0.000 | | | | 0.019 | | -0.002 | | | | 0.023 | | | | 0.012 | | | | 0.029 | | -0.011 | | 0.029 | | 0.001 | | 0.029 | |
| rs12630209 | T | G | | | -0.002 | | | | 0.011 | | -0.014 | | | | 0.013 | | | | -0.010 | | | | 0.017 | | -0.021 | | 0.017 | | 0.008 | | 0.018 | |
| rs8192675 | T | C | | | 0.012 | | | | 0.010 | | 0.012 | | | | 0.013 | | | | -0.005 | | | | 0.016 | | 0.025 | | 0.016 | | 0.013 | | 0.016 | |
| rs529200 | A | G | | | -0.003 | | | | 0.010 | | -0.004 | | | | 0.012 | | | | -0.007 | | | | 0.015 | | -0.001 | | 0.015 | | -0.017 | | 0.015 | |
| rs13061117 | T | C | | | 0.012 | | | | 0.016 | | 0.025 | | | | 0.019 | | | | 0.020 | | | | 0.025 | | 0.032 | | 0.025 | | 0.003 | | 0.025 | |
| rs262956 | T | G | | | -0.006 | | | | 0.010 | | -0.008 | | | | 0.012 | | | | -0.009 | | | | 0.015 | | -0.001 | | 0.016 | | -0.028 | | 0.016 | |
| rs869400 | T | G | | | 0.021 | | | | 0.013 | | 0.020 | | | | 0.015 | | | | 0.025 | | | | 0.019 | | 0.013 | | 0.020 | | 0.002 | | 0.020 | |
| rs80236973 | C | T | | | 0.013 | | | | 0.015 | | 0.035 | | | | 0.018 | | | | 0.028 | | | | 0.022 | | 0.050 | | 0.023 | | 0.017 | | 0.023 | |
| rs4677813 | T | C | | | 0.027 | | | | 0.011 | | 0.020 | | | | 0.013 | | | | 0.021 | | | | 0.017 | | 0.012 | | 0.018 | | 0.025 | | 0.018 | |
| rs6583310 | G | C | | | -0.001 | | | | 0.010 | | -0.008 | | | | 0.012 | | | | -0.013 | | | | 0.015 | | -0.001 | | 0.016 | | 0.008 | | 0.016 | |
| rs2051559 | T | C | | | -0.017 | | | | 0.014 | | -0.010 | | | | 0.017 | | | | 0.003 | | | | 0.022 | | -0.034 | | 0.022 | | -0.059 | | 0.022 | |
| rs35852935 | A | C | | | 0.027 | | | | 0.027 | | 0.066 | | | | 0.032 | | | | 0.045 | | | | 0.041 | | 0.082 | | 0.042 | | 0.032 | | 0.041 | |
| rs1477890 | A | G | | | -0.017 | | | | 0.010 | | -0.013 | | | | 0.012 | | | | -0.010 | | | | 0.015 | | -0.017 | | 0.015 | | -0.039 | | 0.015 | |
| rs34811474 | G | A | | | 0.022 | | | | 0.013 | | 0.038 | | | | 0.016 | | | | 0.033 | | | | 0.020 | | 0.043 | | 0.021 | | 0.016 | | 0.021 | |
| rs73213484 | A | T | | | 0.008 | | | | 0.013 | | 0.014 | | | | 0.016 | | | | 0.010 | | | | 0.020 | | 0.020 | | 0.021 | | 0.036 | | 0.021 | |
| rs4527444 | A | G | | | -0.002 | | | | 0.010 | | -0.005 | | | | 0.012 | | | | 0.013 | | | | 0.015 | | -0.024 | | 0.015 | | 0.001 | | 0.015 | |
| rs36023504 | C | T | | | 0.005 | | | | 0.010 | | 0.009 | | | | 0.012 | | | | 0.021 | | | | 0.016 | | -0.003 | | 0.016 | | 0.010 | | 0.016 | |
| rs12507026 | A | T | | | 0.000 | | | | 0.010 | | -0.005 | | | | 0.012 | | | | -0.007 | | | | 0.015 | | 0.002 | | 0.015 | | 0.000 | | 0.015 | |
| rs2237025 | T | C | | | -0.043 | | | | 0.010 | | -0.024 | | | | 0.012 | | | | -0.024 | | | | 0.015 | | -0.029 | | 0.015 | | -0.067 | | 0.015 | |
| rs28462076 | A | G | | | 0.008 | | | | 0.011 | | -0.001 | | | | 0.014 | | | | -0.005 | | | | 0.017 | | -0.005 | | 0.018 | | -0.005 | | 0.018 | |
| rs2164300 | C | T | | | -0.005 | | | | 0.010 | | -0.005 | | | | 0.012 | | | | 0.000 | | | | 0.015 | | -0.012 | | 0.015 | | -0.005 | | 0.015 | |
| rs13104584 | G | A | | | -0.012 | | | | 0.010 | | -0.011 | | | | 0.012 | | | | -0.011 | | | | 0.015 | | -0.011 | | 0.015 | | -0.005 | | 0.015 | |
| rs4148155 | A | G | | | 0.004 | | | | 0.015 | | 0.014 | | | | 0.019 | | | | 0.029 | | | | 0.024 | | 0.003 | | 0.025 | | 0.018 | | 0.025 | |
| rs4419475 | A | T | | | -0.001 | | | | 0.010 | | -0.007 | | | | 0.012 | | | | -0.025 | | | | 0.015 | | 0.019 | | 0.016 | | -0.012 | | 0.016 | |
| rs1229984 | T | C | | | -0.039 | | | | 0.021 | | -0.039 | | | | 0.026 | | | | -0.061 | | | | 0.034 | | -0.015 | | 0.034 | | -0.063 | | 0.035 | |
| rs2583410 | A | C | | | -0.008 | | | | 0.013 | | -0.022 | | | | 0.016 | | | | -0.050 | | | | 0.020 | | 0.007 | | 0.021 | | -0.004 | | 0.021 | |
| rs13107325 | C | T | | | 0.008 | | | | 0.019 | | 0.012 | | | | 0.022 | | | | -0.027 | | | | 0.028 | | 0.044 | | 0.029 | | -0.006 | | 0.029 | |
| rs1381010 | G | A | | | -0.006 | | | | 0.010 | | -0.004 | | | | 0.013 | | | | -0.012 | | | | 0.016 | | 0.001 | | 0.016 | | 0.002 | | 0.016 | |
| rs2952863 | T | G | | | -0.005 | | | | 0.010 | | -0.003 | | | | 0.013 | | | | -0.005 | | | | 0.016 | | 0.002 | | 0.016 | | -0.003 | | 0.016 | |
| rs1296328 | A | C | | | -0.012 | | | | 0.010 | | -0.001 | | | | 0.012 | | | | -0.009 | | | | 0.015 | | 0.008 | | 0.015 | | -0.025 | | 0.015 | |
| rs809955 | G | A | | | 0.010 | | | | 0.010 | | 0.006 | | | | 0.012 | | | | -0.015 | | | | 0.015 | | 0.027 | | 0.016 | | 0.013 | | 0.016 | |
| rs35390852 | G | A | | | -0.009 | | | | 0.015 | | -0.007 | | | | 0.018 | | | | -0.005 | | | | 0.023 | | -0.006 | | 0.024 | | -0.040 | | 0.024 | |
| rs12644329 | G | A | | | -0.014 | | | | 0.010 | | -0.016 | | | | 0.012 | | | | -0.009 | | | | 0.015 | | -0.031 | | 0.015 | | -0.002 | | 0.016 | |
| rs6823268 | A | G | | | 0.020 | | | | 0.010 | | 0.014 | | | | 0.012 | | | | 0.016 | | | | 0.015 | | 0.013 | | 0.015 | | 0.034 | | 0.016 | |
| rs113079574 | C | T | | | 0.005 | | | | 0.012 | | 0.016 | | | | 0.014 | | | | 0.027 | | | | 0.018 | | 0.000 | | 0.019 | | -0.004 | | 0.019 | |
| rs6843852 | C | T | | | -0.020 | | | | 0.010 | | -0.028 | | | | 0.012 | | | | -0.018 | | | | 0.015 | | -0.032 | | 0.015 | | -0.001 | | 0.015 | |
| rs698147 | A | G | | | -0.003 | | | | 0.010 | | -0.011 | | | | 0.012 | | | | -0.009 | | | | 0.015 | | -0.010 | | 0.015 | | 0.001 | | 0.015 | |
| rs67913249 | C | G | | | 0.020 | | | | 0.010 | | 0.022 | | | | 0.012 | | | | 0.022 | | | | 0.015 | | 0.016 | | 0.016 | | 0.038 | | 0.016 | |
| rs114263339 | C | T | | | 0.028 | | | | 0.032 | | 0.022 | | | | 0.038 | | | | 0.006 | | | | 0.048 | | 0.063 | | 0.050 | | -0.035 | | 0.049 | |
| rs10805383 | G | A | | | 0.025 | | | | 0.010 | | 0.022 | | | | 0.012 | | | | 0.020 | | | | 0.015 | | 0.027 | | 0.015 | | 0.018 | | 0.015 | |
| rs9291822 | C | T | | | 0.006 | | | | 0.010 | | 0.013 | | | | 0.012 | | | | 0.008 | | | | 0.015 | | 0.018 | | 0.015 | | -0.009 | | 0.015 | |
| rs27215 | C | A | | | -0.006 | | | | 0.011 | | -0.002 | | | | 0.014 | | | | -0.005 | | | | 0.017 | | 0.008 | | 0.018 | | -0.008 | | 0.018 | |
| rs2307111 | T | C | | | 0.016 | | | | 0.010 | | 0.008 | | | | 0.012 | | | | -0.003 | | | | 0.015 | | 0.018 | | 0.015 | | 0.009 | | 0.015 | |
| rs252749 | G | A | | | -0.008 | | | | 0.011 | | 0.003 | | | | 0.014 | | | | 0.015 | | | | 0.017 | | -0.020 | | 0.018 | | -0.022 | | 0.018 | |
| rs59893724 | A | G | | | 0.004 | | | | 0.012 | | 0.005 | | | | 0.014 | | | | 0.015 | | | | 0.017 | | -0.013 | | 0.018 | | 0.003 | | 0.018 | |
| rs79236537 | G | T | | | 0.012 | | | | 0.032 | | 0.019 | | | | 0.038 | | | | -0.005 | | | | 0.048 | | 0.051 | | 0.051 | | 0.104 | | 0.051 | |
| rs6870983 | C | T | | | -0.031 | | | | 0.012 | | -0.036 | | | | 0.014 | | | | -0.032 | | | | 0.017 | | -0.043 | | 0.018 | | -0.011 | | 0.018 | |
| rs1477290 | T | C | | | 0.004 | | | | 0.013 | | -0.009 | | | | 0.016 | | | | -0.002 | | | | 0.020 | | -0.020 | | 0.021 | | 0.020 | | 0.021 | |
| rs142503704 | G | A | | | 0.041 | | | | 0.042 | | 0.044 | | | | 0.049 | | | | 0.049 | | | | 0.063 | | 0.029 | | 0.065 | | 0.011 | | 0.066 | |
| rs62379271 | T | G | | | -0.008 | | | | 0.010 | | -0.008 | | | | 0.012 | | | | -0.009 | | | | 0.015 | | -0.003 | | 0.015 | | -0.009 | | 0.015 | |
| rs149457 | C | T | | | 0.008 | | | | 0.013 | | 0.008 | | | | 0.015 | | | | 0.020 | | | | 0.019 | | -0.003 | | 0.020 | | 0.014 | | 0.020 | |
| rs12517187 | C | T | | | -0.004 | | | | 0.010 | | -0.010 | | | | 0.012 | | | | -0.019 | | | | 0.015 | | -0.008 | | 0.015 | | 0.008 | | 0.015 | |
| rs347551 | C | G | | | 0.000 | | | | 0.010 | | -0.002 | | | | 0.012 | | | | 0.001 | | | | 0.015 | | -0.006 | | 0.016 | | 0.003 | | 0.016 | |
| rs1582931 | G | A | | | -0.008 | | | | 0.010 | | -0.016 | | | | 0.012 | | | | -0.006 | | | | 0.015 | | -0.024 | | 0.015 | | -0.017 | | 0.015 | |
| rs4836133 | C | A | | | -0.004 | | | | 0.010 | | -0.014 | | | | 0.012 | | | | -0.022 | | | | 0.015 | | -0.006 | | 0.015 | | 0.002 | | 0.015 | |
| rs329118 | C | T | | | 0.005 | | | | 0.010 | | 0.008 | | | | 0.012 | | | | 0.018 | | | | 0.015 | | 0.000 | | 0.015 | | 0.025 | | 0.015 | |
| rs13174863 | A | G | | | -0.029 | | | | 0.013 | | -0.032 | | | | 0.016 | | | | -0.027 | | | | 0.020 | | -0.034 | | 0.021 | | -0.035 | | 0.021 | |
| rs7719067 | A | G | | | 0.012 | | | | 0.010 | | 0.018 | | | | 0.012 | | | | 0.014 | | | | 0.015 | | 0.009 | | 0.015 | | -0.011 | | 0.015 | |
| rs11134512 | T | G | | | 0.009 | | | | 0.010 | | 0.006 | | | | 0.012 | | | | 0.015 | | | | 0.016 | | -0.009 | | 0.016 | | 0.009 | | 0.016 | |
| rs11134679 | A | G | | | 0.005 | | | | 0.010 | | 0.009 | | | | 0.012 | | | | 0.009 | | | | 0.016 | | 0.009 | | 0.016 | | -0.009 | | 0.016 | |
| rs4467770 | G | A | | | 0.018 | | | | 0.011 | | 0.014 | | | | 0.013 | | | | 0.009 | | | | 0.016 | | 0.025 | | 0.017 | | 0.032 | | 0.017 | |
| rs9395520 | C | T | | | 0.006 | | | | 0.010 | | 0.005 | | | | 0.013 | | | | 0.006 | | | | 0.016 | | -0.001 | | 0.016 | | 0.016 | | 0.016 | |
| rs3806114 | G | A | | | -0.008 | | | | 0.010 | | -0.004 | | | | 0.013 | | | | 0.030 | | | | 0.016 | | -0.038 | | 0.016 | | -0.010 | | 0.016 | |
| rs75499503 | C | T | | | 0.017 | | | | 0.012 | | 0.026 | | | | 0.014 | | | | 0.035 | | | | 0.017 | | 0.023 | | 0.018 | | 0.035 | | 0.018 | |
| rs34388845 | A | G | | | -0.029 | | | | 0.012 | | -0.022 | | | | 0.015 | | | | -0.035 | | | | 0.018 | | -0.015 | | 0.019 | | -0.039 | | 0.019 | |
| rs2260051 | A | T | | | -0.030 | | | | 0.010 | | -0.034 | | | | 0.012 | | | | -0.036 | | | | 0.015 | | -0.034 | | 0.016 | | -0.043 | | 0.016 | |
| rs9277992 | G | A | | | 0.003 | | | | 0.012 | | -0.011 | | | | 0.015 | | | | -0.014 | | | | 0.019 | | -0.005 | | 0.019 | | 0.009 | | 0.019 | |
| rs9366863 | T | C | | | -0.007 | | | | 0.010 | | -0.002 | | | | 0.012 | | | | -0.034 | | | | 0.015 | | 0.028 | | 0.016 | | -0.039 | | 0.016 | |
| rs34298980 | T | C | | | -0.005 | | | | 0.010 | | -0.014 | | | | 0.012 | | | | -0.020 | | | | 0.016 | | -0.003 | | 0.016 | | 0.003 | | 0.016 | |
| rs9462670 | G | C | | | -0.005 | | | | 0.011 | | 0.001 | | | | 0.014 | | | | 0.007 | | | | 0.017 | | -0.010 | | 0.018 | | -0.015 | | 0.018 | |
| rs72892910 | G | T | | | 0.011 | | | | 0.013 | | 0.008 | | | | 0.015 | | | | 0.019 | | | | 0.019 | | 0.001 | | 0.020 | | 0.026 | | 0.020 | |
| rs1327259 | A | G | | | 0.012 | | | | 0.010 | | 0.013 | | | | 0.012 | | | | 0.015 | | | | 0.015 | | 0.008 | | 0.015 | | 0.004 | | 0.016 | |
| rs1547026 | T | C | | | 0.003 | | | | 0.011 | | 0.016 | | | | 0.013 | | | | 0.000 | | | | 0.016 | | 0.032 | | 0.017 | | -0.004 | | 0.017 | |
| rs72910629 | A | G | | | -0.014 | | | | 0.014 | | -0.032 | | | | 0.017 | | | | -0.025 | | | | 0.022 | | -0.032 | | 0.022 | | 0.014 | | 0.023 | |
| rs1040046 | C | A | | | 0.004 | | | | 0.014 | | 0.001 | | | | 0.017 | | | | 0.001 | | | | 0.021 | | 0.004 | | 0.022 | | 0.011 | | 0.022 | |
| rs1324110 | G | C | | | 0.005 | | | | 0.010 | | 0.009 | | | | 0.012 | | | | 0.018 | | | | 0.015 | | 0.001 | | 0.015 | | -0.011 | | 0.015 | |
| rs10499014 | C | G | | | -0.013 | | | | 0.011 | | -0.011 | | | | 0.013 | | | | -0.017 | | | | 0.017 | | -0.006 | | 0.017 | | -0.007 | | 0.017 | |
| rs6938973 | T | C | | | -0.029 | | | | 0.010 | | -0.023 | | | | 0.012 | | | | -0.002 | | | | 0.015 | | -0.046 | | 0.015 | | -0.035 | | 0.015 | |
| rs9496567 | G | A | | | 0.003 | | | | 0.012 | | -0.007 | | | | 0.014 | | | | -0.012 | | | | 0.017 | | 0.008 | | 0.018 | | 0.010 | | 0.018 | |
| rs156126 | T | C | | | -0.001 | | | | 0.012 | | -0.007 | | | | 0.015 | | | | -0.012 | | | | 0.018 | | -0.007 | | 0.019 | | 0.000 | | 0.019 | |
| rs2253310 | C | G | | | -0.014 | | | | 0.010 | | -0.017 | | | | 0.012 | | | | -0.034 | | | | 0.015 | | -0.008 | | 0.015 | | -0.007 | | 0.015 | |
| rs13218383 | C | G | | | -0.001 | | | | 0.010 | | -0.006 | | | | 0.012 | | | | -0.001 | | | | 0.015 | | -0.012 | | 0.016 | | -0.005 | | 0.016 | |
| rs2875762 | G | C | | | 0.003 | | | | 0.011 | | -0.002 | | | | 0.014 | | | | -0.013 | | | | 0.017 | | 0.006 | | 0.018 | | 0.001 | | 0.018 | |
| rs10457469 | G | A | | | -0.004 | | | | 0.010 | | 0.000 | | | | 0.012 | | | | 0.001 | | | | 0.015 | | 0.001 | | 0.015 | | -0.013 | | 0.015 | |
| rs12213441 | C | T | | | -0.003 | | | | 0.012 | | -0.010 | | | | 0.015 | | | | 0.006 | | | | 0.018 | | -0.018 | | 0.019 | | 0.009 | | 0.019 | |
| rs7749708 | C | T | | | -0.011 | | | | 0.010 | | 0.001 | | | | 0.013 | | | | 0.019 | | | | 0.016 | | -0.019 | | 0.016 | | -0.017 | | 0.016 | |
| rs9478496 | T | C | | | -0.018 | | | | 0.013 | | -0.012 | | | | 0.016 | | | | -0.017 | | | | 0.020 | | -0.003 | | 0.020 | | -0.007 | | 0.020 | |
| rs9480184 | C | T | | | 0.014 | | | | 0.012 | | 0.018 | | | | 0.014 | | | | 0.024 | | | | 0.018 | | 0.011 | | 0.018 | | 0.005 | | 0.018 | |
| rs36007635 | G | A | | | 0.029 | | | | 0.014 | | 0.042 | | | | 0.017 | | | | 0.061 | | | | 0.022 | | 0.025 | | 0.022 | | 0.016 | | 0.022 | |
| rs6950388 | G | A | | | -0.010 | | | | 0.012 | | -0.012 | | | | 0.015 | | | | -0.015 | | | | 0.018 | | -0.012 | | 0.019 | | -0.026 | | 0.019 | |
| rs2056477 | G | C | | | 0.010 | | | | 0.011 | | 0.009 | | | | 0.013 | | | | 0.009 | | | | 0.017 | | 0.010 | | 0.017 | | 0.021 | | 0.017 | |
| rs4307239 | A | G | | | -0.008 | | | | 0.010 | | -0.017 | | | | 0.012 | | | | -0.019 | | | | 0.015 | | -0.022 | | 0.015 | | 0.016 | | 0.015 | |
| rs215634 | A | G | | | -0.003 | | | | 0.010 | | -0.010 | | | | 0.012 | | | | -0.007 | | | | 0.015 | | -0.012 | | 0.015 | | -0.010 | | 0.015 | |
| rs2237402 | G | A | | | 0.013 | | | | 0.010 | | 0.022 | | | | 0.012 | | | | 0.036 | | | | 0.015 | | 0.006 | | 0.016 | | 0.004 | | 0.016 | |
| rs2289379 | C | T | | | 0.015 | | | | 0.010 | | 0.009 | | | | 0.012 | | | | 0.002 | | | | 0.015 | | 0.020 | | 0.015 | | 0.000 | | 0.015 | |
| rs3823674 | C | T | | | 0.000 | | | | 0.010 | | -0.017 | | | | 0.012 | | | | -0.014 | | | | 0.015 | | -0.020 | | 0.015 | | 0.035 | | 0.015 | |
| rs11765062 | T | C | | | -0.022 | | | | 0.010 | | -0.023 | | | | 0.012 | | | | -0.025 | | | | 0.015 | | -0.018 | | 0.015 | | 0.004 | | 0.015 | |
| rs2866720 | C | T | | | 0.008 | | | | 0.010 | | 0.005 | | | | 0.012 | | | | 0.019 | | | | 0.015 | | -0.015 | | 0.016 | | -0.009 | | 0.016 | |
| rs1852006 | G | A | | | -0.011 | | | | 0.010 | | -0.007 | | | | 0.012 | | | | -0.014 | | | | 0.015 | | 0.001 | | 0.016 | | -0.009 | | 0.016 | |
| rs6963840 | C | T | | | -0.004 | | | | 0.014 | | -0.007 | | | | 0.016 | | | | -0.004 | | | | 0.020 | | -0.018 | | 0.021 | | -0.017 | | 0.021 | |
| rs12538826 | T | C | | | 0.013 | | | | 0.015 | | 0.004 | | | | 0.018 | | | | -0.005 | | | | 0.023 | | 0.011 | | 0.023 | | 0.026 | | 0.024 | |
| rs2074686 | G | A | | | -0.016 | | | | 0.010 | | -0.021 | | | | 0.012 | | | | -0.017 | | | | 0.015 | | -0.026 | | 0.015 | | 0.016 | | 0.015 | |
| rs11496125 | C | T | | | 0.004 | | | | 0.010 | | 0.004 | | | | 0.012 | | | | 0.003 | | | | 0.015 | | 0.003 | | 0.015 | | -0.025 | | 0.015 | |
| rs2396625 | T | A | | | 0.010 | | | | 0.010 | | 0.011 | | | | 0.012 | | | | 0.017 | | | | 0.015 | | 0.003 | | 0.015 | | -0.018 | | 0.015 | |
| rs12705894 | G | A | | | -0.007 | | | | 0.010 | | -0.003 | | | | 0.012 | | | | -0.003 | | | | 0.015 | | 0.002 | | 0.016 | | -0.013 | | 0.016 | |
| rs1840660 | G | A | | | 0.003 | | | | 0.010 | | 0.011 | | | | 0.012 | | | | 0.006 | | | | 0.015 | | 0.017 | | 0.016 | | -0.013 | | 0.016 | |
| rs1899689 | C | T | | | -0.004 | | | | 0.010 | | 0.005 | | | | 0.012 | | | | -0.011 | | | | 0.015 | | 0.016 | | 0.015 | | -0.006 | | 0.015 | |
| rs35775580 | A | G | | | 0.029 | | | | 0.026 | | 0.033 | | | | 0.031 | | | | 0.049 | | | | 0.039 | | 0.024 | | 0.040 | | 0.040 | | 0.040 | |
| rs11976084 | C | T | | | -0.012 | | | | 0.011 | | -0.013 | | | | 0.013 | | | | -0.006 | | | | 0.017 | | -0.016 | | 0.017 | | -0.026 | | 0.018 | |
| rs11525873 | T | C | | | 0.017 | | | | 0.016 | | 0.015 | | | | 0.020 | | | | 0.008 | | | | 0.026 | | 0.028 | | 0.027 | | 0.043 | | 0.027 | |
| rs1805123 | T | G | | | 0.000 | | | | 0.012 | | 0.006 | | | | 0.014 | | | | 0.034 | | | | 0.017 | | -0.032 | | 0.018 | | -0.012 | | 0.018 | |
| rs7827182 | G | C | | | -0.009 | | | | 0.011 | | 0.005 | | | | 0.013 | | | | 0.025 | | | | 0.016 | | -0.018 | | 0.017 | | -0.026 | | 0.017 | |
| rs6601451 | C | G | | | 0.009 | | | | 0.011 | | -0.006 | | | | 0.013 | | | | -0.028 | | | | 0.016 | | 0.018 | | 0.017 | | 0.033 | | 0.017 | |
| rs55896564 | G | A | | | 0.010 | | | | 0.011 | | -0.010 | | | | 0.013 | | | | -0.018 | | | | 0.016 | | 0.003 | | 0.017 | | 0.029 | | 0.017 | |
| rs6530737 | A | G | | | -0.009 | | | | 0.010 | | -0.018 | | | | 0.012 | | | | -0.030 | | | | 0.015 | | -0.018 | | 0.016 | | 0.004 | | 0.016 | |
| rs2616143 | G | A | | | 0.016 | | | | 0.011 | | 0.017 | | | | 0.013 | | | | 0.013 | | | | 0.016 | | 0.010 | | 0.016 | | 0.016 | | 0.016 | |
| rs117176448 | C | G | | | 0.007 | | | | 0.017 | | 0.019 | | | | 0.020 | | | | 0.011 | | | | 0.025 | | 0.022 | | 0.026 | | 0.001 | | 0.026 | |
| rs4266606 | C | T | | | -0.001 | | | | 0.014 | | -0.005 | | | | 0.017 | | | | 0.011 | | | | 0.021 | | -0.024 | | 0.022 | | -0.005 | | 0.022 | |
| rs2725371 | A | G | | | 0.011 | | | | 0.011 | | 0.018 | | | | 0.013 | | | | 0.012 | | | | 0.016 | | 0.030 | | 0.016 | | -0.005 | | 0.017 | |
| rs4739558 | A | G | | | 0.007 | | | | 0.010 | | 0.011 | | | | 0.012 | | | | 0.004 | | | | 0.015 | | 0.014 | | 0.015 | | 0.008 | | 0.016 | |
| rs35894137 | C | T | | | 0.009 | | | | 0.019 | | 0.016 | | | | 0.022 | | | | -0.002 | | | | 0.028 | | 0.020 | | 0.029 | | 0.011 | | 0.029 | |
| rs143662847 | C | T | | | -0.025 | | | | 0.032 | | -0.032 | | | | 0.038 | | | | -0.081 | | | | 0.047 | | 0.025 | | 0.051 | | 0.002 | | 0.053 | |
| rs473837 | G | T | | | -0.012 | | | | 0.010 | | -0.016 | | | | 0.012 | | | | -0.013 | | | | 0.015 | | -0.015 | | 0.016 | | -0.005 | | 0.016 | |
| rs12681792 | C | A | | | -0.019 | | | | 0.012 | | -0.010 | | | | 0.015 | | | | -0.021 | | | | 0.019 | | 0.002 | | 0.020 | | -0.015 | | 0.020 | |
| rs4737188 | A | T | | | 0.010 | | | | 0.010 | | 0.020 | | | | 0.012 | | | | 0.031 | | | | 0.015 | | 0.008 | | 0.015 | | 0.020 | | 0.015 | |
| rs35957544 | G | T | | | -0.009 | | | | 0.010 | | -0.014 | | | | 0.012 | | | | -0.008 | | | | 0.015 | | -0.025 | | 0.015 | | -0.008 | | 0.015 | |
| rs2941432 | T | A | | | -0.018 | | | | 0.010 | | -0.010 | | | | 0.012 | | | | -0.016 | | | | 0.015 | | -0.011 | | 0.015 | | -0.035 | | 0.016 | |
| rs78565420 | C | T | | | -0.016 | | | | 0.030 | | -0.063 | | | | 0.035 | | | | -0.099 | | | | 0.044 | | -0.022 | | 0.047 | | -0.015 | | 0.049 | |
| rs17619860 | T | C | | | 0.009 | | | | 0.013 | | 0.005 | | | | 0.016 | | | | -0.002 | | | | 0.020 | | 0.011 | | 0.021 | | 0.029 | | 0.021 | |
| rs1905616 | G | A | | | 0.001 | | | | 0.010 | | 0.001 | | | | 0.012 | | | | -0.007 | | | | 0.016 | | 0.010 | | 0.016 | | 0.008 | | 0.016 | |
| rs2114210 | G | A | | | -0.010 | | | | 0.010 | | -0.009 | | | | 0.012 | | | | 0.022 | | | | 0.016 | | -0.036 | | 0.016 | | -0.005 | | 0.016 | |
| rs17716502 | C | T | | | 0.009 | | | | 0.013 | | 0.015 | | | | 0.015 | | | | 0.018 | | | | 0.019 | | 0.014 | | 0.019 | | 0.009 | | 0.020 | |
| rs72673947 | A | G | | | -0.006 | | | | 0.015 | | 0.002 | | | | 0.018 | | | | -0.007 | | | | 0.023 | | 0.003 | | 0.023 | | -0.006 | | 0.024 | |
| rs112875651 | G | A | | | 0.022 | | | | 0.010 | | 0.033 | | | | 0.012 | | | | 0.020 | | | | 0.015 | | 0.054 | | 0.016 | | -0.007 | | 0.016 | |
| rs11782074 | G | T | | | -0.007 | | | | 0.010 | | 0.001 | | | | 0.012 | | | | 0.007 | | | | 0.015 | | -0.009 | | 0.016 | | 0.000 | | 0.016 | |
| rs1865341 | C | T | | | -0.012 | | | | 0.011 | | -0.010 | | | | 0.014 | | | | -0.019 | | | | 0.017 | | -0.007 | | 0.018 | | -0.014 | | 0.018 | |
| rs10960276 | C | A | | | -0.018 | | | | 0.010 | | -0.019 | | | | 0.012 | | | | -0.019 | | | | 0.015 | | -0.019 | | 0.016 | | -0.005 | | 0.016 | |
| rs7020196 | C | T | | | 0.000 | | | | 0.010 | | 0.010 | | | | 0.012 | | | | 0.010 | | | | 0.015 | | 0.018 | | 0.016 | | -0.032 | | 0.016 | |
| rs13292699 | A | C | | | 0.021 | | | | 0.010 | | 0.027 | | | | 0.012 | | | | 0.044 | | | | 0.015 | | 0.001 | | 0.015 | | 0.008 | | 0.015 | |
| rs1411432 | A | C | | | -0.020 | | | | 0.012 | | -0.013 | | | | 0.015 | | | | -0.039 | | | | 0.018 | | 0.023 | | 0.019 | | 0.008 | | 0.019 | |
| rs17770336 | C | T | | | -0.027 | | | | 0.010 | | -0.022 | | | | 0.013 | | | | -0.036 | | | | 0.016 | | -0.023 | | 0.016 | | -0.018 | | 0.016 | |
| rs10969334 | C | A | | | 0.013 | | | | 0.010 | | 0.003 | | | | 0.012 | | | | -0.015 | | | | 0.015 | | 0.020 | | 0.015 | | 0.021 | | 0.015 | |
| rs10973159 | G | T | | | -0.004 | | | | 0.010 | | 0.004 | | | | 0.012 | | | | 0.014 | | | | 0.015 | | -0.006 | | 0.016 | | -0.017 | | 0.016 | |
| rs7038966 | C | T | | | -0.001 | | | | 0.010 | | 0.005 | | | | 0.012 | | | | -0.008 | | | | 0.015 | | 0.009 | | 0.015 | | -0.024 | | 0.015 | |
| rs1547205 | G | C | | | 0.025 | | | | 0.017 | | 0.014 | | | | 0.020 | | | | 0.036 | | | | 0.026 | | 0.001 | | 0.026 | | 0.045 | | 0.027 | |
| rs4989244 | G | A | | | -0.002 | | | | 0.010 | | 0.000 | | | | 0.012 | | | | -0.003 | | | | 0.015 | | -0.002 | | 0.015 | | 0.005 | | 0.015 | |
| rs2135745 | C | G | | | 0.012 | | | | 0.011 | | 0.006 | | | | 0.013 | | | | 0.007 | | | | 0.017 | | 0.013 | | 0.017 | | 0.025 | | 0.017 | |
| rs2417998 | C | G | | | -0.015 | | | | 0.011 | | -0.022 | | | | 0.013 | | | | -0.028 | | | | 0.016 | | -0.021 | | 0.017 | | -0.020 | | 0.017 | |
| rs12376870 | G | A | | | 0.018 | | | | 0.011 | | 0.021 | | | | 0.014 | | | | 0.013 | | | | 0.017 | | 0.030 | | 0.018 | | 0.013 | | 0.018 | |
| rs7038943 | T | C | | | 0.020 | | | | 0.010 | | 0.032 | | | | 0.012 | | | | 0.030 | | | | 0.015 | | 0.037 | | 0.015 | | 0.025 | | 0.016 | |
| rs6478538 | A | G | | | -0.027 | | | | 0.010 | | -0.024 | | | | 0.012 | | | | -0.015 | | | | 0.016 | | -0.035 | | 0.016 | | -0.042 | | 0.016 | |
| rs10760277 | C | T | | | 0.001 | | | | 0.010 | | -0.008 | | | | 0.012 | | | | -0.025 | | | | 0.015 | | 0.003 | | 0.016 | | 0.011 | | 0.016 | |
| rs7030609 | A | G | | | -0.024 | | | | 0.017 | | -0.018 | | | | 0.021 | | | | 0.016 | | | | 0.027 | | -0.051 | | 0.027 | | -0.028 | | 0.027 | |
| rs113132247 | G | A | | | -0.002 | | | | 0.013 | | 0.021 | | | | 0.016 | | | | 0.024 | | | | 0.020 | | 0.015 | | 0.021 | | -0.012 | | 0.021 | |
| rs7913496 | C | T | | | 0.001 | | | | 0.013 | | -0.004 | | | | 0.015 | | | | -0.008 | | | | 0.019 | | -0.007 | | 0.020 | | 0.006 | | 0.020 | |
| rs7893571 | G | T | | | 0.001 | | | | 0.010 | | 0.005 | | | | 0.012 | | | | -0.009 | | | | 0.016 | | 0.020 | | 0.016 | | -0.005 | | 0.016 | |
| rs12253527 | G | A | | | -0.028 | | | | 0.010 | | -0.024 | | | | 0.012 | | | | -0.024 | | | | 0.016 | | -0.021 | | 0.016 | | -0.051 | | 0.016 | |
| rs71495049 | G | A | | | -0.012 | | | | 0.017 | | -0.023 | | | | 0.021 | | | | -0.021 | | | | 0.026 | | -0.029 | | 0.027 | | -0.038 | | 0.027 | |
| rs3125326 | A | C | | | -0.005 | | | | 0.010 | | -0.012 | | | | 0.012 | | | | -0.028 | | | | 0.015 | | 0.001 | | 0.016 | | -0.012 | | 0.016 | |
| rs7924036 | G | T | | | -0.008 | | | | 0.010 | | -0.012 | | | | 0.012 | | | | -0.016 | | | | 0.015 | | -0.002 | | 0.015 | | 0.002 | | 0.015 | |
| rs11000993 | T | C | | | 0.011 | | | | 0.015 | | 0.025 | | | | 0.018 | | | | 0.004 | | | | 0.023 | | 0.039 | | 0.023 | | -0.033 | | 0.023 | |
| rs1250597 | A | G | | | -0.025 | | | | 0.010 | | -0.029 | | | | 0.012 | | | | -0.029 | | | | 0.015 | | -0.030 | | 0.015 | | -0.022 | | 0.015 | |
| rs17399739 | A | G | | | 0.022 | | | | 0.019 | | 0.034 | | | | 0.023 | | | | 0.048 | | | | 0.029 | | 0.033 | | 0.030 | | 0.031 | | 0.030 | |
| rs2450444 | G | A | | | -0.019 | | | | 0.010 | | -0.019 | | | | 0.012 | | | | -0.024 | | | | 0.016 | | -0.013 | | 0.016 | | -0.007 | | 0.016 | |
| rs41310284 | C | A | | | -0.011 | | | | 0.017 | | -0.006 | | | | 0.020 | | | | -0.013 | | | | 0.025 | | 0.006 | | 0.026 | | -0.010 | | 0.026 | |
| rs10736156 | C | A | | | -0.006 | | | | 0.013 | | -0.007 | | | | 0.016 | | | | -0.012 | | | | 0.020 | | 0.002 | | 0.020 | | -0.005 | | 0.020 | |
| rs7086898 | A | G | | | -0.022 | | | | 0.016 | | -0.032 | | | | 0.020 | | | | -0.042 | | | | 0.025 | | -0.016 | | 0.026 | | 0.002 | | 0.026 | |
| rs4575195 | C | A | | | -0.002 | | | | 0.011 | | 0.000 | | | | 0.012 | | | | 0.011 | | | | 0.016 | | -0.014 | | 0.016 | | 0.000 | | 0.016 | |
| rs9421249 | C | T | | | 0.025 | | | | 0.011 | | 0.010 | | | | 0.013 | | | | 0.000 | | | | 0.017 | | 0.022 | | 0.017 | | 0.020 | | 0.017 | |
| rs845084 | G | A | | | 0.011 | | | | 0.011 | | 0.009 | | | | 0.013 | | | | 0.012 | | | | 0.017 | | 0.007 | | 0.017 | | 0.011 | | 0.017 | |
| rs4962725 | T | C | | | -0.005 | | | | 0.010 | | -0.008 | | | | 0.012 | | | | -0.011 | | | | 0.015 | | -0.007 | | 0.015 | | -0.006 | | 0.015 | |
| rs2542615 | C | T | | | -0.008 | | | | 0.010 | | -0.001 | | | | 0.012 | | | | 0.010 | | | | 0.015 | | -0.009 | | 0.016 | | -0.018 | | 0.016 | |
| rs2035806 | G | A | | | 0.009 | | | | 0.010 | | 0.004 | | | | 0.012 | | | | 0.006 | | | | 0.015 | | 0.005 | | 0.015 | | 0.013 | | 0.015 | |
| rs67257872 | A | G | | | 0.002 | | | | 0.010 | | 0.010 | | | | 0.012 | | | | 0.009 | | | | 0.015 | | 0.013 | | 0.015 | | -0.001 | | 0.015 | |
| rs28711392 | T | C | | | -0.012 | | | | 0.010 | | -0.005 | | | | 0.012 | | | | -0.003 | | | | 0.015 | | -0.002 | | 0.015 | | -0.029 | | 0.015 | |
| rs6265 | C | T | | | 0.008 | | | | 0.012 | | 0.015 | | | | 0.015 | | | | 0.020 | | | | 0.018 | | 0.010 | | 0.019 | | 0.010 | | 0.019 | |
| rs10835498 | G | A | | | -0.001 | | | | 0.010 | | 0.001 | | | | 0.012 | | | | 0.014 | | | | 0.015 | | -0.019 | | 0.015 | | -0.006 | | 0.015 | |
| rs1222216 | C | T | | | 0.002 | | | | 0.012 | | -0.004 | | | | 0.014 | | | | -0.019 | | | | 0.018 | | 0.004 | | 0.019 | | -0.002 | | 0.019 | |
| rs59227842 | A | G | | | -0.013 | | | | 0.011 | | -0.017 | | | | 0.013 | | | | -0.007 | | | | 0.016 | | -0.024 | | 0.016 | | 0.011 | | 0.017 | |
| rs868784 | G | A | | | -0.012 | | | | 0.010 | | -0.012 | | | | 0.012 | | | | -0.023 | | | | 0.015 | | -0.003 | | 0.016 | | -0.022 | | 0.016 | |
| rs6416134 | G | C | | | 0.002 | | | | 0.012 | | 0.014 | | | | 0.015 | | | | 0.025 | | | | 0.018 | | 0.007 | | 0.019 | | 0.000 | | 0.019 | |
| rs12798028 | C | T | | | -0.008 | | | | 0.010 | | -0.004 | | | | 0.012 | | | | -0.013 | | | | 0.015 | | 0.000 | | 0.015 | | -0.006 | | 0.015 | |
| rs12363672 | A | C | | | -0.072 | | | | 0.055 | | -0.058 | | | | 0.064 | | | | -0.081 | | | | 0.081 | | -0.094 | | 0.088 | | -0.183 | | 0.092 | |
| rs34292685 | C | T | | | 0.023 | | | | 0.013 | | 0.023 | | | | 0.016 | | | | 0.005 | | | | 0.020 | | 0.033 | | 0.021 | | 0.007 | | 0.021 | |
| rs2234458 | C | T | | | 0.026 | | | | 0.010 | | 0.033 | | | | 0.012 | | | | 0.025 | | | | 0.015 | | 0.046 | | 0.016 | | 0.021 | | 0.016 | |
| rs667515 | G | C | | | 0.033 | | | | 0.010 | | 0.043 | | | | 0.012 | | | | 0.043 | | | | 0.015 | | 0.037 | | 0.016 | | 0.019 | | 0.016 | |
| rs10160769 | G | C | | | 0.001 | | | | 0.011 | | 0.004 | | | | 0.014 | | | | 0.012 | | | | 0.017 | | -0.011 | | 0.018 | | 0.013 | | 0.018 | |
| rs7102934 | T | C | | | -0.002 | | | | 0.010 | | 0.004 | | | | 0.012 | | | | -0.010 | | | | 0.016 | | 0.025 | | 0.016 | | 0.002 | | 0.016 | |
| rs61903695 | A | G | | | 0.002 | | | | 0.011 | | 0.008 | | | | 0.013 | | | | -0.007 | | | | 0.017 | | 0.027 | | 0.017 | | 0.014 | | 0.018 | |
| rs2658797 | C | T | | | 0.015 | | | | 0.010 | | 0.018 | | | | 0.012 | | | | 0.029 | | | | 0.015 | | 0.003 | | 0.015 | | -0.014 | | 0.015 | |
| rs680071 | T | C | | | -0.017 | | | | 0.015 | | -0.009 | | | | 0.018 | | | | -0.011 | | | | 0.023 | | -0.012 | | 0.023 | | -0.006 | | 0.023 | |
| rs719802 | T | C | | | 0.002 | | | | 0.010 | | 0.006 | | | | 0.012 | | | | -0.015 | | | | 0.015 | | 0.033 | | 0.016 | | 0.003 | | 0.016 | |
| rs11607476 | A | C | | | 0.010 | | | | 0.010 | | 0.015 | | | | 0.012 | | | | 0.018 | | | | 0.015 | | 0.006 | | 0.015 | | 0.000 | | 0.015 | |
| rs7928320 | C | G | | | 0.002 | | | | 0.020 | | 0.002 | | | | 0.024 | | | | 0.020 | | | | 0.031 | | -0.023 | | 0.031 | | 0.030 | | 0.032 | |
| rs12281009 | A | G | | | -0.002 | | | | 0.020 | | -0.004 | | | | 0.024 | | | | 0.014 | | | | 0.030 | | -0.022 | | 0.031 | | 0.021 | | 0.032 | |
| rs7925100 | G | A | | | -0.018 | | | | 0.010 | | -0.013 | | | | 0.012 | | | | -0.001 | | | | 0.015 | | -0.023 | | 0.015 | | -0.028 | | 0.016 | |
| rs11218510 | G | A | | | -0.002 | | | | 0.010 | | -0.007 | | | | 0.012 | | | | -0.009 | | | | 0.015 | | -0.012 | | 0.015 | | 0.018 | | 0.015 | |
| rs10791113 | A | G | | | -0.015 | | | | 0.010 | | -0.010 | | | | 0.012 | | | | -0.006 | | | | 0.015 | | -0.013 | | 0.015 | | -0.021 | | 0.015 | |
| rs12788343 | T | C | | | 0.005 | | | | 0.010 | | 0.004 | | | | 0.012 | | | | 0.009 | | | | 0.015 | | -0.003 | | 0.015 | | 0.013 | | 0.016 | |
| rs11223204 | A | G | | | 0.009 | | | | 0.010 | | 0.014 | | | | 0.012 | | | | 0.016 | | | | 0.015 | | 0.012 | | 0.015 | | 0.012 | | 0.015 | |
| rs329651 | G | T | | | -0.006 | | | | 0.013 | | -0.001 | | | | 0.015 | | | | -0.013 | | | | 0.019 | | 0.011 | | 0.019 | | -0.005 | | 0.020 | |
| rs61909165 | T | A | | | -0.012 | | | | 0.013 | | 0.001 | | | | 0.016 | | | | -0.012 | | | | 0.020 | | 0.006 | | 0.021 | | -0.065 | | 0.021 | |
| rs55726687 | G | A | | | -0.015 | | | | 0.012 | | -0.026 | | | | 0.014 | | | | -0.033 | | | | 0.018 | | -0.016 | | 0.019 | | 0.007 | | 0.019 | |
| rs10774018 | G | C | | | -0.014 | | | | 0.011 | | -0.027 | | | | 0.014 | | | | -0.031 | | | | 0.017 | | -0.030 | | 0.018 | | -0.010 | | 0.018 | |
| rs1799507 | G | A | | | -0.013 | | | | 0.014 | | -0.005 | | | | 0.017 | | | | -0.007 | | | | 0.021 | | -0.002 | | 0.022 | | 0.010 | | 0.022 | |
| rs10505836 | A | C | | | -0.023 | | | | 0.013 | | -0.002 | | | | 0.016 | | | | 0.001 | | | | 0.020 | | -0.001 | | 0.021 | | -0.042 | | 0.021 | |
| rs10842231 | A | T | | | -0.007 | | | | 0.016 | | -0.014 | | | | 0.020 | | | | -0.003 | | | | 0.025 | | -0.021 | | 0.026 | | 0.019 | | 0.026 | |
| rs1458156 | C | T | | | 0.008 | | | | 0.010 | | 0.020 | | | | 0.012 | | | | 0.026 | | | | 0.015 | | 0.020 | | 0.015 | | 0.008 | | 0.015 | |
| rs1126930 | G | C | | | -0.004 | | | | 0.029 | | -0.020 | | | | 0.035 | | | | -0.007 | | | | 0.044 | | -0.050 | | 0.046 | | -0.034 | | 0.046 | |
| rs7132908 | G | A | | | -0.012 | | | | 0.010 | | -0.006 | | | | 0.012 | | | | 0.004 | | | | 0.015 | | -0.014 | | 0.015 | | -0.006 | | 0.016 | |
| rs4077093 | T | G | | | 0.008 | | | | 0.012 | | -0.002 | | | | 0.014 | | | | 0.014 | | | | 0.018 | | -0.021 | | 0.019 | | 0.020 | | 0.018 | |
| rs4759073 | G | A | | | -0.012 | | | | 0.010 | | -0.011 | | | | 0.012 | | | | -0.008 | | | | 0.015 | | -0.006 | | 0.016 | | -0.019 | | 0.016 | |
| rs4759228 | G | C | | | 0.003 | | | | 0.011 | | 0.017 | | | | 0.014 | | | | 0.011 | | | | 0.017 | | 0.038 | | 0.018 | | 0.009 | | 0.018 | |
| rs12821416 | C | T | | | -0.008 | | | | 0.015 | | -0.012 | | | | 0.017 | | | | -0.011 | | | | 0.022 | | -0.018 | | 0.023 | | -0.016 | | 0.023 | |
| rs61754230 | C | T | | | 0.014 | | | | 0.049 | | -0.006 | | | | 0.058 | | | | -0.021 | | | | 0.073 | | 0.016 | | 0.077 | | 0.056 | | 0.077 | |
| rs12427047 | C | T | | | -0.011 | | | | 0.011 | | -0.023 | | | | 0.014 | | | | -0.040 | | | | 0.017 | | -0.014 | | 0.018 | | 0.019 | | 0.018 | |
| rs2712667 | G | C | | | -0.009 | | | | 0.010 | | -0.005 | | | | 0.012 | | | | -0.001 | | | | 0.015 | | -0.008 | | 0.015 | | -0.028 | | 0.016 | |
| rs4764949 | A | G | | | 0.000 | | | | 0.010 | | 0.008 | | | | 0.012 | | | | -0.001 | | | | 0.015 | | 0.016 | | 0.016 | | -0.017 | | 0.016 | |
| rs6606686 | G | C | | | 0.009 | | | | 0.011 | | 0.020 | | | | 0.013 | | | | 0.010 | | | | 0.016 | | 0.028 | | 0.016 | | -0.004 | | 0.017 | |
| rs11513729 | C | T | | | 0.065 | | | | 0.010 | | 0.074 | | | | 0.012 | | | | 0.080 | | | | 0.015 | | 0.069 | | 0.016 | | 0.065 | | 0.016 | |
| rs181617194 | T | C | | | 0.020 | | | | 0.032 | | 0.025 | | | | 0.038 | | | | 0.072 | | | | 0.048 | | -0.001 | | 0.049 | | -0.004 | | 0.050 | |
| rs147730268 | G | T | | | 0.020 | | | | 0.020 | | 0.028 | | | | 0.024 | | | | 0.061 | | | | 0.030 | | -0.005 | | 0.031 | | 0.025 | | 0.031 | |
| rs9579775 | A | C | | | -0.034 | | | | 0.016 | | -0.043 | | | | 0.020 | | | | -0.039 | | | | 0.025 | | -0.044 | | 0.025 | | -0.021 | | 0.026 | |
| rs9507791 | G | A | | | -0.010 | | | | 0.012 | | -0.007 | | | | 0.015 | | | | -0.006 | | | | 0.018 | | 0.004 | | 0.019 | | -0.023 | | 0.019 | |
| rs1967772 | G | A | | | 0.006 | | | | 0.011 | | 0.011 | | | | 0.013 | | | | 0.011 | | | | 0.016 | | 0.010 | | 0.017 | | -0.009 | | 0.017 | |
| rs35193668 | C | T | | | -0.003 | | | | 0.010 | | 0.006 | | | | 0.012 | | | | -0.007 | | | | 0.015 | | 0.019 | | 0.016 | | -0.021 | | 0.016 | |
| rs61954177 | G | C | | | 0.009 | | | | 0.010 | | 0.016 | | | | 0.012 | | | | 0.010 | | | | 0.016 | | 0.019 | | 0.016 | | 0.013 | | 0.016 | |
| rs12429545 | G | A | | | 0.005 | | | | 0.014 | | 0.015 | | | | 0.017 | | | | 0.016 | | | | 0.022 | | 0.006 | | 0.022 | | 0.020 | | 0.023 | |
| rs7321285 | A | C | | | 0.016 | | | | 0.012 | | 0.016 | | | | 0.014 | | | | 0.008 | | | | 0.018 | | 0.024 | | 0.019 | | 0.014 | | 0.019 | |
| rs2576135 | T | A | | | 0.022 | | | | 0.017 | | 0.010 | | | | 0.020 | | | | 0.012 | | | | 0.025 | | 0.002 | | 0.026 | | 0.027 | | 0.026 | |
| rs9317002 | C | A | | | -0.009 | | | | 0.010 | | -0.009 | | | | 0.012 | | | | -0.003 | | | | 0.015 | | -0.022 | | 0.015 | | -0.011 | | 0.015 | |
| rs9529148 | G | A | | | 0.001 | | | | 0.010 | | 0.007 | | | | 0.012 | | | | -0.011 | | | | 0.015 | | 0.032 | | 0.015 | | 0.013 | | 0.016 | |
| rs1576655 | A | C | | | -0.017 | | | | 0.010 | | -0.021 | | | | 0.012 | | | | -0.020 | | | | 0.015 | | -0.024 | | 0.016 | | -0.009 | | 0.016 | |
| rs61971082 | T | G | | | -0.014 | | | | 0.011 | | -0.014 | | | | 0.013 | | | | -0.012 | | | | 0.017 | | -0.017 | | 0.017 | | -0.027 | | 0.017 | |
| rs7331420 | G | A | | | -0.011 | | | | 0.011 | | -0.021 | | | | 0.013 | | | | -0.003 | | | | 0.016 | | -0.038 | | 0.017 | | -0.002 | | 0.017 | |
| rs9888533 | C | T | | | 0.018 | | | | 0.010 | | 0.010 | | | | 0.012 | | | | 0.000 | | | | 0.015 | | 0.013 | | 0.016 | | 0.029 | | 0.016 | |
| rs9522180 | C | T | | | 0.015 | | | | 0.010 | | 0.013 | | | | 0.012 | | | | 0.002 | | | | 0.015 | | 0.013 | | 0.015 | | -0.010 | | 0.015 | |
| rs9515446 | A | G | | | 0.000 | | | | 0.010 | | 0.002 | | | | 0.012 | | | | 0.009 | | | | 0.015 | | -0.007 | | 0.015 | | -0.013 | | 0.015 | |
| rs8015400 | C | A | | | 0.009 | | | | 0.012 | | 0.010 | | | | 0.012 | | | | 0.014 | | | | 0.015 | | 0.003 | | 0.016 | | 0.014 | | 0.016 | |
| rs9788550 | G | C | | | -0.006 | | | | 0.013 | | 0.018 | | | | 0.014 | | | | -0.001 | | | | 0.017 | | 0.033 | | 0.018 | | -0.007 | | 0.018 | |
| rs12883788 | C | T | | | -0.007 | | | | 0.011 | | -0.007 | | | | 0.012 | | | | -0.008 | | | | 0.015 | | -0.004 | | 0.015 | | -0.010 | | 0.015 | |
| rs7141912 | A | T | | | -0.014 | | | | 0.017 | | 0.010 | | | | 0.017 | | | | 0.010 | | | | 0.022 | | 0.008 | | 0.023 | | -0.004 | | 0.023 | |
| rs8011566 | T | A | | | 0.034 | | | | 0.012 | | 0.007 | | | | 0.012 | | | | 0.009 | | | | 0.015 | | 0.005 | | 0.015 | | 0.029 | | 0.015 | |
| rs724623 | A | C | | | -0.001 | | | | 0.011 | | -0.003 | | | | 0.012 | | | | -0.014 | | | | 0.015 | | 0.010 | | 0.015 | | -0.025 | | 0.015 | |
| rs217672 | A | C | | | -0.001 | | | | 0.013 | | -0.009 | | | | 0.013 | | | | 0.006 | | | | 0.017 | | -0.020 | | 0.017 | | 0.002 | | 0.017 | |
| rs3902951 | T | G | | | 0.010 | | | | 0.013 | | 0.019 | | | | 0.014 | | | | 0.020 | | | | 0.017 | | 0.021 | | 0.018 | | 0.013 | | 0.018 | |
| rs61986330 | C | A | | | 0.009 | | | | 0.013 | | 0.028 | | | | 0.013 | | | | 0.021 | | | | 0.017 | | 0.036 | | 0.017 | | -0.013 | | 0.017 | |
| rs10146997 | A | G | | | -0.012 | | | | 0.015 | | -0.003 | | | | 0.014 | | | | 0.003 | | | | 0.018 | | -0.003 | | 0.019 | | 0.003 | | 0.019 | |
| rs8008772 | A | T | | | 0.013 | | | | 0.014 | | 0.005 | | | | 0.014 | | | | -0.013 | | | | 0.017 | | 0.025 | | 0.018 | | -0.005 | | 0.018 | |
| rs1286138 | T | G | | | -0.008 | | | | 0.012 | | -0.007 | | | | 0.012 | | | | -0.020 | | | | 0.016 | | 0.006 | | 0.016 | | 0.001 | | 0.016 | |
| rs6575340 | G | A | | | -0.004 | | | | 0.012 | | 0.012 | | | | 0.012 | | | | 0.004 | | | | 0.015 | | 0.024 | | 0.016 | | -0.004 | | 0.016 | |
| rs12885251 | G | A | | | 0.016 | | | | 0.012 | | 0.027 | | | | 0.012 | | | | 0.036 | | | | 0.015 | | 0.016 | | 0.015 | | 0.022 | | 0.015 | |
| rs12147845 | C | T | | | -0.035 | | | | 0.019 | | 0.001 | | | | 0.019 | | | | 0.014 | | | | 0.023 | | -0.012 | | 0.024 | | -0.020 | | 0.024 | |
| rs61992671 | A | G | | | 0.012 | | | | 0.012 | | 0.021 | | | | 0.013 | | | | 0.025 | | | | 0.016 | | 0.014 | | 0.016 | | 0.011 | | 0.016 | |
| rs7145882 | T | C | | | -0.009 | | | | 0.012 | | -0.004 | | | | 0.012 | | | | -0.006 | | | | 0.015 | | -0.002 | | 0.016 | | -0.010 | | 0.016 | |
| rs3759584 | T | C | | | 0.011 | | | | 0.012 | | 0.005 | | | | 0.012 | | | | -0.005 | | | | 0.015 | | 0.011 | | 0.016 | | 0.020 | | 0.016 | |
| rs76520838 | C | T | | | -0.040 | | | | 0.027 | | -0.073 | | | | 0.032 | | | | -0.099 | | | | 0.040 | | -0.054 | | 0.041 | | -0.011 | | 0.042 | |
| rs7182917 | T | C | | | 0.001 | | | | 0.010 | | -0.010 | | | | 0.012 | | | | 0.000 | | | | 0.015 | | -0.028 | | 0.015 | | 0.025 | | 0.015 | |
| rs2247401 | G | A | | | -0.015 | | | | 0.011 | | -0.022 | | | | 0.013 | | | | -0.030 | | | | 0.017 | | -0.010 | | 0.017 | | 0.010 | | 0.017 | |
| rs28465175 | A | G | | | 0.017 | | | | 0.019 | | 0.027 | | | | 0.022 | | | | 0.034 | | | | 0.028 | | 0.026 | | 0.029 | | 0.028 | | 0.029 | |
| rs7175642 | T | G | | | -0.013 | | | | 0.011 | | -0.019 | | | | 0.013 | | | | -0.022 | | | | 0.016 | | -0.014 | | 0.017 | | -0.004 | | 0.017 | |
| rs28408562 | C | G | | | -0.024 | | | | 0.010 | | -0.024 | | | | 0.012 | | | | -0.022 | | | | 0.015 | | -0.028 | | 0.015 | | -0.017 | | 0.015 | |
| rs1369159 | C | T | | | 0.002 | | | | 0.010 | | 0.007 | | | | 0.012 | | | | 0.017 | | | | 0.015 | | -0.001 | | 0.016 | | 0.020 | | 0.016 | |
| rs111584879 | T | C | | | -0.011 | | | | 0.011 | | -0.008 | | | | 0.014 | | | | 0.000 | | | | 0.017 | | -0.023 | | 0.018 | | -0.046 | | 0.018 | |
| rs2241420 | G | A | | | 0.044 | | | | 0.011 | | 0.043 | | | | 0.014 | | | | 0.057 | | | | 0.017 | | 0.028 | | 0.018 | | 0.012 | | 0.018 | |
| rs62004865 | T | A | | | -0.021 | | | | 0.017 | | -0.023 | | | | 0.020 | | | | -0.034 | | | | 0.025 | | -0.010 | | 0.026 | | -0.033 | | 0.026 | |
| rs11856579 | G | A | | | -0.010 | | | | 0.011 | | -0.019 | | | | 0.013 | | | | -0.021 | | | | 0.017 | | -0.024 | | 0.017 | | -0.011 | | 0.017 | |
| rs57488047 | T | C | | | 0.014 | | | | 0.010 | | 0.015 | | | | 0.012 | | | | 0.003 | | | | 0.015 | | 0.027 | | 0.015 | | 0.011 | | 0.015 | |
| rs34994596 | T | C | | | -0.030 | | | | 0.011 | | -0.017 | | | | 0.013 | | | | -0.018 | | | | 0.016 | | -0.010 | | 0.017 | | -0.046 | | 0.017 | |
| rs7498044 | G | A | | | 0.007 | | | | 0.012 | | 0.006 | | | | 0.014 | | | | 0.005 | | | | 0.018 | | 0.005 | | 0.019 | | 0.008 | | 0.019 | |
| rs8038574 | T | C | | | 0.002 | | | | 0.010 | | 0.002 | | | | 0.012 | | | | 0.001 | | | | 0.015 | | -0.001 | | 0.016 | | -0.005 | | 0.016 | |
| rs56803094 | A | G | | | -0.002 | | | | 0.012 | | -0.004 | | | | 0.014 | | | | -0.007 | | | | 0.018 | | -0.004 | | 0.018 | | -0.009 | | 0.018 | |
| rs412243 | T | C | | | 0.007 | | | | 0.010 | | 0.002 | | | | 0.012 | | | | 0.011 | | | | 0.015 | | -0.005 | | 0.016 | | 0.017 | | 0.016 | |
| rs2516726 | T | C | | | 0.013 | | | | 0.012 | | 0.014 | | | | 0.014 | | | | -0.003 | | | | 0.018 | | 0.028 | | 0.019 | | 0.001 | | 0.018 | |
| rs879620 | C | T | | | -0.010 | | | | 0.010 | | -0.004 | | | | 0.012 | | | | -0.004 | | | | 0.015 | | -0.012 | | 0.015 | | -0.012 | | 0.015 | |
| rs2660241 | T | C | | | 0.021 | | | | 0.010 | | 0.012 | | | | 0.012 | | | | -0.001 | | | | 0.015 | | 0.021 | | 0.016 | | 0.026 | | 0.016 | |
| rs11642387 | A | G | | | -0.029 | | | | 0.017 | | -0.030 | | | | 0.020 | | | | -0.054 | | | | 0.025 | | -0.004 | | 0.027 | | -0.036 | | 0.026 | |
| rs39674 | C | G | | | -0.002 | | | | 0.010 | | -0.013 | | | | 0.012 | | | | 0.004 | | | | 0.016 | | -0.035 | | 0.016 | | 0.000 | | 0.016 | |
| rs12927792 | C | T | | | -0.001 | | | | 0.010 | | -0.011 | | | | 0.012 | | | | -0.015 | | | | 0.015 | | -0.006 | | 0.015 | | 0.016 | | 0.015 | |
| rs8054082 | C | T | | | -0.010 | | | | 0.014 | | -0.018 | | | | 0.017 | | | | 0.004 | | | | 0.022 | | -0.041 | | 0.022 | | 0.017 | | 0.022 | |
| rs4432271 | C | T | | | -0.008 | | | | 0.014 | | -0.004 | | | | 0.017 | | | | -0.006 | | | | 0.022 | | 0.014 | | 0.022 | | -0.001 | | 0.022 | |
| rs11864909 | C | T | | | 0.002 | | | | 0.011 | | -0.009 | | | | 0.013 | | | | -0.003 | | | | 0.016 | | -0.012 | | 0.017 | | 0.012 | | 0.017 | |
| rs9922288 | A | G | | | -0.011 | | | | 0.012 | | -0.020 | | | | 0.015 | | | | -0.016 | | | | 0.018 | | -0.018 | | 0.019 | | 0.018 | | 0.019 | |
| rs7498665 | A | G | | | -0.011 | | | | 0.010 | | -0.016 | | | | 0.012 | | | | -0.020 | | | | 0.015 | | -0.017 | | 0.016 | | -0.007 | | 0.016 | |
| rs3814883 | C | T | | | -0.002 | | | | 0.010 | | -0.006 | | | | 0.012 | | | | 0.012 | | | | 0.015 | | -0.021 | | 0.015 | | 0.015 | | 0.015 | |
| rs34898535 | C | T | | | 0.021 | | | | 0.010 | | 0.019 | | | | 0.012 | | | | 0.033 | | | | 0.015 | | 0.009 | | 0.016 | | 0.017 | | 0.016 | |
| rs56094641 | A | G | | | 0.004 | | | | 0.010 | | -0.002 | | | | 0.012 | | | | 0.000 | | | | 0.015 | | -0.007 | | 0.015 | | 0.008 | | 0.015 | |
| rs862320 | C | T | | | 0.019 | | | | 0.010 | | 0.026 | | | | 0.012 | | | | 0.033 | | | | 0.015 | | 0.020 | | 0.015 | | -0.003 | | 0.015 | |
| rs12149660 | G | A | | | -0.030 | | | | 0.016 | | -0.030 | | | | 0.019 | | | | -0.010 | | | | 0.025 | | -0.045 | | 0.025 | | -0.021 | | 0.026 | |
| rs811054 | C | T | | | -0.010 | | | | 0.010 | | -0.014 | | | | 0.012 | | | | -0.030 | | | | 0.015 | | 0.001 | | 0.015 | | 0.003 | | 0.015 | |
| rs4500770 | A | T | | | 0.024 | | | | 0.010 | | 0.025 | | | | 0.012 | | | | 0.020 | | | | 0.016 | | 0.026 | | 0.016 | | 0.030 | | 0.016 | |
| rs9673839 | A | G | | | -0.004 | | | | 0.010 | | -0.006 | | | | 0.012 | | | | -0.010 | | | | 0.015 | | 0.002 | | 0.015 | | 0.007 | | 0.015 | |
| rs12926506 | C | T | | | -0.011 | | | | 0.013 | | -0.007 | | | | 0.016 | | | | -0.004 | | | | 0.021 | | -0.012 | | 0.021 | | -0.005 | | 0.022 | |
| rs11150462 | T | A | | | 0.008 | | | | 0.010 | | 0.014 | | | | 0.012 | | | | 0.016 | | | | 0.015 | | 0.019 | | 0.016 | | -0.007 | | 0.016 | |
| rs7206608 | C | G | | | -0.028 | | | | 0.011 | | -0.029 | | | | 0.013 | | | | -0.028 | | | | 0.016 | | -0.034 | | 0.016 | | -0.032 | | 0.016 | |
| rs4790292 | C | A | | | -0.007 | | | | 0.014 | | -0.007 | | | | 0.017 | | | | 0.000 | | | | 0.021 | | -0.023 | | 0.021 | | 0.014 | | 0.022 | |
| rs58351927 | A | G | | | 0.004 | | | | 0.010 | | 0.003 | | | | 0.013 | | | | -0.002 | | | | 0.016 | | 0.014 | | 0.016 | | -0.002 | | 0.016 | |
| rs4792716 | A | G | | | 0.007 | | | | 0.010 | | 0.006 | | | | 0.012 | | | | -0.001 | | | | 0.015 | | 0.013 | | 0.015 | | 0.005 | | 0.015 | |
| rs1320251 | C | T | | | 0.018 | | | | 0.010 | | 0.021 | | | | 0.012 | | | | 0.024 | | | | 0.015 | | 0.023 | | 0.015 | | -0.001 | | 0.015 | |
| rs1017529 | C | A | | | 0.009 | | | | 0.014 | | 0.007 | | | | 0.017 | | | | 0.002 | | | | 0.021 | | 0.005 | | 0.022 | | -0.004 | | 0.022 | |
| rs73982435 | C | T | | | -0.014 | | | | 0.011 | | -0.022 | | | | 0.014 | | | | -0.008 | | | | 0.017 | | -0.041 | | 0.018 | | 0.003 | | 0.018 | |
| rs113962925 | C | T | | | -0.005 | | | | 0.017 | | 0.002 | | | | 0.021 | | | | 0.011 | | | | 0.026 | | -0.004 | | 0.027 | | -0.003 | | 0.027 | |
| rs11079849 | C | T | | | -0.009 | | | | 0.010 | | -0.008 | | | | 0.012 | | | | -0.019 | | | | 0.015 | | 0.007 | | 0.016 | | -0.013 | | 0.016 | |
| rs78369934 | T | C | | | -0.010 | | | | 0.023 | | -0.008 | | | | 0.027 | | | | -0.023 | | | | 0.034 | | -0.006 | | 0.035 | | -0.061 | | 0.035 | |
| rs11150745 | A | G | | | -0.007 | | | | 0.010 | | -0.008 | | | | 0.012 | | | | 0.000 | | | | 0.016 | | -0.008 | | 0.016 | | 0.007 | | 0.016 | |
| rs2083323 | G | A | | | -0.007 | | | | 0.013 | | -0.003 | | | | 0.015 | | | | -0.030 | | | | 0.019 | | 0.033 | | 0.020 | | 0.000 | | 0.020 | |
| rs512121 | T | C | | | -0.006 | | | | 0.012 | | 0.006 | | | | 0.014 | | | | -0.001 | | | | 0.018 | | 0.019 | | 0.019 | | -0.008 | | 0.019 | |
| rs1788808 | A | G | | | 0.017 | | | | 0.010 | | 0.014 | | | | 0.012 | | | | 0.004 | | | | 0.015 | | 0.026 | | 0.015 | | 0.027 | | 0.015 | |
| rs16940823 | C | A | | | 0.006 | | | | 0.013 | | 0.018 | | | | 0.016 | | | | 0.028 | | | | 0.020 | | 0.012 | | 0.020 | | 0.009 | | 0.020 | |
| rs6507054 | T | C | | | 0.009 | | | | 0.010 | | 0.015 | | | | 0.012 | | | | 0.010 | | | | 0.015 | | 0.027 | | 0.015 | | -0.006 | | 0.015 | |
| rs559231 | G | T | | | 0.005 | | | | 0.010 | | 0.008 | | | | 0.012 | | | | 0.020 | | | | 0.015 | | -0.007 | | 0.016 | | -0.002 | | 0.016 | |
| rs1834144 | C | A | | | 0.020 | | | | 0.010 | | 0.032 | | | | 0.012 | | | | 0.025 | | | | 0.015 | | 0.045 | | 0.016 | | 0.015 | | 0.016 | |
| rs7230240 | C | T | | | -0.008 | | | | 0.011 | | -0.006 | | | | 0.013 | | | | -0.011 | | | | 0.016 | | 0.007 | | 0.017 | | 0.004 | | 0.017 | |
| rs58243949 | C | T | | | 0.003 | | | | 0.012 | | 0.003 | | | | 0.014 | | | | 0.004 | | | | 0.018 | | 0.016 | | 0.019 | | 0.016 | | 0.019 | |
| rs11659764 | T | A | | | 0.001 | | | | 0.022 | | 0.006 | | | | 0.026 | | | | 0.039 | | | | 0.033 | | -0.027 | | 0.033 | | 0.019 | | 0.034 | |
| rs1517037 | C | T | | | 0.005 | | | | 0.012 | | -0.001 | | | | 0.015 | | | | -0.008 | | | | 0.019 | | 0.009 | | 0.019 | | -0.004 | | 0.019 | |
| rs58084604 | C | T | | | -0.019 | | | | 0.011 | | -0.011 | | | | 0.014 | | | | -0.009 | | | | 0.017 | | -0.011 | | 0.018 | | -0.026 | | 0.018 | |
| rs17773370 | G | A | | | 0.010 | | | | 0.022 | | 0.006 | | | | 0.027 | | | | 0.010 | | | | 0.034 | | 0.004 | | 0.035 | | 0.019 | | 0.035 | |
| rs57636386 | T | C | | | 0.018 | | | | 0.017 | | 0.012 | | | | 0.021 | | | | 0.004 | | | | 0.026 | | 0.022 | | 0.027 | | 0.031 | | 0.027 | |
| rs12454712 | T | C | | | -0.006 | | | | 0.010 | | 0.000 | | | | 0.013 | | | | 0.003 | | | | 0.016 | | 0.002 | | 0.016 | | -0.017 | | 0.016 | |
| rs1373349 | C | T | | | -0.001 | | | | 0.010 | | -0.015 | | | | 0.012 | | | | -0.006 | | | | 0.015 | | -0.034 | | 0.016 | | 0.002 | | 0.016 | |
| rs8089514 | T | A | | | -0.024 | | | | 0.010 | | -0.024 | | | | 0.012 | | | | -0.028 | | | | 0.015 | | -0.021 | | 0.016 | | -0.023 | | 0.016 | |
| rs45521740 | G | A | | | -0.016 | | | | 0.025 | | -0.001 | | | | 0.030 | | | | -0.021 | | | | 0.037 | | 0.021 | | 0.039 | | 0.044 | | 0.040 | |
| rs72976986 | G | A | | | -0.033 | | | | 0.013 | | -0.022 | | | | 0.015 | | | | 0.009 | | | | 0.019 | | -0.056 | | 0.019 | | -0.048 | | 0.019 | |
| rs75957461 | C | T | | | -0.023 | | | | 0.021 | | -0.043 | | | | 0.024 | | | | -0.052 | | | | 0.031 | | -0.037 | | 0.032 | | 0.006 | | 0.032 | |
| rs6511826 | G | A | | | 0.006 | | | | 0.019 | | -0.010 | | | | 0.023 | | | | 0.012 | | | | 0.029 | | -0.032 | | 0.030 | | 0.021 | | 0.030 | |
| rs273505 | T | C | | | 0.002 | | | | 0.010 | | 0.001 | | | | 0.012 | | | | 0.001 | | | | 0.015 | | 0.000 | | 0.015 | | -0.007 | | 0.015 | |
| rs113230003 | G | A | | | 0.003 | | | | 0.011 | | -0.001 | | | | 0.013 | | | | 0.020 | | | | 0.017 | | -0.030 | | 0.017 | | 0.003 | | 0.018 | |
| rs10404726 | C | T | | | 0.023 | | | | 0.010 | | 0.027 | | | | 0.012 | | | | 0.039 | | | | 0.015 | | 0.016 | | 0.015 | | 0.039 | | 0.015 | |
| rs112253053 | T | A | | | -0.028 | | | | 0.013 | | -0.031 | | | | 0.016 | | | | -0.038 | | | | 0.020 | | -0.026 | | 0.020 | | -0.026 | | 0.020 | |
| rs12462975 | G | A | | | -0.025 | | | | 0.010 | | -0.028 | | | | 0.012 | | | | -0.013 | | | | 0.016 | | -0.035 | | 0.016 | | -0.028 | | 0.016 | |
| rs73026723 | C | T | | | 0.010 | | | | 0.014 | | 0.014 | | | | 0.017 | | | | 0.013 | | | | 0.021 | | 0.016 | | 0.022 | | 0.009 | | 0.022 | |
| rs7255223 | C | A | | | 0.009 | | | | 0.011 | | 0.004 | | | | 0.013 | | | | -0.005 | | | | 0.016 | | 0.016 | | 0.017 | | 0.001 | | 0.017 | |
| rs429358 | T | C | | | 0.005 | | | | 0.014 | | 0.017 | | | | 0.017 | | | | 0.029 | | | | 0.022 | | -0.001 | | 0.022 | | 0.007 | | 0.022 | |
| rs12971645 | G | A | | | 0.009 | | | | 0.011 | | 0.010 | | | | 0.013 | | | | 0.021 | | | | 0.017 | | -0.006 | | 0.017 | | 0.019 | | 0.017 | |
| rs10423928 | T | A | | | -0.005 | | | | 0.012 | | -0.002 | | | | 0.014 | | | | -0.002 | | | | 0.018 | | 0.001 | | 0.018 | | -0.007 | | 0.019 | |
| rs3810291 | G | A | | | 0.000 | | | | 0.010 | | 0.003 | | | | 0.013 | | | | -0.008 | | | | 0.016 | | 0.017 | | 0.016 | | -0.005 | | 0.016 | |
| rs4545921 | A | G | | | 0.003 | | | | 0.010 | | -0.003 | | | | 0.012 | | | | 0.002 | | | | 0.015 | | -0.006 | | 0.015 | | -0.019 | | 0.016 | |
| rs61746970 | G | A | | | 0.022 | | | | 0.029 | | 0.028 | | | | 0.034 | | | | 0.011 | | | | 0.043 | | 0.042 | | 0.045 | | 0.033 | | 0.045 | |
| rs8111074 | G | T | | | 0.003 | | | | 0.011 | | 0.005 | | | | 0.013 | | | | 0.009 | | | | 0.017 | | 0.007 | | 0.017 | | -0.004 | | 0.017 | |
| rs6075658 | T | C | | | -0.004 | | | | 0.010 | | 0.001 | | | | 0.011 | | | | 0.013 | | | | 0.015 | | -0.016 | | 0.015 | | -0.015 | | 0.015 | |
| rs2206925 | T | C | | | 0.049 | | | | 0.010 | | 0.031 | | | | 0.012 | | | | 0.024 | | | | 0.015 | | 0.037 | | 0.016 | | 0.061 | | 0.016 | |
| rs4813224 | T | C | | | 0.007 | | | | 0.011 | | 0.002 | | | | 0.013 | | | | 0.016 | | | | 0.017 | | -0.009 | | 0.017 | | 0.019 | | 0.017 | |
| rs8124896 | T | C | | | -0.011 | | | | 0.016 | | -0.020 | | | | 0.019 | | | | -0.030 | | | | 0.024 | | 0.000 | | 0.025 | | 0.030 | | 0.025 | |
| rs6050446 | A | G | | | -0.004 | | | | 0.032 | | -0.017 | | | | 0.038 | | | | -0.053 | | | | 0.048 | | 0.030 | | 0.049 | | 0.032 | | 0.049 | |
| rs201475383 | G | A | | | 0.008 | | | | 0.037 | | 0.024 | | | | 0.044 | | | | 0.054 | | | | 0.056 | | -0.014 | | 0.056 | | -0.028 | | 0.057 | |
| rs1987960 | T | C | | | -0.015 | | | | 0.032 | | -0.035 | | | | 0.037 | | | | -0.054 | | | | 0.047 | | -0.020 | | 0.049 | | 0.019 | | 0.050 | |
| rs4911382 | C | T | | | 0.011 | | | | 0.010 | | 0.000 | | | | 0.012 | | | | -0.012 | | | | 0.015 | | 0.005 | | 0.015 | | 0.028 | | 0.015 | |
| rs6029180 | A | G | | | -0.003 | | | | 0.010 | | 0.011 | | | | 0.013 | | | | 0.004 | | | | 0.016 | | 0.023 | | 0.017 | | 0.005 | | 0.017 | |
| rs6030803 | T | C | | | 0.018 | | | | 0.014 | | 0.022 | | | | 0.017 | | | | 0.026 | | | | 0.022 | | 0.027 | | 0.022 | | 0.028 | | 0.022 | |
| rs2425856 | A | G | | | 0.002 | | | | 0.010 | | -0.003 | | | | 0.012 | | | | 0.001 | | | | 0.015 | | -0.008 | | 0.015 | | 0.012 | | 0.015 | |
| rs112852122 | G | A | | | -0.010 | | | | 0.014 | | -0.014 | | | | 0.017 | | | | -0.016 | | | | 0.021 | | -0.013 | | 0.022 | | -0.003 | | 0.022 | |
| rs66460909 | G | A | | | 0.024 | | | | 0.013 | | 0.025 | | | | 0.015 | | | | 0.032 | | | | 0.019 | | 0.017 | | 0.019 | | 0.020 | | 0.020 | |
| rs559390 | A | T | | | -0.008 | | | | 0.010 | | -0.002 | | | | 0.012 | | | | 0.005 | | | | 0.015 | | -0.012 | | 0.016 | | -0.014 | | 0.016 | |
| rs8134638 | T | C | | | 0.000 | | | | 0.010 | | -0.002 | | | | 0.012 | | | | 0.000 | | | | 0.015 | | 0.009 | | 0.016 | | 0.018 | | 0.016 | |
| rs2837398 | A | C | | | 0.002 | | | | 0.010 | | 0.003 | | | | 0.012 | | | | -0.009 | | | | 0.015 | | 0.013 | | 0.015 | | -0.012 | | 0.015 | |
| rs1964926 | A | G | | | -0.005 | | | | 0.010 | | -0.003 | | | | 0.012 | | | | -0.003 | | | | 0.015 | | 0.005 | | 0.016 | | -0.014 | | 0.016 | |
| rs403694 | C | T | | | 0.003 | | | | 0.010 | | 0.003 | | | | 0.012 | | | | 0.004 | | | | 0.015 | | 0.004 | | 0.015 | | 0.012 | | 0.015 | |
| rs1296685 | A | G | | | 0.017 | | | | 0.012 | | 0.000 | | | | 0.015 | | | | -0.003 | | | | 0.019 | | 0.000 | | 0.019 | | 0.003 | | 0.019 | |
| rs12484438 | T | C | | | 0.015 | | | | 0.010 | | 0.008 | | | | 0.012 | | | | 0.004 | | | | 0.016 | | 0.013 | | 0.016 | | 0.018 | | 0.016 | |
| rs738140 | A | G | | | -0.022 | | | | 0.011 | | -0.032 | | | | 0.012 | | | | -0.032 | | | | 0.016 | | -0.027 | | 0.016 | | -0.008 | | 0.016 | |
| rs9615723 | C | T | | | -0.019 | | | | 0.010 | | -0.031 | | | | 0.012 | | | | -0.016 | | | | 0.015 | | -0.042 | | 0.016 | | -0.008 | | 0.016 | |
| rs34778589 | A | C | | | 0.012 | | | | 0.019 | | 0.001 | | | | 0.022 | | | | -0.018 | | | | 0.028 | | 0.023 | | 0.029 | | 0.055 | | 0.029 | |
| Adult body size SNPs (men only) | | | | | | | | | | | | | | | | | | | | | | | | | | | | | | | | |
| SNP | EA | | OA | | | | | beta_ca | se_ca | | | beta_co | | | se_co | | | | | beta_prox | | | se_prox | | beta_dist | | se_dist | | beta_re | | se_re | |
| rs3762444 | C | | T | | | | | 0.021 | 0.014 | | | 0.018 | | | 0.017 | | | | | 0.027 | | | 0.022 | | 0.022 | | 0.022 | | 0.031 | | 0.021 | |
| rs1284373 | C | | T | | | | | 0.008 | 0.016 | | | 0.000 | | | 0.020 | | | | | -0.014 | | | 0.026 | | 0.013 | | 0.025 | | -0.012 | | 0.024 | |
| rs34052145 | A | | G | | | | | -0.015 | 0.015 | | | -0.036 | | | 0.018 | | | | | -0.039 | | | 0.023 | | -0.034 | | 0.022 | | -0.012 | | 0.022 | |
| rs1167311 | G | | A | | | | | 0.025 | 0.015 | | | 0.035 | | | 0.019 | | | | | 0.034 | | | 0.024 | | 0.033 | | 0.023 | | 0.037 | | 0.023 | |
| rs12140153 | G | | T | | | | | 0.000 | 0.026 | | | 0.012 | | | 0.032 | | | | | -0.008 | | | 0.041 | | 0.027 | | 0.040 | | -0.034 | | 0.038 | |
| rs11208779 | G | | C | | | | | 0.012 | 0.014 | | | 0.006 | | | 0.017 | | | | | 0.015 | | | 0.022 | | 0.013 | | 0.021 | | -0.013 | | 0.020 | |
| rs61765650 | A | | G | | | | | 0.010 | 0.019 | | | 0.022 | | | 0.023 | | | | | 0.038 | | | 0.029 | | 0.018 | | 0.028 | | 0.010 | | 0.027 | |
| rs34517439 | C | | A | | | | | -0.008 | 0.024 | | | -0.004 | | | 0.029 | | | | | 0.014 | | | 0.037 | | -0.030 | | 0.036 | | -0.018 | | 0.035 | |
| rs2181375 | A | | G | | | | | 0.022 | 0.014 | | | 0.020 | | | 0.017 | | | | | 0.030 | | | 0.022 | | 0.010 | | 0.021 | | 0.030 | | 0.020 | |
| rs17024258 | C | | T | | | | | -0.002 | 0.039 | | | -0.029 | | | 0.048 | | | | | -0.003 | | | 0.062 | | -0.014 | | 0.060 | | -0.037 | | 0.058 | |
| rs984225 | G | | A | | | | | 0.001 | 0.014 | | | -0.004 | | | 0.017 | | | | | -0.002 | | | 0.022 | | -0.012 | | 0.021 | | -0.027 | | 0.021 | |
| rs61813324 | C | | T | | | | | -0.037 | 0.024 | | | -0.036 | | | 0.029 | | | | | -0.020 | | | 0.037 | | -0.066 | | 0.036 | | -0.026 | | 0.036 | |
| rs543874 | A | | G | | | | | 0.013 | 0.017 | | | -0.001 | | | 0.022 | | | | | -0.025 | | | 0.028 | | 0.008 | | 0.027 | | 0.031 | | 0.026 | |
| rs2125232 | C | | T | | | | | -0.008 | 0.014 | | | -0.008 | | | 0.018 | | | | | 0.013 | | | 0.023 | | -0.028 | | 0.022 | | 0.001 | | 0.022 | |
| rs1529897 | T | | G | | | | | 0.006 | 0.014 | | | -0.014 | | | 0.017 | | | | | -0.025 | | | 0.022 | | -0.010 | | 0.021 | | -0.002 | | 0.021 | |
| rs935166 | G | | A | | | | | 0.010 | 0.014 | | | 0.006 | | | 0.017 | | | | | 0.012 | | | 0.021 | | 0.009 | | 0.021 | | 0.026 | | 0.020 | |
| rs1861410 | C | | T | | | | | -0.009 | 0.014 | | | -0.013 | | | 0.017 | | | | | -0.017 | | | 0.021 | | -0.016 | | 0.021 | | 0.000 | | 0.020 | |
| rs3552 | G | | A | | | | | 0.015 | 0.014 | | | 0.023 | | | 0.017 | | | | | 0.008 | | | 0.022 | | 0.031 | | 0.021 | | 0.001 | | 0.020 | |
| rs396354 | T | | C | | | | | 0.001 | 0.015 | | | -0.012 | | | 0.019 | | | | | -0.003 | | | 0.024 | | -0.022 | | 0.024 | | 0.010 | | 0.023 | |
| rs6753397 | C | | T | | | | | 0.009 | 0.015 | | | 0.001 | | | 0.019 | | | | | 0.001 | | | 0.024 | | 0.010 | | 0.024 | | 0.029 | | 0.023 | |
| rs13405033 | C | | T | | | | | -0.046 | 0.020 | | | -0.036 | | | 0.024 | | | | | -0.006 | | | 0.031 | | -0.055 | | 0.030 | | -0.037 | | 0.029 | |
| rs1451077 | G | | A | | | | | -0.003 | 0.014 | | | 0.004 | | | 0.017 | | | | | -0.014 | | | 0.022 | | 0.021 | | 0.021 | | -0.023 | | 0.021 | |
| rs7581907 | A | | G | | | | | 0.020 | 0.020 | | | 0.027 | | | 0.025 | | | | | 0.029 | | | 0.031 | | 0.029 | | 0.031 | | 0.028 | | 0.030 | |
| rs6436661 | C | | T | | | | | -0.033 | 0.024 | | | -0.013 | | | 0.029 | | | | | -0.034 | | | 0.038 | | 0.015 | | 0.036 | | -0.054 | | 0.036 | |
| rs7619139 | T | | A | | | | | 0.005 | 0.014 | | | 0.005 | | | 0.017 | | | | | -0.019 | | | 0.022 | | 0.023 | | 0.021 | | 0.001 | | 0.021 | |
| rs2526389 | C | | T | | | | | 0.018 | 0.014 | | | 0.015 | | | 0.017 | | | | | 0.033 | | | 0.022 | | 0.004 | | 0.022 | | 0.024 | | 0.021 | |
| rs2336558 | C | | T | | | | | -0.007 | 0.014 | | | -0.008 | | | 0.017 | | | | | -0.007 | | | 0.022 | | -0.002 | | 0.021 | | 0.000 | | 0.021 | |
| rs11708540 | G | | A | | | | | -0.036 | 0.020 | | | -0.046 | | | 0.024 | | | | | -0.064 | | | 0.030 | | -0.014 | | 0.030 | | -0.037 | | 0.029 | |
| rs55782528 | C | | A | | | | | -0.029 | 0.014 | | | -0.027 | | | 0.017 | | | | | -0.013 | | | 0.022 | | -0.034 | | 0.022 | | -0.039 | | 0.021 | |
| rs9861443 | A | | C | | | | | -0.028 | 0.016 | | | -0.025 | | | 0.019 | | | | | -0.022 | | | 0.025 | | -0.032 | | 0.024 | | -0.029 | | 0.023 | |
| rs13066686 | C | | A | | | | | 0.006 | 0.014 | | | 0.000 | | | 0.017 | | | | | -0.002 | | | 0.022 | | -0.003 | | 0.022 | | 0.016 | | 0.021 | |
| rs2918217 | C | | T | | | | | 0.023 | 0.020 | | | 0.052 | | | 0.025 | | | | | 0.031 | | | 0.032 | | 0.059 | | 0.031 | | -0.023 | | 0.030 | |
| rs73875019 | T | | A | | | | | -0.032 | 0.022 | | | -0.045 | | | 0.026 | | | | | -0.034 | | | 0.034 | | -0.057 | | 0.033 | | -0.055 | | 0.032 | |
| rs1320903 | G | | A | | | | | -0.003 | 0.015 | | | 0.001 | | | 0.018 | | | | | -0.005 | | | 0.023 | | 0.000 | | 0.022 | | -0.022 | | 0.022 | |
| rs10935143 | G | | A | | | | | -0.006 | 0.014 | | | -0.014 | | | 0.017 | | | | | -0.020 | | | 0.021 | | -0.015 | | 0.021 | | -0.003 | | 0.020 | |
| rs61789562 | T | | C | | | | | 0.023 | 0.022 | | | 0.020 | | | 0.026 | | | | | 0.037 | | | 0.034 | | -0.011 | | 0.033 | | 0.021 | | 0.032 | |
| rs1568488 | G | | C | | | | | 0.018 | 0.014 | | | 0.024 | | | 0.017 | | | | | 0.044 | | | 0.022 | | 0.010 | | 0.022 | | 0.016 | | 0.021 | |
| rs8192675 | T | | C | | | | | 0.018 | 0.015 | | | 0.026 | | | 0.018 | | | | | 0.017 | | | 0.023 | | 0.032 | | 0.023 | | 0.004 | | 0.022 | |
| rs894000 | T | | C | | | | | -0.011 | 0.015 | | | -0.016 | | | 0.018 | | | | | -0.010 | | | 0.023 | | -0.012 | | 0.022 | | -0.024 | | 0.021 | |
| rs55742087 | C | | T | | | | | -0.029 | 0.018 | | | -0.022 | | | 0.022 | | | | | -0.043 | | | 0.028 | | -0.001 | | 0.028 | | 0.000 | | 0.027 | |
| rs10938398 | G | | A | | | | | 0.017 | 0.014 | | | 0.014 | | | 0.017 | | | | | 0.015 | | | 0.022 | | 0.014 | | 0.021 | | 0.013 | | 0.020 | |
| rs2537860 | A | | C | | | | | -0.038 | 0.014 | | | -0.023 | | | 0.017 | | | | | -0.033 | | | 0.022 | | -0.017 | | 0.022 | | -0.054 | | 0.021 | |
| rs13107325 | C | | T | | | | | -0.009 | 0.026 | | | -0.003 | | | 0.032 | | | | | -0.012 | | | 0.040 | | -0.009 | | 0.039 | | 0.000 | | 0.039 | |
| rs1296328 | A | | C | | | | | -0.028 | 0.014 | | | -0.016 | | | 0.017 | | | | | -0.013 | | | 0.022 | | -0.027 | | 0.021 | | -0.039 | | 0.021 | |
| rs2307111 | T | | C | | | | | 0.023 | 0.014 | | | 0.021 | | | 0.017 | | | | | 0.014 | | | 0.022 | | 0.032 | | 0.021 | | 0.030 | | 0.021 | |
| rs7703782 | T | | A | | | | | -0.009 | 0.019 | | | -0.010 | | | 0.024 | | | | | -0.002 | | | 0.031 | | -0.026 | | 0.030 | | 0.013 | | 0.029 | |
| rs1459843 | C | | A | | | | | 0.009 | 0.014 | | | 0.000 | | | 0.017 | | | | | -0.004 | | | 0.022 | | -0.002 | | 0.021 | | 0.024 | | 0.020 | |
| rs2591496 | G | | A | | | | | -0.004 | 0.015 | | | -0.005 | | | 0.019 | | | | | -0.009 | | | 0.024 | | 0.002 | | 0.023 | | -0.028 | | 0.023 | |
| rs9379829 | C | | T | | | | | 0.033 | 0.016 | | | 0.049 | | | 0.020 | | | | | 0.055 | | | 0.025 | | 0.052 | | 0.025 | | 0.044 | | 0.024 | |
| rs2260051 | A | | T | | | | | -0.034 | 0.014 | | | -0.036 | | | 0.017 | | | | | -0.036 | | | 0.022 | | -0.037 | | 0.022 | | -0.050 | | 0.021 | |
| rs9277992 | G | | A | | | | | 0.011 | 0.018 | | | 0.000 | | | 0.021 | | | | | -0.004 | | | 0.027 | | -0.009 | | 0.027 | | -0.004 | | 0.026 | |
| rs9469899 | G | | A | | | | | -0.011 | 0.014 | | | -0.001 | | | 0.017 | | | | | 0.035 | | | 0.022 | | -0.025 | | 0.022 | | 0.045 | | 0.021 | |
| rs9471333 | C | | T | | | | | 0.020 | 0.014 | | | 0.019 | | | 0.017 | | | | | 0.019 | | | 0.022 | | 0.023 | | 0.021 | | 0.031 | | 0.021 | |
| rs3798519 | A | | C | | | | | 0.005 | 0.017 | | | 0.006 | | | 0.021 | | | | | 0.018 | | | 0.028 | | -0.006 | | 0.027 | | 0.005 | | 0.026 | |
| rs74621225 | A | | G | | | | | -0.023 | 0.021 | | | -0.018 | | | 0.025 | | | | | -0.007 | | | 0.032 | | -0.035 | | 0.031 | | -0.019 | | 0.030 | |
| rs9320823 | T | | C | | | | | -0.037 | 0.014 | | | -0.032 | | | 0.017 | | | | | 0.005 | | | 0.022 | | -0.065 | | 0.022 | | -0.032 | | 0.021 | |
| rs9478671 | A | | G | | | | | 0.021 | 0.017 | | | 0.014 | | | 0.020 | | | | | 0.036 | | | 0.026 | | 0.004 | | 0.026 | | 0.027 | | 0.025 | |
| rs34714518 | G | | A | | | | | 0.025 | 0.021 | | | 0.042 | | | 0.026 | | | | | 0.048 | | | 0.034 | | 0.046 | | 0.033 | | 0.038 | | 0.031 | |
| rs12666574 | G | | A | | | | | -0.036 | 0.014 | | | -0.041 | | | 0.018 | | | | | -0.030 | | | 0.022 | | -0.055 | | 0.022 | | -0.058 | | 0.021 | |
| rs62457529 | A | | G | | | | | 0.018 | 0.023 | | | 0.012 | | | 0.028 | | | | | -0.017 | | | 0.035 | | 0.038 | | 0.035 | | 0.041 | | 0.034 | |
| rs6962280 | A | | G | | | | | -0.032 | 0.014 | | | -0.019 | | | 0.017 | | | | | 0.006 | | | 0.022 | | -0.047 | | 0.021 | | -0.030 | | 0.020 | |
| rs17145600 | C | | T | | | | | 0.001 | 0.038 | | | 0.006 | | | 0.047 | | | | | -0.018 | | | 0.060 | | 0.005 | | 0.058 | | 0.041 | | 0.057 | |
| rs12538826 | T | | C | | | | | 0.000 | 0.021 | | | 0.001 | | | 0.026 | | | | | -0.007 | | | 0.033 | | 0.000 | | 0.033 | | -0.005 | | 0.032 | |
| rs10236214 | C | | T | | | | | -0.011 | 0.015 | | | -0.021 | | | 0.018 | | | | | -0.036 | | | 0.023 | | -0.004 | | 0.022 | | -0.010 | | 0.021 | |
| rs4840941 | A | | G | | | | | -0.008 | 0.015 | | | -0.015 | | | 0.018 | | | | | -0.025 | | | 0.023 | | -0.011 | | 0.022 | | -0.002 | | 0.022 | |
| rs9329197 | A | | T | | | | | 0.009 | 0.015 | | | 0.009 | | | 0.019 | | | | | 0.011 | | | 0.024 | | 0.003 | | 0.023 | | 0.003 | | 0.023 | |
| rs3750310 | G | | A | | | | | 0.009 | 0.015 | | | 0.012 | | | 0.019 | | | | | 0.001 | | | 0.024 | | 0.018 | | 0.023 | | 0.009 | | 0.023 | |
| rs11780420 | G | | A | | | | | 0.006 | 0.015 | | | 0.001 | | | 0.019 | | | | | 0.011 | | | 0.024 | | -0.013 | | 0.023 | | -0.001 | | 0.023 | |
| rs7827210 | G | | A | | | | | -0.022 | 0.014 | | | -0.031 | | | 0.017 | | | | | -0.026 | | | 0.022 | | -0.038 | | 0.022 | | -0.001 | | 0.021 | |
| rs35732620 | G | | T | | | | | -0.020 | 0.014 | | | -0.022 | | | 0.017 | | | | | -0.008 | | | 0.022 | | -0.040 | | 0.021 | | -0.014 | | 0.021 | |
| rs1812736 | G | | A | | | | | -0.014 | 0.018 | | | -0.003 | | | 0.022 | | | | | 0.009 | | | 0.028 | | -0.026 | | 0.028 | | -0.028 | | 0.027 | |
| rs72674843 | T | | C | | | | | 0.017 | 0.015 | | | 0.010 | | | 0.019 | | | | | -0.017 | | | 0.024 | | 0.030 | | 0.024 | | 0.002 | | 0.023 | |
| rs800526 | A | | C | | | | | -0.019 | 0.016 | | | -0.043 | | | 0.021 | | | | | 0.014 | | | 0.026 | | -0.089 | | 0.026 | | 0.015 | | 0.025 | |
| rs1412239 | C | | G | | | | | -0.024 | 0.015 | | | -0.015 | | | 0.018 | | | | | -0.023 | | | 0.023 | | -0.026 | | 0.023 | | -0.017 | | 0.022 | |
| rs10828247 | A | | G | | | | | -0.030 | 0.015 | | | -0.029 | | | 0.018 | | | | | -0.028 | | | 0.023 | | -0.036 | | 0.022 | | -0.041 | | 0.021 | |
| rs10824218 | A | | T | | | | | 0.000 | 0.014 | | | -0.006 | | | 0.017 | | | | | 0.001 | | | 0.022 | | -0.017 | | 0.021 | | 0.018 | | 0.021 | |
| rs10883026 | C | | T | | | | | 0.011 | 0.014 | | | -0.008 | | | 0.017 | | | | | 0.001 | | | 0.022 | | -0.013 | | 0.021 | | 0.027 | | 0.021 | |
| rs117597828 | C | | T | | | | | -0.017 | 0.016 | | | -0.011 | | | 0.020 | | | | | -0.013 | | | 0.026 | | -0.005 | | 0.025 | | -0.014 | | 0.025 | |
| rs4962671 | T | | C | | | | | 0.009 | 0.014 | | | 0.002 | | | 0.017 | | | | | -0.007 | | | 0.022 | | 0.019 | | 0.021 | | 0.025 | | 0.021 | |
| rs72867447 | C | | G | | | | | 0.018 | 0.014 | | | 0.005 | | | 0.017 | | | | | -0.002 | | | 0.022 | | -0.002 | | 0.021 | | 0.050 | | 0.020 | |
| rs6265 | C | | T | | | | | 0.017 | 0.017 | | | 0.011 | | | 0.021 | | | | | 0.015 | | | 0.027 | | 0.011 | | 0.026 | | 0.025 | | 0.025 | |
| rs1222216 | C | | T | | | | | 0.002 | 0.017 | | | 0.001 | | | 0.021 | | | | | -0.022 | | | 0.026 | | 0.025 | | 0.026 | | 0.013 | | 0.025 | |
| rs4755726 | T | | G | | | | | 0.001 | 0.015 | | | 0.012 | | | 0.018 | | | | | 0.007 | | | 0.023 | | 0.020 | | 0.023 | | -0.040 | | 0.022 | |
| rs12798028 | C | | T | | | | | -0.017 | 0.014 | | | -0.009 | | | 0.017 | | | | | -0.013 | | | 0.022 | | -0.013 | | 0.021 | | -0.013 | | 0.021 | |
| rs7940691 | C | | T | | | | | 0.025 | 0.014 | | | 0.035 | | | 0.018 | | | | | 0.026 | | | 0.022 | | 0.050 | | 0.022 | | 0.028 | | 0.021 | |
| rs10898317 | C | | T | | | | | 0.002 | 0.014 | | | 0.003 | | | 0.017 | | | | | 0.005 | | | 0.022 | | -0.013 | | 0.021 | | -0.017 | | 0.021 | |
| rs55726687 | G | | A | | | | | -0.012 | 0.017 | | | -0.023 | | | 0.021 | | | | | -0.017 | | | 0.027 | | -0.028 | | 0.026 | | -0.021 | | 0.026 | |
| rs76895963 | T | | G | | | | | -0.232 | 0.056 | | | -0.252 | | | 0.068 | | | | | -0.259 | | | 0.087 | | -0.210 | | 0.085 | | -0.242 | | 0.083 | |
| rs7132908 | G | | A | | | | | 0.001 | 0.014 | | | 0.005 | | | 0.017 | | | | | 0.006 | | | 0.022 | | 0.004 | | 0.022 | | 0.018 | | 0.021 | |
| rs4759228 | G | | C | | | | | 0.015 | 0.016 | | | 0.024 | | | 0.020 | | | | | 0.024 | | | 0.025 | | 0.036 | | 0.025 | | 0.006 | | 0.024 | |
| rs7308188 | T | | C | | | | | 0.008 | 0.015 | | | 0.011 | | | 0.019 | | | | | -0.010 | | | 0.024 | | 0.021 | | 0.024 | | -0.002 | | 0.023 | |
| rs6490030 | C | | A | | | | | 0.028 | 0.014 | | | 0.031 | | | 0.017 | | | | | 0.029 | | | 0.022 | | 0.034 | | 0.022 | | 0.029 | | 0.021 | |
| rs147730268 | G | | T | | | | | 0.024 | 0.028 | | | 0.016 | | | 0.034 | | | | | 0.026 | | | 0.044 | | -0.001 | | 0.043 | | 0.021 | | 0.042 | |
| rs11619722 | T | | C | | | | | 0.003 | 0.015 | | | 0.000 | | | 0.019 | | | | | 0.004 | | | 0.024 | | -0.006 | | 0.023 | | -0.012 | | 0.023 | |
| rs61954177 | G | | C | | | | | 0.000 | 0.015 | | | 0.016 | | | 0.018 | | | | | 0.019 | | | 0.023 | | 0.013 | | 0.023 | | 0.004 | | 0.022 | |
| rs4477562 | C | | T | | | | | 0.011 | 0.020 | | | 0.042 | | | 0.025 | | | | | 0.066 | | | 0.033 | | 0.017 | | 0.031 | | -0.007 | | 0.030 | |
| rs9317002 | C | | A | | | | | -0.009 | 0.014 | | | -0.010 | | | 0.017 | | | | | -0.010 | | | 0.021 | | -0.020 | | 0.021 | | -0.016 | | 0.020 | |
| rs7983454 | T | | C | | | | | 0.001 | 0.014 | | | 0.010 | | | 0.017 | | | | | 0.013 | | | 0.021 | | 0.005 | | 0.021 | | -0.005 | | 0.020 | |
| rs9522279 | C | | T | | | | | -0.019 | 0.014 | | | -0.029 | | | 0.017 | | | | | -0.029 | | | 0.022 | | -0.039 | | 0.021 | | -0.008 | | 0.021 | |
| rs10132280 | C | | A | | | | | -0.012 | 0.015 | | | -0.031 | | | 0.021 | | | | | -0.034 | | | 0.027 | | -0.020 | | 0.027 | | 0.003 | | 0.027 | |
| rs9788550 | G | | C | | | | | 0.008 | 0.016 | | | 0.045 | | | 0.023 | | | | | 0.033 | | | 0.029 | | 0.058 | | 0.029 | | -0.018 | | 0.029 | |
| rs2143975 | C | | G | | | | | 0.021 | 0.014 | | | 0.033 | | | 0.020 | | | | | 0.042 | | | 0.025 | | 0.025 | | 0.025 | | 0.037 | | 0.025 | |
| rs10131761 | T | | A | | | | | 0.005 | 0.018 | | | 0.037 | | | 0.025 | | | | | 0.034 | | | 0.033 | | 0.016 | | 0.033 | | -0.023 | | 0.033 | |
| rs4898556 | A | | C | | | | | -0.005 | 0.014 | | | -0.002 | | | 0.020 | | | | | 0.005 | | | 0.025 | | -0.011 | | 0.025 | | -0.039 | | 0.025 | |
| rs217669 | T | | C | | | | | 0.012 | 0.015 | | | 0.010 | | | 0.023 | | | | | 0.060 | | | 0.029 | | -0.021 | | 0.025 | | 0.018 | | 0.029 | |
| rs8008910 | G | | A | | | | | 0.001 | 0.017 | | | 0.007 | | | 0.023 | | | | | 0.039 | | | 0.032 | | -0.022 | | 0.031 | | -0.006 | | 0.029 | |
| rs8008772 | A | | T | | | | | 0.003 | 0.016 | | | 0.016 | | | 0.024 | | | | | 0.014 | | | 0.030 | | 0.018 | | 0.030 | | 0.001 | | 0.030 | |
| rs1887197 | C | | T | | | | | 0.002 | 0.014 | | | 0.008 | | | 0.019 | | | | | -0.019 | | | 0.025 | | 0.001 | | 0.026 | | 0.017 | | 0.023 | |
| rs61992671 | A | | G | | | | | 0.016 | 0.015 | | | 0.026 | | | 0.022 | | | | | 0.022 | | | 0.027 | | 0.021 | | 0.027 | | 0.029 | | 0.027 | |
| rs11631651 | A | | C | | | | | 0.013 | 0.027 | | | 0.023 | | | 0.033 | | | | | 0.060 | | | 0.042 | | -0.004 | | 0.041 | | -0.027 | | 0.039 | |
| rs57488047 | T | | C | | | | | 0.021 | 0.014 | | | 0.031 | | | 0.017 | | | | | 0.028 | | | 0.022 | | 0.033 | | 0.021 | | 0.006 | | 0.020 | |
| rs2238435 | C | | G | | | | | -0.010 | 0.014 | | | -0.004 | | | 0.017 | | | | | -0.014 | | | 0.022 | | -0.002 | | 0.021 | | -0.015 | | 0.021 | |
| rs1990573 | A | | G | | | | | 0.022 | 0.015 | | | 0.025 | | | 0.018 | | | | | 0.026 | | | 0.023 | | 0.029 | | 0.023 | | 0.040 | | 0.022 | |
| rs8054079 | C | | T | | | | | -0.005 | 0.020 | | | -0.001 | | | 0.025 | | | | | 0.023 | | | 0.032 | | -0.021 | | 0.031 | | 0.002 | | 0.030 | |
| rs27741 | G | | A | | | | | 0.002 | 0.014 | | | -0.006 | | | 0.017 | | | | | 0.003 | | | 0.022 | | -0.017 | | 0.022 | | 0.007 | | 0.021 | |
| rs62048402 | G | | A | | | | | 0.000 | 0.014 | | | 0.002 | | | 0.017 | | | | | -0.005 | | | 0.022 | | 0.009 | | 0.021 | | 0.003 | | 0.020 | |
| rs12923231 | C | | T | | | | | 0.016 | 0.014 | | | 0.012 | | | 0.017 | | | | | 0.015 | | | 0.022 | | 0.011 | | 0.021 | | 0.009 | | 0.021 | |
| rs12149660 | G | | A | | | | | -0.014 | 0.023 | | | 0.009 | | | 0.028 | | | | | 0.027 | | | 0.037 | | -0.007 | | 0.036 | | -0.002 | | 0.035 | |
| rs3923783 | C | | A | | | | | 0.029 | 0.018 | | | 0.029 | | | 0.022 | | | | | 0.032 | | | 0.028 | | 0.018 | | 0.028 | | 0.052 | | 0.027 | |
| rs9901404 | A | | G | | | | | 0.017 | 0.016 | | | 0.015 | | | 0.019 | | | | | 0.035 | | | 0.025 | | 0.013 | | 0.024 | | 0.005 | | 0.023 | |
| rs12941009 | C | | T | | | | | 0.001 | 0.014 | | | -0.004 | | | 0.017 | | | | | 0.004 | | | 0.022 | | -0.015 | | 0.022 | | 0.021 | | 0.021 | |
| rs11079849 | C | | T | | | | | -0.003 | 0.015 | | | 0.002 | | | 0.018 | | | | | -0.014 | | | 0.023 | | 0.021 | | 0.022 | | -0.007 | | 0.022 | |
| rs11150745 | A | | G | | | | | 0.011 | 0.015 | | | 0.006 | | | 0.018 | | | | | 0.009 | | | 0.023 | | 0.018 | | 0.023 | | 0.030 | | 0.022 | |
| rs1652376 | G | | T | | | | | 0.017 | 0.014 | | | 0.016 | | | 0.017 | | | | | -0.001 | | | 0.022 | | 0.024 | | 0.021 | | 0.029 | | 0.021 | |
| rs7232171 | G | | T | | | | | 0.013 | 0.014 | | | 0.013 | | | 0.017 | | | | | 0.014 | | | 0.022 | | 0.016 | | 0.021 | | -0.013 | | 0.020 | |
| rs58243949 | C | | T | | | | | -0.002 | 0.017 | | | 0.002 | | | 0.021 | | | | | 0.012 | | | 0.027 | | 0.003 | | 0.026 | | 0.013 | | 0.025 | |
| rs7240682 | C | | G | | | | | -0.019 | 0.016 | | | -0.020 | | | 0.020 | | | | | -0.022 | | | 0.025 | | -0.012 | | 0.025 | | -0.016 | | 0.024 | |
| rs8112818 | A | | G | | | | | 0.035 | 0.014 | | | 0.051 | | | 0.017 | | | | | 0.065 | | | 0.022 | | 0.036 | | 0.022 | | 0.020 | | 0.021 | |
| rs10423928 | T | | A | | | | | 0.006 | 0.017 | | | -0.006 | | | 0.020 | | | | | -0.012 | | | 0.026 | | 0.001 | | 0.026 | | 0.007 | | 0.025 | |
| rs3810291 | G | | A | | | | | 0.020 | 0.015 | | | 0.028 | | | 0.018 | | | | | 0.015 | | | 0.023 | | 0.048 | | 0.023 | | 0.008 | | 0.022 | |
| rs6054427 | G | | A | | | | | 0.025 | 0.014 | | | 0.015 | | | 0.017 | | | | | -0.002 | | | 0.022 | | 0.027 | | 0.022 | | 0.032 | | 0.021 | |
| rs6096886 | A | | G | | | | | 0.028 | 0.017 | | | 0.025 | | | 0.021 | | | | | 0.028 | | | 0.027 | | 0.019 | | 0.027 | | 0.026 | | 0.026 | |
| rs9977825 | T | | C | | | | | -0.015 | 0.014 | | | -0.032 | | | 0.018 | | | | | -0.034 | | | 0.023 | | -0.025 | | 0.022 | | -0.017 | | 0.021 | |
| rs17421586 | T | | A | | | | | 0.007 | 0.014 | | | -0.006 | | | 0.018 | | | | | -0.019 | | | 0.023 | | -0.003 | | 0.022 | | 0.003 | | 0.022 | |
| Adult body size SNPs (women only) | | | | | | | | | | | | | | | | | | | | | | | | | | | | | | | | |
| SNP | EA | OA | | | beta_ca | | | | se_ca | beta_co | | | | | | | se_co | beta_prox | | | | | se_prox | | beta_dist | | se_dist | | beta_re | | se_re | |
| rs74892851 | C | A | | | 0.008 | | | | 0.017 | 0.020 | | | | | | | 0.021 | 0.042 | | | | | 0.025 | | -0.017 | | 0.028 | | 0.004 | | 0.028 | |
| rs78886584 | A | G | | | -0.024 | | | | 0.016 | -0.006 | | | | | | | 0.019 | -0.007 | | | | | 0.023 | | -0.009 | | 0.025 | | -0.060 | | 0.027 | |
| rs72660086 | T | G | | | 0.005 | | | | 0.017 | 0.028 | | | | | | | 0.020 | 0.027 | | | | | 0.024 | | 0.037 | | 0.027 | | -0.018 | | 0.028 | |
| rs12144626 | T | C | | | -0.013 | | | | 0.014 | -0.009 | | | | | | | 0.017 | -0.009 | | | | | 0.020 | | -0.010 | | 0.022 | | -0.066 | | 0.023 | |
| rs1494461 | C | T | | | -0.002 | | | | 0.015 | -0.012 | | | | | | | 0.017 | -0.011 | | | | | 0.021 | | -0.015 | | 0.024 | | -0.005 | | 0.025 | |
| rs12140153 | G | T | | | 0.028 | | | | 0.027 | 0.038 | | | | | | | 0.031 | 0.080 | | | | | 0.039 | | -0.038 | | 0.042 | | -0.026 | | 0.044 | |
| rs2815757 | C | T | | | -0.016 | | | | 0.018 | -0.022 | | | | | | | 0.021 | 0.005 | | | | | 0.026 | | -0.055 | | 0.029 | | 0.005 | | 0.030 | |
| rs1514173 | C | T | | | 0.009 | | | | 0.014 | 0.010 | | | | | | | 0.016 | 0.007 | | | | | 0.020 | | 0.002 | | 0.022 | | -0.010 | | 0.023 | |
| rs34517439 | C | A | | | -0.007 | | | | 0.024 | -0.028 | | | | | | | 0.028 | -0.035 | | | | | 0.034 | | -0.019 | | 0.038 | | -0.015 | | 0.039 | |
| rs10922911 | C | T | | | 0.009 | | | | 0.014 | 0.006 | | | | | | | 0.017 | -0.008 | | | | | 0.020 | | 0.017 | | 0.022 | | 0.030 | | 0.023 | |
| rs653958 | A | G | | | -0.025 | | | | 0.014 | -0.027 | | | | | | | 0.017 | -0.013 | | | | | 0.021 | | -0.035 | | 0.023 | | -0.019 | | 0.025 | |
| rs75641275 | A | C | | | 0.025 | | | | 0.020 | 0.015 | | | | | | | 0.024 | 0.031 | | | | | 0.029 | | 0.007 | | 0.032 | | 0.029 | | 0.033 | |
| rs41279738 | T | G | | | 0.002 | | | | 0.042 | 0.002 | | | | | | | 0.049 | -0.015 | | | | | 0.060 | | -0.020 | | 0.066 | | 0.029 | | 0.069 | |
| rs12033257 | A | G | | | 0.017 | | | | 0.014 | 0.007 | | | | | | | 0.017 | 0.025 | | | | | 0.021 | | -0.013 | | 0.023 | | 0.039 | | 0.024 | |
| rs3753639 | T | C | | | -0.004 | | | | 0.016 | -0.008 | | | | | | | 0.019 | 0.000 | | | | | 0.023 | | -0.025 | | 0.026 | | -0.041 | | 0.027 | |
| rs61813324 | C | T | | | -0.063 | | | | 0.024 | -0.055 | | | | | | | 0.028 | -0.058 | | | | | 0.034 | | -0.058 | | 0.038 | | -0.076 | | 0.040 | |
| rs539515 | A | C | | | -0.017 | | | | 0.017 | 0.003 | | | | | | | 0.021 | 0.007 | | | | | 0.025 | | 0.005 | | 0.028 | | -0.039 | | 0.029 | |
| rs815163 | T | C | | | 0.001 | | | | 0.014 | -0.008 | | | | | | | 0.016 | -0.001 | | | | | 0.020 | | -0.022 | | 0.022 | | 0.016 | | 0.023 | |
| rs2678204 | T | G | | | -0.027 | | | | 0.015 | -0.037 | | | | | | | 0.017 | -0.043 | | | | | 0.021 | | -0.033 | | 0.024 | | -0.003 | | 0.025 | |
| rs2994320 | A | G | | | -0.019 | | | | 0.017 | -0.042 | | | | | | | 0.020 | -0.013 | | | | | 0.025 | | -0.083 | | 0.027 | | 0.002 | | 0.029 | |
| rs62106258 | T | C | | | 0.041 | | | | 0.036 | 0.060 | | | | | | | 0.042 | 0.027 | | | | | 0.051 | | 0.121 | | 0.058 | | 0.000 | | 0.059 | |
| rs6548237 | A | C | | | -0.004 | | | | 0.018 | -0.007 | | | | | | | 0.021 | -0.033 | | | | | 0.026 | | 0.027 | | 0.028 | | 0.006 | | 0.030 | |
| rs6749422 | C | G | | | -0.009 | | | | 0.014 | -0.003 | | | | | | | 0.016 | -0.006 | | | | | 0.020 | | -0.006 | | 0.022 | | -0.027 | | 0.023 | |
| rs34606703 | G | A | | | -0.009 | | | | 0.014 | -0.016 | | | | | | | 0.017 | -0.028 | | | | | 0.021 | | -0.009 | | 0.023 | | -0.022 | | 0.024 | |
| rs13420048 | C | A | | | 0.021 | | | | 0.015 | 0.022 | | | | | | | 0.017 | 0.033 | | | | | 0.021 | | 0.012 | | 0.023 | | 0.033 | | 0.024 | |
| rs6545468 | C | G | | | 0.001 | | | | 0.014 | 0.004 | | | | | | | 0.016 | -0.014 | | | | | 0.020 | | 0.017 | | 0.022 | | 0.008 | | 0.023 | |
| rs4671328 | T | G | | | -0.002 | | | | 0.014 | -0.001 | | | | | | | 0.016 | 0.002 | | | | | 0.020 | | 0.000 | | 0.022 | | 0.024 | | 0.023 | |
| rs13416992 | A | C | | | 0.006 | | | | 0.014 | 0.007 | | | | | | | 0.017 | -0.003 | | | | | 0.020 | | 0.020 | | 0.022 | | 0.027 | | 0.023 | |
| rs10192894 | A | G | | | -0.014 | | | | 0.014 | -0.026 | | | | | | | 0.016 | -0.036 | | | | | 0.020 | | -0.017 | | 0.022 | | -0.025 | | 0.023 | |
| rs12477088 | T | C | | | -0.004 | | | | 0.014 | -0.014 | | | | | | | 0.016 | -0.032 | | | | | 0.020 | | 0.011 | | 0.022 | | -0.025 | | 0.023 | |
| rs11691869 | C | A | | | 0.015 | | | | 0.014 | 0.018 | | | | | | | 0.017 | -0.008 | | | | | 0.021 | | 0.043 | | 0.023 | | 0.019 | | 0.024 | |
| rs7602120 | C | T | | | -0.016 | | | | 0.014 | -0.016 | | | | | | | 0.016 | -0.015 | | | | | 0.020 | | -0.021 | | 0.022 | | -0.012 | | 0.023 | |
| rs1083472 | C | G | | | 0.028 | | | | 0.014 | 0.030 | | | | | | | 0.016 | 0.034 | | | | | 0.020 | | 0.023 | | 0.022 | | 0.034 | | 0.023 | |
| rs4482463 | C | A | | | -0.024 | | | | 0.024 | -0.021 | | | | | | | 0.029 | -0.030 | | | | | 0.036 | | -0.018 | | 0.040 | | -0.082 | | 0.042 | |
| rs4673553 | T | G | | | 0.004 | | | | 0.014 | 0.004 | | | | | | | 0.016 | 0.004 | | | | | 0.020 | | 0.013 | | 0.022 | | 0.010 | | 0.023 | |
| rs2433733 | G | A | | | -0.009 | | | | 0.014 | -0.007 | | | | | | | 0.017 | -0.005 | | | | | 0.021 | | -0.009 | | 0.023 | | -0.014 | | 0.024 | |
| rs113706999 | T | A | | | -0.047 | | | | 0.056 | -0.028 | | | | | | | 0.065 | -0.114 | | | | | 0.078 | | 0.065 | | 0.091 | | -0.219 | | 0.089 | |
| rs72906474 | G | T | | | 0.015 | | | | 0.015 | 0.019 | | | | | | | 0.017 | 0.011 | | | | | 0.021 | | 0.030 | | 0.024 | | 0.027 | | 0.025 | |
| rs9843653 | T | C | | | -0.015 | | | | 0.014 | -0.010 | | | | | | | 0.016 | -0.005 | | | | | 0.020 | | -0.011 | | 0.022 | | -0.046 | | 0.023 | |
| rs6774533 | C | T | | | -0.001 | | | | 0.015 | -0.006 | | | | | | | 0.018 | -0.028 | | | | | 0.022 | | 0.029 | | 0.025 | | -0.007 | | 0.026 | |
| rs13066308 | C | G | | | -0.010 | | | | 0.014 | -0.015 | | | | | | | 0.017 | -0.002 | | | | | 0.021 | | -0.026 | | 0.023 | | -0.013 | | 0.024 | |
| rs1454687 | C | G | | | -0.009 | | | | 0.014 | -0.008 | | | | | | | 0.016 | 0.006 | | | | | 0.020 | | -0.019 | | 0.022 | | 0.001 | | 0.023 | |
| rs13081671 | C | T | | | -0.001 | | | | 0.016 | -0.012 | | | | | | | 0.018 | -0.029 | | | | | 0.022 | | 0.004 | | 0.025 | | -0.007 | | 0.026 | |
| rs2035936 | G | T | | | -0.033 | | | | 0.029 | -0.041 | | | | | | | 0.037 | -0.089 | | | | | 0.044 | | 0.028 | | 0.051 | | -0.123 | | 0.051 | |
| rs529200 | A | G | | | 0.013 | | | | 0.014 | 0.012 | | | | | | | 0.016 | -0.009 | | | | | 0.020 | | 0.037 | | 0.022 | | -0.003 | | 0.023 | |
[truncated: 120,722 more chars]
